# Gene expression during larval caste determination and differentiation in intermediately eusocial bumblebees, and a comparative analysis with advanced eusocial honeybees

**DOI:** 10.1111/mec.15752

**Published:** 2021-01-07

**Authors:** David H. Collins, Anders Wirén, Marjorie Labédan, Michael Smith, David C. Prince, Irina Mohorianu, Tamas Dalmay, Andrew F. G. Bourke

**Affiliations:** ^1^ School of Biological Sciences University of East Anglia Norwich UK; ^2^ School of Medical Sciences Faculty of Medicine and Health Örebro University Örebro Sweden; ^3^ Department of Ecology and Evolution University of Lausanne Lausanne Switzerland; ^4^ Jeffrey Cheah Biomedical Centre WT‐MRC Cambridge Stem Cell Institute Cambridge UK

**Keywords:** *Bombus terrestris*, caste determination, caste‐associated gene expression, eusociality, larval development, mRNA‐seq

## Abstract

The queen‐worker caste system of eusocial insects represents a prime example of developmental polyphenism (environmentally‐induced phenotypic polymorphism) and is intrinsic to the evolution of advanced eusociality. However, the comparative molecular basis of larval caste determination and subsequent differentiation in the eusocial Hymenoptera remains poorly known. To address this issue within bees, we profiled caste‐associated gene expression in female larvae of the intermediately eusocial bumblebee *Bombus terrestris*. In *B*. *terrestris*, female larvae experience a queen‐dependent period during which their caste fate as adults is determined followed by a nutrition‐sensitive period also potentially affecting caste fate but for which the evidence is weaker. We used mRNA‐seq and qRT‐PCR validation to isolate genes differentially expressed between each caste pathway in larvae at developmental stages before and after each of these periods. We show that differences in gene expression between caste pathways are small in totipotent larvae, then peak after the queen‐dependent period. Relatively few novel (i.e., taxonomically‐restricted) genes were differentially expressed between castes, though novel genes were significantly enriched in late‐instar larvae in the worker pathway. We compared sets of caste‐associated genes in *B*. *terrestris* with those reported from the advanced eusocial honeybee, *Apis mellifera*, and found significant but relatively low levels of overlap of gene lists between the two species. These results suggest both the existence of low numbers of shared toolkit genes and substantial divergence in caste‐associated genes between *Bombus* and the advanced eusocial *Apis* since their last common eusocial ancestor.

## 1 INTRODUCTION

The caste systems of eusocial Hymenoptera, in which females develop as reproductive queens or nonreproductive workers, represent one of the best characterised examples of developmental polyphenism. In these, multiple phenotypes can develop from a single, species‐specific genome via differing environmental inputs (Kapheim, 2019; Sumner et al., 2018; West‐Eberhard, 2003; Wheeler, 1986). Understanding the mechanistic and evolutionary basis of the polyphenism represented by the castes of eusocial species is therefore central to understanding the generation of adaptive diversity via polyphenism. It also serves to illuminate the basis of the major transition entailed by the evolution of eusociality (Bourke, 2011). For these reasons, several studies have isolated caste‐associated genes in eusocial Hymenoptera using microarrays or mRNA‐seq to profile adult queens and workers (Feldmeyer et al., 2014; Ferreira et al., 2013; Grozinger et al., 2007; Harrison et al., 2015; Jones et al., 2017; Kapheim et al., 2020; Niu et al., 2014; Toth et al., 2010; Woodard et al., 2014).

Caste determination in eusocial Hymenoptera represents the branching point when diploid females cease being totipotent (i.e., capable of becoming a queen or worker) and their caste fate becomes fixed. Caste differentiation describes the developmental processes occurring along each caste‐specific pathway. In species in which caste determination occurs in larvae, relatively few studies have profiled gene expression changes across sensitive periods of caste determination. To do so is important because genes isolated from such studies are likely to play key roles in the genetic pathways underpinning eusocial castes.

Genes associated with caste determination and/or differentiation in eusocial species have been hypothesised to fall into two broad categories: (a) Novel genes, i.e., taxonomically‐restricted genes that confer the convergent traits associated with eusociality in each independent eusocial lineage (e.g., Johnson & Tsutsui, 2011; Sumner, 2015, 2014); and (b) toolkit genes, i.e., highly conserved genes that have been co‐opted to underpin eusocial traits in repeated origins of eusociality (e.g., Toth & Robinson, 2007). Previous studies have variously demonstrated a role in caste evolution for novel genes (Berens et al., 2015; Chen et al., 2012; Feldmeyer et al., 2014; Ferreira et al., 2013; Jasper et al., 2015; Johnson & Tsutsui, 2011; Patalano et al., 2015) and conserved genes (Rehan et al., 2014; Woodard et al., 2011, 2014), and it now appears probable that both classes of genes are involved in the evolution of eusociality (Kapheim, 2016; Qiu et al., 2018; Toth & Rehan, 2017; Warner et al., 2019). However, the full extent to which genes associated with larval caste determination and/or differentiation are made up of novel genes, toolkit genes, or a combination of both, remains an open issue.

The honeybee, *Apis mellifera*, and bumblebee, *Bombus terrestris*, are likely to be informative for isolating caste‐associated genes because a wealth of genomic resources are available for them (Elsik et al., 2014; Sadd et al., 2015) and their physiological mechanisms of caste determination and/or differentiation are relatively well characterised (Amsalem, Grozinger, et al., 2015; Winston, 1987). In addition, *A*. *mellifera* and *B*. *terrestris* share a common eusocial ancestor within the corbiculate bees (Bossert et al., 2019; Cardinal & Danforth, 2011) while also exhibiting key differences (Holland & Bloch, 2020). *A*. *mellifera* is an advanced eusocial species, with queens and workers having distinct morphologies and reproductive anatomies (e.g., *Apis* queens have 150–180 ovarioles per ovary whereas workers cannot mate and have 2–12 ovarioles per ovary; Winston, 1987). By contrast, *B*. *terrestris* is intermediately eusocial, in that queens and workers have similar morphologies but workers cannot mate, and queens and workers each have four ovarioles per ovary (Amsalem, Grozinger, et al., 2015). The castes of these species differ in other ways; for example, *B*. *terrestris* queens undergo diapause (Amsalem, Galbraith, et al., 2015) whereas *A*. *mellifera* queens do not (Winston, 1987). While there has been long‐standing interest in characterising caste‐associated gene expression in eusocial Hymenopteran larvae (e.g., Barchuk et al., 2007; Corona et al., 1999; Evans & Wheeler, 1999, 2001), the only studies that have used high‐throughput approaches to investigate gene expression associated with sensitive periods of larval caste determination and subsequent differentiation in eusocial Hymenoptera have been conducted in advanced eusocial species, including *A*. *mellifera* (Cameron et al., 2013; Chen et al., 2012; He et al., 2017; Warner et al., 2019). Hence, investigating genes associated with sensitive periods of larval caste determination in *B*. *terrestris* extends the range of species for which such genes have been profiled. It also permits, via a comparison with *A*. *mellifera*, a study of the caste‐associated gene expression differences between species that have and have not undergone a transition to advanced eusociality.

In *B*. *terrestris*, female larvae develop through four instars and are thought to undergo two sensitive periods affecting caste determination (Figure S1). In the first of these, totipotent first/second‐instar larvae undergo a “queen‐dependent period” of caste determination approximately 3.5–5 days after hatching (Cnaani et al., 2000; Röseler, 1970), since larvae in the presence of the colony queen before this period become worker‐destined. This has been hypothesised to occur via exposure of the young larvae to an unidentified queen‐produced pheromone (Amsalem, Grozinger, et al., 2015; Röseler, 1970). Later in the colony cycle, the production of this pheromone is downregulated, thereby inducing totipotent larvae to develop as queens. The second sensitive period is less strongly supported and is hypothesised to occur in the fourth instar, when queen‐destined larvae eat significantly more than worker‐destined larvae (Ribeiro et al., 1999) and if underfed may revert to a worker‐like phenotype (Pereboom et al., 2003). This is suggestive of a “nutrition‐sensitive period” of caste determination (Röseler, 1991). In this scheme, the earlier, queen‐dependent period is the most consequential, since it causes determination on the worker caste pathway (for larvae in the presence of the queen) to become fixed (Amsalem, Grozinger, et al., 2015). The later, nutrition‐sensitive period appears less important but exerts a potential influence on caste fate in queen‐destined larvae up to their fourth instar, by which stage queen‐destined larvae are approximately four times the size of worker‐destined larvae (Cnaani et al., 1997). Caste determination and/or differentiation is also associated with two peaks in juvenile hormone (JH) level in the second and third instar in queen‐destined larvae, the first of which broadly corresponds to the queen‐dependent period (Cnaani et al., 2000). JH is essential for queen differentiation in *B*. *terrestris*, and JH application can cause worker‐destined larvae to become queen destined (Bortolotti et al., 2001). Overall, therefore, the physiological mechanisms of *B*. *terrestris* caste determination are relatively well understood, but the genetic mechanisms are not.

Previous studies of differential gene expression in *B*. *terrestris* larvae have focussed on relatively few genes (Collins et al., 2017; Pereboom et al., 2005) or have isolated genes and gene variants in a single larval caste pathway (Colgan et al., 2011; Harrison et al., 2015; Price et al., 2018). For example, Collins et al. (2017) used sRNA‐seq in *B*. *terrestris* to identify a mirtron (*Bte‐miR‐6001*) associated with caste differentiation that was expressed from the intron of a protein‐coding gene, *vitellogenin‐like*, suggesting that *Bte‐miR‐6001* and *vitellogenin‐like* could have a role in caste differentiation in this species.

This study therefore had two main aims. The first was to profile gene expression differences between queen‐ and worker‐destined larvae of *B*. *terrestris* at developmental stages straddling the two sensitive periods. Using mRNA‐seq, we profiled larvae before the queen‐dependent period (first/second‐instar larvae), between the queen‐dependent period and nutrition‐sensitive period (third‐instar larvae), and after the nutrition‐sensitive period (fourth‐instar larvae). We term these “early‐”, “mid‐”, and “late‐instar” larval stages, respectively. In general, we refer to genes isolated by these procedures as genes potentially affecting caste determination and/or differentiation. However, we note that for the reasons described above, genes differentially expressed between caste pathways in late‐instar larvae are most likely to be associated with caste differentiation, whereas genes most likely to affect caste determination are those differentially expressed during the queen‐dependent period, i.e., immediately before the mid‐instar larvae were collected. We used gene ontology (GO) analyses and investigations of individual genes and gene families to identify key caste‐associated processes and to select the subset of genes differentially expressed in the mRNA‐seq data that would be most likely to affect caste determination and/or differentiation. We then validated expression differences in these genes using qRT‐PCR. Within our first aim, we tested three interrelated hypotheses regarding caste‐associated gene expression in *B*. *terrestris*. Hypothesis 1 was that differential gene expression between queen‐ and worker‐destined larvae is absent in early‐instar larvae (as they are totipotent), greater in mid‐instar larvae (as they have embarked on one of the caste pathways) and greatest in late‐instar larvae (as they show the greatest phenotypic differences). Hypothesis 2 provided a test of the novel genes hypothesis, which predicts that a relatively high proportion of caste‐associated genes are novel, and that novel and unannotated genes are over‐represented in the worker pathway (e.g., Sumner, 2015, 2014). Hypothesis 3 involved testing previously proposed caste roles for specific genes in bees. Specifically, the hypothesis proposed that the genes *DNA methyl transferase 3B* (*Dnmt3*) and *vitellogenin‐like* are upregulated in the queen pathway. This was because the gene *Dnmt3* is an essential component of methylation and previous studies have suggested roles for methylation in queen determination and/or differentiation in *A*. *mellifera* and in the control of reproduction in *B*. *terrestris* workers (Amarasinghe et al., 2014; Kucharski et al., 2008; Marshall et al., 2019). *Vitellogenin‐like*, as described above, contains a mirtron sequence strongly associated with queen development in *B*. *terrestris* (Collins et al., 2017).

Our second aim was to compare expression data from queen‐ and worker‐destined larvae in *B*. *terrestris* from the current study with similar data from *A*. *mellifera* (Cameron et al., 2013; He et al., 2017). The toolkit genes hypothesis predicts that there should be a high degree of overlap in the caste‐associated gene lists of both species, especially as the two species share a common eusocial ancestor. As described, comparing differential gene expression across caste pathways in these two lineages also potentially illuminates the molecular mechanisms involved in the transition to advanced eusociality. As *B*. *terrestris* and *A*. *mellifera* also differ in the ability of their queens to diapause, we also used data from a previous mRNA‐seq study on diapause in *B*. *terrestris* (Amsalem, Galbraith, et al., 2015) to test whether enrichment of caste‐associated genes with diapause‐associated genes in *B*. *terrestris* explains any contrasts with *A*. *mellifera*. Overall, this is the first study to use next‐generation sequencing (plus validation) to profile the gene expression differences associated with the sensitive periods of larval caste determination in *B*. *terrestris*, and to compare such differences in larval caste pathways across separate eusocial taxa differing in their degree of eusociality (*Bombus* and *Apis*).

## 2 MATERIALS AND METHODS

### 2.1 Caste‐associated genes in *Bombus terrestris* larvae

#### 2.1.1 Sample collection

We obtained samples of queen‐destined larvae (by removing colony queens from a subset of colonies) and worker‐destined larvae (by retaining colony queens in a subset of colonies) of *B*. *terrestris*. (Detailed methods are available in Supporting information and summarised in Figure S2.) To address our first aim, we collected queen‐ and worker‐destined larvae at three different stages to produce samples for mRNA‐seq (Figure S3; Table S1). These consisted of: (a) Early‐instar queen‐destined larvae (EQ, 63 larvae from three colonies, representing three biological replicates for mRNA‐seq, labelled EQ1‐3) and early‐instar worker‐destined larvae (EW, 104 larvae from three colonies, representing three biological replicates for mRNA‐seq, EW1‐3); (b) mid‐instar queen‐destined larvae (MQ, 20 larvae from four colonies, representing four biological replicates for mRNA‐seq, MQ1‐4) and mid‐instar worker‐destined larvae (MW, 33 larvae from three colonies, representing three biological replicates for mRNA‐seq, MW1‐4); and (c) late‐instar queen‐destined larvae (LQ, 42 larvae from four colonies, representing four biological replicates for mRNA‐seq, LQ1‐4), and late‐instar worker‐destined larvae (LW, 33 larvae from three colonies, representing three biological replicates for mRNA‐seq, LW1‐3). Using an independent sample of colonies, we also collected 36 larvae (six larvae of the six phenotypes from six colonies; Table S1) and we used each larva as an independent biological replicate for qRT‐PCR (i.e., six biological replicates per phenotype). To confirm the caste fate of all sampled larvae, we measured their mass and head width and found that they were all within the expected ranges for larvae of each caste pathway and instar (Table S2). We also allowed unsampled larvae from each colony that had developed at the same time as sampled larvae to develop into adults (or into fourth‐instar larvae in the case of queen‐destined larvae). For each sampled larval phenotype, 95%–100% of unsampled larvae developed into an adult of the expected caste (Table S1).

#### 2.1.2 RNA extraction and sequencing

We extracted total RNA following methods detailed in the Supporting information. For mRNA‐seq, we produced 20 pooled‐larvae samples of total RNA, consisting of three EQ samples, three EW samples, four MQ samples, three MW samples, four LQ samples, and three LW samples. The samples were sequenced by the Earlham Institute (Norwich, UK; see Supporting information). For qRT‐PCR, we extracted total RNA from each of the 36 individual larvae, which were then assayed for gene expression as described below.

#### 2.1.3 Bioinformatic analysis

We conducted bioinformatic analysis of the mRNA‐seq data following the approach of Mohorianu et al. (2017a). We used custom‐made Perl scripts (available at https://github.com/sRNAworkbenchuea/UEA_sRNA_Workbench) to quality filter mRNA‐seq reads and aligned them to the *B*. *terrestris* reference genome (Sadd et al., 2015) with PatMaN (Prufer et al., 2008). We next summarised gene expression as the algebraic sum of abundances of reads incident to a transcript, normalised the count matrix using a nonparametric subsampling normalisation, and then determined that the quality of the libraries following these steps was sufficient for differential expression analysis (Figures S4, S5, S6, S7, S8; see Supporting information for details). As part of the quality filtering process, we removed three replicate libraries (MQ3, LQ3, MW1), leaving 17 mRNA‐seq libraries for differential expression analysis (see Supporting information for details).

#### 2.1.4 Differential gene expression between caste phenotypes

In the mRNA‐seq data, we defined each gene/transcript (including unannotated exons) as a significantly differentially expressed gene (DEG) between caste phenotypes when the maximal confidence intervals (difference between the minimum and maximum normalised reads counts across biological replicates) for queen‐ and worker‐destined larvae in a given developmental stage did not overlap (Mohorianu et al., 2013). Following Mohorianu et al. (2013, 2017a), we calculated offset fold change (OFC) for upregulation in k (i.e., expression higher in sample k than k’) as follows (with min and max representing the minimum and maximum normalised read counts for the denoted samples, respectively, and 20 representing a base‐level offset to prevent the rank of OFCs being masked by low‐abundance reads):

$${\text{OFC = log}}_{2}\frac{\min_{k}+20}{\max_{k^{\prime }}+20}.$$

To isolate the DEGs with the greatest potential effects on caste determination and/or differentiation and highest likelihood of qRT‐PCR validation, we defined DEGs as highly significantly differentially expressed genes (HDEGs) when OFC >1 between caste phenotypes within a larval stage and after accounting for multiple unannotated exons making up a longer multiple‐exon transcript (Collins et al., 2017; Mohorianu et al., 2013, 2017a; See Supporting information for details). Unless otherwise stated, we define a DEG or HDEG as upregulated if it had higher expression in one caste phenotype compared to the other within a larval stage; hence, for example, a gene defined as upregulated in EQ would be upregulated in EQ relative to EW (alternatively, downregulated in EW relative to EQ) and a gene defined as upregulated in EW would be upregulated in EW relative to EQ (alternatively, downregulated in EQ relative to EW). Using these methods and definitions, we produced six lists each of DEGs and HDEGs upregulated in EQ, EW, MQ, MW, LQ and LW. To address Hypothesis 1 (differential gene expression across the caste pathways increases during development), we compared gene expression between caste pathways for each larval stage, defining the degree of differential gene expression as: (a) Number of differentially expressed genes; and (b) the magnitude of OFC. We refer to the above method of isolating DEGs and HDEGs from raw sequence data as the “PatMan/confidence‐interval pipeline”, and found that it compared favourably with two other pipelines (Kallisto and HISAT2/HTSeq) used by previous studies to isolate differentially expressed genes in mRNA‐seq analyses of *B*. *terrestris* (Bebane et al., 2019; Colgan, Carolan, et al., 2019; Colgan, Fletcher, et al., 2019). We used these pipelines to analyse our mRNA‐seq data and found the PatMan/confidence‐interval pipeline predicted a high proportion of the DEGs that Kallisto and HISAT2/HTSeq predicted (means of 80.8% and 84.9%, respectively), along with DEGs not predicted by them (Figures S9, S10; Table S3). Furthermore, previous studies of *D*. *melanogaster* found a high degree of congruence (78%) between mRNA‐seq data analysed using the PatMan/confidence‐interval pipeline and qRT‐PCR data (Mohorianu et al., 2017b). Use of the PatMan/confidence‐interval pipeline over Kallisto and HISAT2/HTSeq did not affect the main conclusions of the analysis (see Supporting information for details).

#### 2.1.5 Unannotated and novel genes

To test Hypothesis 2 (novel genes hypothesis), we classified transcripts that mapped to the genome and were found to be HDEGs into four annotation classes: (a) Annotated genes (transcripts that map to *B*. *terrestris* gene annotations); (b) unannotated but not‐novel genes (transcripts that map to unannotated/uncharacterised regions, but that have significant homology to sequences in other insect genera); (c) unannotated, novel non‐coding RNA (non‐coding transcripts that map to unannotated/uncharacterised regions, and that do not have homology to other insect genera); and (d) unannotated, novel coding RNA (coding‐transcripts that map to unannotated/uncharacterised regions, and that do not have significant homology to other insect genera). We used BLASTX/BLASTN to compare each transcript with the NCBI nucleotide and protein databases, defining each transcript as novel if either search returned a hit to the genus *Bombus* but no other genus at E value ≤ 1 × 10^−5^ (Feldmeyer et al., 2014; Ferreira et al., 2013; Toth & Rehan, 2017). We then performed Fisher's exact test to test for significant differences in the relative proportions of novel genes associated with the queen‐ and worker‐ pathways within each developmental stage.

#### 2.1.6 Gene ontology (GO) enrichment analysis

We used a reciprocal best‐hit protein (RBH) BLAST search (Bork et al., 1998; Tatusov et al., 1997) between *B*. *terrestris* and *D*. *melanogaster* (Hoskins et al., 2015) to generate lists of *D*. *melanogaster* orthologues for the *B*. *terrestris* DEGs in each phenotype (Table S4). We then used GOrilla (Eden et al., 2009) and Revigo (Supek et al., 2011) software to conduct GO term enrichment analyses on each of the six *B*. *terrestris* lists of DEG orthologues (target sets), against a background set of *D*. *melanogaster* orthologues with *B*. *terrestris* (Table S4). We did not conduct GO term enrichment analyses on the HDEG lists as the numbers of *D*. *melanogaster* orthologues in each phenotype were too small (1–19 orthologues depending on phenotype; Table 1) to be informative (see Supporting information for details).

**TABLE 1:** Numbers of differentially expressed genes (DEGs) from mRNA‐seq libraries prepared from pooled whole‐body samples of female larvae of *Bombus terrestris* in six larval phenotypes

	EQ	EW	MQ	MW	LQ	LW
DEGs	817	1,337	3,720	2,572	1,976	1,432
Significant *Drosophila* orthologues	174	554	1,456	432	644	496
GO terms for significant orthologues	45	72	89	6	24	83
DEOs	287	900	2,397	829	1,174	774
HDEGs	0	0	54	92	40	14
Highly significant *Drosophila* orthologues	N/A	N/A	19	11	6	1
HDEOs	N/A	N/A	21	18	13	2
Highest OFC	0.89	0.89	5.8	3.94	3.69	3.14

Columns indicate: EQ, early‐instar queen‐destined larvae (*n* = 3); EW, early‐instar worker‐destined larvae (*n* = 3); MQ, mid‐instar queen‐destined larvae (*n* = 3); MW, mid‐instar worker‐destined larvae (*n* = 2); LQ, late‐instar queen‐destined larvae (*n* = 3); LW, late‐instar worker‐destined larvae (*n* = 3). Rows indicate: DEGs, the number of differentially expressed genes (upregulated in a given caste‐pathway (queen or worker) relative to the opposite caste pathway (worker or queen) within each developmental stage [early‐, mid‐, or late‐instar]); Significant *Drosophila* orthologues, the number of DEGs that had a reciprocal best‐hit (RBH) *BLAST* hit to a gene in *D*. *melanogaster* in each phenotype; GO terms for significant orthologues, the number of significant gene ontology terms for the significant *Drosophila* orthologues in each phenotype; DEOs, the number of DEGs that had a RBH *BLAST* hit to a gene in *Apis mellifera*; HDEGs, the number of highly differentially expressed genes (see text for definition) in each phenotype; Highly significant *Drosophila* orthologues, the number of HDEGs that had a RBH *BLAST* hit to a gene in *D*. *melanogaster* in each phenotype; HDEOs, the number of HDEGs that had a RBH *BLAST* hit to a gene in *A*. *mellifera*; Highest OFC, the OFC value of the most highly upregulated gene in each phenotype. N/A, not applicable.

#### 2.1.7 Selection and validation of genes of interest for qRT‐PCR

Using the mRNA‐seq data, we selected genes appearing most likely to influence caste determination and/or differentiation as targets for validation by qRT‐PCR. Specifically, we listed genes that met any of the following overlapping criteria: (a) Showed the highest degree of differential expression in each of the phenotypes; (b) was classed as a HDEG (i.e., OFC >1 between caste phenotypes) within at least one of the three developmental phenotypes; (c) was classed as a HDEG within at least two of the three developmental phenotypes; (d) fell within a gene family with at least one other member that was also highly significantly differentially expressed between caste phenotypes within at least one of the three larval stages; and (e) had been linked by published studies to caste determination and/or differentiation in *B*. *terrestris* or other eusocial Hymenoptera (Table 2, Table S5). In total, in the entire mRNA‐seq data set, there were 22 genes that matched selection criterion(a) and/or at least two of the other selection criteria, and we defined all 22 such genes as “genes of interest”. We then selected the 14 genes of interest with the highest expression levels in at least one phenotype as “target genes” for qRT‐PCR (Table S6).

**TABLE 2:** Genes of interest isolated from mRNAseq of pooled female larvae of *Bombus terrestris* and validated using qRT‐PCR in the current study (Figure 2) and possible functions as reported for these or related genes by other studies

Gene	Taxon or taxa	Possible function(s) from previous studies	Reference(s)
**Hexamerin**	*B. terrestris*	This gene and relatives are upregulated in larvae and pupae compared to adults. Upregulated in adult queens compared to workers.	Colgan et al. (2011)
**Hexamerin**	*A. mellifera*	Close association with gonad development/maturation in queens.	Martins et al. (2011)
Hexamerin relative	*Reticulitermes flavipes*	Associated with JH during worker caste differentiation.	Zhou et al. (2007)
Hexamerin family	*A. mellifera*	Induced expression in response to topical application of JH and roles in caste differentiation.	Martins et al. (2010)
Hexamerin family	*Solenopsis invicta*	Upregulated in queens/nurses compared to foragers. Topical application of a JH analogue induced repression.	Hawkings et al. (2019)
**Takeout‐like**	*A. mellifera*	Antagonistically relationship with JH in adults.	Hagai et al. (2007)
**Takeout‐like**	*B. terrestris*	Sex differentiated in larvae.	Harrison et al. (2015)
**Kr‐h1**	*B. terrestris*	Associated with JH in regulation of reproduction. Downregulated in workers in response to the queen's presence.	Shpigler et al. (2010)
**Kr‐h1**	*A. mellifera*	Association with brain‐specific changes that may induce foraging behaviour.	Fussnecker and Grozinger (2008); Grozinger et al. (2003)
Kruppel family	*Insects*	Functional link to JH. Plays a key role in repression of metamorphosis in insects.	Kayukawa et al. (2012); Lozano and Belles (2011)
Chymotrypsin−2 relative	*A. mellifera*	Possible role for processing royal jelly in queen‐ destined larvae.	Matsuoka et al. (2014)
Chymotrypsin−2 relative	*A. mellifera*	Upregulated in worker‐destined compared to queen‐destined larvae.	Vojvodic et al. (2015)
Yellow family	*A. mellifera*	Roles in development and behaviour. Caste‐specific expression. Related to major royal jelly family.	Drapeau et al. (2006)
**Yellow**	*D. melanogaster*	Associated with development and melanisation.	Han et al. (2002)
**CYP6 k1**	*Blattella germanica*	Expressed throughout development.	Wen and Scott (2001)
**CYP303a1**	*D. melanogaster; Locusta migratoria*	Essential for proper development and metamorphosis.	Wu et al. (2019)
Nrf proteins	*B. terrestris*	Association with sex differentiation in larvae.	Harrison et al. (2015)
**Dnmt3**	*A. mellifera*	Silencing in larvae induces development as queens.	Kucharski et al. (2008)
**Vitellogenin‐like**	*B. terrestris*	Contains mirtron sequences that are caste‐differentiated in larvae.	Collins et al. (2017)
**Vitellogenin‐like**	*A. mellifera*	Larval‐specific expression with potential role in metamorphosis.	Shipman et al. (1987)
Vitellogenin family	*A. mellifera*	Roles in caste‐specific expression and queen longevity. Functional link to JH.	Corona et al. (2007); Amdam et al. (2006)

Gene names highlighted in bold represent the same genes (or homologues) as those reported in this study; the remaining rows represent genes that are related to, or represent, the wider gene family of genes reported in the current study. The names given are the *B*. *terrestris* gene names from NCBI, which may differ from names in other species.

To address Hypothesis 3 (*Dnmt3* and *vitellogenin‐like* upregulation in queen‐destined larvae), we inspected the DEG lists for evidence of upregulation. We found that these two genes were not among the DEGs in the mRNA‐seq data. However, as these could have been false negatives, we added these two genes to the 14 genes already selected for qRT‐PCR. Hence, in total, we assayed gene expression in 16 target genes using qRT‐PCR (Table S6).

#### 2.1.8 Primer design and qRT‐PCR

Using methods detailed in the Supporting information we produced primers for 16 target genes and eight candidate reference genes (Table S6). We synthesised cDNA from each of the 36 RNA samples. We then ran qRT‐PCR reactions using the primers with the highest specificity and efficiency for each gene plus cDNA, alongside a no‐RT control, a no‐template control, and an interplate calibrator (IPC) (Hellemans et al., 2007), to generate Cq values for differential expression analysis of six biological replicates for each of the six phenotypes. We analysed the normalised Cq values of the eight candidate reference genes and, using geNorm (Vandesompele et al., 2002) and BestKeeper (Pfaffl, 2004), determined that at least two reference genes were required. We then selected the three most stable candidate reference genes, which were Ribosomal protein S5a (XM_012308701), Ribosomal protein 18S (XM_003400778), and TATA‐binding protein (XM_003402044; Figure S11). We used the Cq values for these genes and each target gene to calculate relative quantification for each target gene (see Supporting information for details), testing for significant differences in gene expression levels between queen and worker‐destined larvae within each larval stage with Mann–Whitney *U* tests.

For each of the 16 target genes, we compared the qRT‐PCR expression results against the mRNA‐seq data in three comparisons (one within each developmental stage) between queen‐ and worker‐destined larvae (16 × 3 = 48 comparisons in total). We then assessed whether for each comparison the relatively quantified Cq values obtained from qRT‐PCR showed the same results as the mRNA‐seq data (see Supporting information for details).

### 2.2 Comparative analysis of caste‐associated genes in larvae

#### 2.2.1 Direct gene list comparative analysis

To address our second aim (compare caste‐associated genes between *B*. *terrestris* and *A*. *mellifera*), we used a RBH BLAST analysis (Holman et al., 2019; Jackson et al., 2019; Jones et al., 2017) to isolate the orthologues between the two species (Table S7; see Supporting information for details). Using this orthologue list, we produced two gene lists within each of the six *B*. *terrestris* phenotypes, i.e lists of *B*. *terrestris* DEGs and HDEGs that had orthologues in *A*. *mellifera* (DEOs and HDEOs, respectively). We then compared these *B*. *terrestris* orthologue lists with gene lists from two studies of *A*. *mellifera*: (a) Cameron et al. (2013), which used microarrays to isolate gene expression differences between queen‐destined and worker‐destined larvae at the following timepoints: 6 h (first‐instar), 12 h (second‐instar), 36 h (second‐instar), 60 h (third‐instar), 84 h (fourth‐instar), 108 h (fifth‐instar) and 132 h (fifth‐instar); and mRNAseq at the 60 h timepoint; and (b) He et al. (2017), which used mRNA‐seq to isolated differentially expressed genes between queen‐destined and worker‐destined larvae at ages 2 days (third‐instar) and 4 days (fourth/fifth‐instar). From each study, we downloaded the total gene list and the list of genes that were differentially expressed (as defined by each study) between caste pathways at each timepoint. We then used the original list of orthologues between *B*. *terrestris* and *A*. *mellifera* to generate separate lists of *B*. *terrestris* orthologues differentially expressed in each of the *A*. *mellifera* comparison studies.

The caste determination mechanisms and number of instars differ between *B*. *terrestris* and *A*. *mellifera* (Haydak, 1970; Slater et al., 2020; Wheeler et al., 2014; Winston, 1987), so we constructed multiple contingency tables to compare gene lists within the closest phenotypes between species in each study (Figure S12; Table S8). Overall, we made 36 comparisons between our lists of orthologues and those derived from Cameron et al. (2013; from their microarray data only), and 18 comparisons between our lists of orthologues and those derived from He et al. (2017; from their mRNA‐seq data; Table S8). We then performed a Fisher's exact test on each pair of compared lists of orthologues to detect significant overlaps between each pair, accounting for multiple testing using Bonferroni correction.

#### 2.2.2 HISAT2/HTSeq comparative analysis

We also tested whether any gene list differences between the two species stemmed from either: (a) Technical differences in the analysis pipelines used across different studies; or (b) biological differences occurring from queen diapause in *B*. *terrestris* but not *A*. *mellifera*. We used a shared HISAT2/HTSeq pipeline to: (a) Align, and conduct differential expression analysis on, the three raw mRNA‐seq data sets from the current study, Cameron et al. (2013) and He et al. (2017), using the same comparative approach as for the Direct gene list comparative analysis to test for overlaps; (b) isolate genes associated with *B*. *terrestris* queen diapause from Amsalem, Galbraith, et al. (2015) and test whether they were over‐represented among the caste‐associated DEGs for each *B*. *terrestris* larval phenotype; and (c) conduct the HISAT2/HTSeq comparative analysis in (a) but with the diapause‐associated genes excluded (see Supporting information for details).

## 3 RESULTS

### 3.1 Caste‐associated genes in *Bombus terrestris* larvae

#### 3.1.1 mRNA‐seq: Overall results

We sequenced 742,242,464 reads (after quality filtering) across all 17 retained libraries (Table S9; Collins et al., 2016). A total of 603,460,884 reads (81.3%) mapped to the genome. Overall, our analysis revealed 14,817 expressed genes present in at least one of the 17 mRNA‐seq libraries representing the six larval phenotypes (Table S10). A multidimensional scaling plot analysis showed that after removal of three of the libraries, the biological replicates clustered closely by larval developmental stage though not by caste (Figure S4), suggesting that relatively few differentially expressed genes of the total set are associated with caste within each developmental stage.

#### 3.1.2 Differential gene expression between caste phenotypes

**FIGURE 1:**
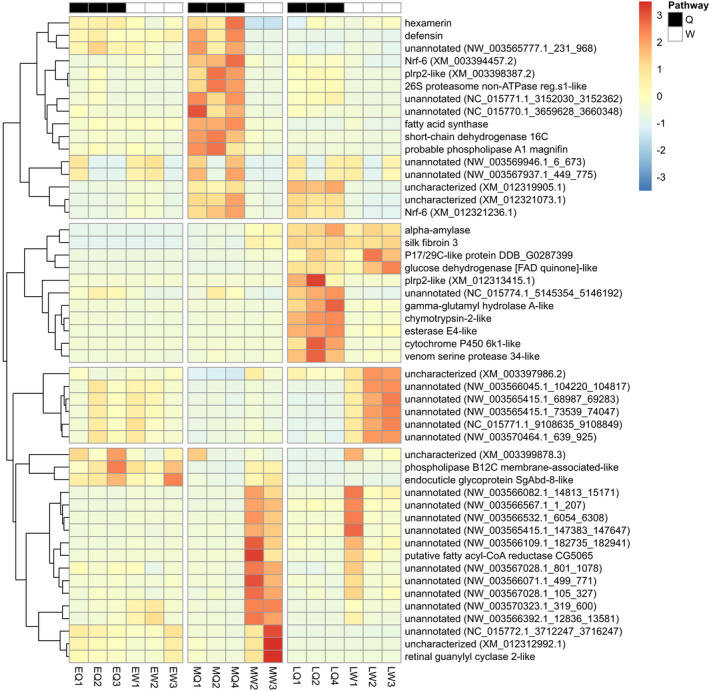
Gene expression differences from mRNA‐seq libraries prepared from pooled whole‐body samples of female larvae of *Bombus terrestris* in six larval phenotypes. Differences expressed in a heatmap showing relative changes in gene expression (OFC) within each gene for the 50 most highly differentially expressed genes (out of 200 HDEGs in total), with each row representing an individual gene and each column representing a biological replicate from the mRNA‐seq data. Vertical breaks represent the three developmental stages (early‐, mid‐, or late‐instar larvae), and the top row represents queen‐ (black) and worker (white)‐destined larvae. The dendogram shows genes that cluster together according to their gene expression patterns, and the four most dissimilar clusters are represented by horizontal breaks. For each annotated gene the NCBI Refseq description is provided, and for each unannotated/uncharacterised gene the sequence ID is provided. See Table 1 legend for phenotype abbreviations and sample sizes

A total of 11,854 genes/transcripts were classified as DEGs and 200 as HDEGs (Table S11). Thirteen HDEGs were highly differentially expressed between castes at multiple timepoints (Table S12). For DEGs within each larval stage, the data partially supported Hypothesis 1 (differential gene expression between caste phenotypes increases during development), as the lowest numbers of DEGs were in early‐instar larvae (817 upregulated in EQ, 1,337 upregulated in EW). However, the highest numbers were in mid‐instar larvae (3,720 genes upregulated in MQ, 2,572 upregulated in MW). Similarly, for HDEGs, the lowest numbers were in early‐instar larvae (0 HDEGs in each caste pathway) and the highest numbers were in mid‐instar larvae (54 upregulated in MQ, 92 upregulated in MW; Table 1; Figure 1). Furthermore, mid‐instar larvae showed the highest expression differences in terms of having the highest OFC values, with pancreatic lipase‐related protein 2‐like (*Plrp2‐like*; XM_003398387.3) in MQ being the most differentiated gene in mid‐instar larvae with an OFC of 5.8, followed by *P17/29C‐like protein DDB_G0287399* (*P17/29C‐like*, XP_003398842.1) in MW with an OFC of 3.94 (Tables 1, S10). By contrast, the most highly differentiated genes in the early‐instar larvae were an unannotated transcript (NC_015770.1_12249827_12250301) in EQ with an OFC of 0.89, and an uncharacterised gene (XM_012314904.2) in EW with an OFC of 0.89 (Tables 1, S10). These results confirm the prediction of Hypothesis 1 that gene expression differences would be lowest in early‐instar larvae but not the prediction that the greatest differences would be in late‐instar larvae.

#### 3.1.3 Unannotated and novel genes

**FIGURE 2:**
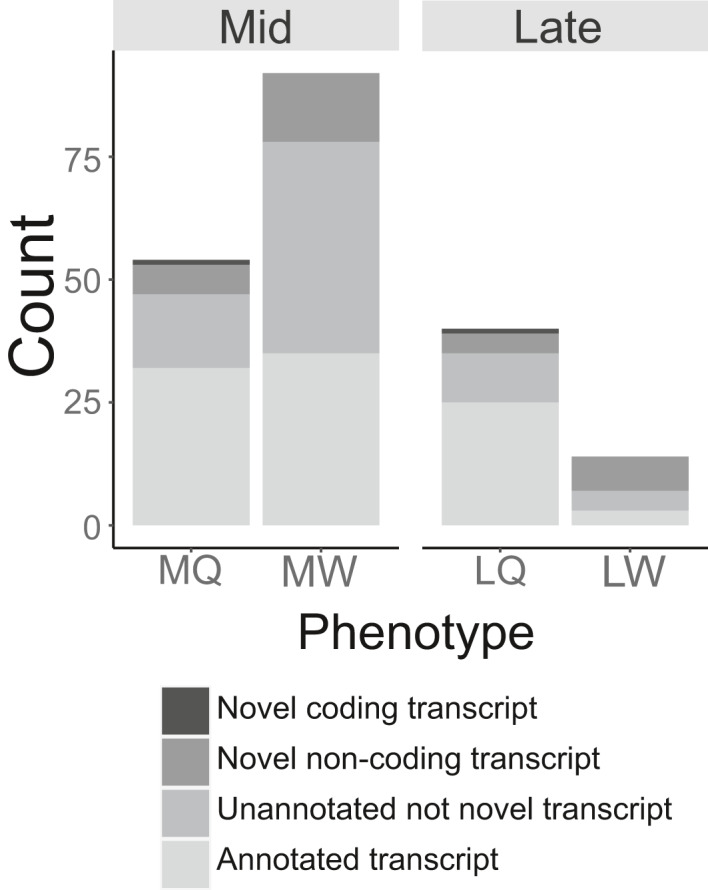
Relative abundance of highly differentially expressed transcripts (HDEGs) of different classes from mRNA‐seq analysis of pooled whole‐body samples of female larvae of *Bombus terrestris*. Data for EQ and EW are not shown as these phenotypes did not return any HDEGs. See Table 1 legend for phenotype abbreviations and sample sizes. The numbers for each gene category are shown in Table S11

Of the 200 HDEGs in queen‐ vs. worker‐destined larvae of all stages, 95/200 (47.5%) were annotated transcripts, 79/200 (39.5%) were unannotated transcripts and 26/200 (13%) were uncharacterised transcripts (Figure 2; Table S11). Of the 79 + 26 = 105 unannotated/uncharacterised transcripts, 33 (31.4% of the unannotated/uncharacterised transcripts, 16.5% of the HDEGs) were found to be novel (i.e., contain no orthologues outside *Bombus*), lending partial support to Hypothesis 2 (novel genes hypothesis). Worker‐destined larvae had proportionally more novel HDEGs (with 14/92 (15.2%) and 7/14 (50.0%) of the total upregulated transcripts being novel in MW and LW, respectively; Figure 2) than queen‐destined larvae (with 7/54 (13.0%) and 5/40 (12.5%) of the total upregulated transcripts being novel in MQ and LQ, respectively; Figure 2). This difference was significant in late‐ but not mid‐instar larvae (Fisher's exact test; *p* = 0.042 and *p* = 0.813, respectively). Of the 33 novel transcripts, one (NW_003568470.1_7_877; upregulated in MQ and LQ) was predicted to be protein‐coding and all remaining ones were predicted to be long non‐coding RNAs (lncRNAs) (Table S11).

#### 3.1.4 Gene ontology (GO) enrichment analysis

**FIGURE 3:**
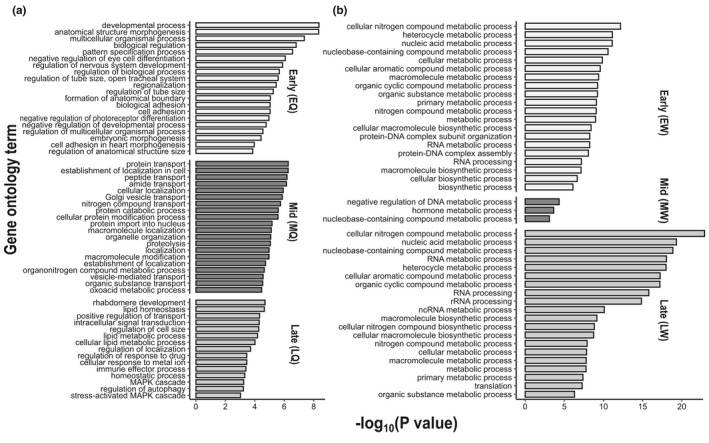
Enriched gene ontology (GO) terms for differentially expressed genes (DEGs) between caste pathways isolated from pooled whole‐body samples of female larvae of *Bombus terrestris* by mRNA‐seq. Due to the high number of GO terms (319 nonredundant terms across all six phenotypes), the 20 most significant biological‐processes GO terms in each phenotype are shown (or the most significant terms for phenotypes with fewer than 20 biological‐processes GO terms). In each figure, early‐, mid‐, and late‐instar larvae are represented by light, dark, and mid‐grey boxes, respectively. (a) GO terms associated with genes upregulated in queen‐destined larvae relative to worker‐destined larvae; (b) GO terms associated with genes upregulated in worker‐destined larvae relative to queen‐destined larvae. See Table 1 legend for phenotype abbreviations and sample sizes and Table S13 for the full list of GO terms

The RBH *BLAST* search between the transcriptome of *B*. *terrestris* and the annotated gene set of *D*. *melanogaster* revealed 7,689 orthologous genes (51.9% of 14,817 genes in the *B*. *terrestris* mRNA‐seq libraries; Table 1). These were used to isolate 319 nonredundant GO terms for the DEGs (Figure 3; Table S13). In the queen‐destined pathway, the enriched GO terms were closely associated with morphogenesis, developmental processes, and transport processes. For example, in EQ, among the most significant GO terms were the terms “developmental processes”, “anatomical structure morphogenesis”, and “pattern specification process”; in MQ the most significant term was protein transport (and 11/52 GO terms referred to “transport”); and, in LQ, among the most significant GO terms were “rhabdomere development” and “positive regulation of transport” (Figure 3). In addition, MQ contained the significantly enriched GO term “reproductive processes”. In the worker‐destined pathway, the most significant enriched GO terms were closely associated with metabolic processes, with 17/42, 3/3, and 17/46 terms referring to “metabolic processes” in EW, MW, and LW, respectively (Figure 3; Table S13).

#### 3.1.5 Validation of genes of interest by qRT‐PCR

**FIGURE 4:**
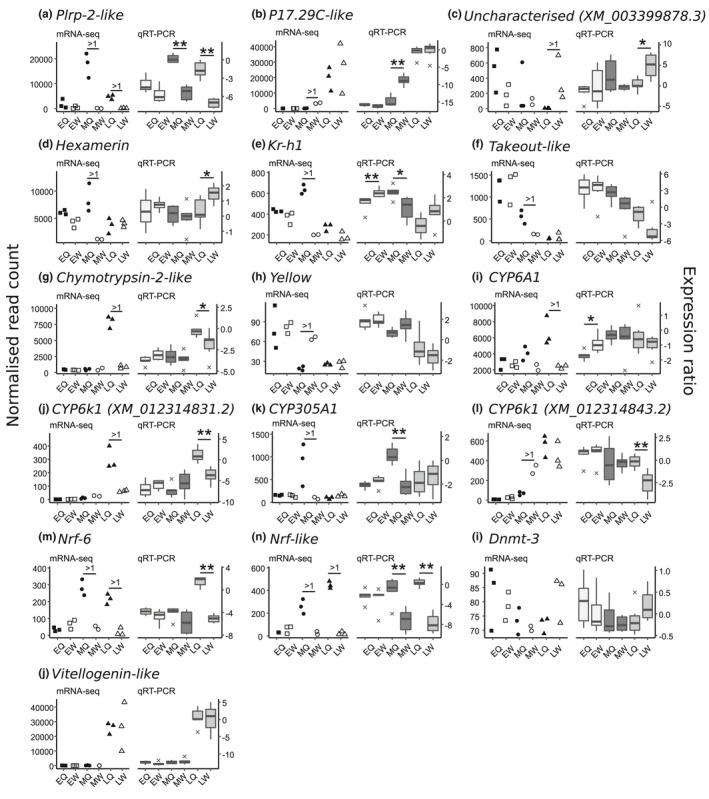
Gene expression levels from mRNA‐seq and qRT‐PCR assays of selected target genes in pooled whole‐body samples of female larvae of *Bombus terrestris*. Univariate scatterplots (left‐hand graphs in each panel) represent normalised read counts of target genes from mRNA‐seq. Early‐, mid‐, and late‐instar larvae are represented by circles, squares, and triangles, respectively, and queen‐ and worker‐destined larvae are represented by black and white shapes, respectively. Horizontal bars annotated with “>1” represent queen‐worker pathway comparisons that showed a log. offset fold change >1 (i.e., were highly differentially expressed genes (HDEGs) in the mRNA‐seq data). Boxplots (right‐hand graphs in each panel) represent relative gene expression levels (expression ratios) of target genes from qRT‐PCR, where early‐, mid‐, and late‐instar larvae are represented by light, dark, and mid‐grey boxes, respectively. Horizontal bars annotated with asterisks represent queen‐worker pathway comparisons that were significantly differentially expressed in the qRT‐PCR data (Mann‐Whitney U test; * *p* < 0.05; ** *p* < 0.01). In the boxplots, black horizontal bars represent the median gene expression values, boxes represent the interquartile ranges, error bars represent ranges up to 1.5 × the interquartile range, and black crosses represent points that fell beyond the 1.5 × interquartile range. See Table S14 for full gene names and accession numbers. See Table 1 legend for phenotype abbreviations. For the mRNA‐seq data: EQ, *n* = 3; EW, *n* = 3; MQ, *n* = 3; MW, *n* = 2; LQ, *n* = 3; LW, *n* = 3; for the qRT‐PCR data: EQ, *n* = 6; EW, *n* = 6; MQ, *n* = 6; MW, *n* = 6; LQ, *n* = 6; LW, *n* = 6

The selected genes of interest that were HDEGs fell into five gene categories (described in Supporting information). From the mRNA‐seq results (Figure 4), we made 48 phenotypic comparisons of the 16 genes of interest (representing a comparison between queen‐destined and worker‐destined larvae in three developmental stages for each gene). In the mRNA‐seq data, the genes were highly significantly differentially expressed across phenotypes in 17 cases and not highly significantly differentially expressed across phenotypes in 31 cases. Of the 17 cases in which the mRNA‐seq data showed significant differential expression between phenotypes, 11 also showed significant differential expression in the same direction in the qRT‐PCR data (Figure 4; Table S14). Of the 31 cases in which the mRNA‐seq data did not show highly significant differential expression, 27 also did not show significant differential expression in the qRT‐PCR data (Figure 4; Table S14). Overall, these findings demonstrated a high degree of congruence between the mRNA‐seq results (as predicted by the PatMan/confidence‐interval pipeline) and qRT‐PCR results for the 16 target genes, as 38 (i.e., 11 + 27) of 48 (79.2%) of phenotypic comparisons showed the same results in the qRT‐PCR data as in the mRNA‐seq data (binomial test of the proportion of successful matches relative to mismatches, *p* = 3.1 × 10^−5^). For each of the remaining 6/17 HDEG comparisons that were not significantly differentially expressed in the same direction in the qRT‐PCR data, the mean expression value in these data was higher in the same phenotype as in the mRNA‐seq data. Therefore, the trend in differential expression for these six genes was the same for both methods (Table S14). In addition to this, 11/16 genes of interest showed high Pearson correlation coefficients (> 0.7) between the mRNA‐seq and qRT‐PCR data (Figure S13).

We found no support for Hypothesis 3 (*Dnmt3* and *vitellogenin‐like* upregulation in queen‐destined larvae), as neither gene was differentially expressed between caste pathways in either the mRNA‐seq or the qRT‐PCR data (Figures 4O–P).

### 3.2 Comparative analysis of caste‐associated genes in larvae

#### 3.2.1 Direct gene list comparative analysis

**FIGURE 5:**
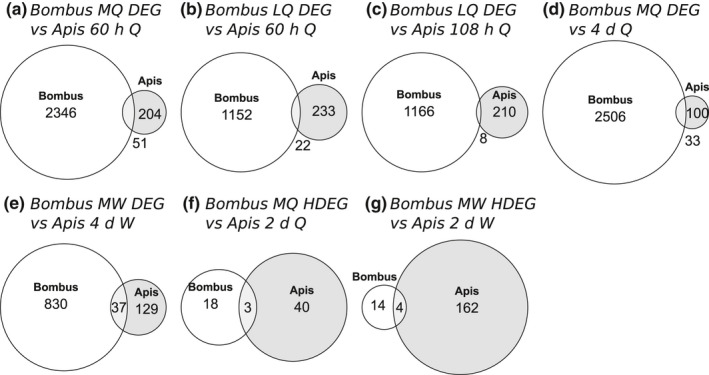
Results of direct gene list comparative analysis: Euler diagrams of significant overlaps between caste‐associated genes isolated from female larvae of *Bombus terrestris* by mRNA‐seq in the current study and caste‐associated genes isolated from female larvae of *Apis mellifera*. *A*. *mellifera* data came from Cameron et al. (2013) (a, b; 3/36 comparisons were significant), and He et al. (2017) (c–f; 4/18 comparisons were significant). Overlap between differentially expressed genes (DEGs) upregulated in: (a) MQ in *B*. *terrestris* with genes upregulated in 60 h‐old queen‐destined larvae in *A*. *mellifera*; (b) LQ in *B*. *terrestris* with genes upregulated in 60 h‐old queen‐destined larvae in *A*. *mellifera*; (c) LQ in *B*. *terrestris* with genes upregulated in 108 h‐old queen‐destined larvae in *A*. *mellifera*; (d) MQ in *B*. *terrestris* and genes upregulated in 4‐day‐old queen‐destined larvae in *A*. *mellifera*; (e) MW in *B*. *terrestris* and genes upregulated in 4‐day‐old worker‐destined larvae in *A*. *mellifera*. Overlap between highly differentially expressed genes (HDEGs) upregulated in: (f) MQ in *B*. *terrestris* and genes upregulated in 2‐day‐old queen‐destined larvae in *A*. *mellifera*; (g) MW in *B*. *terrestris* and genes upregulated in 2‐day‐old worker‐destined larvae in *A*. *mellifera*. Numbers show numbers of orthologues in each category (numbers shown outside the area of overlap where there is no room for them). Nonsignificant overlaps are not shown. See Table 1 legend for phenotype abbreviations

To address our second aim (compare caste‐associated genes between *B*. *terrestris* and *A*. *mellifera*), we isolated 7,598 orthologues that were present in the mRNA‐seq data (including genes that were not differentially expressed) from *B*. *terrestris* (current study) and in the microarray data from *A*. *mellifera* in Cameron et al. (2013) and then directly compared lists of differentially expressed orthologues from the two studies. Overall, 3/22 DEO comparisons and 0/14 HDEO comparisons showed significant overlaps between the two studies (Table S8). The significant overlaps were: (a) 51/2,397 (2.13%) of the DEOs that were upregulated in MQ in *B*. *terrestris* were also upregulated in 36 h old queen‐destined larvae (relative to worker‐destined larvae) in *A*. *mellifera* (Fisher's exact test, *p* = 3.6 × 10^−5^; Figure 5a); (b) 22/1,174 (1.87%) of the DEOs that were upregulated in LQ in *B*. *terrestris* were also upregulated in 60 h old queen‐destined larvae (relative to worker‐destined larvae) in *A*. *mellifera* (Fisher's exact test, *p* = 1.4 × 10^−3^; Figure 5b); and (c) 8/1,174 (0.68%) of the DEOs that were upregulated in LQ in *B*. *terrestris* were also upregulated in 108 h old queen‐destined larvae in *A*. *mellifera* (*p* = 3.2 × 10^−8^; Figure 5c).

We also isolated 6,677 orthologues that were present in the mRNA‐seq data from *B*. *terrestris* (current study) and mRNA‐seq data from the second focal *A*. *mellifera* study (He et al., 2017) in order to directly compare gene lists from the two studies. Overall, 2/10 DEO comparisons and 2/8 HDEO comparisons showed significant overlaps between the two studies (Table S8). The significant overlaps were: (a) 33/2,539 (1.3%) of the DEOs that were upregulated in MQ in *B*. *terrestris* were also upregulated in 4‐day‐old queen‐destined larvae in *A*. *mellifera* (Fisher's exact test, *p* = 1.5 × 10^−3^; Figure 5d); (b) 37/867 (4.27%) of the DEOs that were upregulated in MW in *B*. *terrestris* were also upregulated in 4‐day‐old worker‐destined larvae in *A*. *mellifera* (Fisher's exact test, *p* = 9.18 × 10^−4^; Figure 5e); (c) 3/21 (14.29%) of the HDEOs that were upregulated in MQ in *B*. *terrestris* were also upregulated in 2‐day‐old queen‐destined larvae in *A*. *mellifera* (Fisher's exact test, *p* = 3.1 × 10^−4^; Figure 5f); and (d) 4/18 (22.22%) of the HDEOs that were upregulated in MW in *B*. *terrestris* were also upregulated in 4‐day‐old worker‐destined larvae in *A*. *mellifera* (Fisher's exact test, *p* = 8.59 × 10^−4^; Figure 5g). Across each comparison study, the majority of significantly overlapping genes were with DEOs or HDEOs from mid‐instar larvae in *B*. *terrestris* (5/7 significant comparisons; Figure 5).

From these overlapping lists, we found two HDEOs that were upregulated in queen‐destined larvae in the current study and in both *A*. *mellifera* comparison studies (*probable CYP303a1*, XM_003396659.3 and *alpha‐glucosidase‐like*, XM_012309279.2) and a total of 14 HDEOs that showed significant upregulation in the same direction in the current study and at least one of the *A*. *mellifera* comparison studies (Table S15). Three of the genes that were upregulated in both species were among the target genes selected for qRT‐PCR. These genes were *Hexamerin* (XM_003401733.3), *Krüppel‐homologue 1* (*Kr*‐*h1*; NM_001280921.1), and *probable CYP303a1* (XM_003396659.3). They all showed upregulation in queen‐destined larvae in the mRNA‐seq and qRT‐PCR data from *B*. *terrestris* in the current study and in the data from at least one *A*. *mellifera* study, which supports a caste‐associated role for these genes in both lineages. However, relatively few HDEOs showed similar expression patterns in both species (a maximum of 14/54), and the significant overlaps between the current study and the *A*. *mellifera* studies involved low numbers of genes (range: 3–51 genes; % range: 0.68%–22.22%, Table S8).

#### 3.2.2 HISAT2/HTSeq comparative analysis

These results were similar when the HISAT2/HTSeq pipeline was used (Figure S14; Table S16; see Supporting information for details). In addition, we found no evidence that diapause‐associated genes were over‐represented among the *B*. *terrestris* DEGs (Table S17), and there was no effect on the HISAT2/HTSeq results comparing *B*. *terrestris* and *A*. *mellifera* when diapause genes were excluded from the analysis (Table S16). Overall, the findings provide only partial support for the toolkit genes hypothesis.

We summarise all key findings from the current study in Table S18.

## 4 DISCUSSION

### 4.1 Caste‐associated genes in *Bombus terrestris* larvae

Our first aim was to profile gene expression differences in queen‐ and worker‐destined larvae of *B*. *terrestris* at developmental stages straddling the two sensitive periods of caste determination (Figure S1). We addressed this using mRNA‐seq to profile gene expression differences between queen‐ and worker‐destined larvae at three stages, i.e., early‐, mid‐ and late‐instar larvae (see Introduction). We then validated these results in independently sampled larvae for a subset of 16 genes using qRT‐PCR and found a high degree of congruence between the mRNA‐seq and qRT‐PCR results for these genes.

Hypothesis 1 predicted that differential gene expression between queen‐ and worker‐destined larvae is absent in early‐instar larvae (as they are totipotent), greater in mid‐instar larvae (as they have embarked on one of the two caste pathways) and greatest in late‐instar larvae (as they show the greatest phenotypic differences; Cnaani et al., 2000; Röseler, 1970). The prediction was partially supported: more DEGs and HDEGs occurred in mid‐ and late‐ than in early‐instar larvae and the maximum OFC values were higher in mid‐ than in early‐instar larvae (Figure 1; Table 1). Correspondingly, these results support physiological and observational studies (Cnaani et al., 2000; Röseler, 1970) showing that the queen‐dependent period of caste determination occurs at the end of the second instar in *B*. *terrestris*. However, contrary to Hypothesis 1, the mRNA‐seq results showed that there were more genes differentially expressed across the caste pathways in mid‐instar larvae than in late‐instar larvae (Table 1). A possible reason for this finding is that the nutrition‐sensitive period affects caste less strongly (see Introduction), and therefore there are fewer caste‐associated genes than during the queen‐dependent period. Evidence that the nutrition‐sensitive period is less influential comes from the findings that worker‐destined larvae cannot change to the queen pathway during the nutrition‐sensitive period (Amsalem, Grozinger, et al., 2015) and that queen‐destined individuals underfed during the nutrition‐sensitive period may be intermediate in size between queens and workers rather than having a full worker phenotype (Pereboom et al., 2003).

Hypothesis 2 predicted that a high number of novel (taxonomically‐restricted) genes would be associated with caste determination and/or differentiation, and that novel genes would be over‐represented in the worker pathway genes (see Introduction). We found that, of the 105 unannotated/uncharacterised HDEGs in *B*. *terrestris*, 33 (31.4%, representing 16.5% of the total HDEGs) were novel. In addition, novel genes were significantly over‐represented in the genes upregulated in LW (although not MW), of which 7/14 (50%) genes were novel compared to 5/40 (12.5%) genes upregulated in LQ being novel. These results partially support Hypothesis 2 as a proportion of caste‐associated genes were novel, and the worker pathway was relatively enriched for novel genes. However, studies of other eusocial insects have found up to 75% novel transcripts among caste‐associated genes and a percentage of novel genes among genes upregulated in workers of up to 81.5% (e.g., Ferreira et al., 2013). This suggests a limited, rather than a major, role for novel genes in larval caste determination and/or differentiation in *Bombus*. In our data, one novel HDEG (represented in both MQ and LQ phenotypes) was predicted to be protein‐coding (feature ID: NW_003568470.1_7_877) and the rest were predicted to be lncRNAs. The large proportion of novel lncRNAs, together with previous results isolating caste‐associated miRNAs in larvae in *B*. *terrestris* (Collins et al., 2017), suggest a role for RNA regulation of caste in *B*. *terrestris* and possibly other eusocial insects.

GO analysis of DEGs showed that queen‐destined larvae tended to be enriched for genes involved in development and morphogenesis (each developmental queen‐destined phenotype had highly significant GO terms related to these terms), while worker‐destined larvae tended to be enriched for genes involved in metabolism (Figure 3; Table S13). This difference could be due to queen‐destined larvae undergoing greater developmental change, given that adult queens are both much larger than adult workers and differ from them in morphology, behaviour, and physiology. The enrichment of the worker pathway in metabolism‐associated genes represents a contrast between *Bombus* and *Apis*, as in *Apis* metabolism genes are generally more closely associated with the queen pathway (Barchuk et al., 2007; Evans & Wheeler, 2001). However, in *B*. *terrestris*, GO analysis may have captured relatively fewer relevant genetic mechanisms in the worker compared to the queen pathway, given the higher proportion of novel genes in the worker pathway (Figure 2).

Several of the genes of interest in this study (e.g., HDEGs including *Hexamerin*, *Kr*‐*h1*, *Takeout‐like*, and relatives of these genes) have previously been shown to be up‐ or downregulated in response to JH in either *B*. *terrestris* or other eusocial insects, and these genes were upregulated in queen‐destined larvae in the mRNA‐seq data from the current study (Figure 4; Table 2). JH regulation is an essential component of caste determination and/or differentiation in *B*. *terrestris* and JH levels peak in queen‐destined larvae following the queen‐dependent period (Bortolotti et al., 2001; Cnaani et al., 2000). Upregulation of *Hexamerin* and *Kr*‐*h1* in MQ (as found in the current study) is consistent with this role, as the mid‐instar stage follows the queen‐dependent period, suggesting that these genes, and their possible interactions with JH, are potentially important candidates for involvement in caste determination and/or differentiation. *Takeout‐like* (XM_003397243.2) also has a negative relationship with JH in brain of adult *A*. *mellifera* workers (Table 2), and so its upregulation in MQ shows that its response to JH may be context‐dependent.

The *CYP* gene family is a large and diverse gene family, and several *CYP* genes have previously been linked to caste determination and/or differentiation in eusocial insects (Cameron et al., 2013; Tarver et al., 2012). We found that *CYP* genes were highly represented (4/54, 4/92, and 5/40 *CYP* genes in MQ, MW, and LQ, respectively) among caste‐associated genes in *B*. *terrestris*, and two of the *CYP* genes in this study (*CYP6 k1*, *CYP303a1*) have been shown to be essential for proper development and metamorphosis in other insects (Table 2), indicating that modulation of these two genes could be associated with the morphological changes in development associated with the castes of *B*. *terrestris*.

Several genes have relatives that have been shown to be linked with caste determination and/or differentiation in other eusocial species, but in the direction opposite to that found in *B*. *terrestris* in the current study. For example, *Chymotrypsin* was upregulated in LQ but its relatives were previously associated with the worker pathway in larval *A*. *mellifera*, and *Yellow* was upregulated in MW but members of its gene family were previously associated with the queen pathway in larval *A*. *mellifera* (Figure 4, Table 2). These results support previous suggestions that caste‐associated gene families shared across different eusocial lineages may alter their caste association as these lineages diverge and that new genes may evolve within each family within divergent lineages (Morandin et al., 2015; Smith et al., 2015, 2014). Furthermore, several HDEGs (e.g., *Nose‐resistant to fluoxetine* (*Nrf*) genes and *Takeout‐like*) that were upregulated in queen‐destined larvae in *B*. *terrestris* have been linked to sex differentiation in other insects (Figure 4; Table 2), consistent with reports linking the processes of caste‐ and sex differentiation in other eusocial species (Kapheim, 2019; Klein et al., 2016).

Hypothesis 3 predicted that two genes, *DNA methyl transferase 3B* and *vitellogenin‐like*, would be upregulated in queen‐destined larvae in *B*. *terrestris*; however, there was no evidence for this in either the mRNA‐seq or the qRT‐PCR data (Figure 4). For *Dnmt3*, this result contrasts with findings in *A*. *mellifera*, in which the experimental silencing of *Dnmt3* in larvae generates development as queens (Kucharski et al., 2008). Hence, *Dnmt3* does not appear to play the same role in caste determination and/or differentiation in *Bombus* as in *Apis*. *Vitellogenin‐like* was more highly expressed in late‐instar than in mid‐ or early‐instar larvae but was not caste associated. We have previously shown that this gene contains a mirtron (*Bte‐miR‐6001*) upregulated in late‐instar queen‐destined larvae (Collins et al., 2017). Unlike other intronic miRNAs, mirtrons are usually co‐expressed with their host sequences, and the two may act in concert to regulate the same biological processes (Amourda & Saunders, 2020). However, the results from our previous study imply only *Bte‐miR‐6001* is associated with caste differentiation whereas *vitellogenin‐like* itself is not. One possibility for this apparent anomaly is that additional mechanisms (e.g., alternative splicing) maintain *Bte‐miR‐6001* at a lower level in late worker‐destined larvae than its host gene.

### 4.2 Comparative analysis of caste‐associated genes in larvae

Our second aim was to test whether larval caste‐associated genes were shared between two eusocial lineages, through comparing the *B*. *terrestris* data set from the current study with two published *A*. *mellifera* data sets (Cameron et al., 2013; He et al., 2017). The toolkit genes hypothesis predicts a large overlap in the genes associated with eusociality between independent eusocial lineages. We found significant overlaps in caste‐associated genes between *B*. *terrestris* and *A*. *mellifera*; however, the degree of overlap was low as the majority of comparisons were not significant, and the significant comparisons involved low numbers and proportions of genes (% range: 0.68%–22.22% of the genes in a given *B*. *terrestris* phenotype; Figures 5a–g; Table S8). Five of seven significant overlaps involved the mid‐instar larvae in *B*. *terrestris*, i.e., the developmental stage immediately following the queen‐dependent period. Similar results were obtained when the gene lists were generated using a common bioinformatic pipeline (HSAT2/HTSeq) applied across all three data sets (Figure S14; Table S8).

While *Bombus* and *Apis* differ in their degree of eusociality, comparisons of caste‐associated genes between them are potentially confounded by queen‐worker differences in traits not connected to caste evolution per se (e.g., diapause, which only *B*. *terrestris* queens undergo). However, we found that: (a) Diapause‐associated genes were not significantly over‐represented among the lists of caste‐associated genes in *B*. *terrestris* (Table S17); and (b) removing diapause‐associated genes did not affect the outcome of comparative analyses, as all six overlaps between *B*. *terrestris* and *A*. *mellifera* gene lists from the shared HSAT2/HTSeq analysis remained significant when diapause genes were removed (Table S16). Therefore, although confounds from traits other than diapause cannot be eliminated, our results potentially highlight genes involved in the evolution of different degrees of eusociality in bees.

As discussed above, some of the genes that overlapped between the two species (e.g., *Hexamerin*, *Kr*‐*h1*, and *CYP303a1* (XM_003396659.3); Table S15) are already considered potentially important candidates for involvement in caste determination and/or differentiation. Queen‐destined *A*. *mellifera* larvae maintain higher overall levels of JH than worker‐destined larvae (Wirtze, 1973) and, as in *B*. *terrestris*, topical application of JH to worker‐destined larvae causes them to have queen‐like characteristics as adults (Wirtze & Beetsma, 1972). Therefore, shared caste associations of these genes in both bee species could reflect an ancestral role of JH signalling in caste evolution. Previous studies in eusocial Hymenoptera have found, as in the current comparison of *Bombus* and *Apis*, significant but low overlaps between caste‐associated genes in related eusocial lineages (Berens et al., 2015; Feldmeyer et al., 2014; Ferreira et al., 2013; Morandin et al., 2015; Toth et al., 2010; Woodard et al., 2014). A possible reason for such findings is that gene pathways and gene functions show convergent evolution between lineages even when the actual genes themselves are not conserved, which would represent a modified version of the toolkit concept (Berens et al., 2015). Nonetheless, in the current study, most caste‐associated genes across the two lineages were nonoverlapping, indicating substantial divergence in the caste‐associated genes between *Bombus* and *Apis* since their last common eusocial ancestor (cf. Linksvayer & Johnson, 2019). Together with the results of our novel genes analysis, these results support the conclusion that caste evolution within bees involves both shared toolkit genes and novel genes, in agreement with other recent studies (Kapheim, 2016; Warner et al., 2019). However, our overall understanding of the molecular basis of polyphenisms and the evolution of advanced eusociality would benefit from characterising gene expression differences across sensitive periods of caste determination in a range of additional eusocial taxa spanning different levels of eusociality.

## AUTHOR CONTRIBUTIONS

D.H.C., T.D., and A.F.G.B. designed the research. D.H.C., M.L., M.S., and D.C.P. performed research. D.H.C., A.W., D.C.P., and I.M. analysed data. D.H.C. and A.F.G.B. wrote the manuscript. All authors provided comments on the manuscript.

## Supporting information

Supplementary information including Figure S1‐S6, S9‐S14

Figure S7

Figure S8

Table S1‐S9, S11‐S18

Table S10

## Data Availability

The raw mRNA‐seq read data are available in the National Centre for Biotechnology Information (NCBI) gene expression omnibus (GEO; accession number: GSE90751; Collins et al., 2016). The scripts used in the analyses are available at https://github.com/dhcollins500/Collins‐et‐al_BB‐M001482‐1_obj1 and https://github.com/sRNAworkbenchuea/UEA_sRNA_Workbench.
