# Supplementary material for: Gene expression during larval caste determination and differentiation in intermediately eusocial bumblebees, and a comparative analysis with advanced eusocial honeybees
Source: Mol Ecol. 2021 Jan 7;30(3):718–35. doi: 10.1111/mec.15752 (PMC7898649; doi:10.1111/mec.15752)
Supplement: Supplementary file 1 — Supplementary information including Figure S1‐S6, S9‐S14 [file MEC-30-718-s001.docx]

# Supplemental Information for:

# Gene expression during larval caste differentiation in intermediately eusocial bumblebees, and a comparative analysis with advanced eusocial honeybees

David H. Collins, Anders Wirén, Marjorie Labédan, Michael Smith, David C. Prince, Irina Mohorianu, Tamas Dalmay, and Andrew F. G. Bourke

**Table of Contents:**

| **Supplemental methods** | Page 3 |
| --- | --- |
| **Sample collection** | Page 3 |
| **RNA extraction** | Page 7 |
| **Bioinformatic analysis** | Page 8 |
| **Comparison with alternative pipelines** | Page 12 |
| **Unannotated and novel genes** | Page 15 |
| **Gene Ontology (GO) enrichment analysis** | Page 16 |
| **Selection of target genes** | Page 18 |
| **Primer design and qRT-PCR** | Page 18 |
| **Comparative analysis** | Page 21 |
| **Diapause genes analysis** | Page 26 |
| **Supplemental references** | Page 28 |
| **Supplemental Figure S1** | Page 35 |
| **Supplemental Figure S2** | Page 36 |
| **Supplemental Figure S3** | Page 37 |
| **Supplemental Figure S4** | Page 38 |
| **Supplemental Figure S5** | Page 39 |
| **Supplemental Figure S6** | Page 40 |
| **Supplemental Figure S7** | Page 41 |
| **Supplemental Figure S8** | Page 41 |
| **Supplemental Figure S9** | Page 42 |
| **Supplemental Figure S10** | Page 43 |
| **Supplemental Figure S11** | Page 44 |
| **Supplemental Figure S12** | Page 45 |
| **Supplemental Figure S13** | Page 46 |
| **Supplemental Figure S14** | Page 47 |

# Supplemental Methods

## Caste-associated genes in *Bombus terrestris* larvae

*Sample collection*

We obtained *Bombus terrestris audax* colonies, consisting of a queen, workers and brood, from Biobest Belgium NV (Westerlo, Belgium) and Biobest UK Ltd (Ashford, UK). On receipt, we transferred the colonies into individual wooden nest-boxes with internal dimensions, 17 cm × 27.5 cm × 16 cm high, and counted the number of workers in each colony. We maintained the colonies in constant darkness at 28°C and 60% humidity, and conducted all manipulations under red light. We supplied the colonies ad libitum with sugar solution ('Biogluc', Biobest Belgium NV/Biobest UK Ltd) and freeze-dried pollen (Koppert UK Ltd, Haverhill, UK). We monitored the colonies daily to identify the sex and caste of newly-eclosed bees (i.e. those newly transformed from pupa to adult) and took daily photographs to identify where in the nest new eggs were being laid.

In growing *B. terrestris* colonies (i.e. colonies that have not yet produced sexuals), diploid larvae develop into workers (Cnaani, Borst, Huang, Robinson, & Hefetz, 1997). However, if in such colonies the queen is removed, female larvae younger than the age of the queen-dependent period will be induced to develop into queens (see main text, introduction). Hence, as in Pereboom et al. (2005) and Collins et al. (2017), we used queen removal to experimentally produce queen-destined larvae. In other colonies, we retained colony queens to produce worker-destined larvae. Thereafter, our strategy for sampling female larvae of known caste fate (i.e. known to be on a given caste-specific developmental pathway) was to monitor individual egg clusters in the colonies and periodically sample a proportion of larvae from them while allowing the remaining ones to develop into late-instar larvae or adults, at which point we verified their caste phenotypically (from larval size or adult size and morphology). To ensure that all sampled larvae were female, we only used colonies that had not yet passed their ‘switch point’ i.e. the point at which queens switch from producing diploid, female eggs to producing some haploid, male eggs (Duchateau & Velthuis, 1988). This was achieved by sampling only from colonies younger than two weeks since first worker eclosion, the date of which was inferred by the size of the colonies on receipt.

*B. terrestris* larvae develop through four larval instars (Alford, 1975). In sampling female larvae, we removed approximately 50% of female larvae as first and second instar larvae (henceforth 'early-instar larvae' – representing the larval stage before the queen-dependent period), 10% as third instar larvae (henceforth 'mid-instar larvae' – representing the larval stage after the queen-dependent period but before the nutrition-sensitive period), and 10% as fourth instar larvae (henceforth 'late-instar larvae' – representing the larval stage when larvae have passed both sensitive periods and larval caste fate becomes distinguishable based on size and head width). We allowed the final 30% of female larvae to develop into adult females. In colonies generating samples of queen-destined larvae, we positively identified unsampled larvae as queen-destined once they had reached the fourth instar (as fourth-instar queen-destined larvae are 2-4 times the mass of fourth-instar worker or male larvae (Cnaani et al., 1997; Ribeiro, 1994)). In colonies generating samples of worker-destined larvae, we positively identified unsampled larvae as worker-destined once they had eclosed, since, as adults, workers differ from queens by being smaller and from males by their possession of pollen baskets and a sting. In addition, to check the instar and caste fate of sampled larvae, we measured the head width and mass of each one collected. Using this strategy, we collected known early-instar queen-destined larvae (EQ), early-instar worker-destined larvae (EW), mid-instar queen-destined larvae (MQ), mid-instar worker-destined larvae (MW), late-instar queen-destined larvae (LQ), and late-instar worker-destined larvae (LW) (Figure S3). All samples were generated in two cohorts, with cohort 1 providing RNA for mRNA-seq analysis and cohort 2 providing RNA for quantitative real-time PCR (qRT-PCR) analysis.

*(a) Cohort 1: female larval samples for mRNA-seq*

We obtained 25 *B. t. audax* colonies on 30 October 2014 from BioBest Belgium NV. We did not sample from four colonies that produced more than 50 workers within the first week of observation (and were therefore likely to be close to their switch point), six colonies that had already produced adult males, and one colony in which the queen failed to produce any eggs in the week following arrival. This left 14 colonies from which female larvae could potentially be sampled.

To generate samples of queen-destined larvae as described above, we removed the colony queen from eight of these 14 colonies. We monitored 155 putatively queen-destined larvae in four of these eight colonies (Table S1). The other four colonies from which the colony queen was removed failed to produce any queen-destined larvae. Of the 155 putatively queen-destined larvae, we sampled 125 larvae (as EQ, MQ and LQ) and observed that, of 72 larvae unsampled as MQ larvae, 71 developed into LQ (of which 42 were sampled as such) and one developed into an adult worker (Table S1). As the proportion of larvae found to develop into queens was high (1/72, or 98.6%), we identified the sampled larvae as queen-destined larvae. Overall, we sampled 63 EQ from three colonies, 20 MQ from four colonies, and 42 LQ from four colonies (Table S1). To generate samples of worker-destined larvae, we used the six colonies from which the colony queen was not removed (queenright colonies). We monitored 233 worker-destined larvae in three of these colonies (Table S1). The other three queenright colonies produced a mix of workers and males and were therefore not used for sampling. Of the 233 worker-destined larvae, we sampled 170 (as EW, MW, LW) and observed that, of 63 unsampled late-instar larvae, 63 developed into adult workers. As the proportion of larvae found to develop into workers was 100%, we identified the sampled larvae as worker-destined larvae. Overall, we sampled 104 EW from three colonies, 33 MW from three colonies, and 33 LW from three colonies (Table S1).

As confirmation of the inferred caste pathways of sampled larvae, the measured head widths and masses of the putatively queen-destined and worker-destined larvae in each instar were found to be consistent with those previously observed for these larval phenotypes (Table S2; Cnaani et al, 1997; Collins et al, 2017). Using these measurements, we pooled larvae according to their colony, instar, and inferred caste (Table S1). We then homogenised the larvae using liquid nitrogen and a mortar and pestle to grind them into a fine powder. We mixed the powder with an appropriate volume (1.5 ml for every 100 mg of tissue) of tri-reagent (Sigma-Aldrich, Gillingham, Dorset, UK). The tri-reagent-larval pools were stored at -80°C prior to mRNA extraction. Because pools of larvae were always taken from the same colony, each individual sample (library) represented several larvae (range, 4-41, Table S1) from a single colony, caste, and larval instar.

Following these procedures, we produced 20 samples for mRNA-seq from four queenless and three queenright colonies, representing 3-4 biological replicates per phenotype (Table S1). These consisted of three EQ samples (EQ1-EQ3), three EW samples (EW1-EW3), four MQ samples (MQ1-MQ4), three MW samples (MW1-MW3), four LQ samples (LQ1-LQ4), and three LW samples (LW1-LW3).

*(b) Cohort 2: female larval samples for qRT-PCR*

We obtained eight *B. t. audax* colonies on 20 April 2016 from Biobest UK Ltd. Following the methods to generate female larvae of known caste fate described above, we removed the colony queen from five of the colonies and monitored 228 putatively queen-destined larvae. Of these, 149 unsampled as MQ larvae developed into LQ (of which 21 were sampled as such). As the proportion of unsampled larvae that developed into LQ was high (149/149, or 100%), we identified the sampled larvae as queen-destined larvae (Table S1). Overall, we collected 54 EQ from three colonies, 25 MQ from two colonies, and 21 LQ from five colonies (Table S1), leaving the remaining 128 LQ to develop into adult queens. In the remaining three colonies we left the colony queens in the nests, and monitored 210 putatively worker-destined larvae. Of these, we sampled 78 (as EW, MW, LW) and observed that, of 132 unsampled late-instar larvae, 129 developed into adult workers and three into adult queens. As the proportion of larvae found to develop into workers was high (129/132, or 97.7%), we identified the sampled larvae as worker-destined larvae. We collected 27 EW, 24 MW, and 27 LW from two colonies (Table S1).

The measured head widths and masses of the putatively queen-destined and worker-destined larvae in each instar were consistent with those previously observed for these larval phenotypes (Table S2; Cnaani et al., 1997; Collins et al., 2017). All collected larvae were snap frozen with liquid nitrogen and then homogenised using a chilled ceramic mortar and pestle. Frozen larvae were stored singly at -80°C.

*RNA extraction*

For cohort 1, we extracted total RNA from the 20 pools of larvae stored in tri-reagent following the manufacturer’s instructions. For cohort 2, we randomly selected six larvae of each phenotype for total RNA extraction (36 larvae in total). For both cohorts, we measured the amount of tissue from each stored larva and then suspended the larvae in tri-reagent at a concentration of 100 mg tissue per 1.5ml TRIzol, breaking up clumps of tissue by gently pipetting the entire suspension until the clumps were dispersed. Hence tissue suspensions from larger larvae (e.g. mid- and late-instar larvae) contained fewer individuals than tissue suspensions from smaller larvae (early-instar larvae), which required more individuals to make up the same amount of suspension. To make each library, we removed an aliquot of 1.5 ml from each suspension (therefore ensuring an equal amount of tissue was prepared for each library) and then centrifuged the samples at 10,000 RCF for ten minutes at 4°C. We then removed samples to a fresh Eppendorf tube and left them at room temperature for five minutes. We added 300 µl of chloroform to each tube and left them at room temperature for a further three minutes. We centrifuged each sample at 10,000 RCF for 15 minutes at 4°C, and removed the top phase of each suspension to the spin columns in the Direct-zolTM RNA extraction kit (Zymo Research, Irvine, CA, USA). We followed the Direct-zolTM kit instructions (including a 15 minute on-column DNase treatment), and eluted the final volume twice with the same 20 µl volume of DNase/RNase-free water. We also carried out an additional DNase treatment using the TurboTM DNA-free kit (Thermo Fisher Scientific, Loughborough, UK) according to the manufacturer's protocol.

For both cohorts we quantified the amount of RNA using a Nanodrop 8000 spectrophotometer (ThermoFisher Scientific). We tested that the RNA was not degraded by separating it on a 1.2% agarose gel and confirming the presence of a single band representing the two ribosomal subunits (Winnebeck, Millar, & Warman, 2010). All RNA used for mRNA-seq and qRT-PCR was found to be pure enough for both procedures (260/280 ratio > 1.7), and there was no evidence of RNA degradation. A 2 µg aliquot of RNA from each totalRNA sample was then sent to the Earlham Institute (Norwich, UK), who used equal amounts of RNA to prepare each sample into 20 Illumina Truseq RNA libraries (each containing equal amounts of cDNA) and then sequenced them on five lanes (four randomly selected samples per lane) using 50 bp single-end sequencing on an Illumina HiSeq2500 in high-output mode.

*Bioinformatic analysis: quality control and filtering of reads*

The mRNA- seq returned 893 million reads across all libraries (22-67 million reads per library; Table S9). We carried out an initial quality control of the raw data using the software tool FastQC (Andrews, 2010) to assess sequence reads per sample, mean quality score per base call, GC content, sequence duplication level and presence of overrepresented sequences. We removed all reads containing uncalled bases (bases that could not be assigned to a specific nucleotide), which represented less than 0.1% of all the sequenced reads. We also removed all reads that contained the first 8nt of the Illumina TruSeq Indexed Adapter or the Illumina RNA 3’ Adapter (RA3), and we removed all reads with low sequence complexity (>70% single nucleotide motif or >50% dinucleotide repeat). We observed that there were substantially fewer reads in MQ3 (22,098,444) and LQ3 (27,111,079) compared to the mean number of reads across all libraries (44,665,192; Table S9). As these libraries were prepared with the same amount of input material as the other libraries, and as the samples were equilibrated before being run on the HiSeq2500, we inferred that the low read counts could have been evidence of RNA degradation. We therefore excluded these libraries from further analysis.

After the above quality filtering steps, we calculated the number of non-redundant reads (unique sequences) in each library and calculated the corresponding sample complexities as the number of non-redundant reads as a proportion of the redundant reads (total number of reads) in each library (Table S9; Mohorianu et al., 2011). Across all libraries, 182,303,895 of reads were non-redundant reads, corresponding to (30.2%) of the total number of reads. The mean sample complexity of all quality-filtered reads across all samples was 0.3 (range, 0.23-0.38) after the removal of the low complexity sequences. As sample complexities of all included libraries were similar, libraries were deemed comparable with one another for differential expression analysis (Mohorianu et al., 2017a).

*Bioinformatic analysis: alignment of mRNA-seq reads to the genome*

We aligned quality-filtered reads to reference sequences of annotated features (mRNA, cDNA, coding sequences, exons, genes, non-coding RNA and tRNA) of the *B. terrestris* genome, using PatMaN (Prufer et al., 2008). These reference sequences were derived from the *B. terrestris* reference genome v.1 (Sadd et al., 2015) using the *B. terrestris* genome annotation (GCF_000214255.1_Bter_1.0_genomic.gff downloaded from https://www.ncbi.nlm.nih.gov/genome/?term=txid30195[orgn] on 2015-11-06). Aligning to the reference sequences allowed us to align reads to all previously annotated genes that matched reads in the mRNA-seq libraries. To allow discovery of previously unannotated features (e.g. genes), we also aligned all reads to the full-length *B. terrestris* genome itself. All alignments were performed allowing up to one mismatch between read and reference. The read counts and percentages mapping to each annotation class are summarised in Table S9. We compared the results of the analysis using zero or one mismatchs, and found that allowing one mismatch increased the proportion of reads that aligned to the genome (a mean of 29,604,717 per library for zero mismatches, compared to 33,840,657 if one mismatch was allowed; Table S9). Because of this, and the fact that the genome was sequenced from *B. terrestris terrestris* while the current study used material from *B. t. audax*, we continued the rest of the analysis allowing one mismatch between reads and the reference genome. PatMaN was selected for these alignments because: 1) it is specialised for aligning short reads (<50 bp); 2) it aligns some transcripts missed by other aligners because, unlike other tools, PatMaN does not require an exact match to its ‘seed sequence’ in order to align reads (the seed sequence being a set number of nucleotides in a target read that the aligner compares against a reference sequence, such that if this matches then the aligner compares the next group of nucleotides between the target and reference sequence, and so on until there is a mismatch, or until the whole target is aligned); and 3), unlike other tools, PatMaN efficiently reports reads that align to multiple regions of the genome. PatMaN cannot map reads across introns, and is therefore unable to align reads to unannotated genes that contain multiple exons. Therefore, to account for the presence of unannotated multi-exon transcripts, we assessed the proximity of unannotated transcripts to other annotated and unannotated transcripts (see ‘*Unannotated and Novel genes*’ section below). The read counts and percentages mapping to each annotation class are summarised in Table S9. The annotation class with highest percentage of reads mapping to it was 'gene', and this class was therefore selected as the basis for differential gene expression analysis. Following alignment, we found that 2.5% of reads across all libraries mapped to more than one location on the genome (Table S9). In accordance with best practice (Conesa et al., 2016), we included these reads in the differential expression analysis.

*Bioinformatic analysis: normalisation, unannotated transcript discovery and length correction*

We normalised the quality-filtered sequence reads using the subsampling method (Mohorianu et al., 2017a). This method randomly resamples each FASTA file to a fixed total number of reads corresponding to the library that contains the fewest reads. In the present case, after removal of MQ3 and LQ3, this library was LW1 (33,411,142 reads). Following this step, we employed the methods used above to realign the remaining reads to the genome and to the gene annotation class. The percentages of reads mapping to the genome and to genes did not change substantially as a result of the subsampling procedure (data not shown).

We assembled the sequence reads that matched the reference genome but not any annotated features into contigs (termed 'unannotated transcripts') by joining reads with overlapping positions on the reference genome sequence. We then used PatMaN to align sequence reads to the contigs to estimate the read counts for unannotated transcripts. We combined sample read count files for both types of feature (annotated genes and unannotated transcripts) into expression matrices. We filtered the expression matrices of both types of feature and removed all short transcripts, i.e. transcripts with an average length shorter than 200 base pairs. We thereby removed most small non-coding RNAs (sRNAs) but retained long non-coding RNAs (lncRNAs). We also removed transcripts with low gene expression, i.e. transcripts with a mean abundance (read count) less than 20 and/or transcripts with a zero abundance in more than one out of three samples in the same caste pathway from the same instar. After filtering out these transcripts, the final datasets (one for each larval phenotype) included 10,005 annotated genes and 4,812 unannotated transcripts (i.e. 14,817 features in total).

We then concatenated the files containing all of the remaining genome-matched features (including previously annotated genes and unannotated transcripts) into one matrix containing data for all instars, and adjusted the read counts for each gene in each sample to account for differences in length between transcripts by multiplying each value by a correction factor calculated as 1000/length of the gene transcript in base pairs. We then normalised the data using quantile normalisation (Bolstad, Irizarry, Astrand, & Speed, 2003).To test the outcome of the normalisation we compared the similarity of individual libraries using pairwise Jaccard indices, i.e. similarity coefficients (Beckers et al., 2017; Mohorianu et al., 2017a), as well as a multi-dimensional scaling plot (MDS; (Torgerson, 1952)), which visualised the clustering of libraries based on Manhattan distance between libraries (Figure S5). We visually inspected the output of the Jaccard index and MDS analyses to determine if biological replicates were clustering more closely to each other than to other libraries. Due to lower Jaccard similarity to, and (in the MDS plots) poor clustering with, the other replicate samples from the same caste and larval instar, we excluded MW1 from raw data matrices, and these matrices were then length corrected and quantile normalised as above with MW1 excluded. We then retested the outcome of normalisation with a second MDS that showed greater similarity between replicates on co-ordinate 1 of the MDS (Figure S4). To further assess the quality of the normalisation, determining whether libraries representing gene expression were comparable between different replicates and phenotypes, we constructed and visually inspected boxplots, scatterplots and MA-plots (Figures S6, S7, S8). Since the boxplots representing the distribution of gene expression values in each sample were very similar between samples (the expected outcome of quantile normalisation), and the pairwise scatter- and MA-plots showed the symmetrical funnel-shape expected from sample pairs with no major systematic differences in expression patterns (particularly between replicate samples), we considered the data sufficiently normalised to continue differential gene expression analysis (see main text, methods: ‘Differential gene expression between caste phenotypes’). We refer to the overall method used for aligning reads and calculating differential gene expression as the ‘PatMan/confidence-interval pipeline’ to contrast it with other pipelines for isolating differentially expressed genes (DEGs). A previous study using similar methods to those described here found a high degree of validation (78%) between mRNA-seq data that were analysed using the PatMan/confidence-interval pipeline and qRT-PCR data (Mohorianu et al., 2017b).

*Bioinformatic analysis: comparison between the PatMan/confidence-interval pipeline and Kallisto and HISAT2/HTSeq pipelines*

To confirm that the PatMan/confidence-interval pipeline used in the current study produced results comparable with those from analysis pipelines used in similar mRNA-seq studies, we analysed our mRNA-seq data using two alternative pipelines. The two pipelines selected for comparison were chosen because they use software found to perform well in a recent comparison ([Sahraeian et al. 2017](https://paperpile.com/c/P8VeBX/Stkc)). In addition, each has recently been used in mRNA-seq studies in *B. terrestris* (Bebane et al., 2019; Colgan et al., 2019a; Colgan, Carolan, Sumner, S, Blaxter & Brown, 2019b). The pipelines selected were: 1) pseudoalignment of reads to the *B. terrestris* transcriptome (Bombus_terrestris.Bter_1.0.cdna.all.fa) with Kallisto v0.46.1 (Bray, Pimentel, Melsted, & Pachter, 2016), with estimated transcript counts being summarised per gene with tximport v1.16.1 (Soneson, Love, & Robinson, 2016), followed by differential gene expression analysis with DESeq2 v1.28.1 (Love, Huber, & Anders, 2014) using an FDR adjusted *p-*value threshold of 0.05; and 2) splice-aware alignment of reads to the *B. terrestris* genome (Bombus_terrestris.Bter_1.0.dna.toplevel.fa) with HISAT2 v2.1.0 (Kim, Langmead, & Salzberg, 2015), extraction of gene-level read counts using HTSeq-count v0.11.2 (Anders, Pyl, & Huber, 2014) with Python v3.7 (Van Rossum & Drake, 2009), and differential gene expression analysis with DESeq2 using the same parameters as for the Kallisto pipeline. Both pipelines were implemented in R v4.0.1 (R Core Team 2020).

We used the Kallisto and HISAT2/HTSeq pipelines to analyse the same mRNA-seq libraries as those analysed in the PatMan/confidence-interval pipeline (i.e. libraries MQ3, LQ3, and MW1 were not included in the analysis) and focussed on annotated genes, as Kallisto is not able to discover unannotated genes. The pseudoalignment of reads with Kallisto and splice-aware alignment of reads with HISAT2 were summarised using MultiQC v1.9 (Ewels, Magnusson, Lundin, & Käller, 2016) with Python v3.7. Boxplots of the normalised count data and principal component analysis from DESeq2 were produced for each pipeline to check normalisation and library clustering respectively (data not shown). The annotated *B. terrestris* DEGs determined by the Kallisto and HISAT2/HTSeq pipelines were then compared (by matching the mapped transcript in each case by its NCBI gene symbol) and custom R scripts were used to determine the level of overlap with: 1) the annotated *B. terrestris* DEGs from the PatMan/confidence-interval pipeline (Figure S9); and 2) the annotated *B. terrestris* HDEGs from the PatMan/confidence-interval pipeline (Figure S10). All scripts necessary to recreate the analysis are available at <https://github.com/dhcollins500/Collins-et-al_BB-M001482-1_obj1>, supplementary file 4.

These analyses showed that the level of overlap between results from the PatMan/confidence-interval pipeline and the two comparison pipelines was high. On average, across phenotypes, 80.8% of genes identified as DEGs using Kallisto and 84.9% of genes identified as DEGs using HISAT2/HTSeq were identified as DEGs using the PatMan/confidence-interval pipeline (Table S3). In addition, the PatMan/confidence-interval pipeline identified many more DEGs than the other pipelines, as an average of 25.7% and 22.6% of the genes identified as DEGs in the PatMan/confidence-interval pipeline were identified as DEGs using Kallisto and HISAT2/HTSeq, respectively (Table S3). For HDEGS, an average of 2.3% of genes identified as DEGs by Kallisto and an average of 2.6% of genes identified as DEGs by HISAT2/HTSeq were identified as HDEGs in the PatMan/confidence-interval pipeline, and an average of 80.4% and 80.6% of genes that were identified as HDEGs in the PatMan/confidence-interval pipeline were identified as DEGs by Kallisto and HISAT2/HTSeq, respectively (Table S3). These proportions compared well with the overlap in DEGs between the two alternative pipelines, as Kallisto identified an average of 90.9% of the genes classified as DEGs in HISAT2/HTSeq, and HISAT2/HTSeq identified an average of 68.5% of the genes identified as DEGs in Kallisto (Table S3). The focus of the analyses in the current study on HDEGs (on the assumption that genes with stronger differences are likely to be more important for caste determination and/or differentiation) protected the conclusions against the possibility that some of the DEGs predicted by the PatMan/confidence-interval pipeline were false positives (given that this pipeline predicted many more DEGs than the alternative pipelines), as did the fact that a high proportion of the HDEGs were predicted by the two alternative pipelines.

The use of different pipelines did not affect the main conclusions of the study as all HDEGs classed as genes of interest (and therefore identified as the genes most likely to affect caste determination and/or differentiation) after being predicted using the PatMaN/confidence-interval pipeline were also called as differentially expressed by the other two pipelines. Furthermore, the conclusion that caste-associated differences in gene expression are low in early-instar larvae, and higher in mid- and late-instar larvae was not affected by the pipeline used, i.e. for all three pipelines there were few or no differences (depending on whether DEGs or HDEGs were considered) in gene expression between caste pathways in the early-instar larvae, whereas in mid- and late-instar larvae the differences were much greater (Table S3).

We used the R package ‘pheatmap’ to produce a heatmap of the 50 most strongly differentially expressed genes from the PatMan/confidence-interval pipeline. These were then clustered to produce a dendrogram of the most closely related gene clusters using the ‘cluster_rows’ argument in the pheatmap function associated with the pheatmap package. All R scripts and accompanying files for these analyses are available at https://github.com/dhcollins500/Collins-et-al_BB-M001482-1_obj1.

*Unannotated and novel genes*

We isolated unannotated transcripts from the list of HDEGS (before accounting for multiple unannotated exons) from each caste and instar. To assess whether these unannotated transcripts either 1) were unannotated exons of previously annotated genes, or 2) represented the exons of larger unannotated transcripts, we estimated inter-exon distances of previously annotated genes in our dataset using the *B. terrestris* genome annotation (GCF_000214255.1_Bter_1.0_genomic.gff downloaded from https://www.ncbi.nlm.nih.gov/genome/?term=txid30195[orgn] on 2015-11-06; (Sadd et al., 2015)). This was to allow us to account for the fact that PatMaN does not map transcripts across introns. We used the information from all 20 libraries (including those excluded for differential expression analysis) to isolate all quality-filtered (but not sub-sampled for normalisation) reads aligning to previously annotated genes (in total 535,251,193 reads from 20 libraries) to generate read counts for each nucleotide position within the reference sequence of each gene. The length of stretches of zero count nucleotides within genes (i.e. introns) were recorded and the median, 5th, and 95th percentile of distances between stretches of zero count nucleotides were computed across 11,134 genes. The 95th percentile, 810 bp, was used as a cut-off below which transcripts were treated as part of the transcript/gene next to it, if they were differentially expressed in the same direction as this transcript with an OFC equal to or greater than 1. Therefore, we identified each highly significantly differentially expressed unannotated transcript as: 1) an unannotated exon of an annotated gene if it was within 810 bp of a neighbouring annotated gene and was differentially expressed in the same direction; 2) part of a larger unannotated multi-exon transcript if it was within 810 bp of one or more neighbouring unannotated genes that was differentially expressed in the same direction; 3) a single-exon transcript if it was not within 810 bp of any other transcripts. Following this analysis, we removed the feature NC_015772.1_16036258_16037622 from the list of HDEGs in MQ (reducing the number of HDEGs in MQ from 58 to 57) from further analysis because it was within 810 bp of estradiol 17-beta-dehydrogenase 11. (LOC100652203). We also combined six features into three unannotated multi-exon transcripts in MQ and four features into two multi-exon transcripts in MW, so reducing the total numbers of HDEGs in these phenotypes to 54 and 92, respectively (Table S10). By combining these results with the official *B. terrestris* annotations (Sadd et al, 2015), we were able to isolate and map all annotated and unannotated exons for all HDEGs. Therefore, the final list of HDEGs (after accounting for multiple exons) was 200.

We tested the coding-potential of each novel gene using the Coding Potential Assessment Tool (CPAT) (Wang et al., 2013) application within Blast2GO v4.1.9 (Conesa et al., 2005). This tool produces a binary output that we used to classify each of the novel genes as either ‘protein-coding’ or ‘non-coding RNA’. As there were no HDEGs in the early-instar larvae, we excluded EQ and EW from this analysis. Using these criteria, we were able to classify each HDEG into one of the four annotation classes described in the main text (section, ‘*Unannotated and novel genes*’).

*Gene Ontology (GO) enrichment analysis*

We used a reciprocal best-hit (RBH) protein BLAST search between *B. terrestris* (from the annotated list of proteins available at https://www.ncbi.nlm.nih.gov/assembly/GCF_000214255.1/) and a *Drosophila melanogaster* list of proteins (v6) (Hoskins et al., 2015) to produce a list of orthologs (including single- and multi-gene orthologs) between the two species (Table S4; Bork et al., 1998; Tatusov, Koonin, & Lipman, 1997). We downloaded the protein sequences for *B. terrestris* and *D. melanogaster* in FASTA format and used these to create BLAST databases in CyVerse (Merchant et al., 2016) using the 'Create BLAST database' app and default settings. We identified the orthologs between the two species by BLASTing each file against the other using the BLASTp 2.2.29+ app in CyVerse and default settings. We determined the RBH for each protein sequence (scripts available at https://github.com/dhcollins500/Collins-et-al_BB-M001482-1_obj1). We then used a *B. terrestris* annotation file and the *D. melanogaster* genome-wide annotation package for R (Carlson M. org.Dm.eg.db.v3.7.0 ) to identify the genes corresponding to the proteins and produce a list of orthologs between the two species (Table S4).

Using GOrilla software (Eden, Navon, Steinfeld, Lipson, & Yakhini, 2009), we conducted a GO term enrichment analyses, removing redundant terms using Revigo (Supek, Bosnjak, Skunca, & Smuc, 2011). Due to the large number of GO terms, we restricted our analysis to the ‘Biological Function’ ontology. We isolated the *D. melanogaster* orthologs of the *B. terrestris* DEGs in each phenotype (DEOs). We did not conduct GO analyses using the HDEGS as the number of *D. melanogaster* orthologs of HDEGs were too low for an informative GO analysis, ie. Zero HDEGs in EQ, and EW phenotypes, and 19, 11, 6, and 1 HDEG for the MQ, MW, LQ, and LW phenotypes respectively.

We used GOrilla to compare the *D. melanogaster* orthologs in the DEG list for each phenotype (the target set) against the unique *D. melanogaster* genes in the orthologs list (the background set; Table S13), using a p-value threshold of 10^-3^. Therefore each list of DEGs from each phenotype was compared to all of the *B. terrestris* orthologs in *D. melanogaster*. We then identified the GO terms that were significantly enriched in each phenotype.

Selection of genes of interest for qRT-PCR

The selected genes of interest that were HDEGs (Figure 4) fell within five categories (note that these differ from the five selection criteria), based on the mRNA-seq data: 1) HDEGs that were the most highly differentially expressed in each phenotype: in MQ, Pancreatic lipase-related protein 2-like (Plrp2-like), in MW, P17/29C-like, in LQ, Plrp2-like (the same gene being the most highly differentially expressed gene in both LQ and MQ), in LW, uncharacterised protein (XM_003399878.3); 2) genes associated with JH signalling: Hexamerin (XM_003401733.3), Krüppel homolog-1 (*Kr-h1*; NM_001280921.1), and Takeout (XM_003397243.2), which were all upregulated in MQ; 3) genes associated with caste differentiation in *A. mellifera* : Chymotrypsin-2-like (XM_012309549.1, upregulated in LQ), and Yellow (XM_003399634.3, upregulated in MW); 4) genes in the Cytochrome P450 (CYP) gene family, which were mostly associated with queen-destined larvae (among HDEGs, 4/54 and 5/40 CYP family genes were upregulated in LQ and MQ, respectively), with examples including: CYP6A1 (XM_012315471.2, upregulated in LQ), CYP6k1 (XM_012314831.2, upregulated in LQ), and CYP305a1 (XM_003396620.3, upregulated in MQ); some CYP HDEGs were also upregulated in MW (4/92 genes in total), e.g. another version of CYP6k1 (XM_012314843.2); and 5) Nose resistant to fluoxetine proteins (Nrf), which were upregulated in queen-destined but not worker-destined larvae and represented in 5/40 and 6/54 HDEGs in LQ and MQ respectively, with HDEGs upregulated in both MQ and LQ including Nrf-6 (XM_012321236.2) and Nrf-like (XM_012319905.2).

*Primer design and qRT-PCR*

We designed primers for the 16 target genes and four candidate reference genes using the NCBI primer BLAST (https://www.ncbi.nlm.nih.gov/tools/primer-blast/) according to specifications in Thornton and Basu (2011) and Bustin et al. (2009). For primers of four additional reference genes, we used published sequences (Table S6). To ensure that the primers we designed would not form dimers, we used the Oligo Analysis function on the Beacon primer design website (http://www.premierbiosoft.com/qOligo/Oligo.jsp?PID=1). We then used the UNAfold function on the IDT website (https://www.idtdna.com/UNAFold) to select only primer pairs that did not produce amplicons that formed stable secondary structures at an annealing temperature of 60°C. We designed between three and seven primer pairs for each of the 16 target genes (plus one gene that was later excluded as we could not design adequate primers for it) and four (of the total of eight) candidate reference genes.

We synthesised cDNA from 36 RNA samples generated from individual larval samples (see main text, methods: ‘Sample collection’) using the nanoScript 2 RT kit (Primer Design), with 2 µg of RNA per reaction. We ran qRT-PCR reactions in 20 μL reactions containing 5 μL of the diluted cDNA or no-RT control (where transcriptase had not been added to RNA during the cDNA synthesis step), 0.5 μM of each primer (Table S6), and PrecisionPLUS MasterMix with low ROX and premixed SYBRgreen (Primer Design). We ran each reaction on a 96-well PCR plate (Thermo Scientific) using the ABI 7500 Real Time qPCR system (ABI), and set up the following thermocycle: 3 min at 95°C, then 40 cycles of i) 30 s at 95°C, ii) 30 s at 62°C, and iii) 30 s at 72°C, followed by melt curve analysis (65–95°C at 0.5°C increments, 5 s for each). We ran an initial reaction to test the specificity of each primer pair, retaining only primer pairs that had high specificity to a given target gene and discarding one gene for which we were unable to design specific primer pairs (Nrf6-like, LOC100645518). For each shortlisted primer pair we used a 1 in 5 serial dilution of the cDNA (repeated five times) to calculate the efficiency of the primers using standard curves and selected the primer pair that had the highest efficiency rating for each gene (ranging from 73.3-104.3%; Table S6). We then ran qRT-PCR reactions using the selected primer pair for each target and reference gene in each of the six phenotypes (EW, MW, LW, EQ, MQ, LQ) using six biological replicates per phenotype, where each replicate for all six phenotypes consisted of RNA extracted from a single larva (labelled q1-6 for each phenotype, e.g. second LW larva = LW.q2; Table S1). Each reaction was run alongside its corresponding no RT control to ensure that no genomic DNA was carried over from the RNA extraction and DNase treatment stages (therefore causing a gene to be amplified by qRT-PCR even in the absence of gene expression). In addition, for each primer pair we also ran no-template controls and no-reverse-transcription controls. As the number of reactions for each gene exceeded the number that could be run on a single plate, an interplate calibrator (IPC) was run on each plate. For each gene the IPC was selected as the cDNA sample that produced the lowest Cq value (threshold cycle) or second lowest if there was too little cDNA left after the first reaction to run a second time. This sample was then run on a second plate alongside the remaining samples. We ran each reaction in triplicate and discarded replicates that were more than one Cq value different from the other two technical replicates (98/900 reactions). If all three technical replicates were more than 1 Cq value apart from one another, the reactions were re-run until at least two reactions were within 1 Cq value of one another, with four reactions being re-run in triplicate a second time (and no reactions being re-run a third time). We calculated the cycle threshold for each reaction as the mean of the two or three technical replicates.

We used the IPC to normalise the Cq values for the same gene run on different plates (Hellemans, Mortier, De Paepe, Speleman, & Vandesompele, 2007). We did this by dividing the Cq value of the Target/Reference gene by the Cq value of the IPC that was run on its corresponding plate and multiplying the resulting value by the mean of the two IPC values. We analysed the normalised Cq values of the eight candidate reference genes and determined the appropriate number and most stable reference genes using geNorm (Vandesompele et al., 2002) and BestKeeper software (Michael W. Pfaffl, 2004). To conduct the geNorm analyses, we used the ReadqPCR and NormqPCR packages in R. We used the three most stable reference genes (see main text, methods: ‘Primer design and qRT-PCR’) to calculate the BestKeeper index for normalisation of the qRT-PCR data across all six phenotypes and biological replicates (Figure S11). We used equation 1 in Pfaffl (2001) to calculate the relative quantification for each gene. For each biological replicate we calculated the numerator of equation 1 in Pfaffl(2001) using 1) the calculated efficiency of the target gene, 2) the normalised Cq values of the target gene, and 3) the Cq value of the reference sample. We calculated the denominator of equation 1 in Pfaffl(2001) using 1) the arithmetic mean of the efficiency of the three selected reference genes, 2) the BestKeeper index for each sample, and 3) the BestKeeper index of the reference sample (arbitrarily selected to be EQ.q3). To calculate relative quantification we then log_2_-transformed the resulting values. For a given target gene we used Mann-Whitney U-tests to test for significant differences in relative quantification between queen and worker-destined larvae within each larval stage i.e. early-instar (EQ vs EW), mid-instar (MQ vs MW), and late-instar larvae (LQ vs LW). For each gene and each phenotypic comparison, we tested the hypothesis that the relatively quantified Cq values would show the same result as the mRNA-seq data with a binomial test. For this, we defined a match between the mRNA-seq and qRT-PCR results for a given gene and larval (developmental) stage as both methods showing significant differential expression in the same direction (DEGs with OFC >1 for mRNA-seq, p < 0.05 for qRT-PCR), or both showing no differential expression, and a mismatch as either of these not occurring. The binomial test then tested the null hypothesis that the proportion of matches was greater than 50%.

In addition, we calculated Pearson correlation coefficients between average read count for each phenotype in the mRNA-seq data and the average relative quantification value for each phenotype in the qRT-PCR data (n = 6 for each gene). This also showed a high degree of correlation for 11/16 of the genes of interest, indicating a high degree of congruence between the mRNAseq and qRT-PCR results (Figure S13). All R scripts and accompanying files for these analyses of the qRT-PCR data are available at https://github.com/dhcollins500/Collins-et-al_BB-M001482-1_obj1.

## Comparative analysis of caste-associated genes in larvae

Direct gene list comparative analysis

To identify the genes involved in caste determination in both *B. terrestris* and *Apis mellifera*, we conducted a RBH BLAST search (Bork et al., 1998; Tatusov et al., 1997) between the annotated list of proteins for *B. terrestris* (Sadd et al., 2015) and the *A. mellifera* genome (Elsik et al., 2014; Weaver et al., 2007) to produce a list of orthologs between the two species (Table S7) using the same procedure as for the RBH BLAST between *B. terrestris* and *D. melanogaster*.

*B. terrestris* larvae undergo four instars, and the most influential, queen-dependent period of caste determination occurs towards the end of the second instar (see main text, Introduction; and Figure S1). *A. mellifera* undergo five instars. Queen-destined larvae hatch in specialised queen cells and are fed royal jelly throughout development, and worker-destined larvae hatch in worker cells and are fed worker jelly throughout development (Haydak, 1970), with caste-specific characteristics appearing in larvae 40-48 hours (during their second larval instar) after hatching (Winston, 1987). This period of caste determination corresponds to a spike in JH in queen-destined larvae and evidence of caste-specific gene expression (Wheeler, Buck, & Evans, 2014). To account for the differences in caste determination mechanisms, timing of the periods of caste determination, and number of instars between *B. terrestris* and *A. mellifera*, we made the following comparisons between our data and data from the two selected *A. mellifera* studies (see main text, methods: ‘Comparative analysis of genes associated with larval caste determination in *B. terrestris* and *A. mellifera*’; and Figure S12): EQ and EW with the first four time-steps (corresponding to the first two instars in *A. mellifera*) in Cameron, Duncan, and Dearden (2013) and the first time-step (corresponding to the second to third instar in *A. mellifera*) in He, Jiang, Zhou, Barron, and Zeng (2017); MQ and MW with the third, fourth and fifth time-steps (corresponding to the second, third, and fourth instar in *A. mellifera*) in Cameron et al. (2013) and the first and second time-step (corresponding to the second to third and fourth to fifth instar in *A. mellifera*) in He et al. (2017) ; and LQ and LW with the fourth, fifth, sixth, and seventh time-steps (corresponding to the third, fourth, and fifth instar in *A. mellifera*) in Cameron et al. (2013) and the first and second time-steps (corresponding to the second to third and fourth to fifth instar in *A. mellifera*) in He et al. (2017; Table S8). Cameron et al. (2013) used microarrays to isolate caste-associated genes in all seven of their timepoints, and mRNA-seq to isolate caste-associated genes at their 60 h timepoint. He et al. (2017) used mRNA-seq to isolate caste-associated genes at two timepoints. Therefore, we compared our data with the microarray data in Cameron et al. (2013) and the mRNA-seq data in He et al. (2017). We constructed contingency tables consisting of orthologs that were differentially expressed between caste pathways in both *B. terrestris* and *A. mellifera*, orthologs that were differentially expressed between caste pathways in *B. terrestris* only, orthologs that were differentially expressed between caste pathways in *A. mellifera* only, and orthologs that were not differentially expressed between caste pathways in either study. Overall, we made a total of 36 direct gene list comparisons between our data and the data presented in Cameron et al. (2013), and 18 direct gene list comparisons between our data and the data presented in He et al. (2017; Table S8). We then performed a Fisher's Exact Test on each pair of compared lists of orthologs to detect significant overlaps between each pair. To correct for multiple testing, we used a Bonferroni correction to adjust the alpha value for each hypothesised comparison of each phenotype (Table S8). All R scripts for this analysis are available at <https://github.com/dhcollins500/Collins-et-al_BB-M001482-1_obj1>.

HISAT2/HTSeq comparative analysis

To control for technical or methodological differences affecting the conclusions, we supplemented the previous analysis by using mRNA-seq data that we reanalysed with a shared pipeline across all three studies. Here, we refer to this analysis as the HISAT2/HTSeq comparative analysis to distinguish it from the Direct gene list comparative analysis discussed above and in the main text. We therefore analysed the raw mRNA-seq data (but not the microarray data) of Cameron et al. (2013) and the raw mRNA-seq data of He et al. (2017) using the HISAT2/HTSeq pipeline, for comparison with the *B. terrestris* mRNA-seq data from the current study analysed with HISAT2/HTSeq as described above. The data of Cameron et al. (2013) and He et al. (2017) were downloaded from the Sequence Read Archive (SRA) using SRA Toolkit v2.10.2 (http://ncbi.github.io/sra-tools/) and the accession numbers in supplementary file 5. The HISAT2/HTSeq pipeline (see *Bioinformatic analysis: comparison between the PatMan/confidence-interval pipeline and Kallisto and HISAT2/HTSeq pipelines* for details) was used to align the mRNA-Seq reads to the *A. mellifera* genome (Apis_mellifera.Amel_4.5.dna.toplevel.fa) and determine significantly differentially expressed genes. Following this, we made the following comparisons between studies: EQ and EW with the 60 h time-step (corresponding to the third instar in *A. mellifera*) in Cameron et al., (2013) and the first time-step (corresponding to the second to third instar in *A. mellifera*) in He et al., (2017); MQ and MW with the 60 h timestep in Cameron et al. (2013) and the first and second time-step (corresponding to the second to third and fourth to fifth instar in *A. mellifera*) in He et al. (2017); and LQ and LW with the 60 h time-steps in Cameron et al. (2013) and the first and second time-steps in He et al. (2017; Table S16). We constructed contingency tables as described above (see ‘Direct gene list comparative analysis’). Overall, we made a total of 6 comparisons between our data and the data presented in Cameron et al. (2013) and 10 comparisons between our data and the data presented in He et al. (2017; Table S16). We then performed a Fisher's Exact Test on each pair of compared lists of orthologs as described above (see ‘Direct gene list comparative analysis’). All R scripts for this analysis are available at <https://github.com/dhcollins500/Collins-et-al_BB-M001482-1_obj1>.

Following analysis of all datasets using the HISAT2/HTSeq comparative analysis, we isolated 8,027 orthologs that were present in the mRNA-seq data (including genes that were not differentially expressed) from *B. terrestris* (current study) and in the mRNA-seq data from *A. mellifera* in Cameron et al (2013) when using the HISAT2/HTSeq pipeline to isolate gene lists from both studies. Overall, 3/6 of the ortholog comparisons showed significant overlaps between the current study and Cameron et al (2013; Table S16). The significant overlaps were: 1) 33/460 (7.17%) of the orthologs that were upregulated in MQ in *B. terrestris* were also upregulated in 60 h old queen-destined larvae (relative to worker-destined larvae) in *A. mellifera* (Fisher’s Exact Test, p = 1.5 × 10^-3^; Figure S14A); 2) 69/365 (18.9%) of the orthologs that were upregulated in LQ in *B. terrestris* were also upregulated in 60 h queen-destined larvae in *A. mellifera* (p = 5.5 × 10^-13^; Figure S14B); 3) 11/628 (1.75%) of the orthologs that were upregulated in LQ in *B. terrestris* were also upregulated in 60 h queen-destined larvae in *A. mellifera* (p = 1.1 × 10^-3^; Figure S14C).

We isolated 8,064 orthologs that were present in the mRNA-seq data (including genes that were not differentially expressed) from *B. terrestris* (current study) and in the mRNA-seq data from *A. mellifera* in He et al (2017) when using the HISAT2/HTSeq pipeline to isolate gene lists from both studies. Overall, 3/10 of the ortholog comparisons showed significant overlaps between the current study and He et al (2017; Table S16). The significant overlaps were: 1) 44/365 (12.05%) of the orthologs that were upregulated in MW in *B. terrestris* were also upregulated in four day old worker-destined larvae (relative to queen-destined larvae) in *A. mellifera* (Fisher’s Exact Test, p = 3.0 × 10^-9^; Figure S14D); 2) 114/460 (24.78%) of the orthologs that were upregulated in MQ in *B. terrestris* were also upregulated in four day old queen-destined larvae (relative to worker-destined larvae) in *A. mellifera* (Fisher’s Exact Test, p = 1.8 × 10^-10^; Figure S14E); 2) 103/365 (28.22%) of the orthologs that were upregulated in MW in *B. terrestris* were also upregulated in four day old worker-destined larvae in *A. mellifera* (p = 3.0 × 10^-13^; Figure S14F).

Overall, we identified six significant overlaps between the current study and the *A. mellifera* studies using the HISAT2/HTSeq comparative analysis (compared to seven using the Direct gene list comparative analysis), but the level of overlap was low (range, 33-114 genes; % range, 1.75-28.22%; Figure S14, Table S16). The significant comparisons were similar irrespective of the comparison analysis methods used. Of the seven significant comparisons identified using the Direct gene list comparative analysis, five were the same as the significant comparisons found when using the HISAT2/HTSeq comparative analysis. Of the two remaining significant comparisons that were significant using the Direct gene list comparative analysis but not shown to be significant using the HISAT2/HTSeq comparative analysis, one was with the microarray data from Cameron et al., 2015 (LQ vs 108 h queen-destined larvae) and therefore was not analysed using the HISAT2/HTSeq. Of the six significant comparisons identified using HISAT2/HTSeq comparative analysis, five were the same as the significant comparisons using the Direct gene list comparative analysis. Hence the method used to identify significant overlaps did not have a strong effect on the significant overlaps that were identified.

As with the Direct gene list comparison, three of the genes that were upregulated in both species were among the target genes selected for qRT-PCR (Hexamerin, XM_003401733.3; *Kr-h1*, NM_001280921.1; and probable CYP303a1, XM_003396659.3).

Diapause genes analysis

The mRNA-Seq data from Amsalem et al. (2015) were downloaded from the SRA using SRA Toolkit v2.10.2 and the accession numbers in supplementary file 5. Data were from five biological replicates (individual queens of different genetic lines) of the fat body of three stages of the *B. terrestris* adult queen lifecycle: 24 hours post-mating (mated); within seconds of ending diapause (diapause); and after egg laying post diapause (founding post diapause). FastQC v0.11.9 (Andrews, 2010) analysis was conducted to assess the quality of the raw data. The HISAT2/HTSeq pipeline (see *Bioinformatic analysis: comparison between the PatMan/confidence-interval pipeline and Kallisto and HISAT2/HTSeq pipelines* for details) was used to align the mRNA-Seq reads to the *B. terrestris* genome (Bombus_terrestris.Bter_1.0.dna.toplevel.fa). Inspection of the FastQC and HISAT2 alignment results led to exclusion of mated (M) line 207 from further analysis as only 17.1% of reads mapped uniquely to the genome (compared to at least 79% for all other samples) and there was a low percentage of unique reads, different per sequence GC content and different sequence duplication level profiles compared to all other samples, and a relatively high percentage of overrepresented sequences (7.18%). Principal component analysis of the DESeq2 results clustered diapause (D) line 141 with mated and founding post-diapause samples, rather than with diapause samples, and therefore D line 141 was excluded and the DESeq2 analysis re-run to identify DEGs between mated and diapause queens, and between diapause queens and founding post-diapause queens. Following the method of Amsalem et al. (2015), we defined genes uniquely regulated in diapause as genes that were up-regulated in diapause and down-regulated in both mated and founding post-diapause, or vice versa (e.g. genes that overlapped in the up-regulated DEGs lists between mated and diapause, and diapause and founding post-diapause). The lists of up-regulated and down-regulated diapause specific genes were then combined.

To determine if diapause-associated genes were overrepresented in the lists of *B.terrestris* larval caste-associated genes from the current study, we used Fisher's Exact Tests with Bonferroni correction to test whether the combined list of diapause-associated genes (1,096 genes) significantly overlapped with each list of caste-associated genes that were significantly upregulated (in queens vs workers or workers vs queens within each timepoint) using the HISAT2/HTSeq analysis (i.e. EQ, EW, MQ, MW, LQ, LW). We also tested for overlaps between the diapause genes and the combined lists of caste-associated genes (queen- and worker-pathway) at each timepoint (early, mid, late).

Diapause-associated genes showed no significant overrepresentation among the significantly differentially expressed caste-associated genes at each developmental timepoint (Fisher’s Exact Test: EQ, p = 1.000; EW, p = 1.000; MQ, p = 0.939; MW, p = 0.534; LQ, p = 0.949; LW, p = 0.356; early, p = 1.000; mid, p = 0.656; late, p = 0.570;Table S17).

We also excluded the *B. terrestris* diapause-associated genes (or their *A. mellifera* orthologs) from the lists of significantly differentially expressed genes in the current study (i.e. *B. terrestris* larval dataset), the Cameron et al. (2013) study (60 h timepoint), and the He et al. (2017) study, in order to repeat the HISAT2/HTSeq comparative analysis without the potentially confounding effects of diapause-associated genes. This had little effect on the results of the HISAT2/HTSeq comparative analysis as all six of the comparisons that were significant with the diapause genes included remained significant once they were excluded, and no additional comparisons were shown to be significant whether the diapause genes were included or excluded (Table S16). In addition, the numbers of genes that overlapped with the diapause genes included (range, 33-114 genes; % range, 1.75-28.22%) were similar to the numbers of genes that overlapped when the diapause genes were excluded (range, 11-100 genes; % range, 1.9-26.8%; Table S16).

All scripts used to conduct the diapause genes analysis are available at <https://github.com/dhcollins500/Collins-et-al_BB-M001482-1_obj1>, supplementary file 5.

A flowchart summarising all methods used in the current study is provided in Figure S2.

# Supplemental References

Alford, D. V. (1975). *Bumblebees*. London, UK: Davis-Poynter.

Amsalem, E., Galbraith, D. A., Cnaani, J., Teal, P. E. A., & Grozinger, C. M. (2015). Conservation and modification of genetic and physiological toolkits underpinning diapause in bumble bee queens. *Molecular Ecology*, *24*, 22. doi: 10.1111/mec.13410

Anders, S., Pyl, P. T., & Huber, W. HTSeq—a Python framework to work with high-throughput sequencing data. *Bioinfomatics*, *31* (2), 166-169. doi: 10.1093/bioinformatics/btu638

Andrews, S. (2010). FastQC: a quality control tool for high throughput sequence data. Available online at: <http://www.bioinformatics.babraham.ac.uk/projects/fastqc>.

Bebane, P. S. A., Hunt, B. J., Pegoraro, M., Jones, A. R. C., Marshall, H., Rosato, E., & Mallon, E. B. (2019). The effects of the neonicotinoid imidacloprid on gene expression and DNA methylation in the buff-tailed bumblebee *Bombus* *terrestris*. *Proceedings of the Royal Society B-Biological Sciences*, *286*(1905), 20190718. doi: 10.1098/rspb.2019.0718

Beckers, M., Mohorianu, I., Stocks, M., Applegate, C., Dalmay, T., & Moulton, V. (2017). Comprehensive processing of high-throughput small RNA sequencing data including quality checking, normalization, and differential expression analysis using the UEA sRNA Workbench. *RNA*, *23*(6), 823-835. doi: 10.1261/rna.059360.116

Bolstad, B. M., Irizarry, R. A., Astrand, M., & Speed, T. P. (2003). A comparison of normalization methods for high density oligonucleotide array data based on variance and bias. *Bioinformatics*, *19*(2), 185-193. doi: 10.1093/bioinformatics/19.2.185

Bork, P., Dandekar, T., Diaz-Lazcoz, Y., Eisenhaber, F., Huynen, M., & Yuan, Y. (1998). Predicting function: from genes to genomes and back. *Journal of Molecular Biology*, *283*(4), 707-725. doi: 10.1006/jmbi.1998.2144

Bray, N., Pimentel, H., Melsted, P., & Pachter, L. (2016). Near-optimal probabilistic RNA-seq quantification. *Nature Biotechnology*, *34*, 525–527. doi: 10.1038/nbt.3519

Bustin, S. A., Benes, V., Garson, J. A., Hellemans, J., Huggett, J., Kubista, M., . . . Wittwer, C. T. (2009). The MIQE Guidelines: Minimum Information for Publication of Quantitative Real-Time PCR Experiments. *Clinical Chemistry*, *55*(4), 611-622. doi: 10.1373/clinchem.2008.112797

Cameron, R. C., Duncan, E. J., & Dearden, P. K. (2013). Biased gene expression in early honeybee larval development. *BMC Genomics*, *14*, 903. doi: 10.1186/1471-2164-14-903

Cnaani, J., Borst, D. W., Huang, Z. Y., Robinson, G. E., & Hefetz, A. (1997). Caste determination in *Bombus terrestris*: Differences in development and rates of JH biosynthesis between queen and worker larvae. *Journal of Insect Physiology*, *43*(4), 373-381. doi: 10.1016/S0022-1910(96)00106-0

Colgan, T.J., Fletcher, I.K., Arce, A.N., Gill, R. J., Rodrigues, A. R., Stolle, E., . . . Wurm, Y. (2019a). Caste‐ and pesticide‐specific effects of neonicotinoid pesticide exposure on gene expression in bumblebees. *Molecular Ecology*, *28*, 1964– 1974. doi: 10.1111/mec.15047

Colgan, T.J., Carolan, J. C., Sumner, S., Blaxter, M. L., & Brown, M. J. F. (2019b). Infection by the castrating parasitic nematode *Sphaerularia bombi* changes gene expression in *Bombus terrestris* bumblebee queens. *Insect Molecular Biology*, *29* (2), 170 – 182. doi: 10.1111/imb.12618

Collins, D. H., Mohorianu, I., Beckers, M., Moulton, V., Dalmay, T., & Bourke, A. F. (2017). MicroRNAs associated with caste determination and differentiation in a primitively eusocial insect. *Scientific Reports*, *7*, 45674. doi: 10.1038/srep45674

Conesa, A., Gotz, S., Garcia-Gomez, J. M., Terol, J., Talon, M., & Robles, M. (2005). Blast2GO: a universal tool for annotation, visualization and analysis in functional genomics research. *Bioinformatics*, *21*(18), 3674-3676. doi: 10.1093/bioinformatics/bti610

Conesa, A., Madrigal, P., Tarazona, S., Gomez-Cabrero, D., Cervera, A., McPherson, A., . . . Mortazavi. A. (2016). A survey of best practices for RNA-seq data analysis. *Genome Biology*, 13, 13. doi: 10.1186/s13059-016-0881-8

Duchateau, M. J., & Velthuis, H. H. W. (1988). Development and reproductive strategies in *Bombus terrestris* colonies. Behaviour, 107(3-4), 186-207. doi: 10.1163/156853988x00340

Eden, E., Navon, R., Steinfeld, I., Lipson, D., & Yakhini, Z. (2009). GOrilla: a tool for discovery and visualization of enriched GO terms in ranked gene lists. *BMC Bioinformatics*, *10*(1), 48. doi: 10.1186/1471-2105-10-48

Elsik, C. G., Worley, K. C., Bennett, A. K., Beye, M., Camara, F., Childers, C. P., . . . Consor, H. B. G. S. (2014). Finding the missing honey bee genes: lessons learned from a genome upgrade. *BMC Genomics*, *15*, 86. doi: 10.1186/1471-2164-15-86

Ewels, P., Magnusson, M., Lundin, S., & Käller, M. (2016). MultiQC: summarize analysis results for multiple tools and samples in a single report. *Bioinformatics*, *32* (19), 3047–3048. doi: 10.1093/bioinformatics/btw354

Haydak, M. H. (1970). Honey bee nutrition. *Annual Review of Entomology*, *15*, 143-156.

He, X. J., Jiang, W. J., Zhou, M., Barron, A. B., & Zeng, Z. J. (2017). A comparison of honeybee (*Apis mellifera*) queen, worker and drone larvae by RNA-Seq. *Insect Science*, *26*, 499-509.

doi:10.1111/1744-7917.12557

Hellemans, J., Mortier, G., De Paepe, A., Speleman, F., & Vandesompele, J. (2007). qBase relative quantification framework and software for management and automated analysis of real-time quantitative PCR data*. Genome Biology*, *8*(2), R19.

Hoskins, R. A., Carlson, J. W., Wan, K. H., Park, S., Mendez, I., Galle, S. E., . . . Celniker, S. E. (2015). The Release 6 reference sequence of the *Drosophila melanogaster* genome. *Genome Research*, *25*(3), 445-458. doi: 10.1101/gr.185579.114

Kim, D., Langmead, B., & Salzberg, S. L. (2015). HISAT: a fast spliced aligner with low memory requirements. *Nature Methods*, 12, 357 – 360. doi: 10.1038/nmeth.3317

Love, M.I., Huber, W. & Anders, S. (2014). Moderated estimation of fold change and dispersion for RNA-seq data with DESeq2. *Genome Biology*, *15*, 550. doi: 10.1186/s13059-014-0550-8

Merchant, N., Lyons, E., Goff, S., Vaughn, M., Ware, D., Micklos, D., & Antin, P. (2016). The iPlant collaborative: Cyberinfrastructure for enabling data to discovery for the life sciences. *PLOS Biology*, *14*(1), e1002342. doi:10.1371/journal.pbio.1002342

Mohorianu, I., Bretman, A., Smith, D. T., Fowler, E. K., Dalmay, T., & Chapman, T. (2017a). Comparison of alternative approaches for analysing multi-level RNA-seq data. *PLoS ONE*, *12*(8). e0182694. doi: 10.1371/journal.pone.0182694

Mohorianu, I., Bretman, A., Smith, D. T., Fowler, E. K., Dalmay, T., & Chapman, T. (2017b). Genomic responses to the socio-sexual environment in male *Drosophila melanogaster* exposed to conspecific rivals. *RNA*, *23*(7), 1048-1059. doi: 10.1261/rna.059246.116.

Mohorianu, I., Schwach, F., Jing, R. C., Lopez-Gomollon, S., Moxon, S., Szittya, G., . . . Dalmay, T. (2011). Profiling of short RNAs during fleshy fruit development reveals stage-specific sRNAome expression patterns. *Plant Journal*, *67*(2), 232-246. doi: 10.1111/j.1365-313X.2011.04586.x

Pereboom, J. J. M., Jordan, W. C., Sumner, S., Hammond, R. L., & Bourke, A. F. G. (2005). Differential gene expression in queen-worker caste determination in bumble-bees. *Proceedings of the Royal Society B-Biological Sciences*, *272*(1568), 1145-1152. doi: 10.1098/rspb.2005.3060

Pfaffl, M. W. (2001). A new mathematical model for relative quantification in real-time RT-PCR. *Nucleic Acids Research*, *29*(9), e45. doi: 10.1093/nar/29.9.e45

Pfaffl MW (2004) Quantification strategies in real‐time PCR. In S. A. Bustin (Ed.), *A‐Z of Quantitative PCR* (pp. 87-112). La Jolla, CA: International University Line.

Prufer, K., Stenzel, U., Dannemann, M., Green, R. E., Lachmann, M., & Kelso, J. (2008). PatMaN: rapid alignment of short sequences to large databases. *Bioinformatics*, *24*(13), 1530-1531. doi: 10.1093/bioinformatics/btn223.

R Core Team (2020). *R: A language and environment for statistical computing*. R Foundation for Statistical Computing, Vienna, Austria. URL https://www.R-project.org/.

Ribeiro, M. F. (1994). Growth in bumble bee larvae - relation between development time, mass, and amount of pollen ingested. *Canadian Journal of Zoology*, *72*(11), 1978-1985. doi: 10.1139/z94-270

Sadd, B. M., Barribeau, S. M., Bloch, G., de Graaf, D. C., Dearden, P., Elsik, C. G., . . . Worley, K. C. (2015). The genomes of two key bumblebee species with primitive eusocial organization*. Genome Biology*, *16*(1), 76. doi: 10.1186/s13059-015-0623-3

Sahraeian, S. M. E., Mohiyuddin, M., Sebra, R., Tilgner, H., Afshar, P. T., Au, K. F., . . . Lam, H. Y. K. (2017). Gaining comprehensive biological insight into the transcriptome by performing a broad-spectrum RNA-seq analysis. *Nature* *Communications*, *8*, 59. doi: 10.1038/s41467-017-00050-4

Soneson, C., Love, M.I., & Robinson, M.D. (2016). Differential analyses for RNA-seq: transcript-level estimates improve gene-level inferences. *F1000Research*, *4*, 1521. doi: 10.12688/f1000research.7563.2

Supek, F., Bosnjak, M., Skunca, N., & Smuc, T. (2011). REVIGO summarizes and visualizes long lists of gene ontology terms. *PLoS ONE*, *6*(7) e21800. doi: 10.1371/journal.pone.0021800

Tatusov, R. L., Koonin, E. V., & Lipman, D. J. (1997). A genomic perspective on protein families. *Science*, *278*(5338), 631-637. doi:10.1126/science.278.5338.631

Thornton, B., & Basu, C. (2011). Real-Time PCR (qPCR) primer design using free online software. *Biochemistry and Molecular Biology Education*, *39*(2), 145-154. doi: 10.1002/bmb.20461

Torgerson, W. S. (1952). Multidimensional scaling: I. theory and method. *Psychometrika*, *17*(4), 401-419. doi: 10.1007/BF02288916

Van Rossum, G., & Drake, F. L. (2009). *Python 3 Reference Manual*. Scotts Valley, CA: CreateSpace.

Vandesompele, J., De Preter, K., Pattyn, F., Poppe, B., Van Roy, N., De Paepe, A., & Speleman, F. (2002). Accurate normalization of real-time quantitative RT-PCR data by geometric averaging of multiple internal control genes. *Genome Biology*, *3*(7), research0034.1 doi: 10.1186/gb-2002-3-7-research0034

Wang, L., Park, H. J., Dasari, S., Wang, S. Q., Kocher, J. P., & Li, W. (2013). CPAT: Coding-Potential Assessment Tool using an alignment-free logistic regression model. *Nucleic Acids Research*, *41*(6) e74. doi: 10.1093/nar/gkt006

Weaver, D. B., Anzola, J. M., Evans, J. D., Reid, J. G., Reese, J. T., Childs, K. L., . . . Elsik, C. G. (2007). Computational and transcriptional evidence for microRNAs in the honey bee genome. *Genome Biology*, *8*(6), R97. doi:10.1186/gb-2007-8-6-r97

Wheeler, D. E., Buck, N. A., & Evans, J. D. (2014). Expression of insulin/insulin-like signalling and TOR pathway genes in honey bee caste determination. *Insect Molecular Biology*, *23*(1), 113-121. doi: 10.1111/imb.12065

Winnebeck, E. C., Millar, C. D., & Warman, G. R. (2010). Why does insect RNA look degraded? *Journal of Insect Science*, *10*, 159. doi: 10.1673/031.010.14119

Winston, M. L. (1987). *The biology of the honey bee*. Cambridge, MA: Harvard University Press.

# Supplemental Figures

## Figure S1


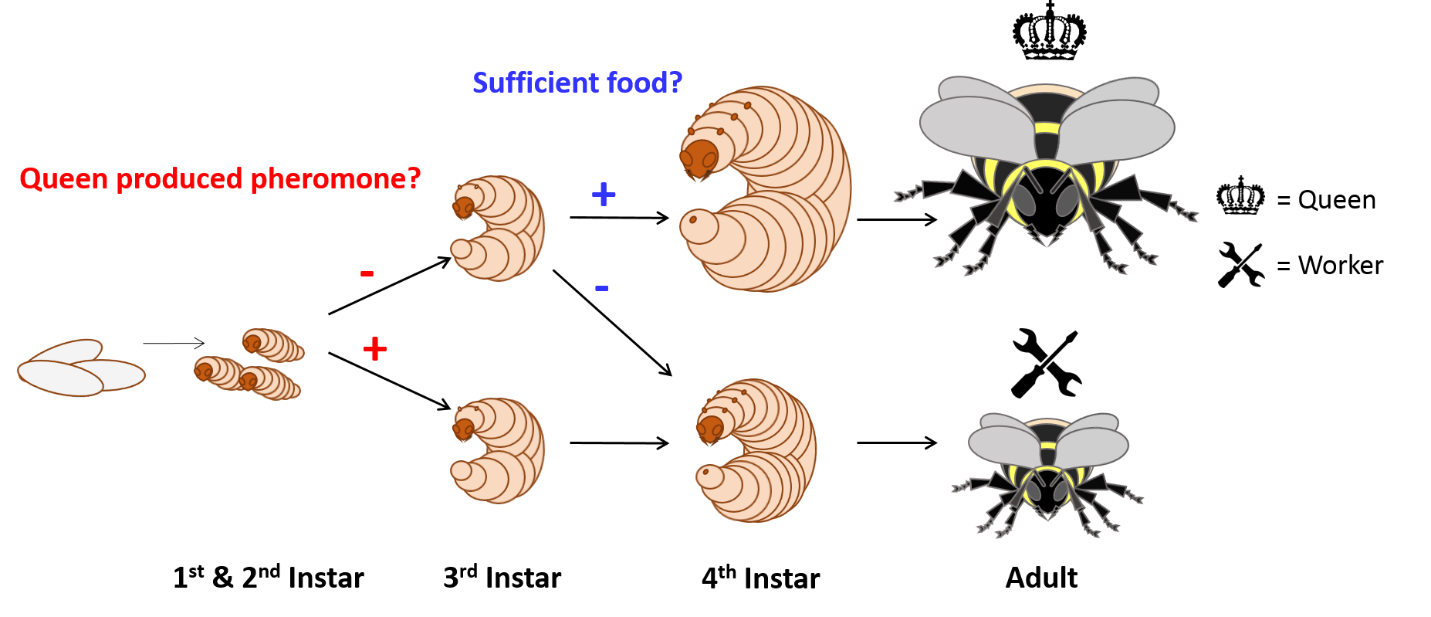


Putative model for caste determination of larvae of *Bombus terrestris* (based on references in main text, Introduction). Black arrows: the development between different larval instars; +/- signs: the presence or absence of the first caste determination factor (whether a hypothesised queen-produced pheromone is present during the more strongly supported queen-dependent period) and the second caste determination factor (whether the larva receives sufficient food to become a queen during the less strongly supported nutrition-sensitive period) during the two sensitive periods of caste determination.

## Figure S2


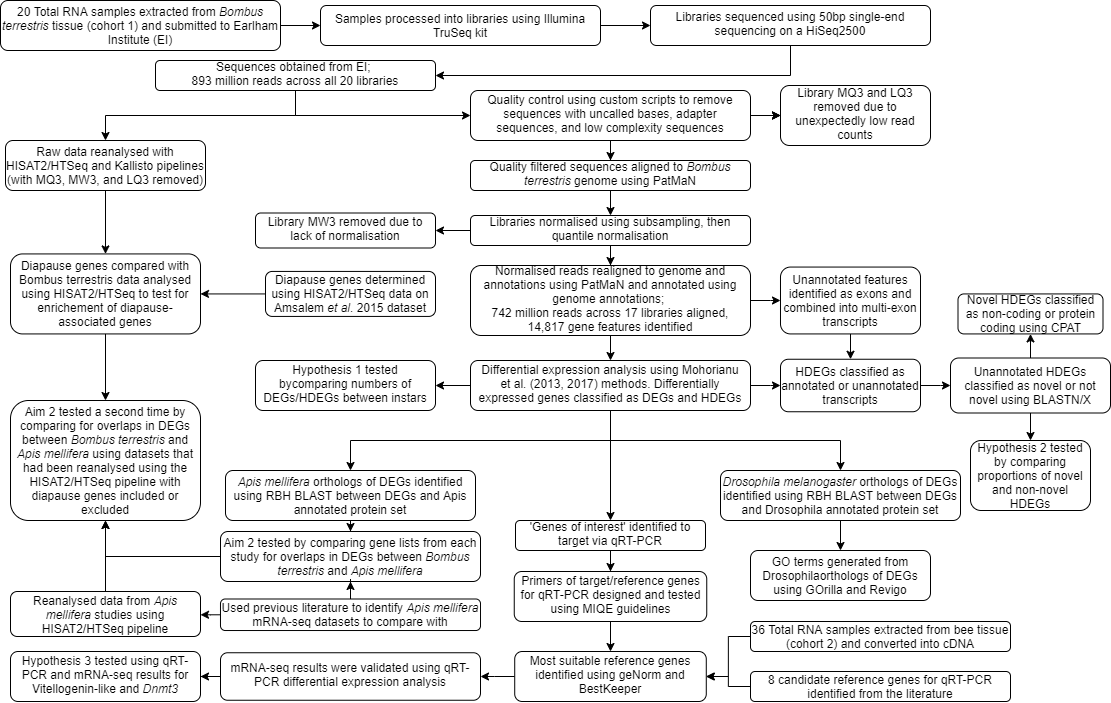


Major steps in the current analysis for testing the two main aims using the mRNA-seq and qRT-PCR data.

## Figure S3


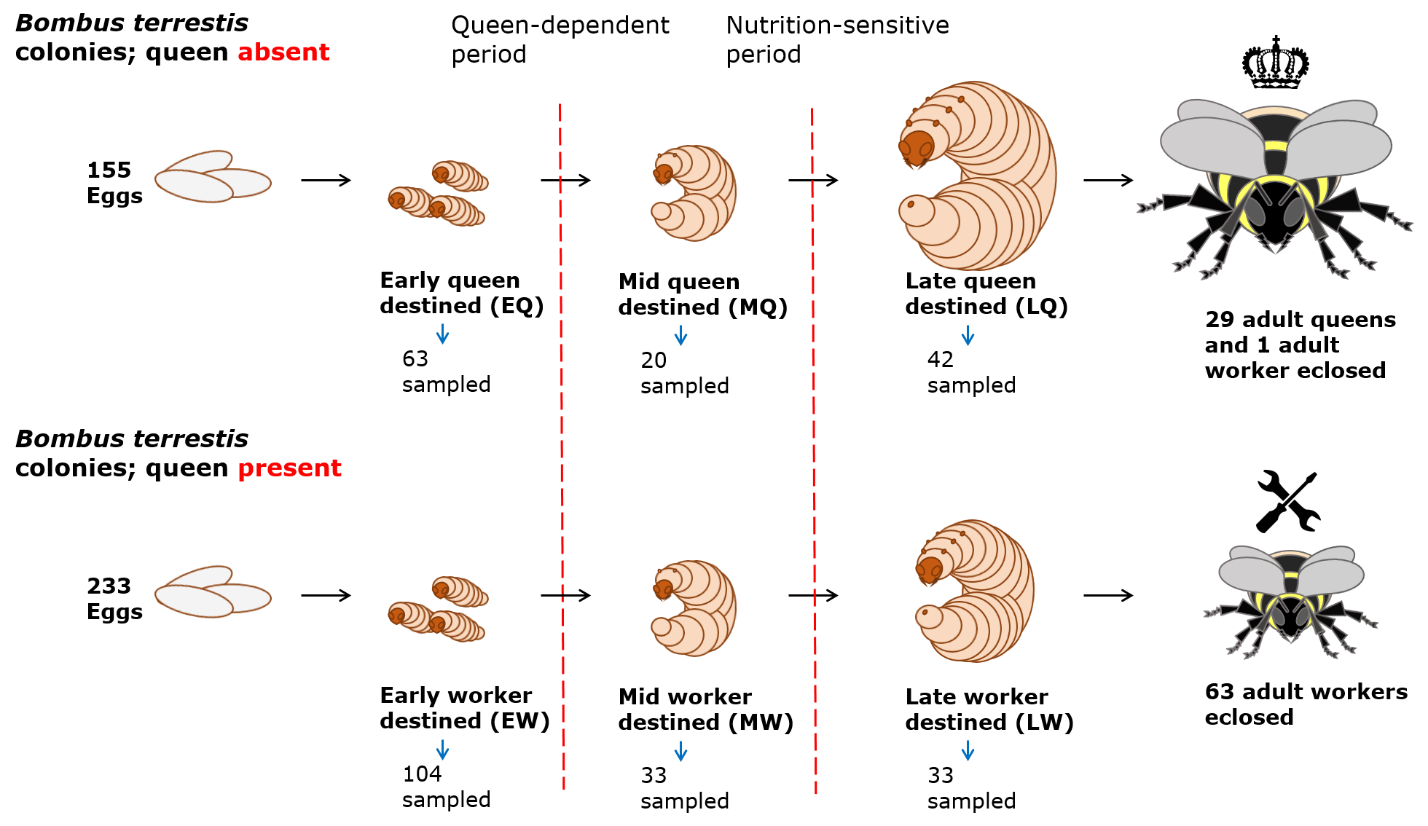


Strategy used to obtain samples of queen- and worker-destined larvae of *Bombus terrestris* for mRNA-seq. Individuals from each treatment (queen absent/present) were sampled from the group of focal larvae at three collection points: 1) early-instar (1^st^/2^nd^ instar) larvae when larvae were totipotent; 2) mid-instar (3^rd^ instar) larvae following the queen-dependent period; and 3) late-instar (4^th^ instar) larvae following the nutrition-sensitive period. Numbers indicate numbers of larvae sampled at each collection stage. Dotted red lines represent the two sensitive periods. The same collection strategy was used for larvae sampled for qRT-PCR (for which numbers of sampled larvae are shown in Table S1).

## Figure S4


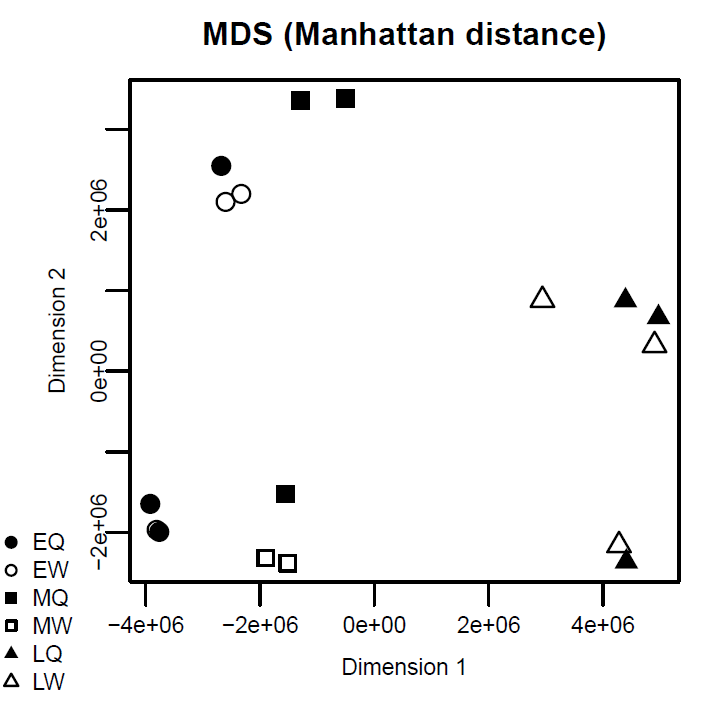


Multidimensional scaling (MDS) plots based on Manhattan distances for all expressed genes isolated from mRNA-seq libraries from pooled whole-body preparations of female larvae of *Bombus terrestris* after library MW1 was excluded from further analysis. Individual points of the same shape and colour indicate biological replicates (sets of pooled whole larvae). Axes represent principal components. EQ = early-instar queen-destined larvae (n = 3); EW = early-instar worker-destined larvae (n = 3); MQ = mid-instar queen-destined larvae (n = 3); MW = mid-instar worker-destined larvae (n = 2); LQ = late-instar queen-destined larvae (n = 3); LW = late-instar worker-destined larvae (n = 3).

## Figure S5

**
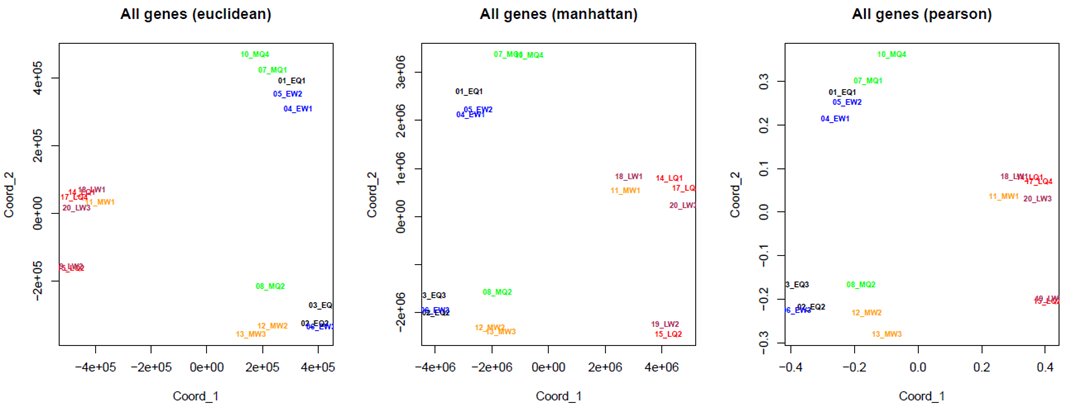
**

Multidimensional scaling (MDS) plots based on Euclidean, Manhattan and Pearson distances for all expressed genes isolated from mRNA-seq libraries from pooled whole-body preparations of female larvae of *Bombus terrestris* before library MW1 was excluded from further analysis. Individual character strings of the same colour indicate biological replicates (sets of pooled whole larvae). Black characters, early-instar queen-destined larvae (EQ, n = 3); blue characters, early-instar worker-destined larvae (EW, n = 3); green characters, mid-instar queen-destined larvae (MQ, n = 3); yellow characters, mid-instar worker-destined larvae (MW, n = 3); red characters, late-instar queen-destined larvae (LQ, n = 3); purple characters, late-instar worker-destined larvae (LW, n = 3). Axes represent principal components.

## Figure S6

**
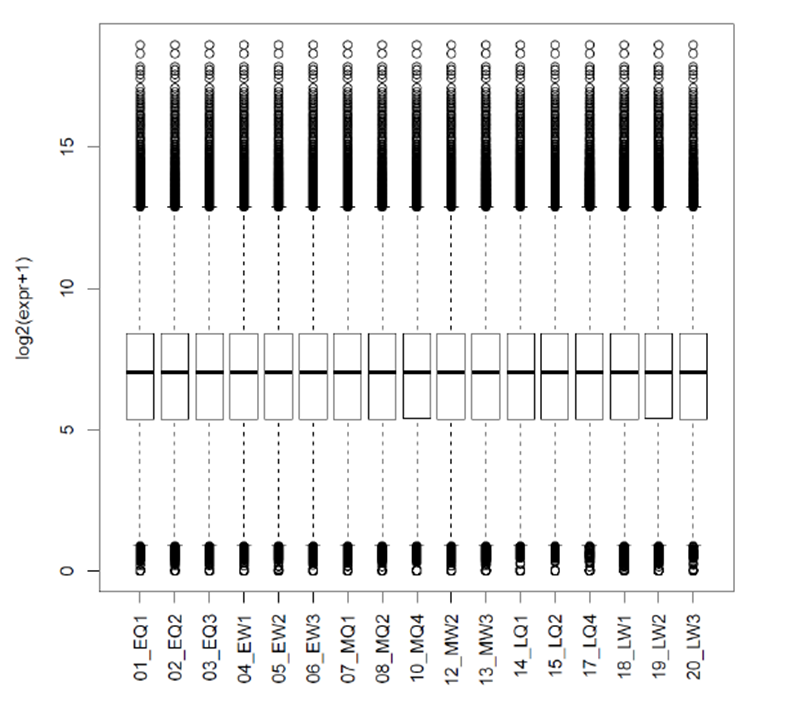
**

Distribution of differences (log_2_ offset fold changes) between pairs of biological replicates within each phenotype class in 17 mRNA-seq libraries prepared from *Bombus terrestris* female larvae. Normalisation boxplots showing the median, interquartile range, 10th and 90th percentile of the log_2_ value of the expression value for genes in *Bombus terrestris* larvae following mRNA-seq. EQ = early-instar queen-destined larvae (n = 3); EW = early-instar worker-destined larvae (n = 3); MQ = mid-instar queen-destined larvae (n = 3); MW = mid-instar worker-destined larvae (n = 2); LQ = late-instar queen-destined larvae (n = 3); LW = late-instar worker-destined larvae (n = 3).

## Figure S7 - See accompanying file ‘Figure S7’ for full figure.

Normalisation scatterplots between pairs of biological replicates in six phenotype classes from 17 mRNA-seq libraries prepared from *Bombus terrestris* female larvae. The X axis represents the expression value (lOFC) for each gene and Y axis represents the corresponding values for the same genes in another sample. EQ = early-instar queen-destined larvae (n = 3); EW = early-instar worker-destined larvae (n = 3); MQ = mid-instar queen-destined larvae (n = 3); MW = mid-instar worker-destined larvae (n = 2); LQ = late-instar queen-destined larvae (n = 3); LW = late-instar worker-destined larvae (n = 3).

## Figure S8 - See accompanying file ‘Figure S8’ for full figure.

Normalisation MA-plots between pairs of biological replicates in six phenotype classes from 17 mRNA-seq libraries prepared from *Bombus terrestris* female larvae. The X axis represents the log_2_ value of the average expression of each gene in two samples and the Y axis represents the log_2_ value of the foldchange of expression between the two samples (i.e. log_2_ of the ratio between expression of one sample and the other sample). EQ = early-instar queen-destined larvae (n = 3); EW = early-instar worker-destined larvae (n = 3); MQ = mid-instar queen-destined larvae (n = 3); MW = mid-instar worker-destined larvae (n = 2); LQ = late-instar queen-destined larvae (n = 3); LW = late-instar worker-destined larvae (n = 3).

## Figure S9


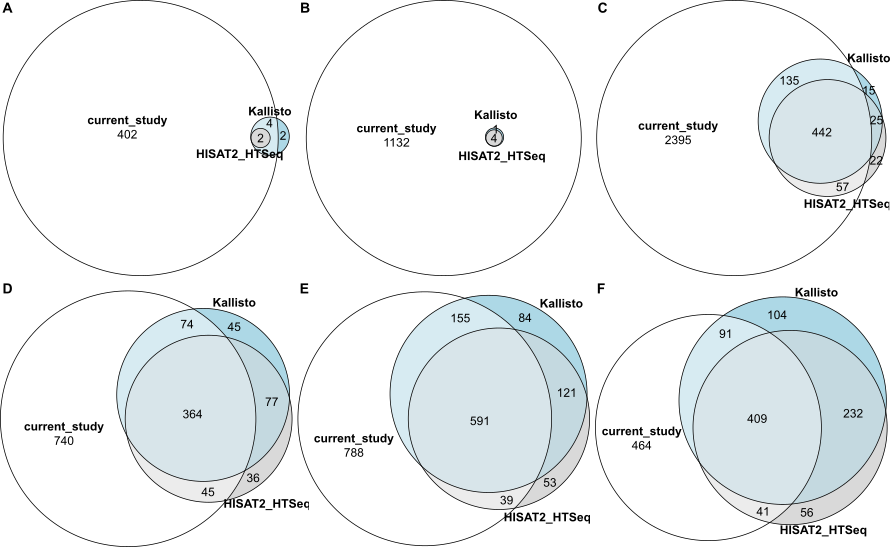


Euler diagrams of significant overlaps between caste-associated differentially expressed genes (DEGs) isolated from female larvae of *Bombus terrestris* by mRNA-seq in six phenotypes using the pipeline described in the current study and two alternative pipelines (Kallisto and HISAT2). Overlap between DEGs upregulated in: A) EQ (early-instar queen-destined larvae; n = 3); B) EW (early-instar worker-destined larvae; n = 3); C) MQ (mid-instar queen-destined larvae; n = 3); D) MW (mid-instar worker-destined larvae; n = 2); E) LQ (late-instar queen-destined larvae; n = 3); F) LW (late-instar worker-destined larvae; n = 3); where n = number of mRNA-seq libraries per phenotype. The numbers in the circles/sectors represent the numbers of DEGs predicted by each pipeline alone and the numbers of DEGs predicted by each pipeline also predicted by one or more of the other pipelines. Different coloured circles/sectors represent sets of genes predicted by one, a combination of two, or all three pipelines.

## Figure S10


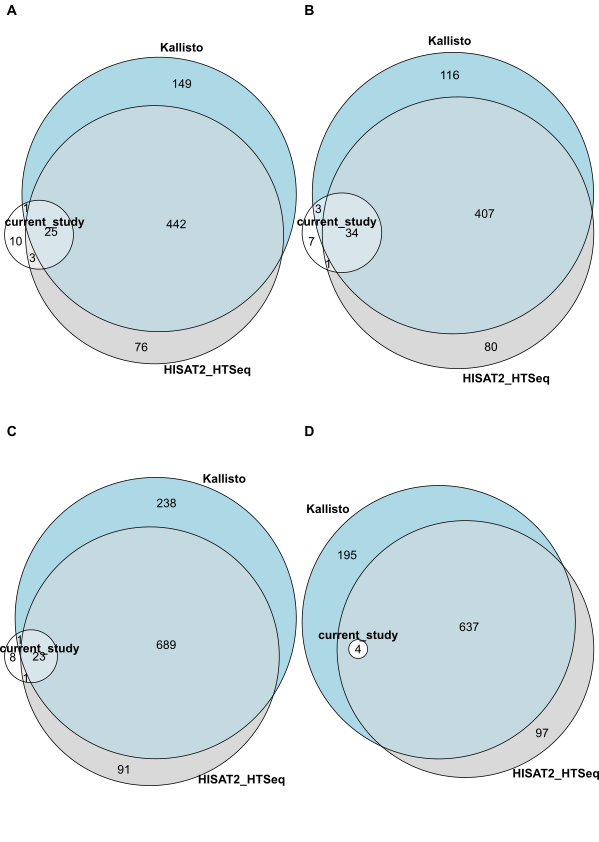


Euler diagrams of significant overlaps between caste-associated highly differentially expressed genes (HDEGs) isolated from female larvae of *Bombus terrestris* by mRNA-seq in six phenotypes using the pipeline described in the current study and two alternative pipelines (Kallisto and HISAT2). Overlap between HDEGs upregulated in A) MQ (mid-instar queen-destined larvae; n = 3); B) MW (mid-instar worker-destined larvae; n = 2); C) LQ (late-instar queen-destined larvae; n = 3); D) LW (late-instar worker-destined larvae; n = 3); where n = number of mRNA-seq libraries per phenotype. The numbers in the circles/sectors represent the numbers of HDEGs (current pipeline) and DEGs (Kallisto and HISAT2) predicted by each pipeline alone and the numbers of DEGs predicted by each pipeline also predicted by one or more of the other pipelines. Different coloured circles/sectors represent sets of genes predicted by one, a combination of two, or all three pipelines.

## Figure S11


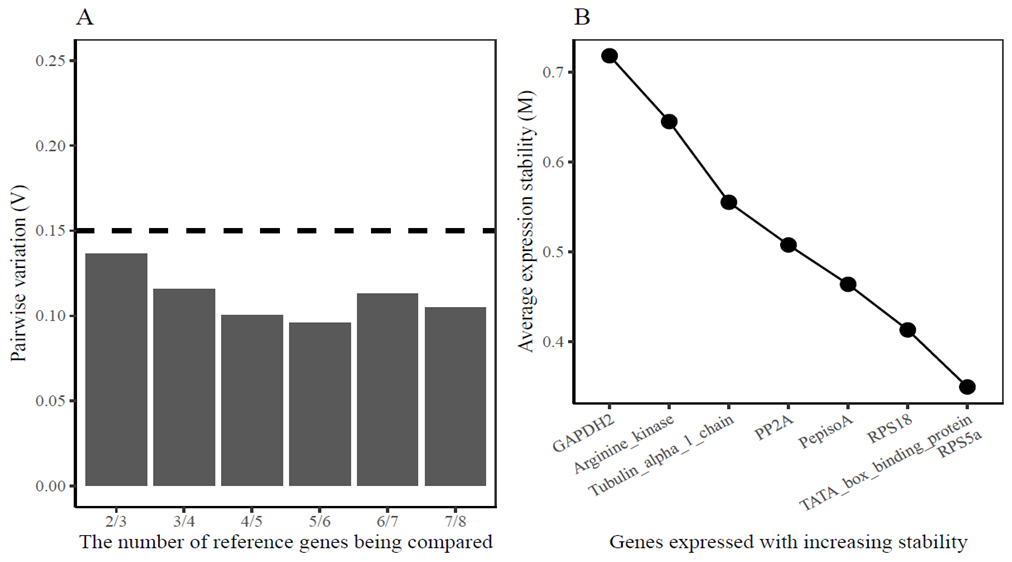


GeNorm results for eight candidate reference genes for qRT-PCR tested for their stability in pooled whole-body preparations of female larvae of *Bombus terrestris*. A) Pairwise variation between candidate reference genes removed stepwise from least stable to most stable to determine the optimal number of reference genes. As the pairwise variation threshold of 0.15 is not crossed, this indicates that two genes were needed as reference genes to ensure stability. B) RPS5a, TATA-binding protein, and RPS18 were the three most stable (lowest M value) candidate reference genes.

## Figure S12


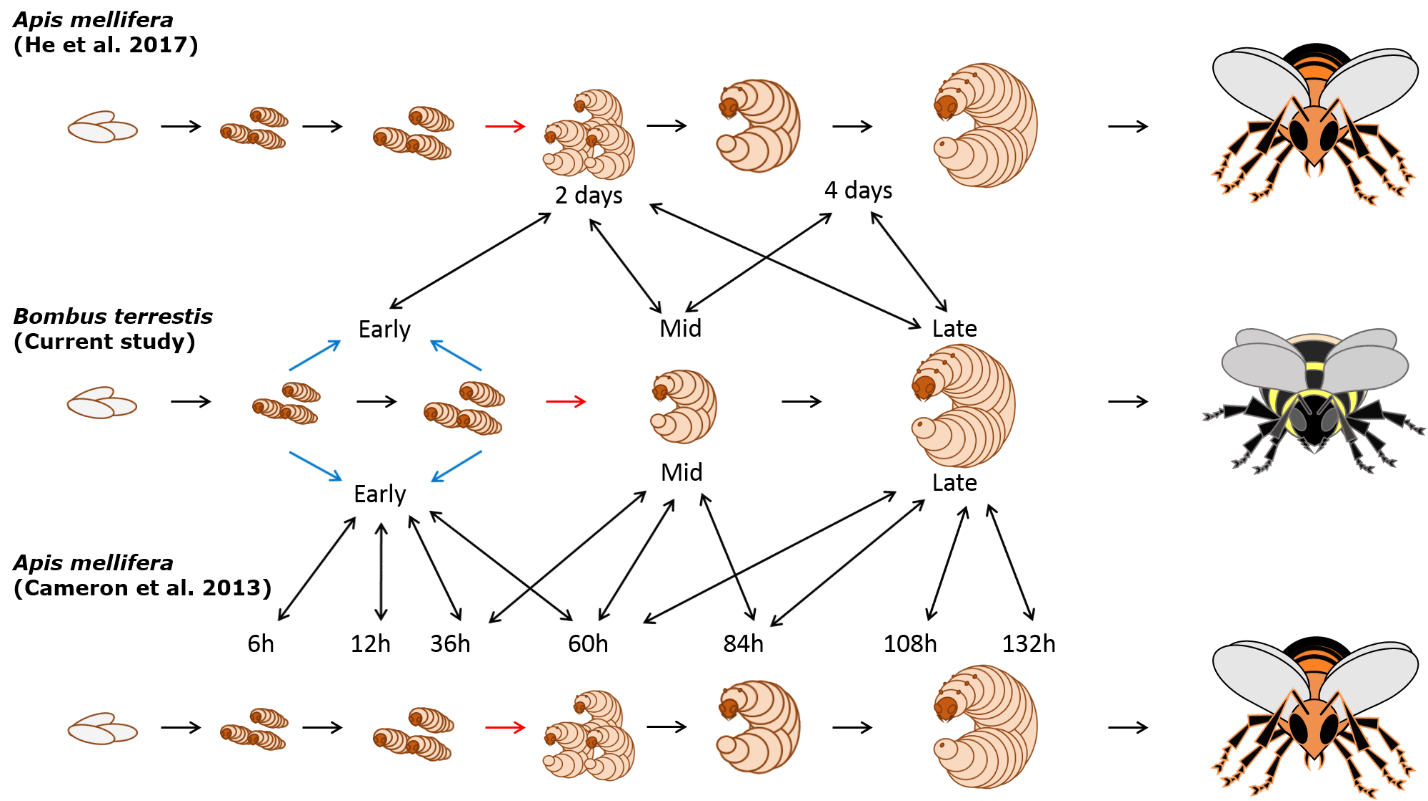


Phenotypes compared in analyses of overlaps between gene lists for queen- vs worker-destined larvae in *Bombus terrestris* in the current study (mRNA-seq data) and *Apis mellifera* in the studies of Cameron et al. (2013) and He et al. (2017). Early, Mid, Late: early-instar (1^st^-2^nd^ instar), mid-instar (3^rd^ instar), and late-instar (4^th^ instar) larvae, respectively, in *B. terrestris* (current study); 2 days, 4 days: larvae that were 2 (3^rd^ instar) and 4 (4^th^ - 5^th^ instar) days old, respectively, in *A. mellifera* (He et al. 2017); 6 h, 12 h etc.: larvae that were 6 (1^st^ instar), 12 (2^nd^ instar), 36 (2^nd^ instar), 60 (3^rd^ instar), 84 (4^th^ instar), 108 (5^th^ instar), or 132 (5^th^ instar) hours old, respectively, in *A. mellifera* (Cameron et al. 2013). Black two-directional arrows: comparisons made in analyses of overlaps of gene lists; black single-directional arrows: progression between separate larval instars; blue arrows: larval instars that were combined into a single developmental stage for RNA extraction; red arrows: developmental stages in each species in which larvae are known to exhibit caste-specific differences in morphology.

## Figure S13


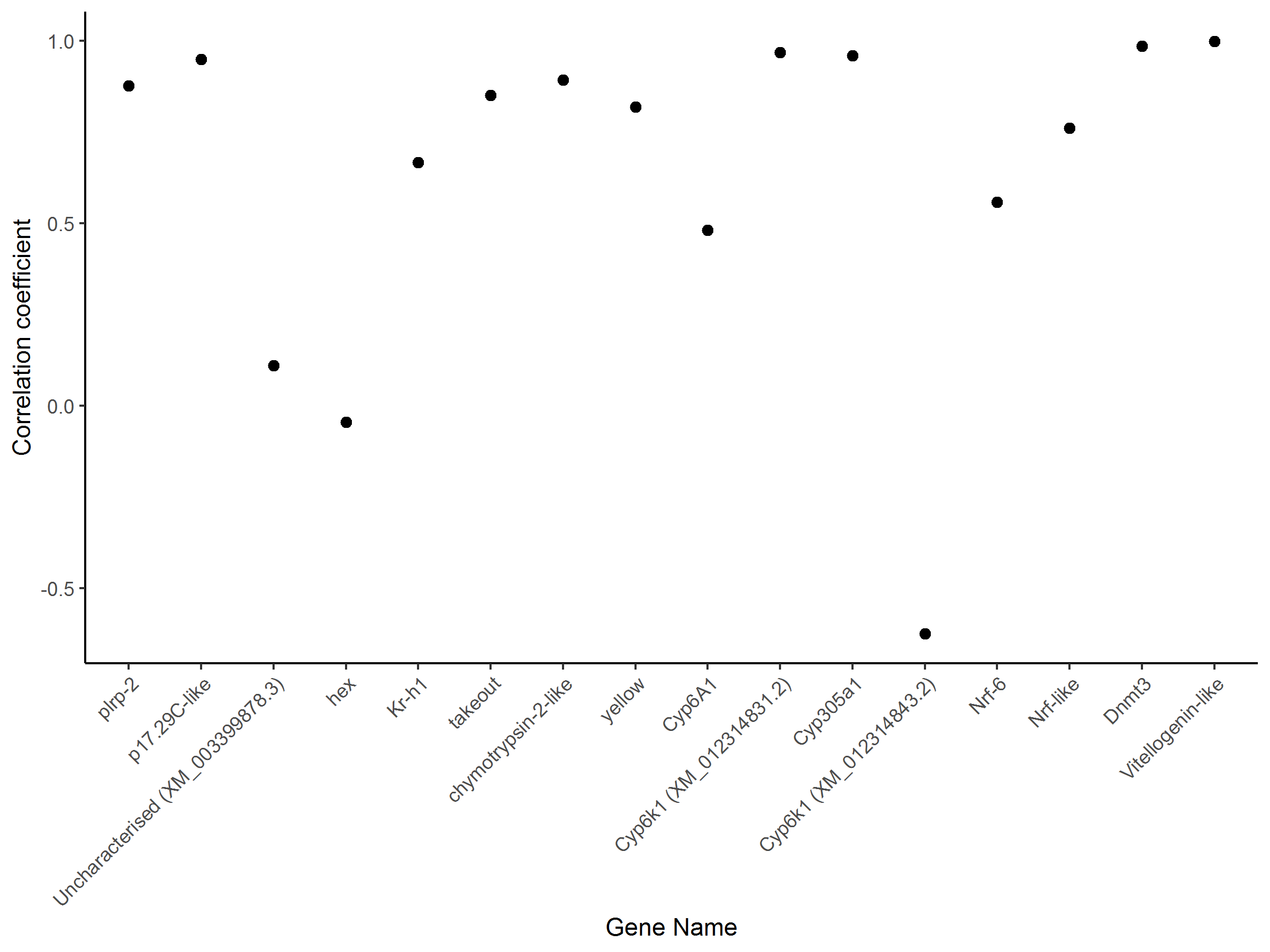


Pearson correlation coefficients between average mRNA-seq read count and average qRT-PCR relative quantification values for six phenotypes for 16 target genes in pooled whole-body preparations of female larvae of *Bombus terrestris*.

## Figure S14


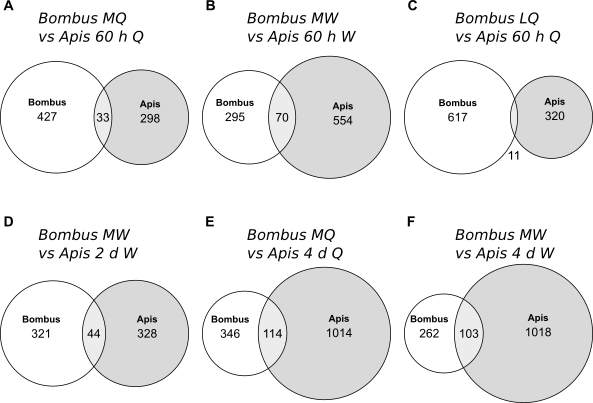


Results of HISAT2/HTSeq comparative analysis (with diapause genes included): Euler diagrams of significant overlaps between caste-associated genes isolated from female larvae of *Bombus terrestris* by mRNA-seq in the current study and caste-associated genes isolated from female larvae from two *Apis mellifera* studies after the mRNA-seq datasets from all three studies were reanalysed using the same bioinformatics pipeline (HISAT2/HTSeq). *A. mellifera* data came from Cameron et al. (2013) (A-C; 3/6 comparisons were significant), and He et al. (2017) (D-F; 2/10 comparisons were significant). Overlap between significantly differentially expressed genes upregulated in: A) MQ in *B. terrestris* with genes upregulated in 60 h-old queen-destined larvae in *A. mellifera*; B) MW in *B. terrestris* with genes upregulated in 60 h-old worker-destined larvae in *A. mellifera*; C) LQ in *B. terrestris* and genes upregulated in 4 day-old queen-destined larvae in *A. mellifera*; D) MQ in *B. terrestris* and genes upregulated in 4 day-old queen-destined larvae in *A. mellifera*; E) MW in *B. terrestris* and genes upregulated in 4 day-old worker-destined larvae in *A. mellifera*. Numbers show numbers of orthologs in each category (numbers shown outside the area of overlap where there is no room for them). Non-significant overlaps are not shown. See Figure S4 legend for phenotype abbreviations.
