# Supplementary figures and images for: Gene expression during larval caste determination and differentiation in intermediately eusocial bumblebees, and a comparative analysis with advanced eusocial honeybees
Source: Mol Ecol. 2021 Jan 7;30(3):718–35. doi: 10.1111/mec.15752 (PMC7898649; doi:10.1111/mec.15752)

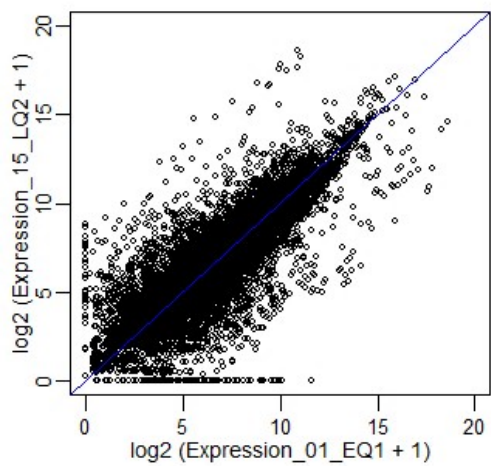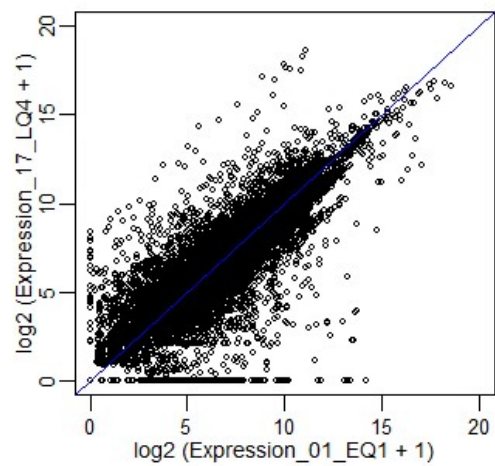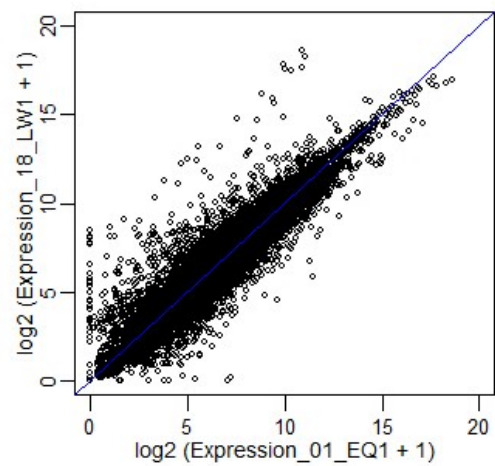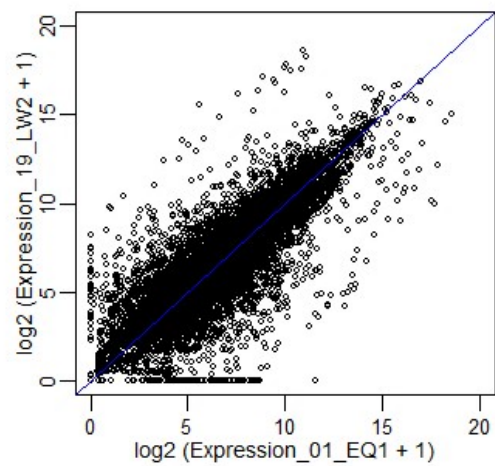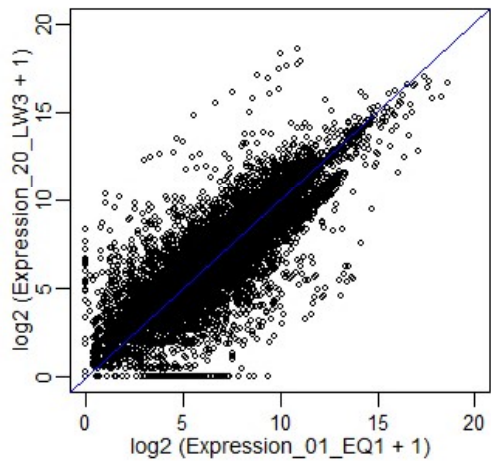

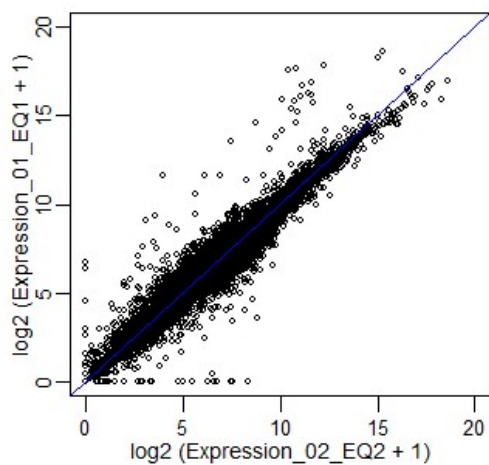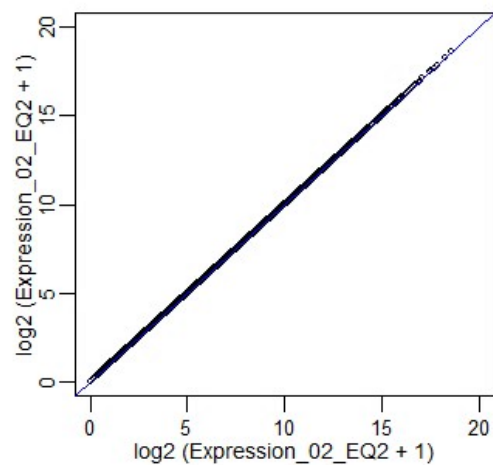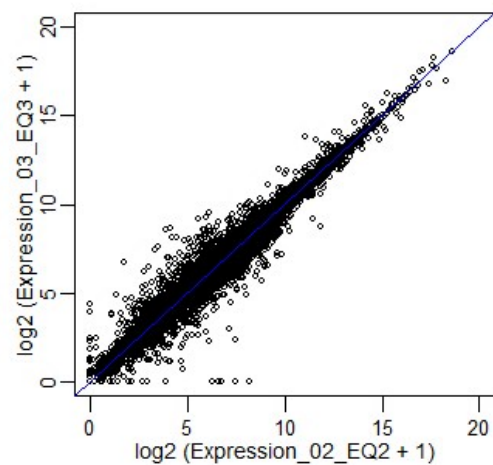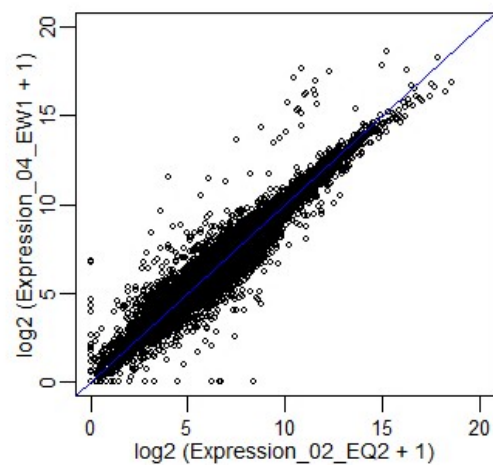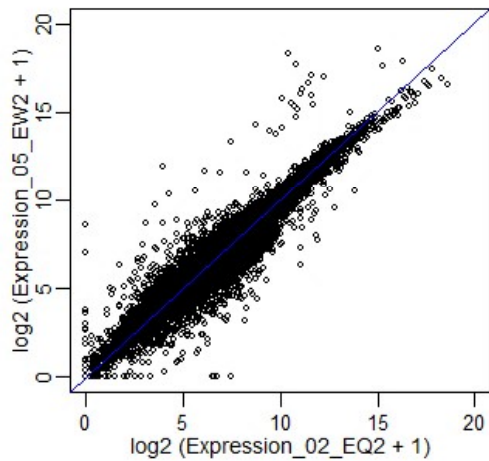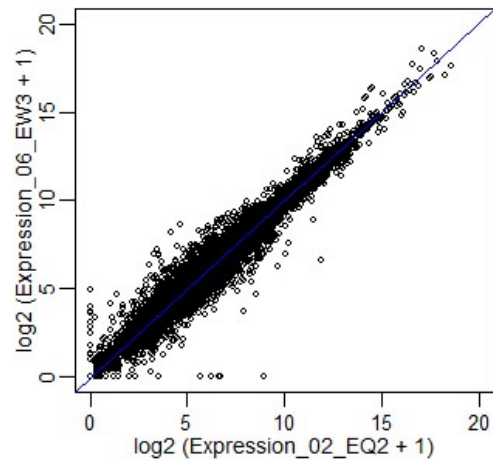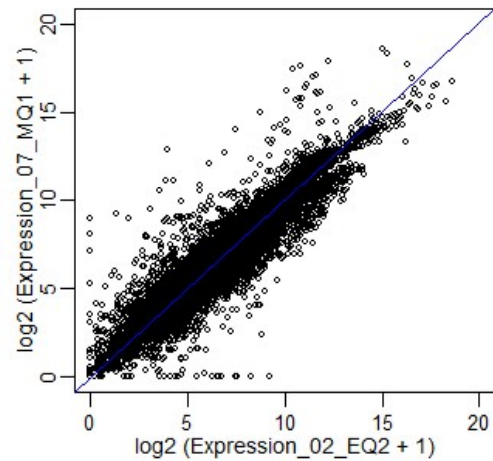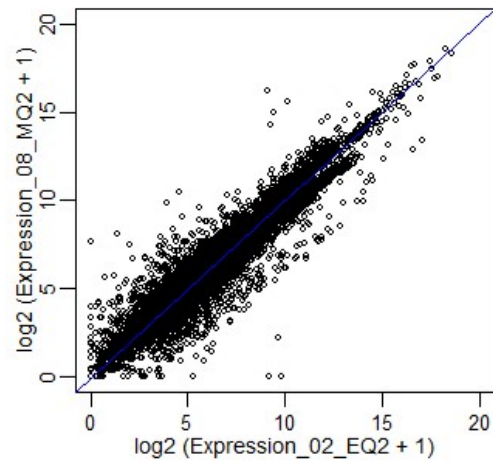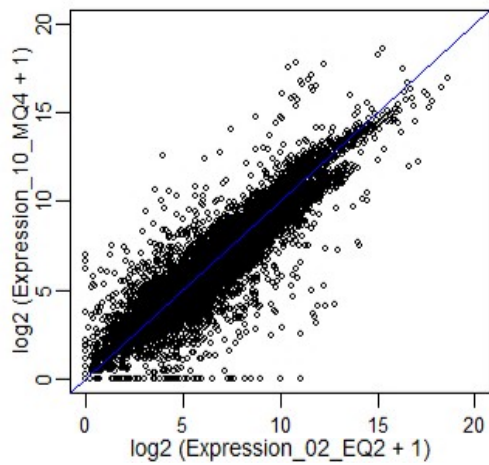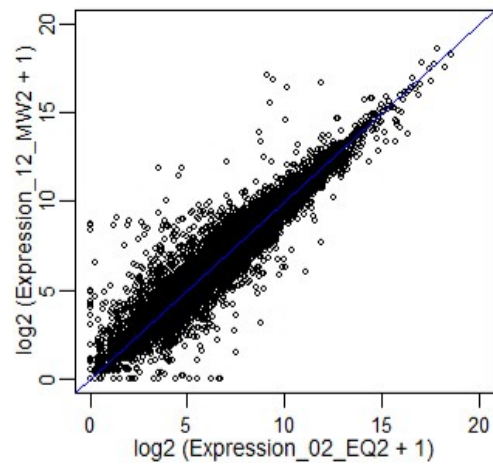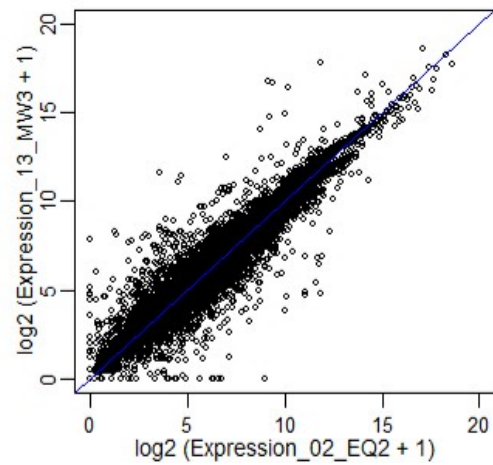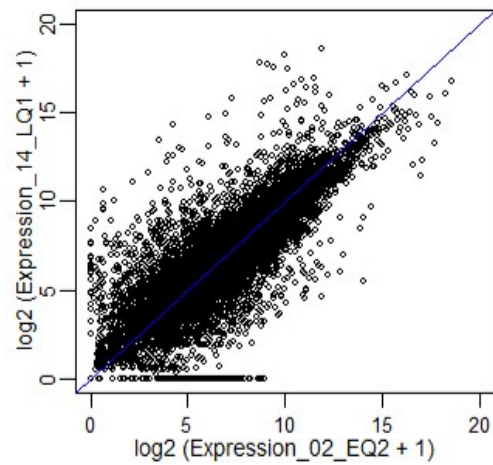

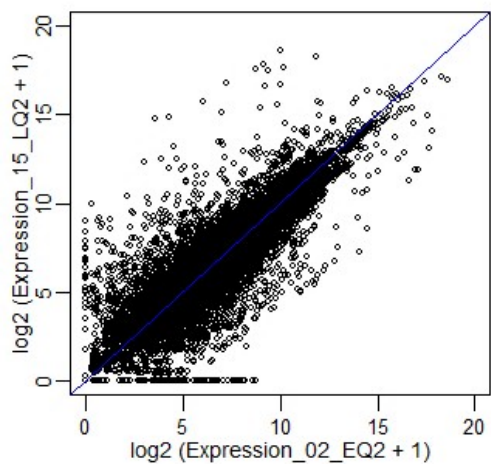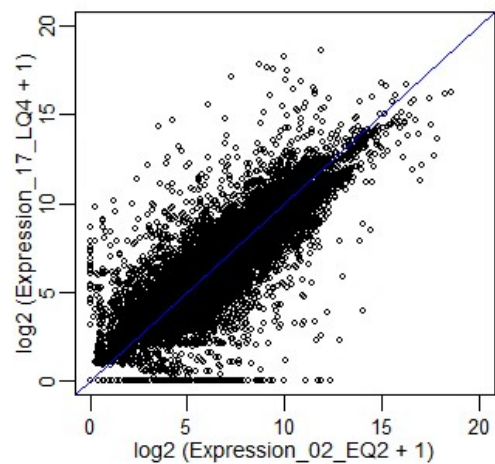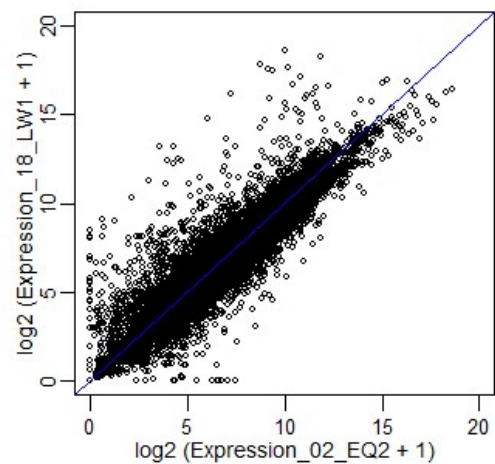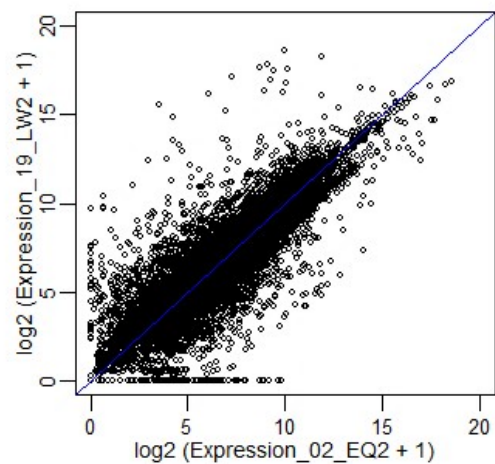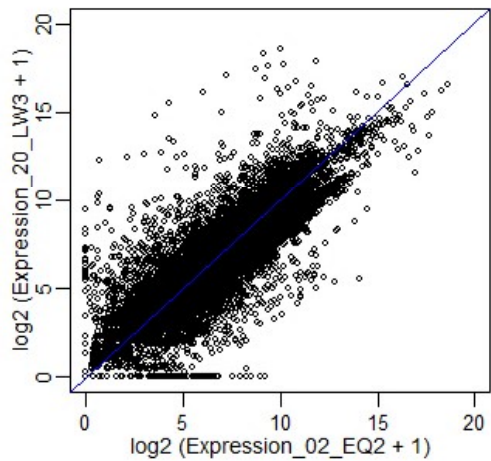

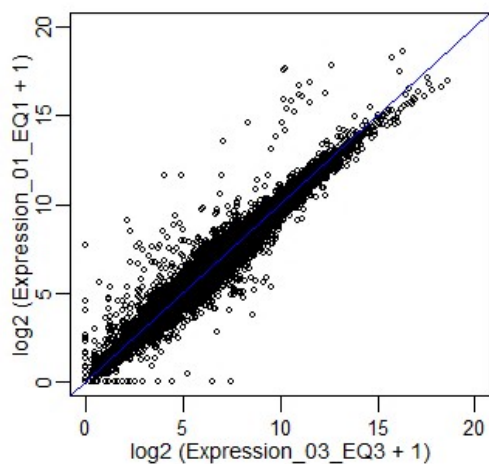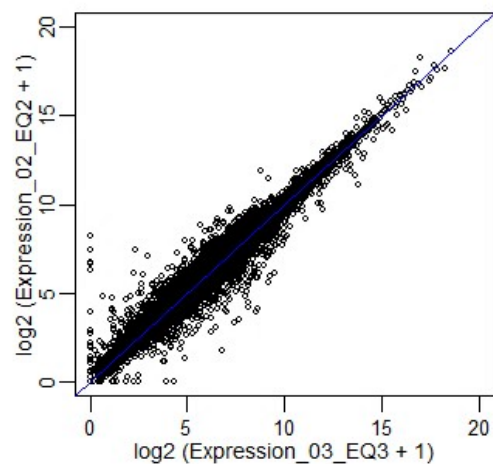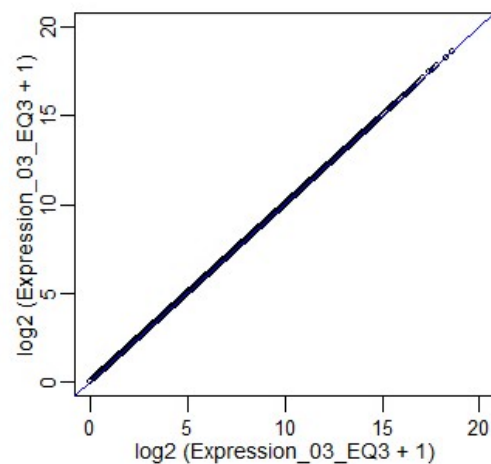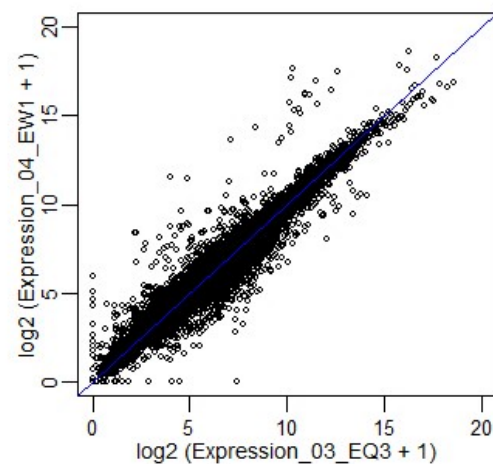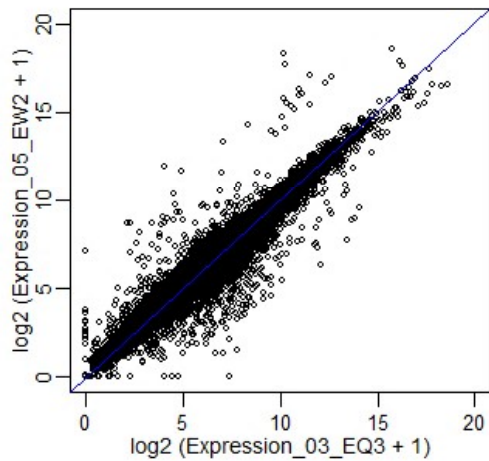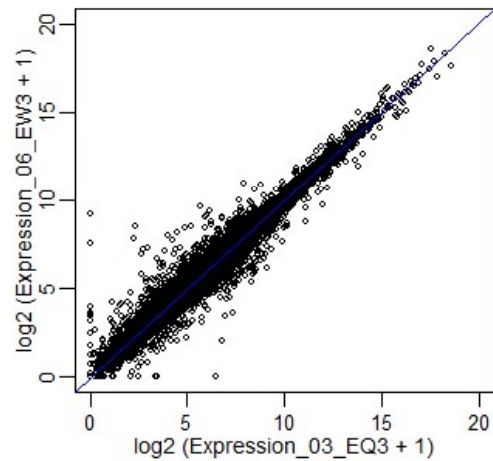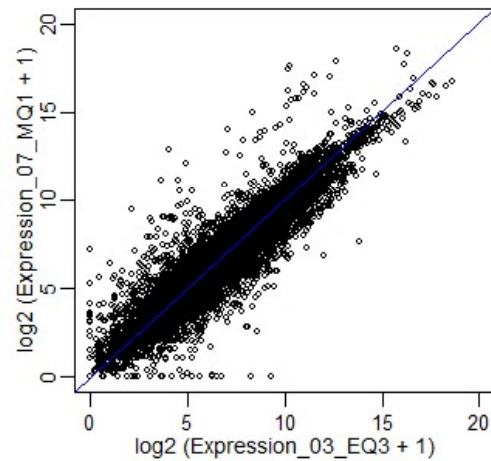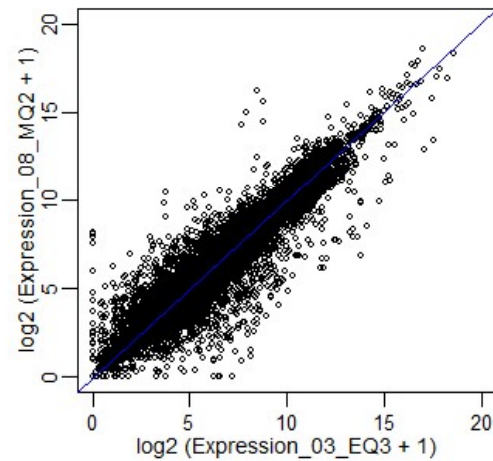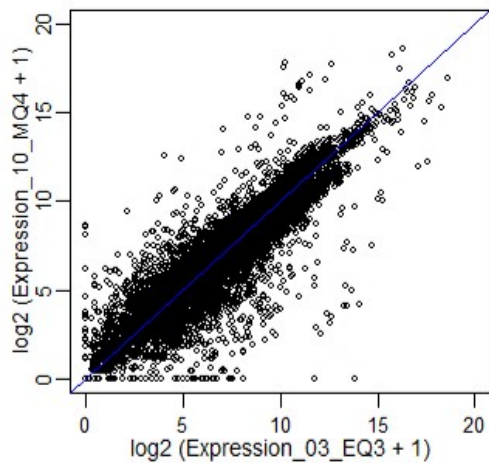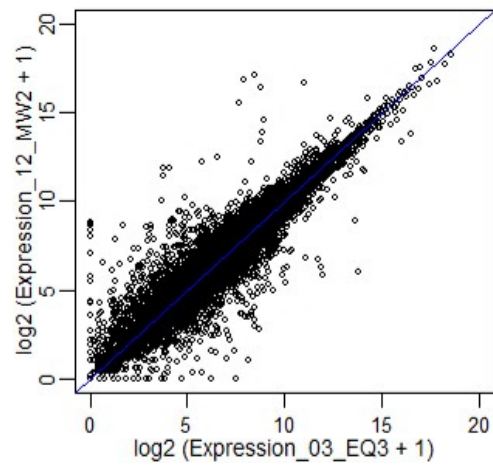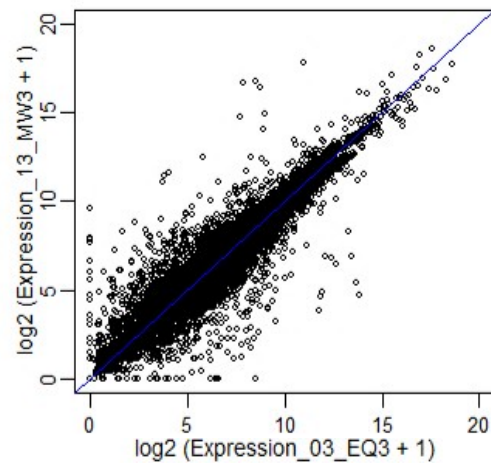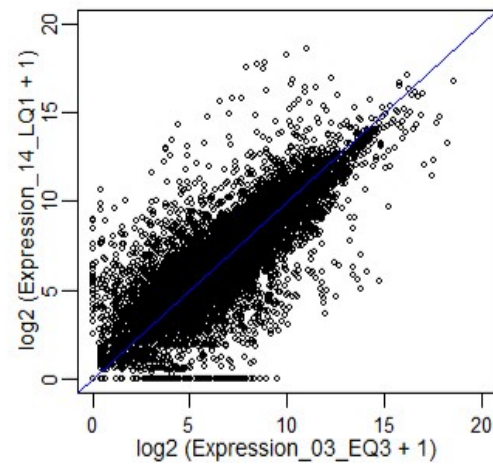

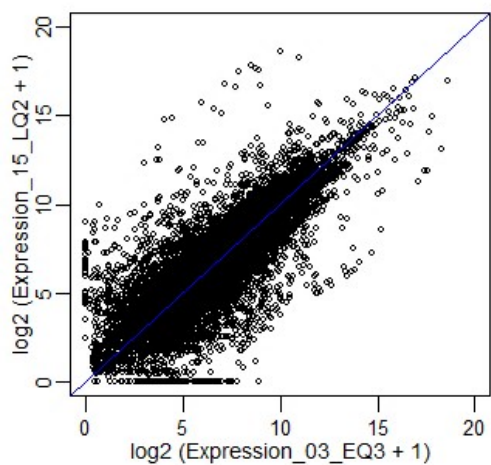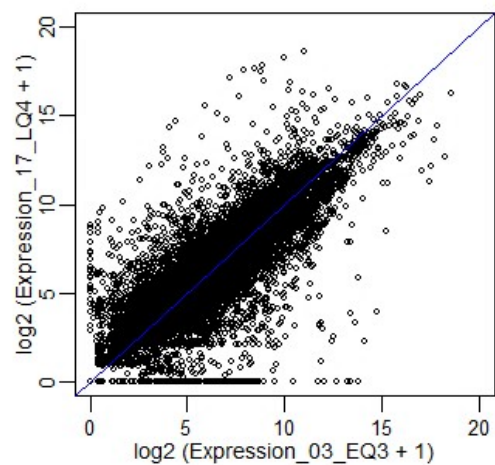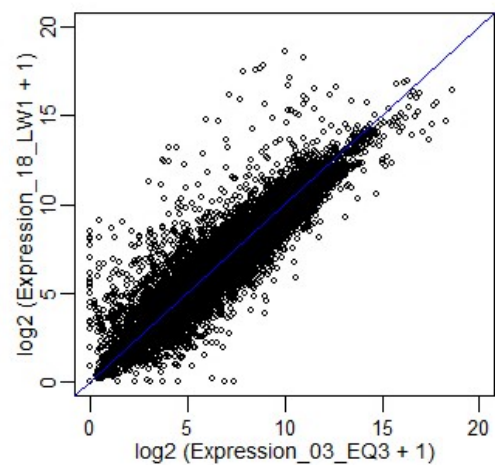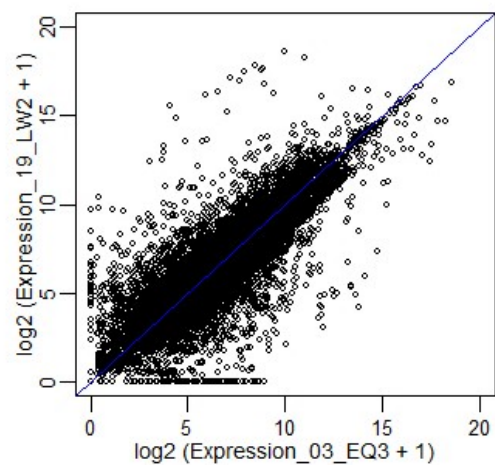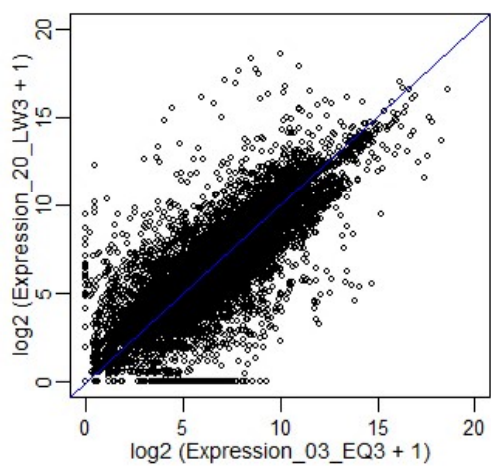

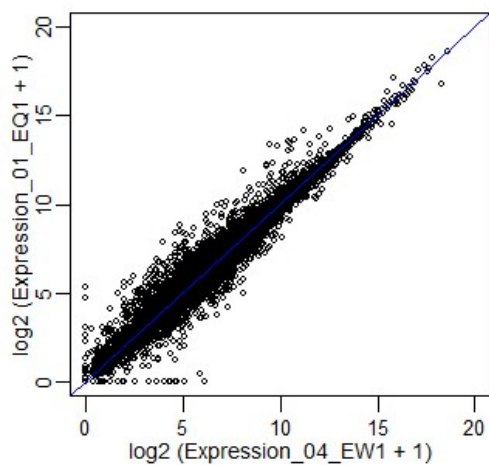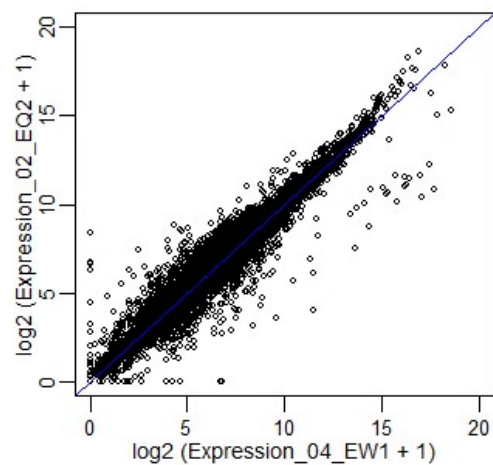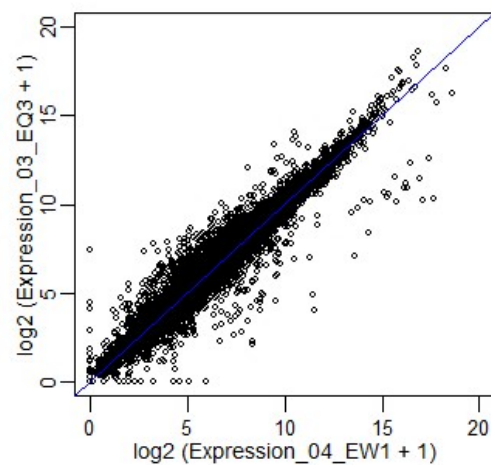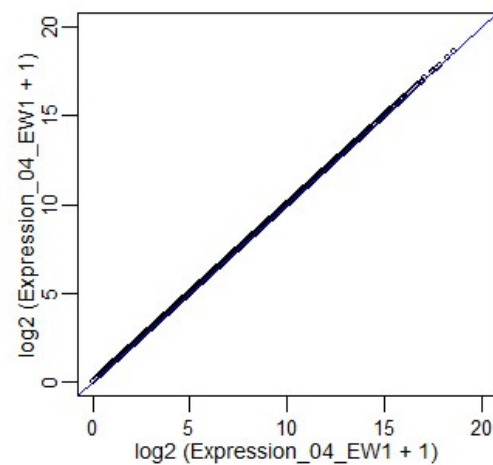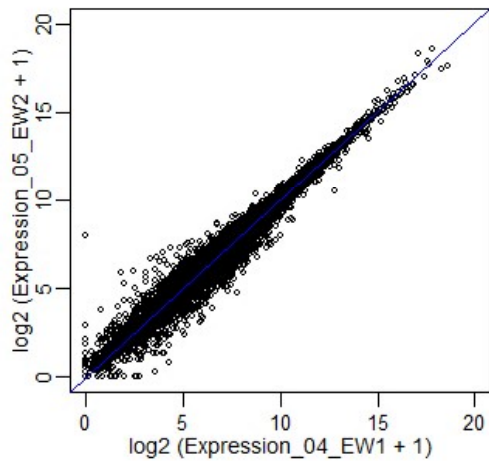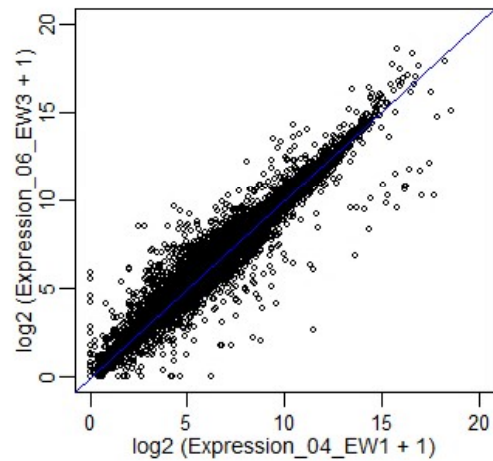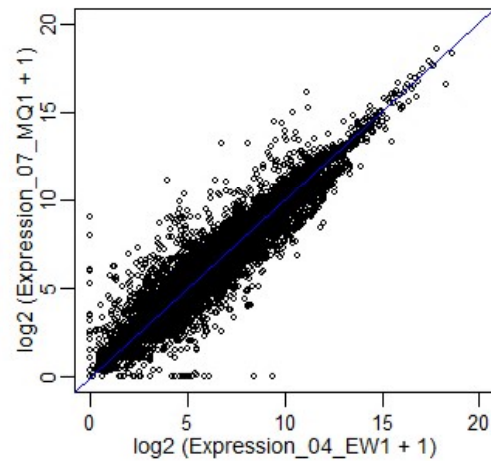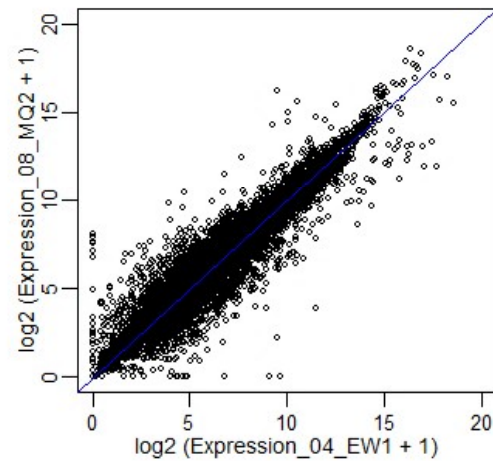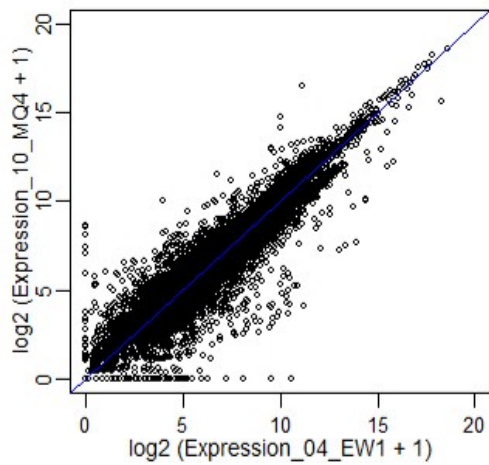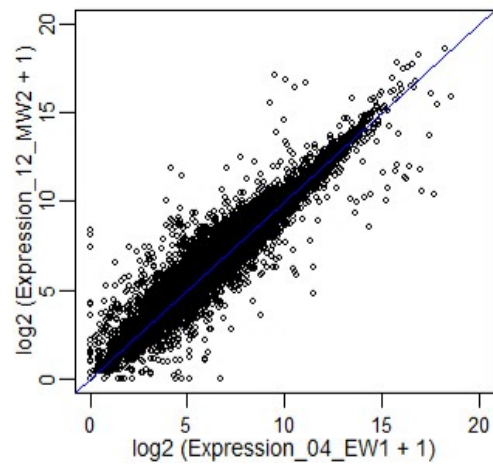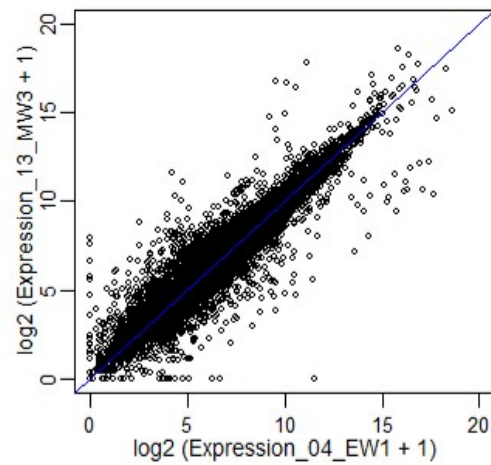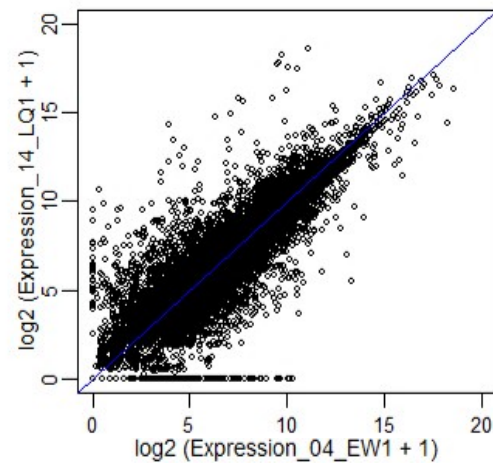

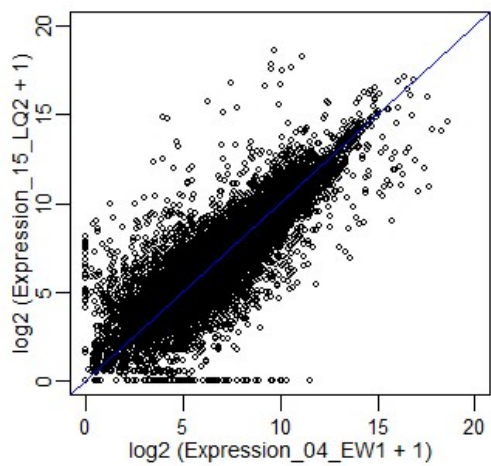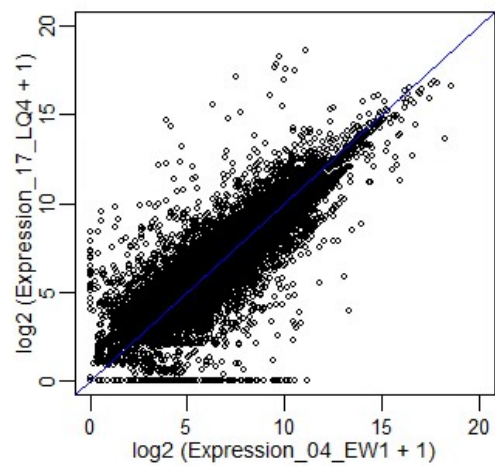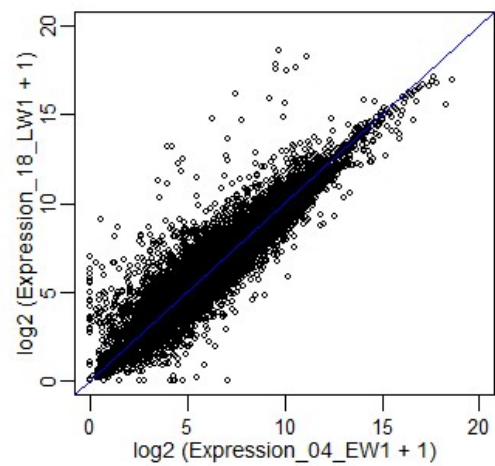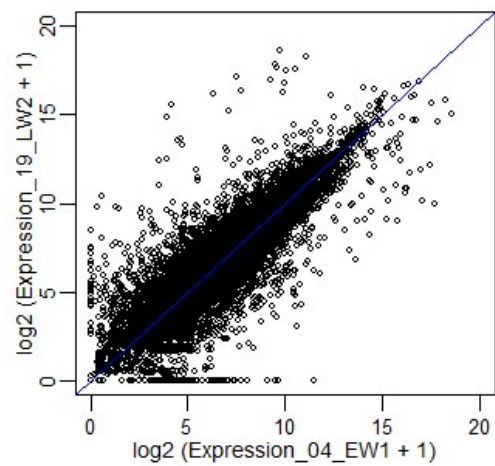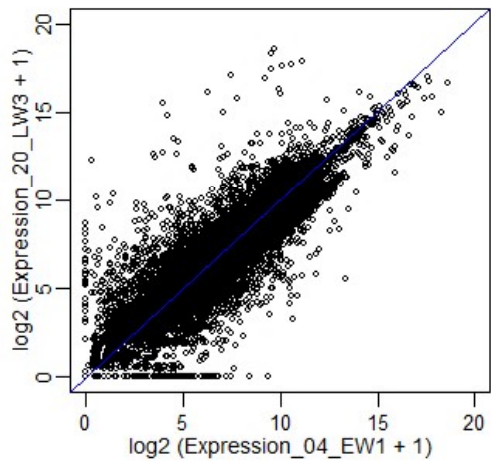

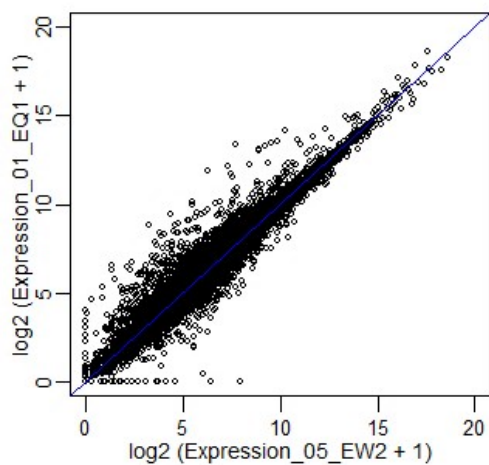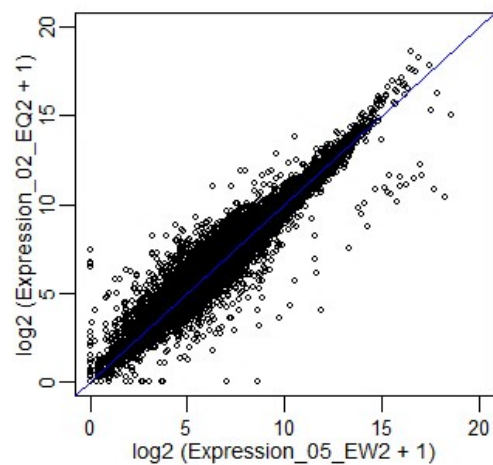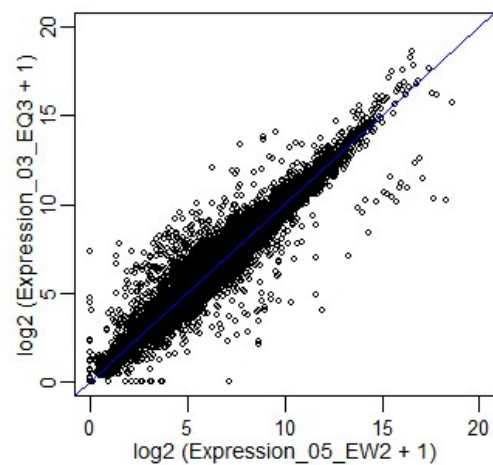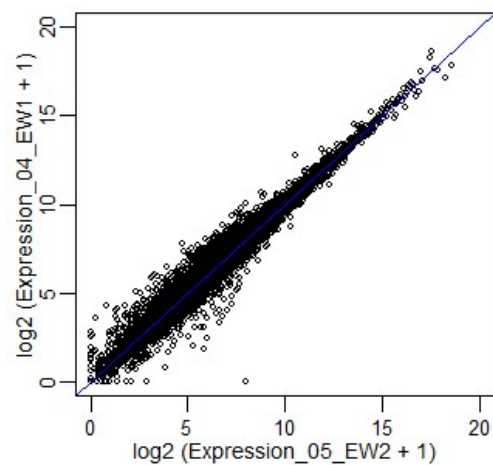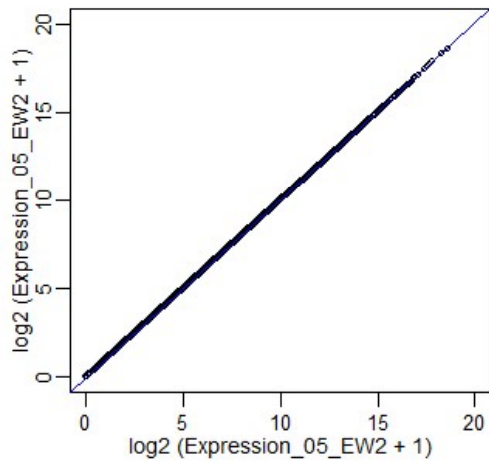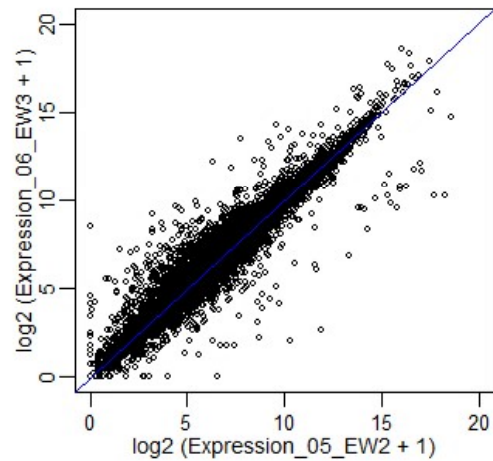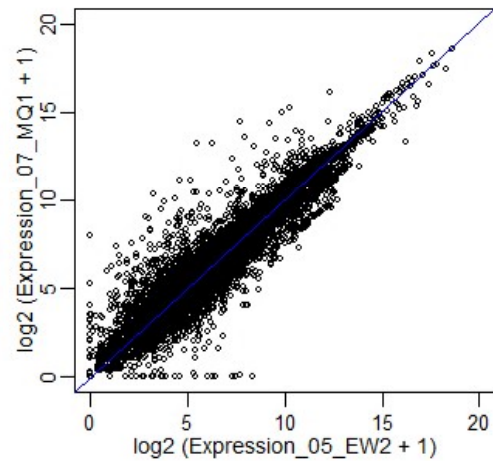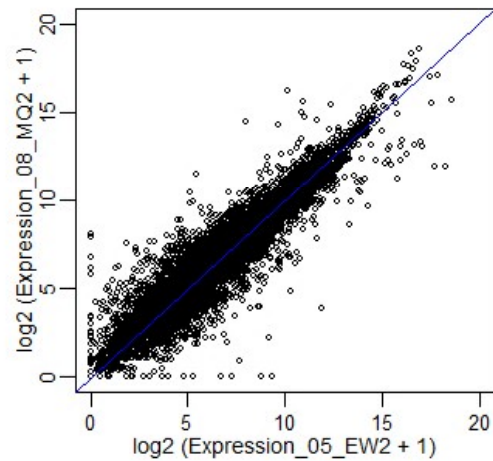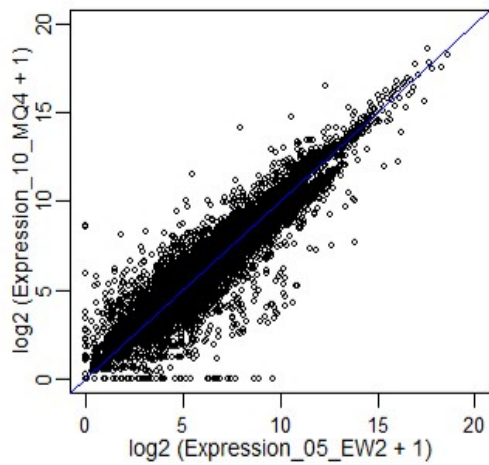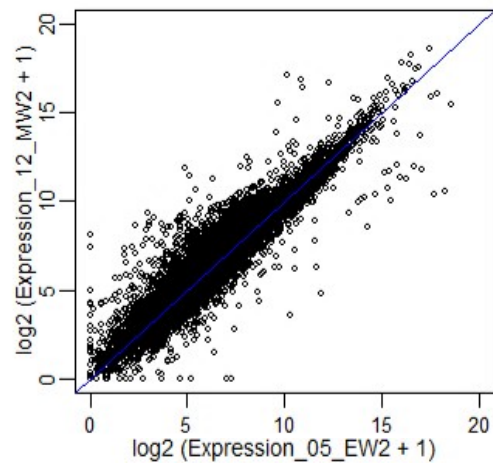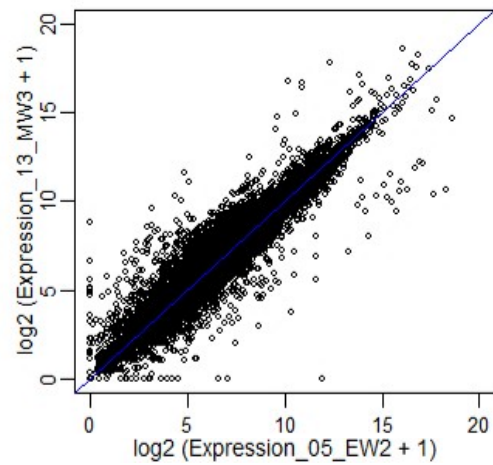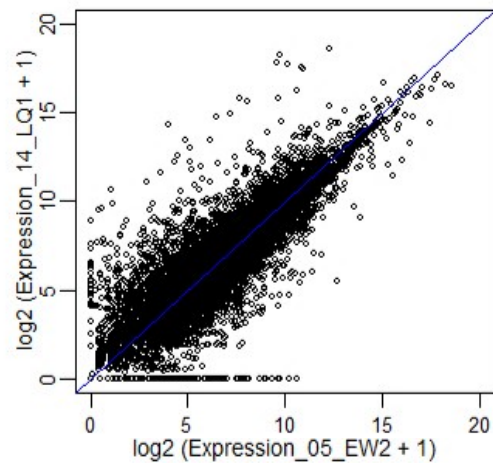

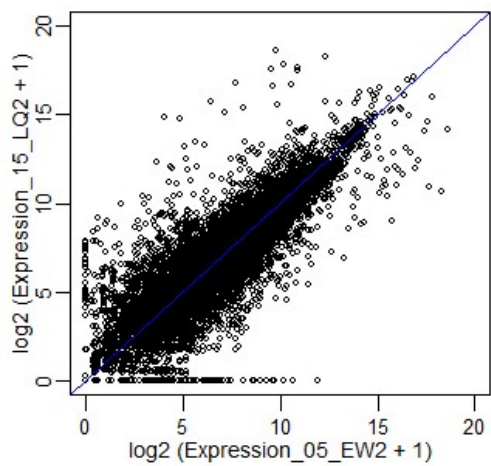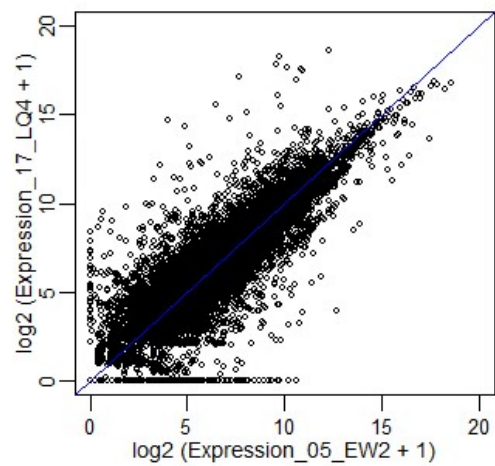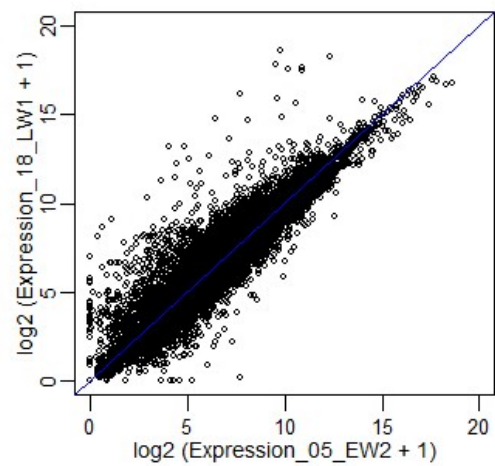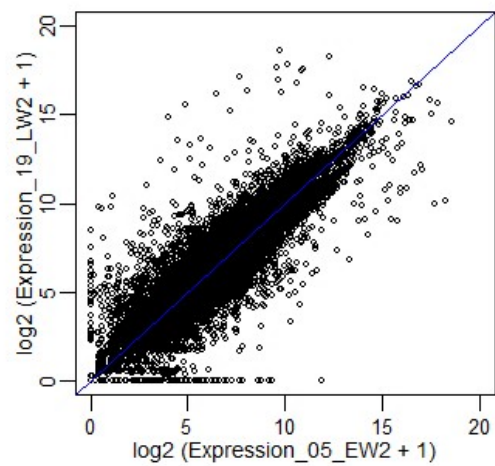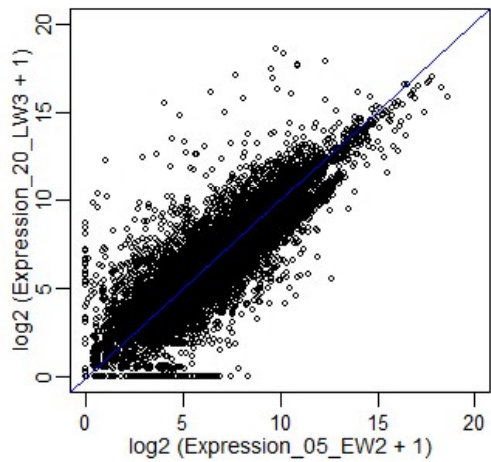

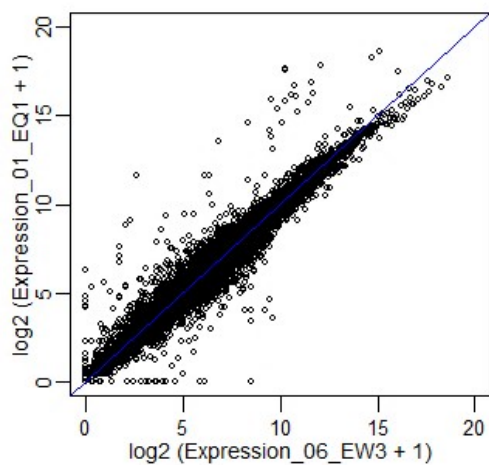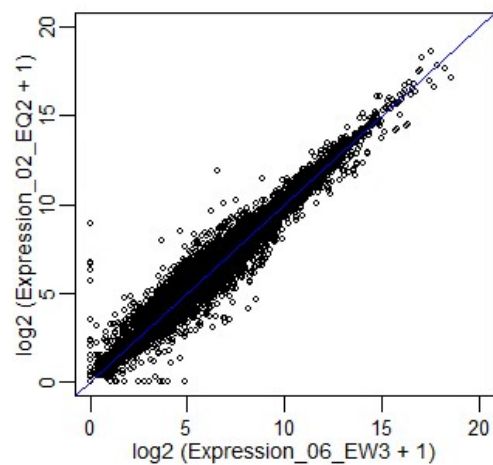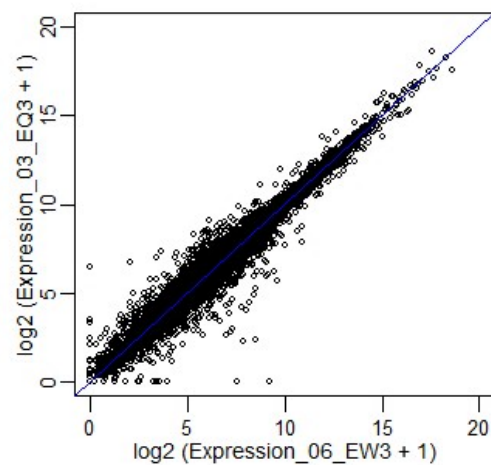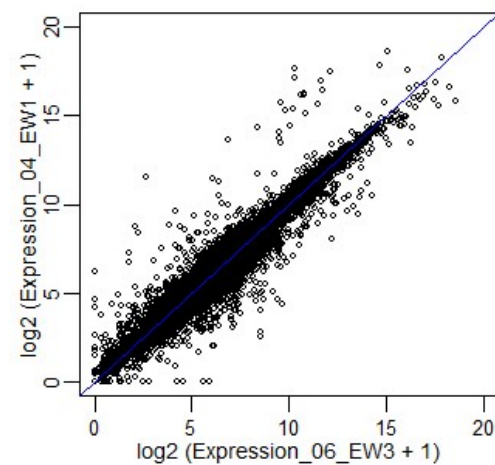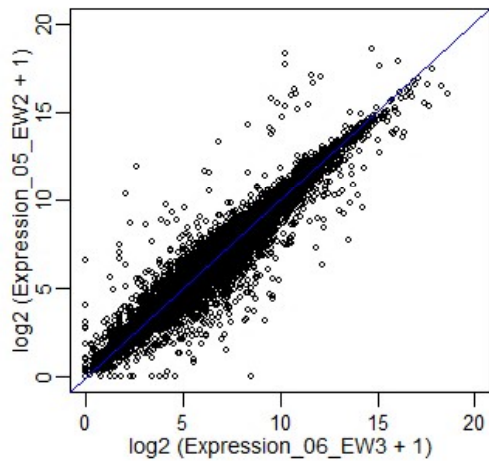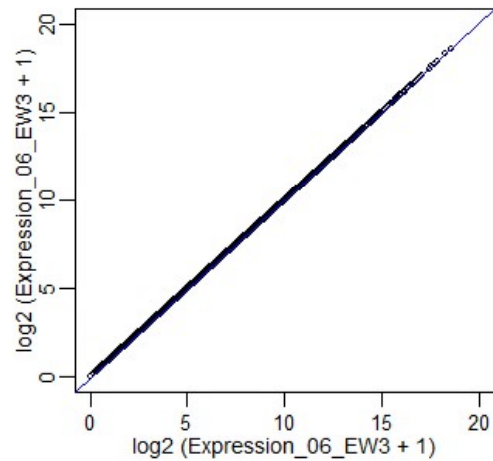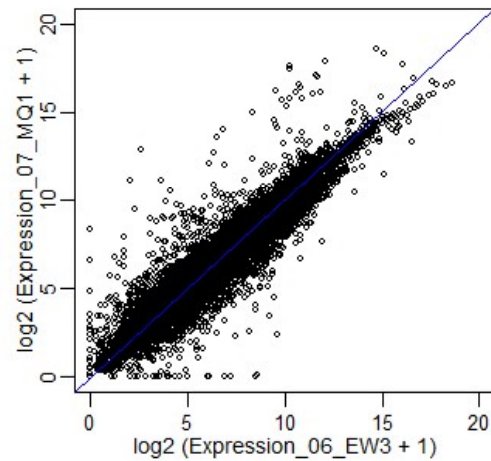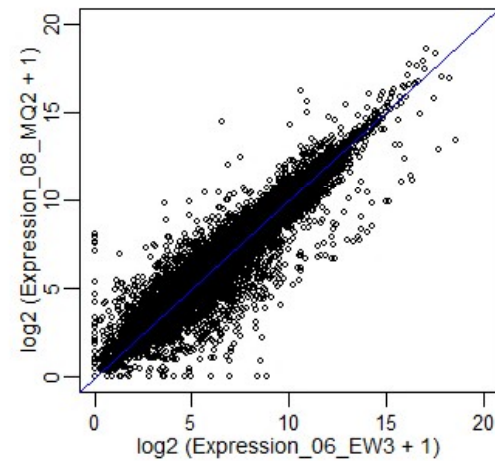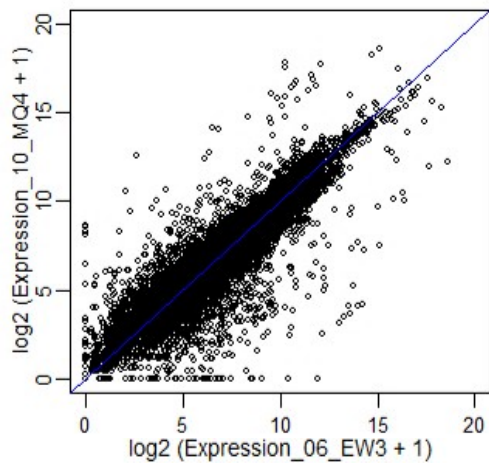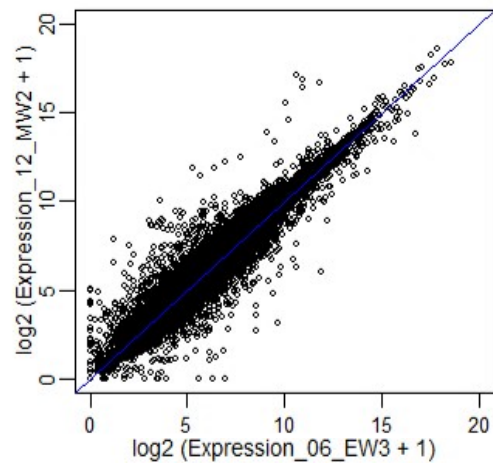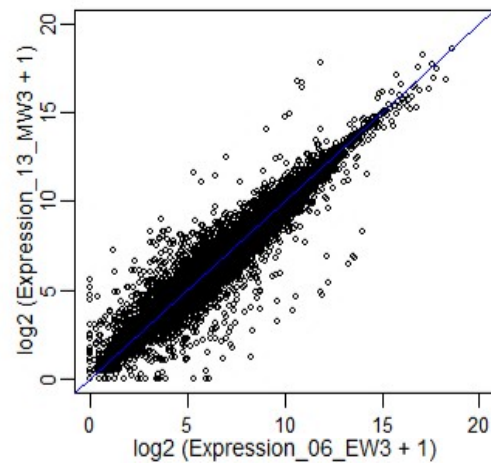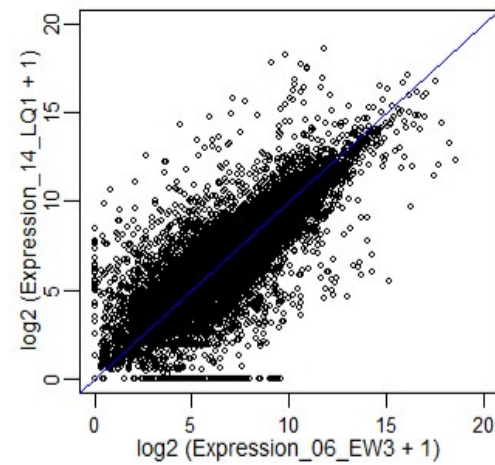

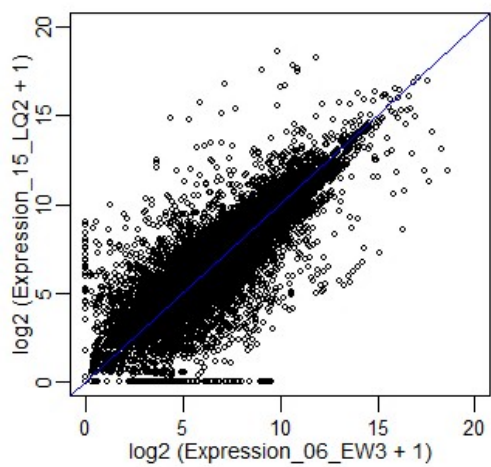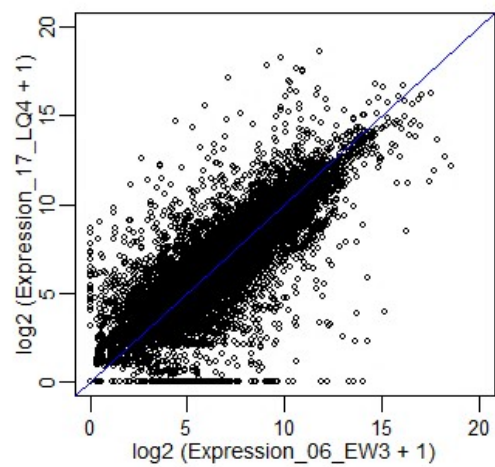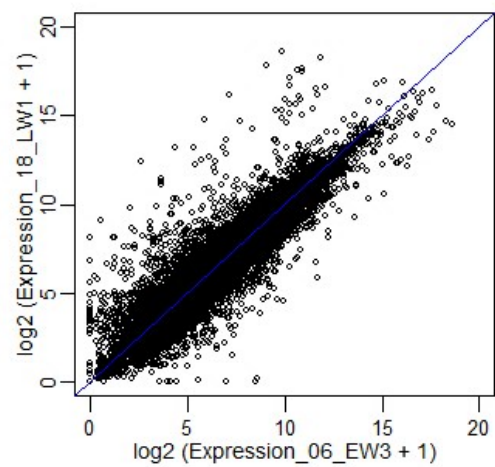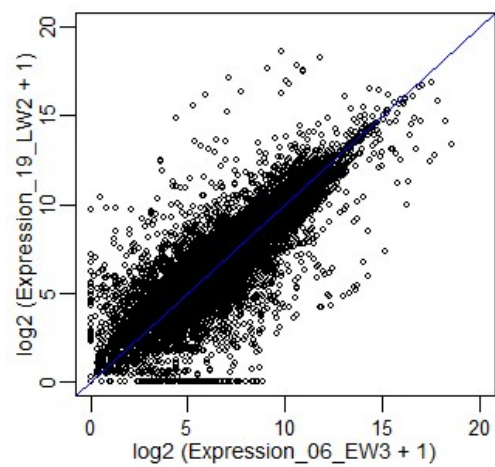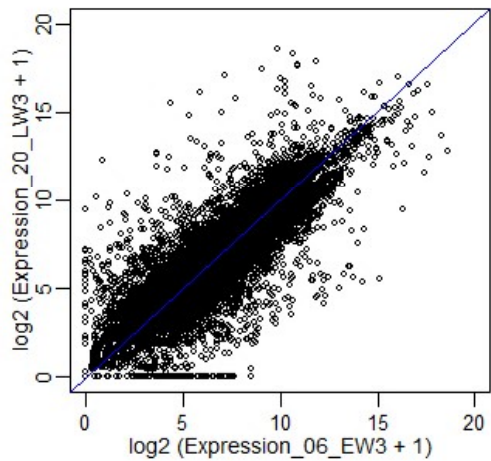

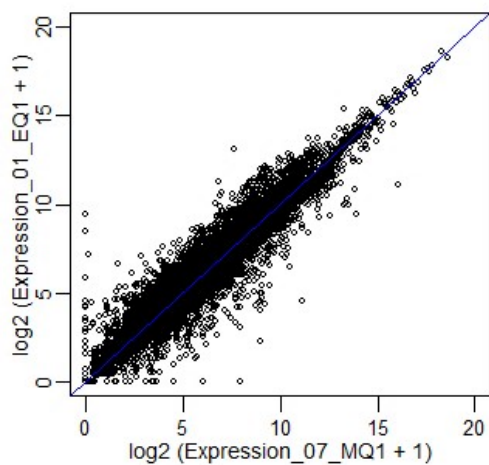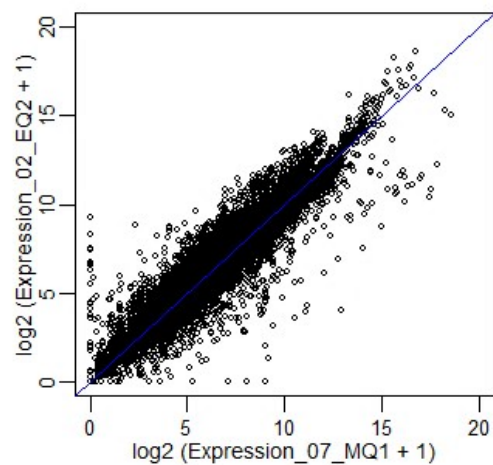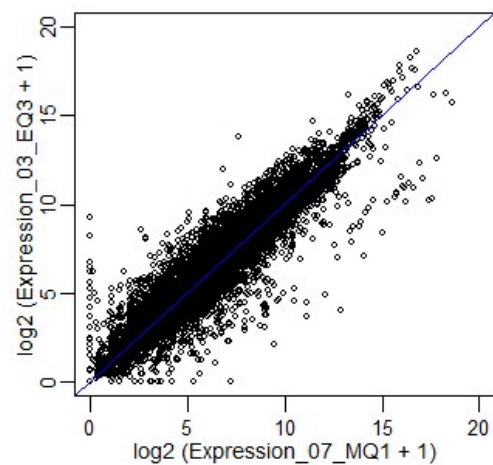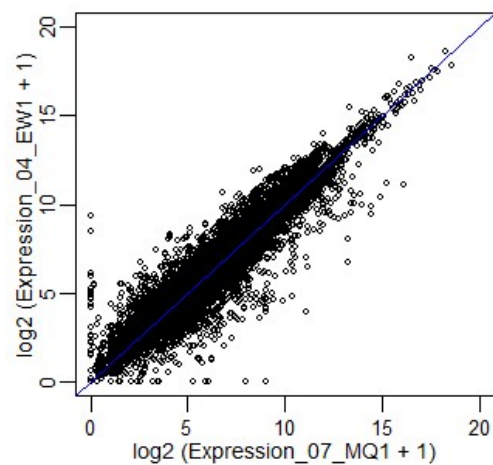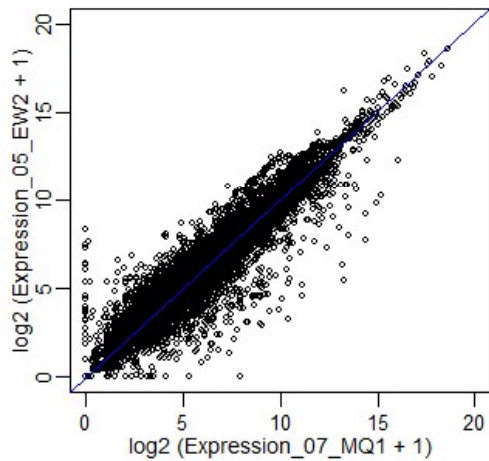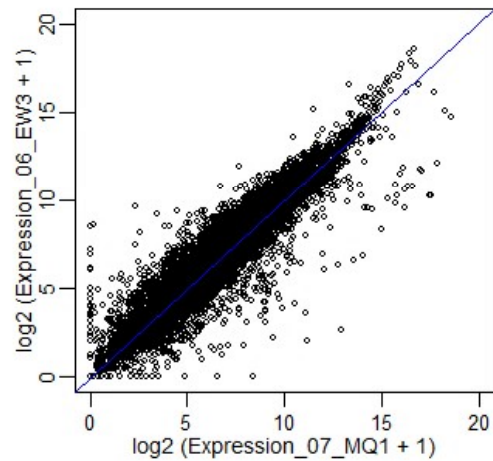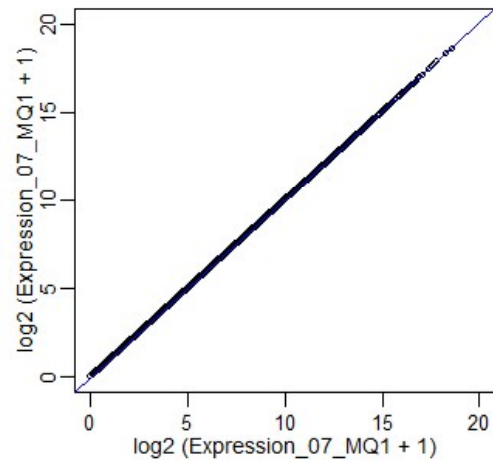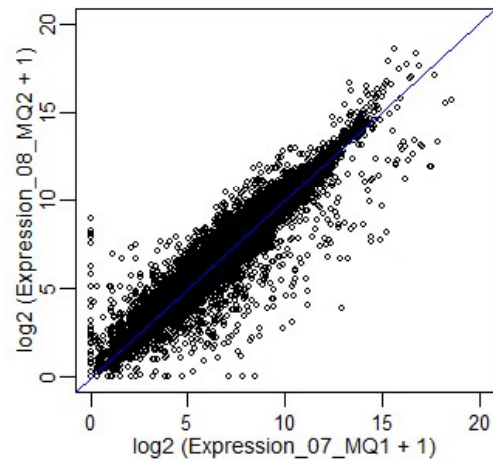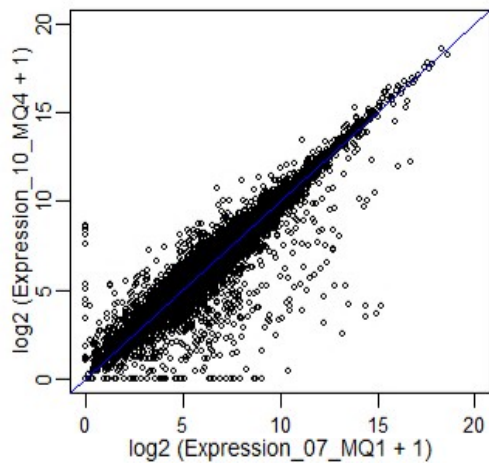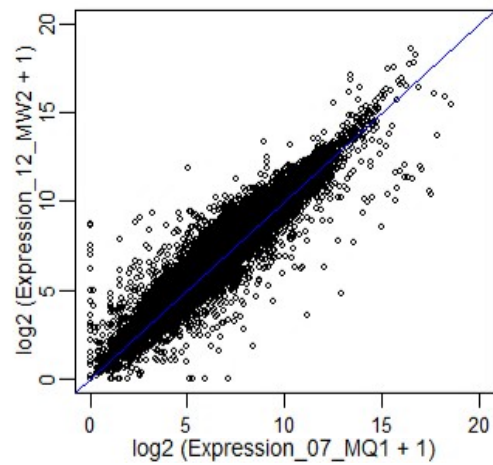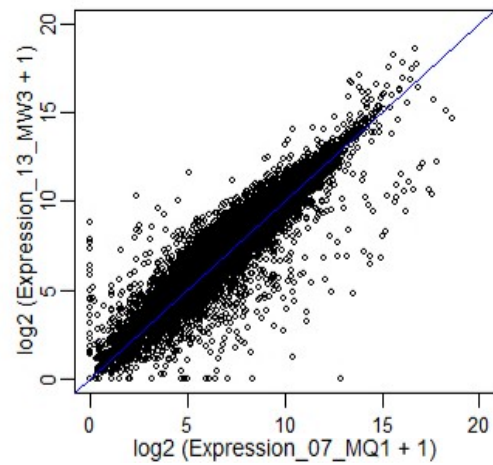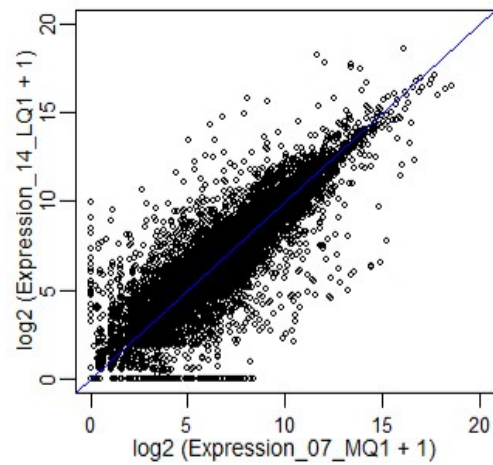

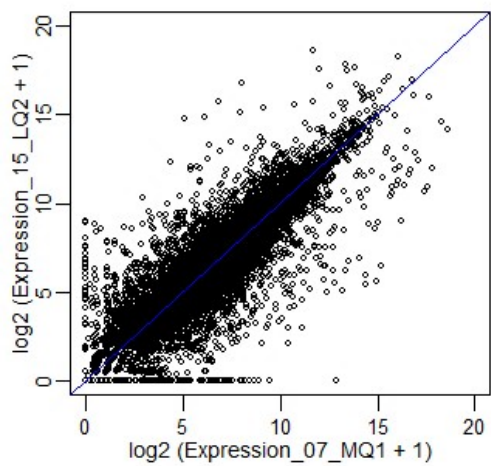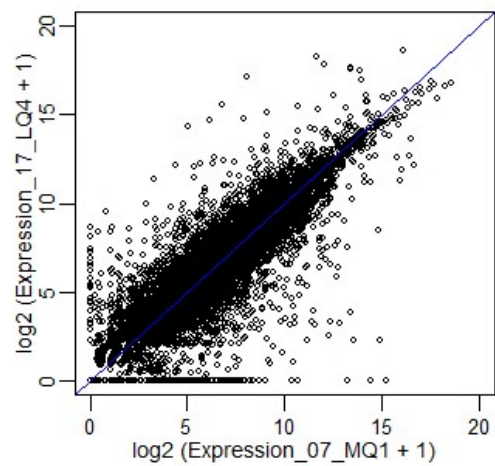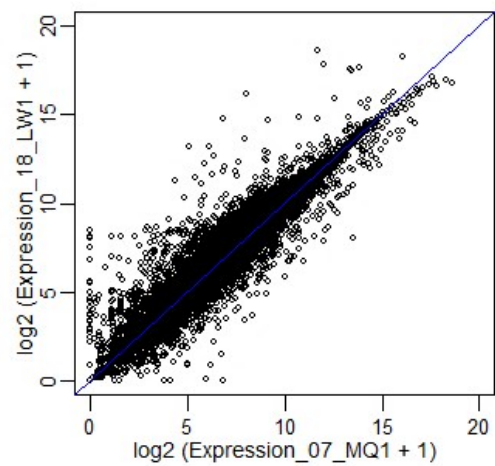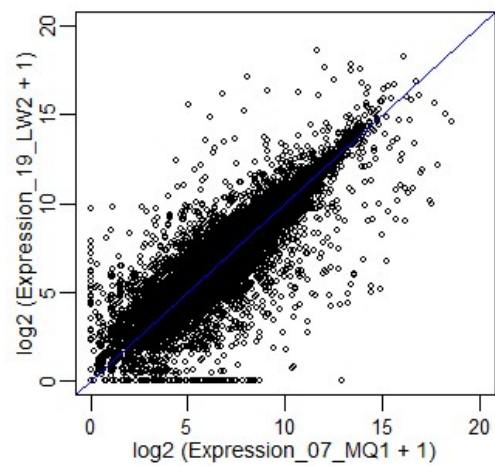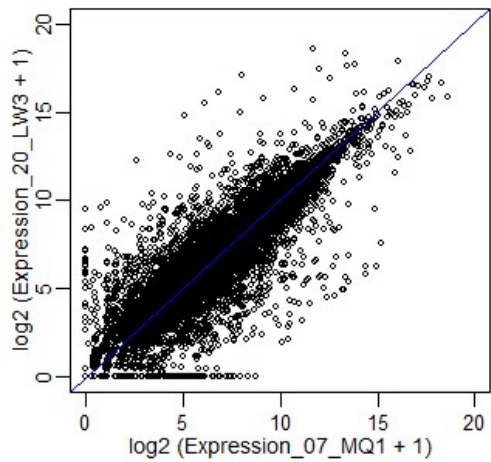

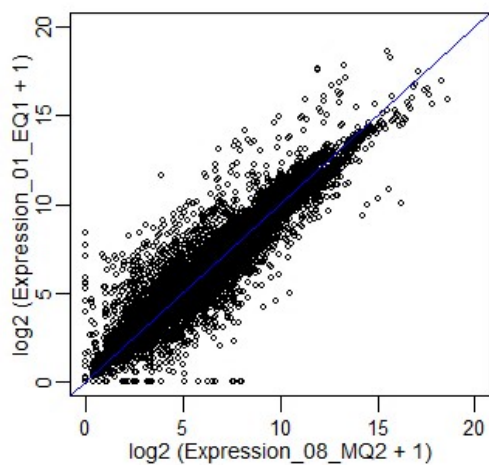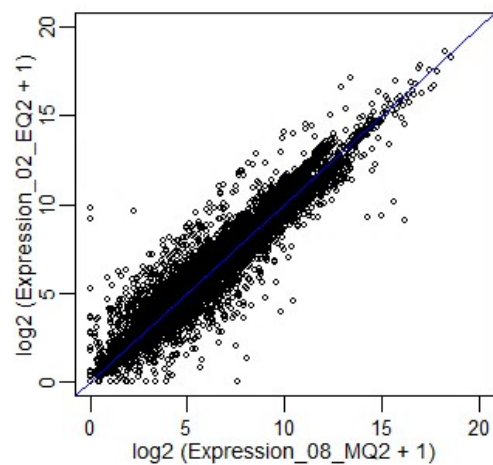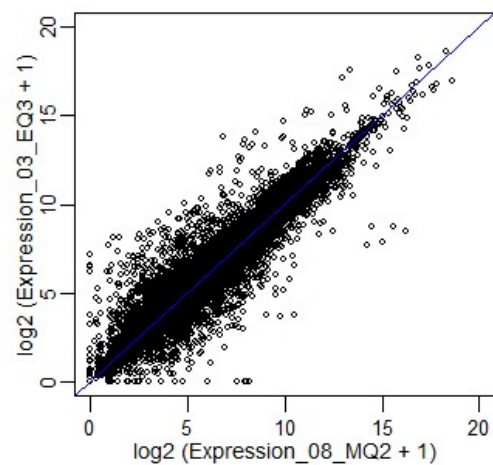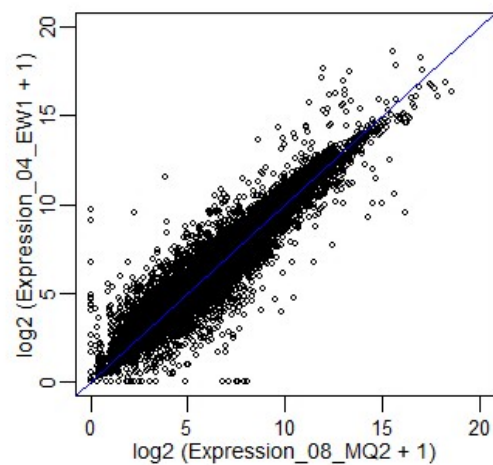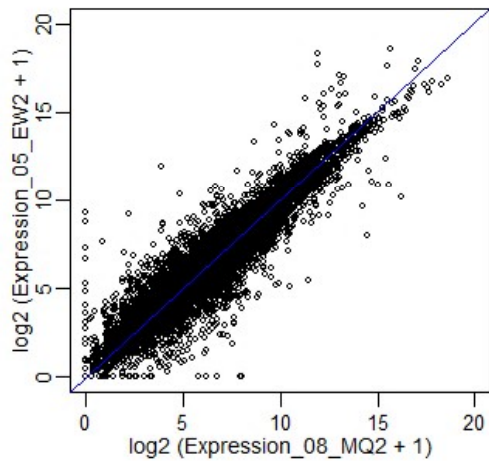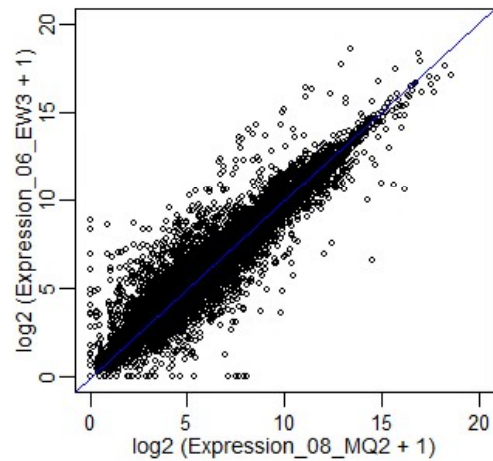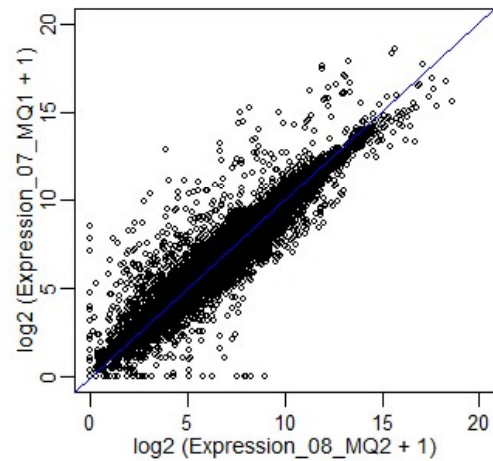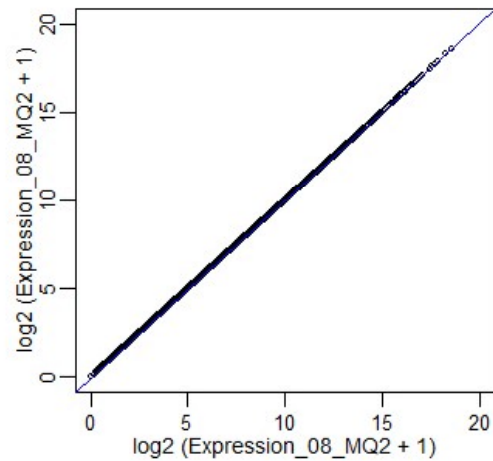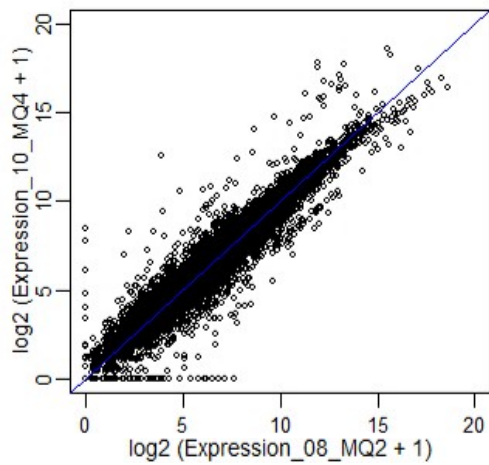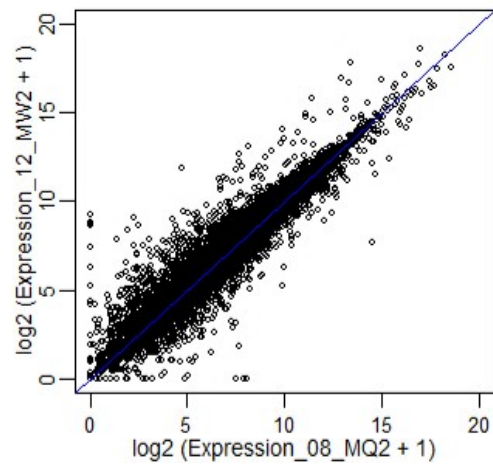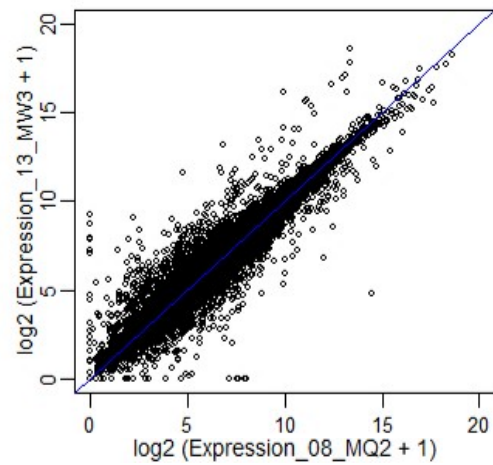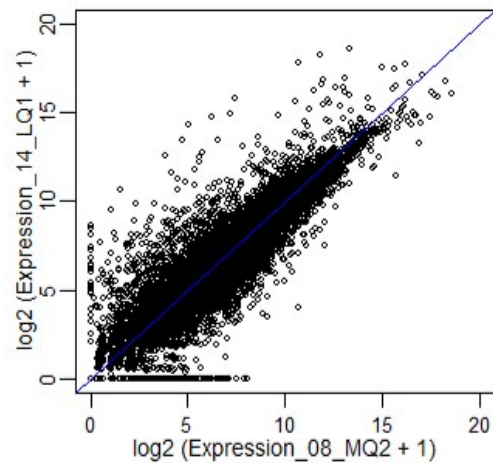

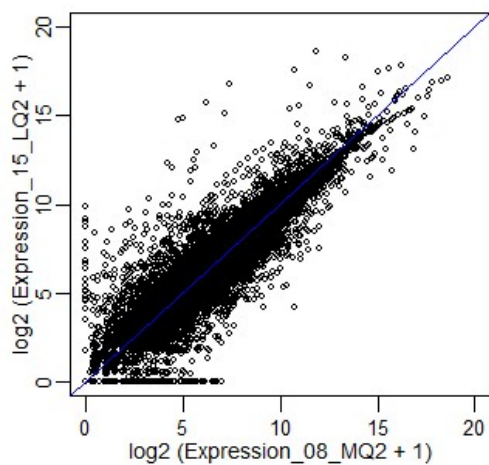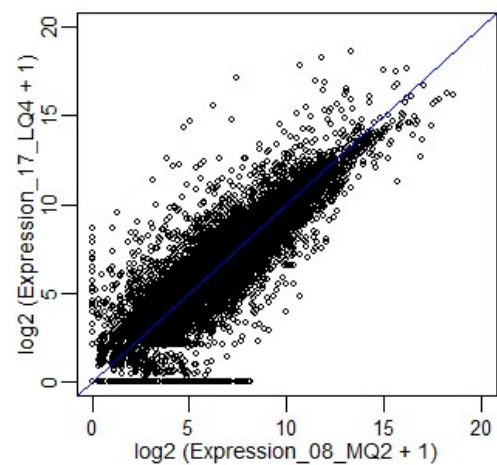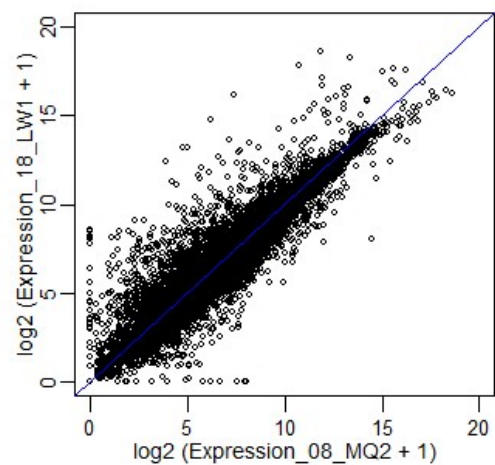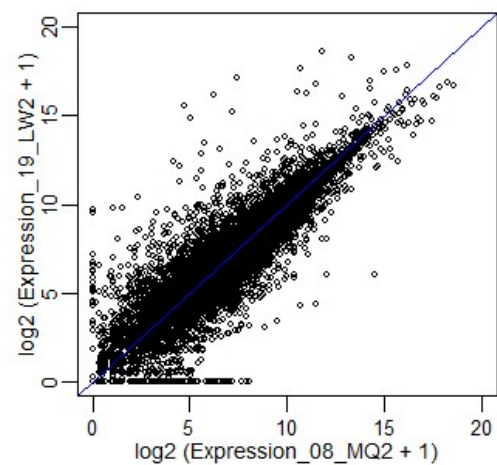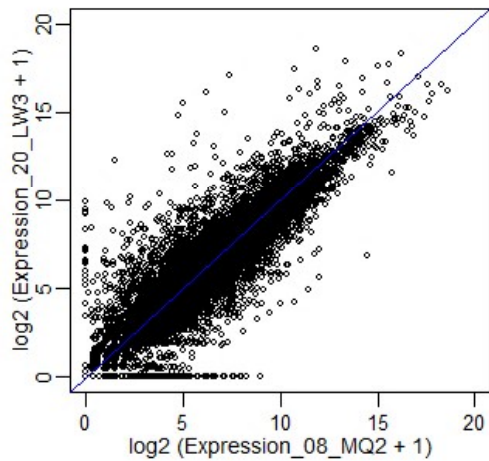

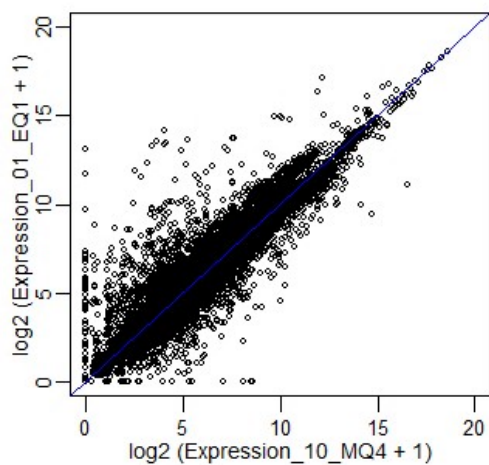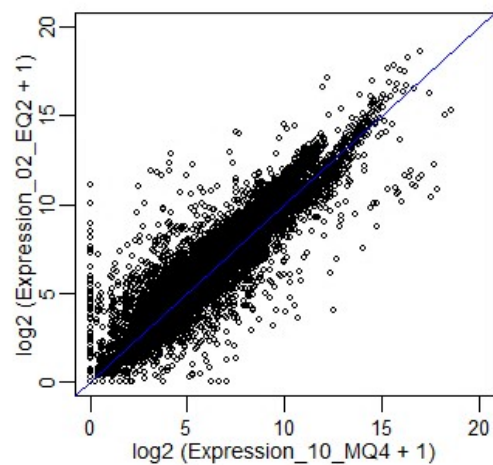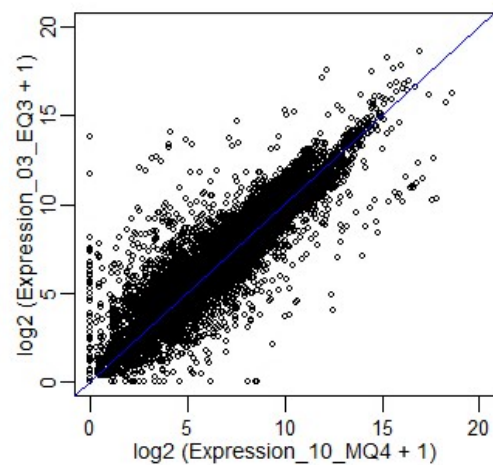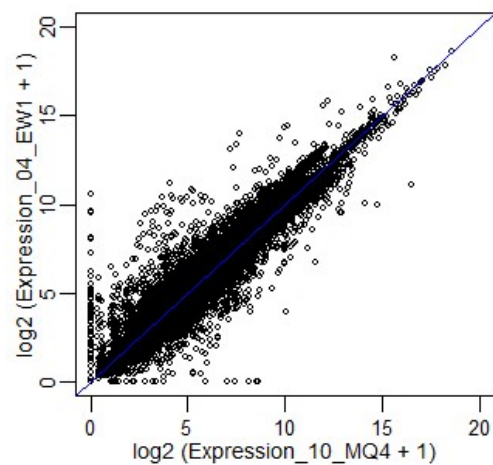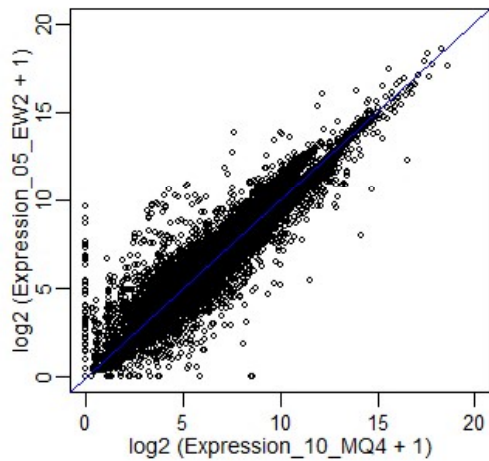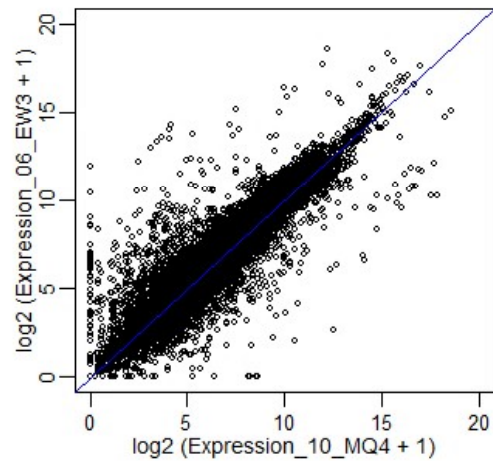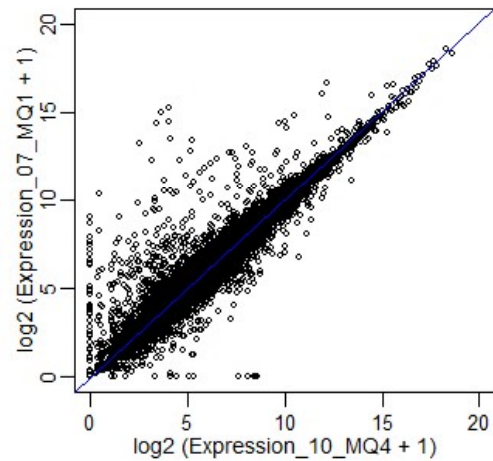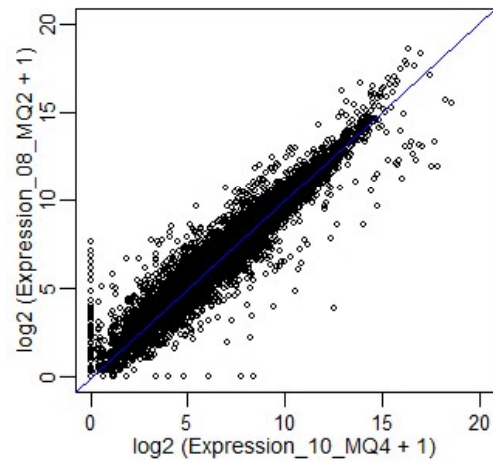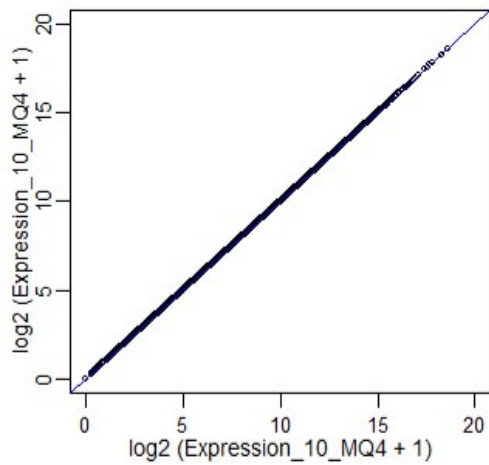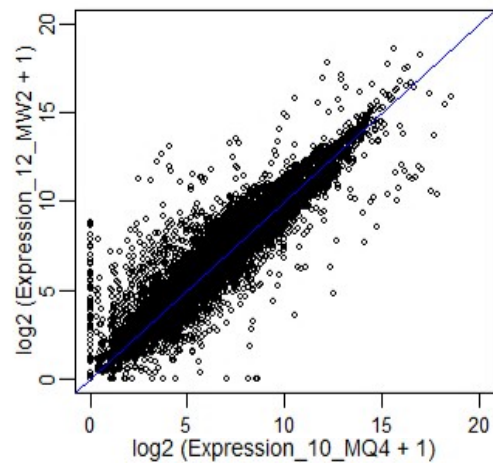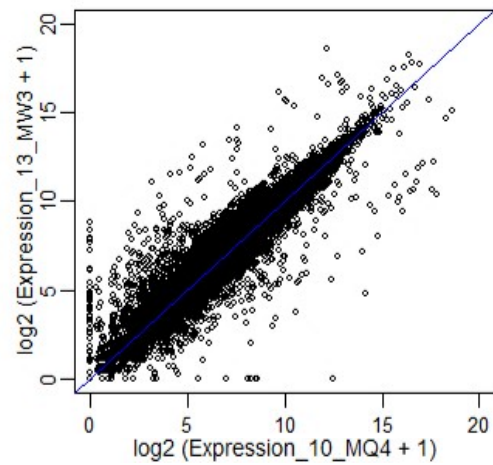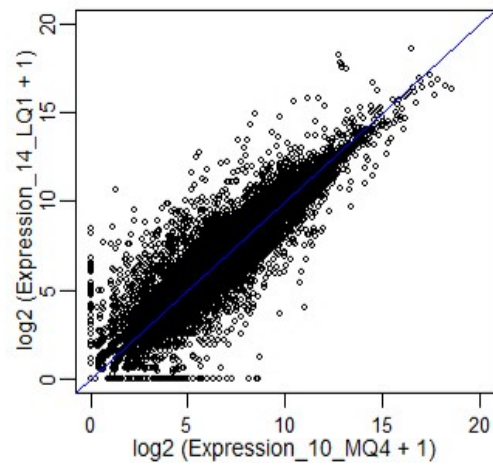

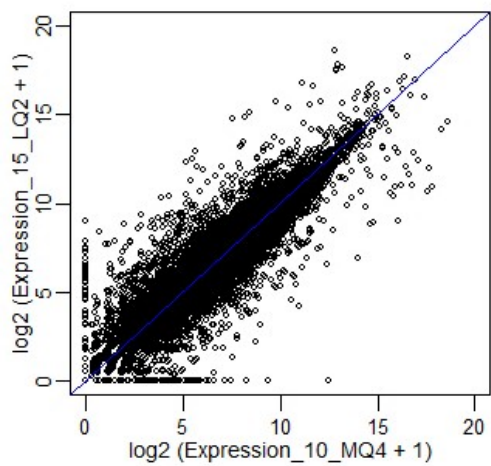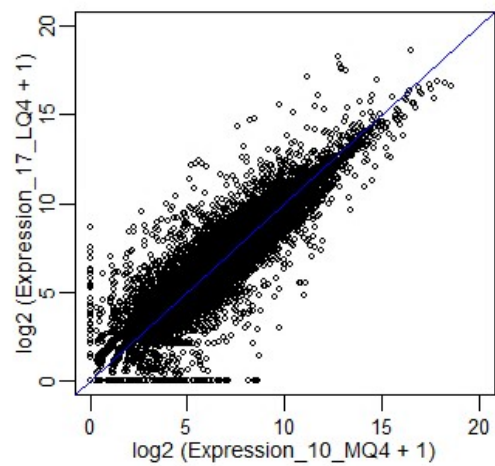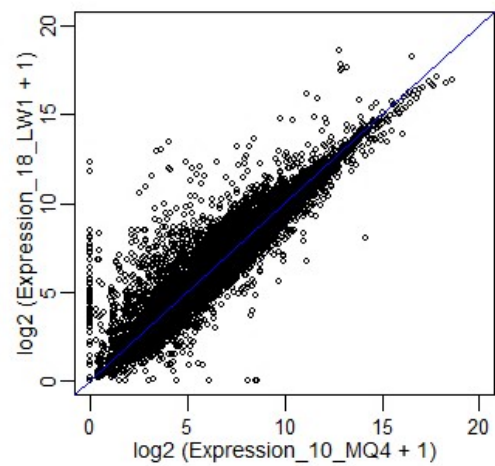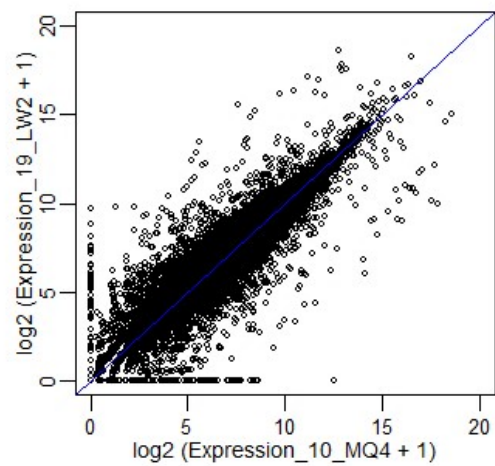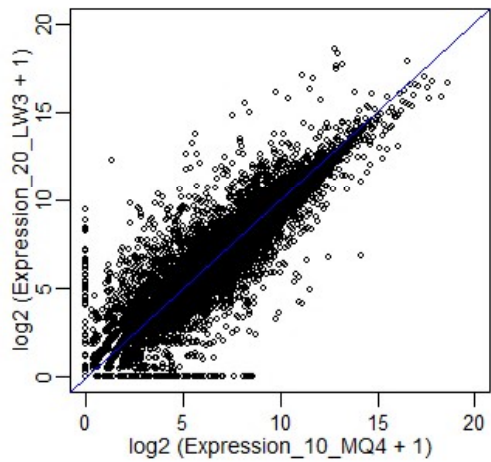

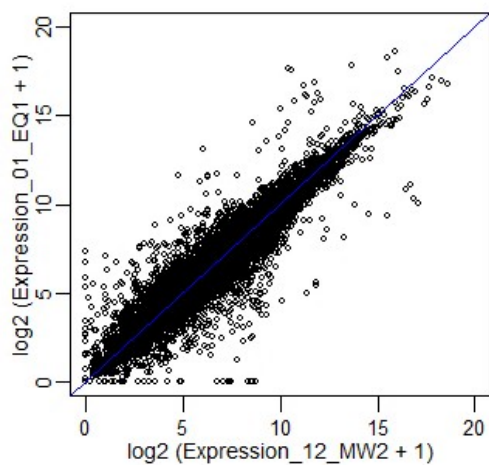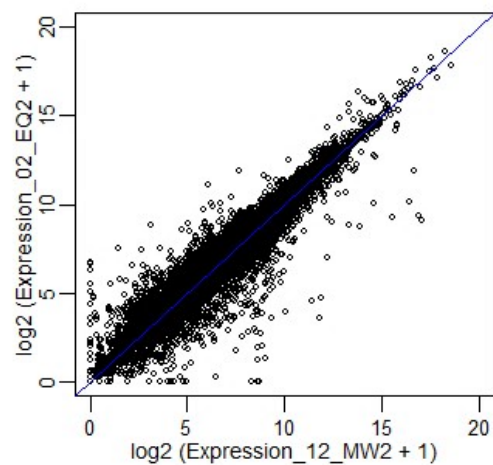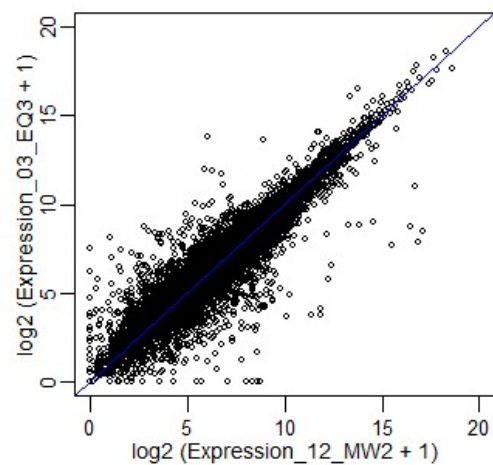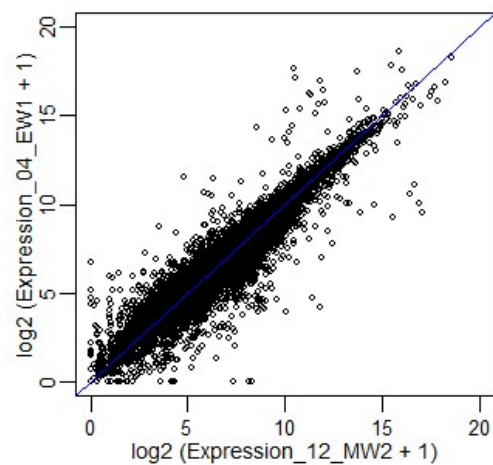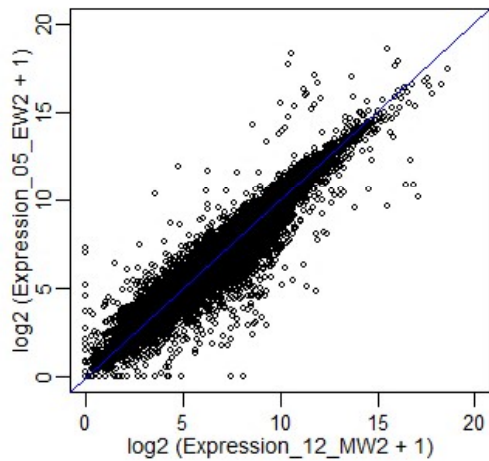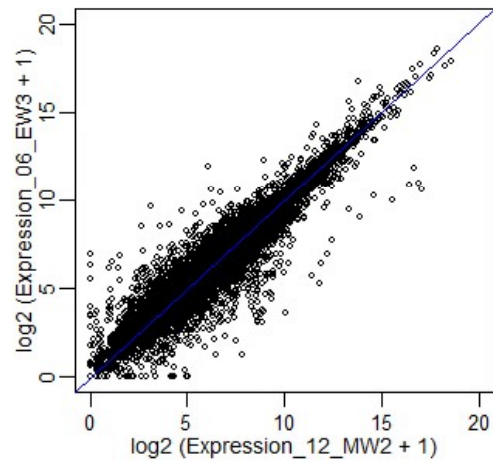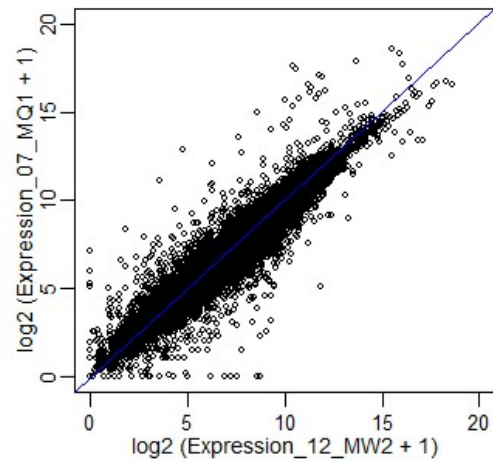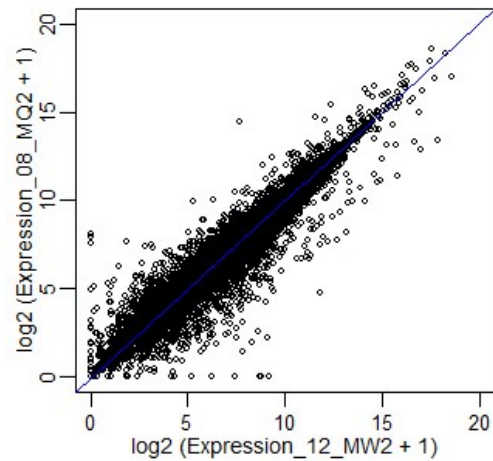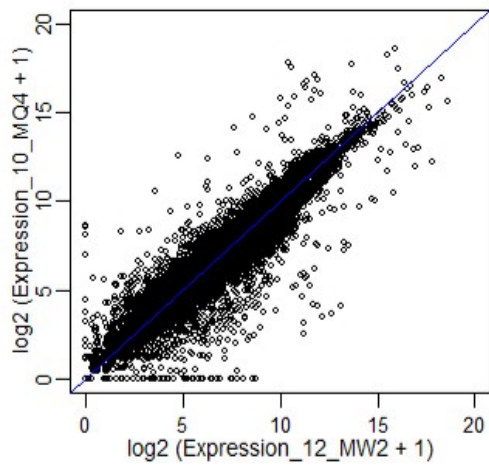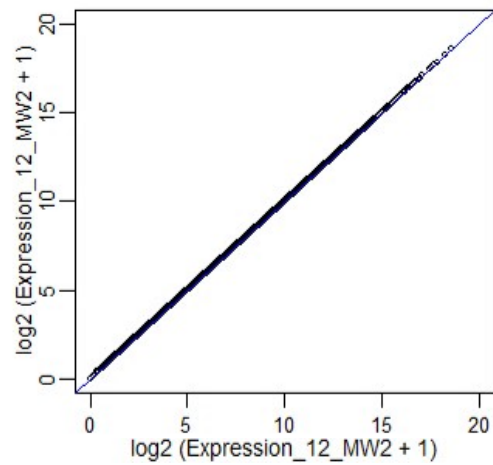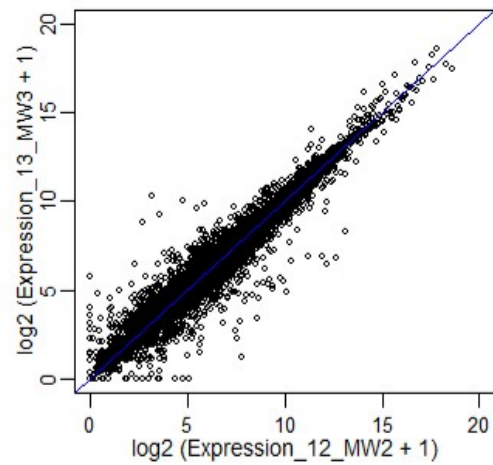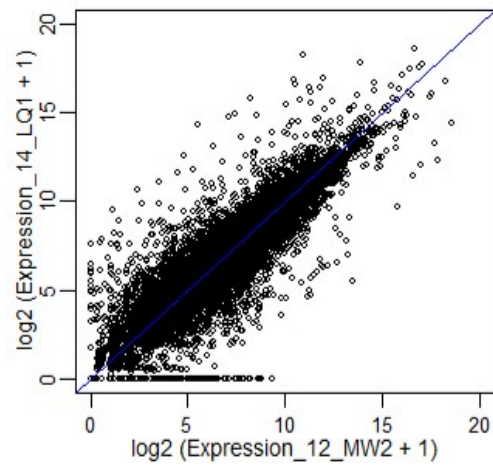

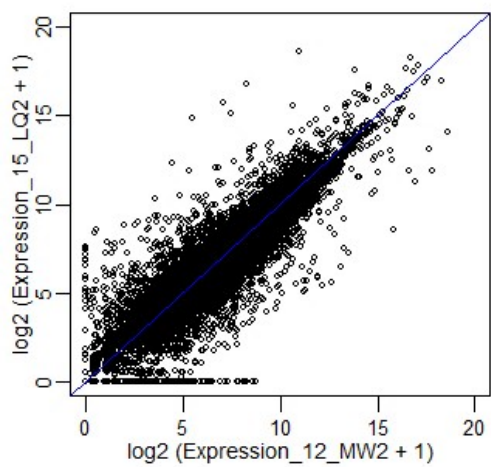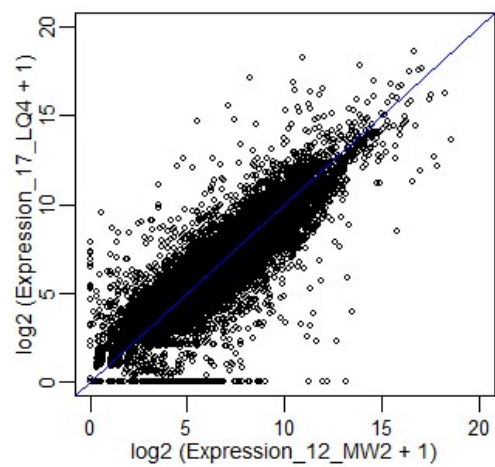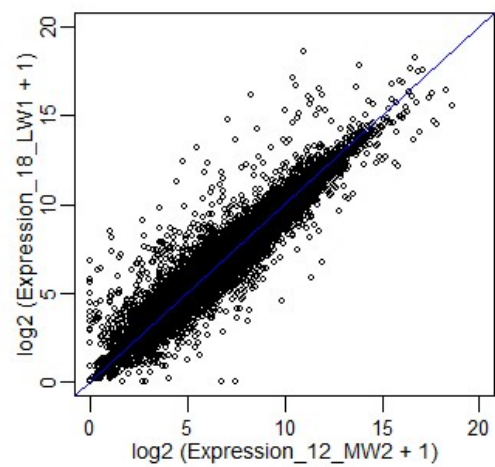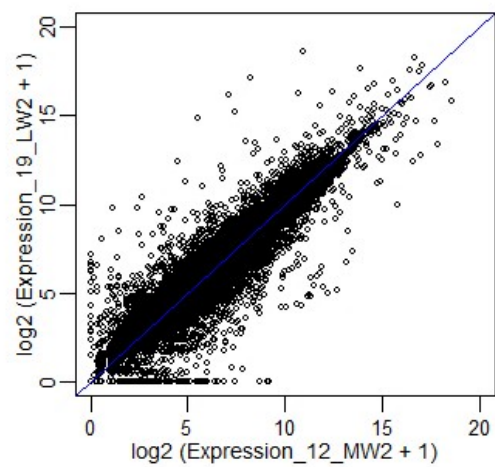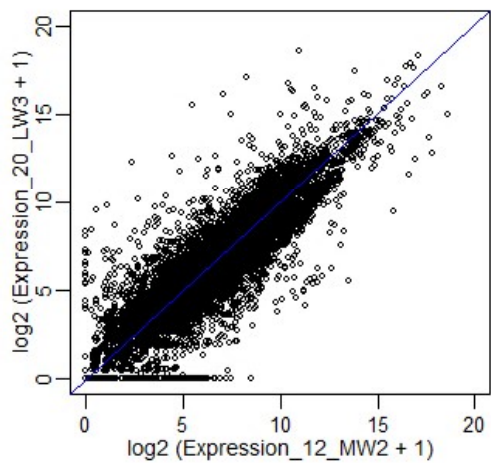

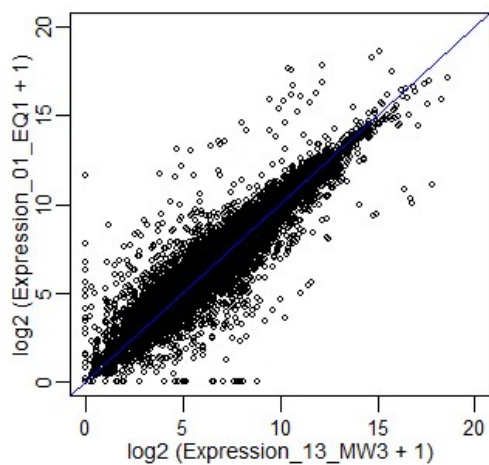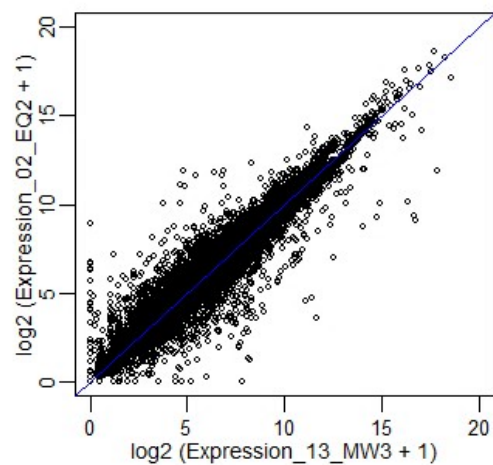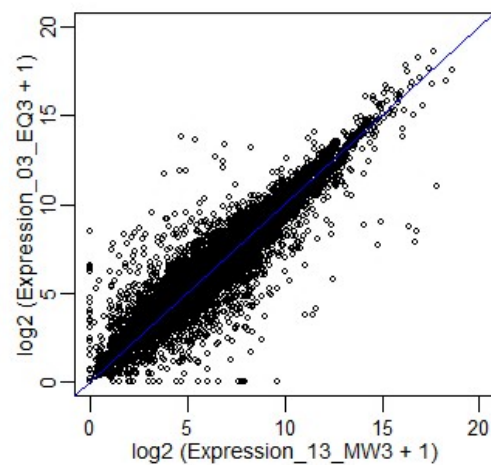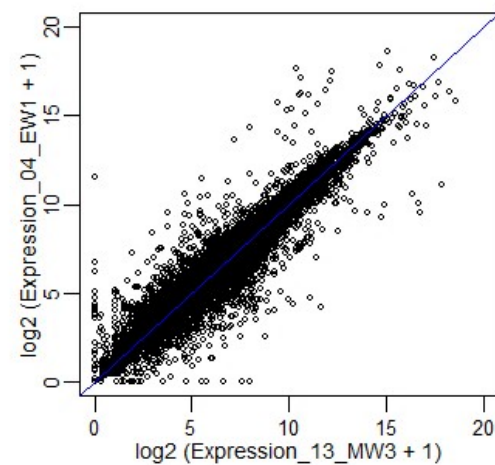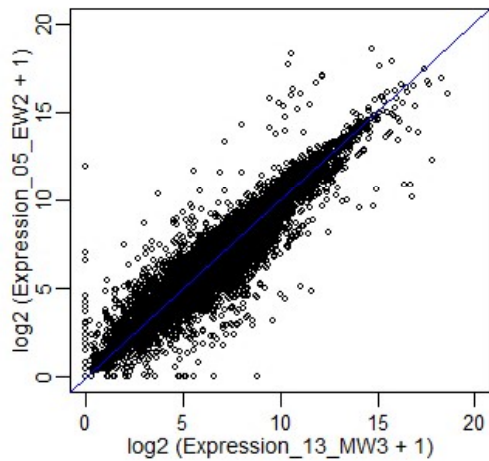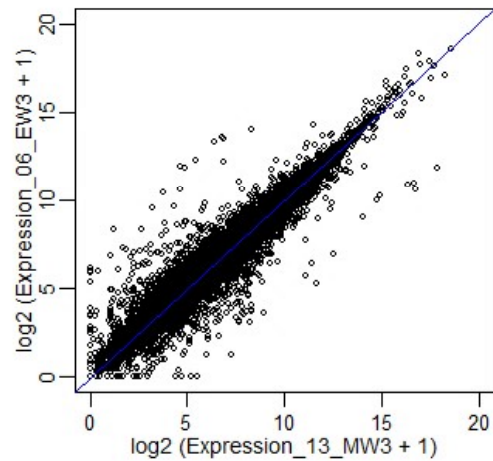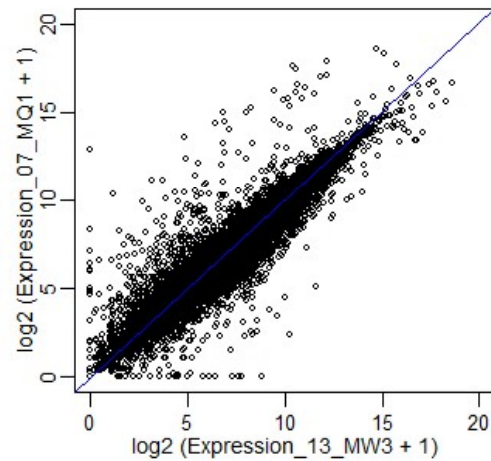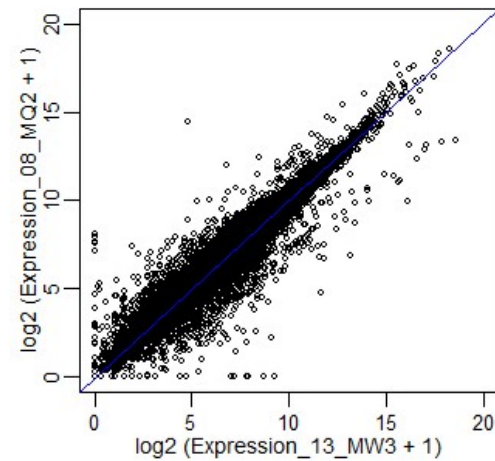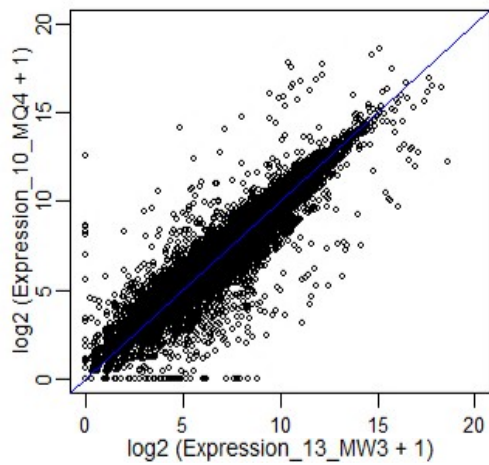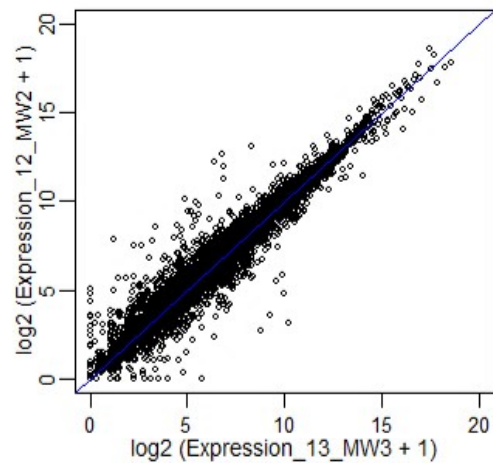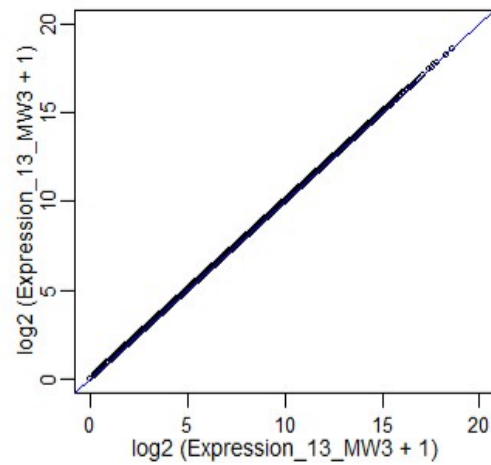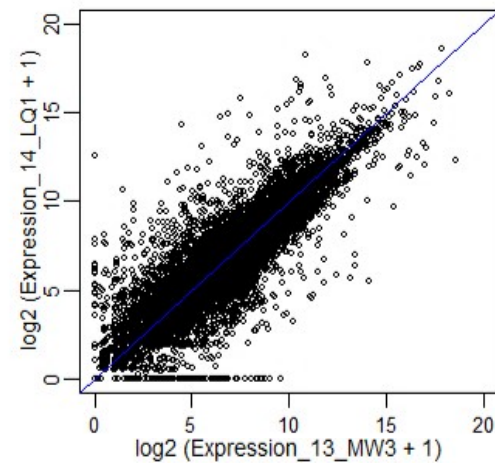

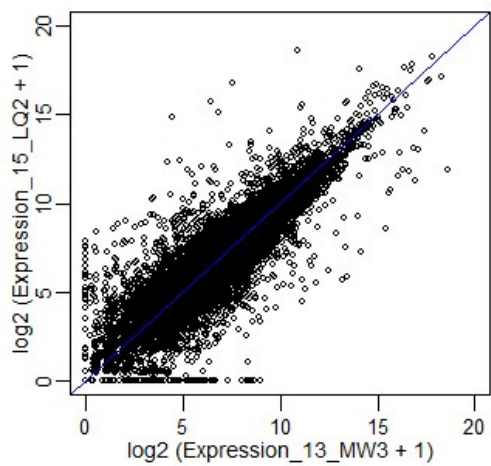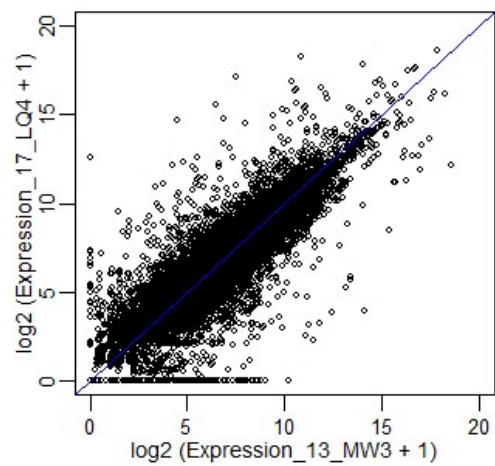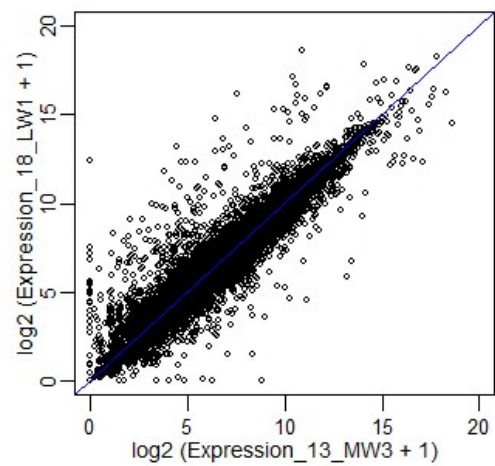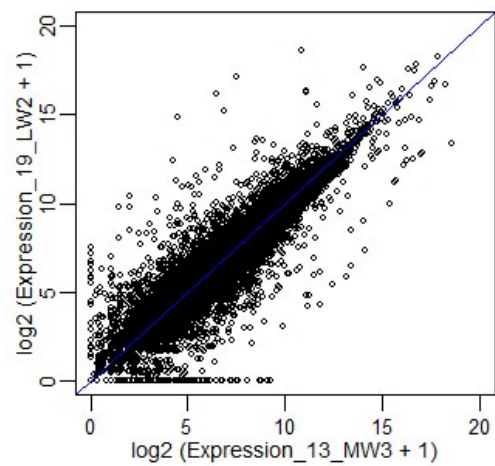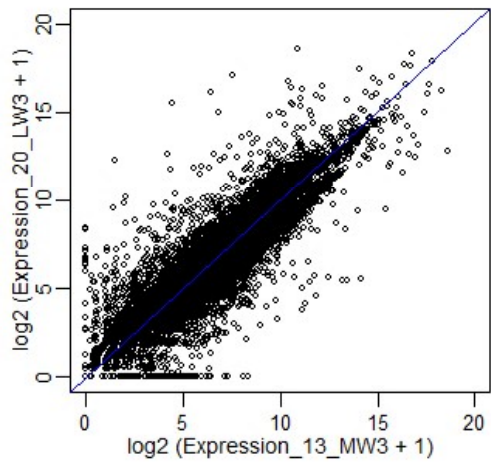

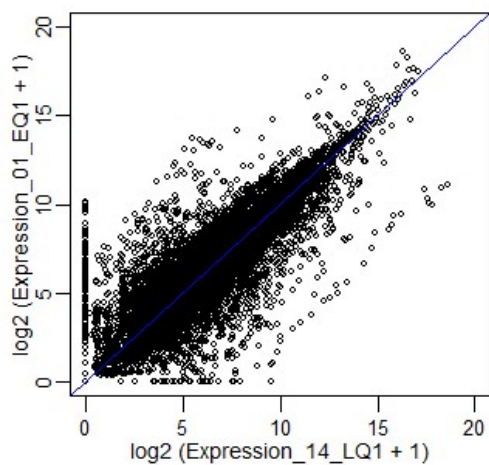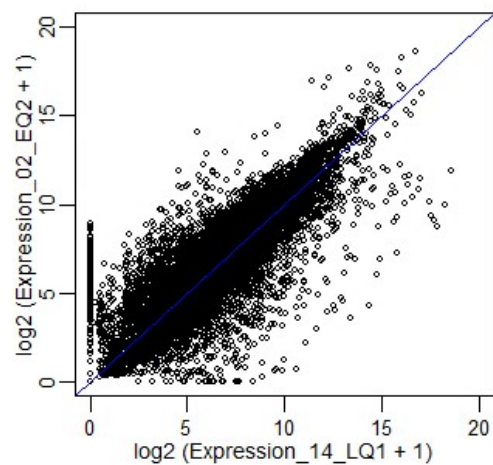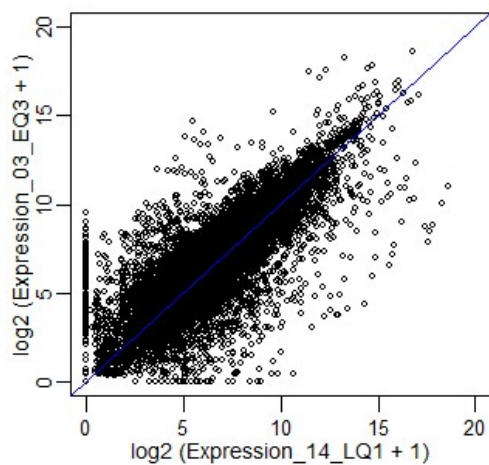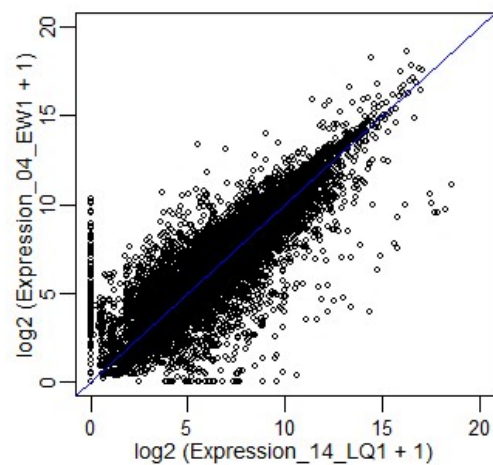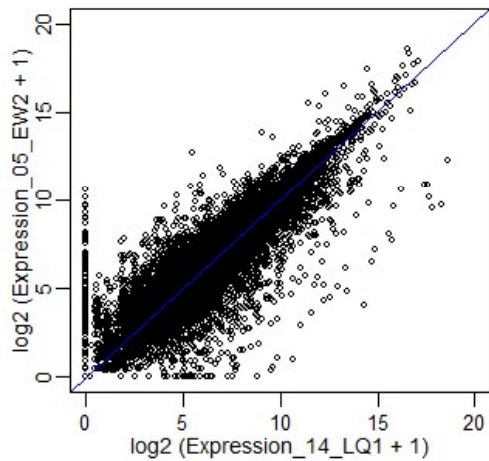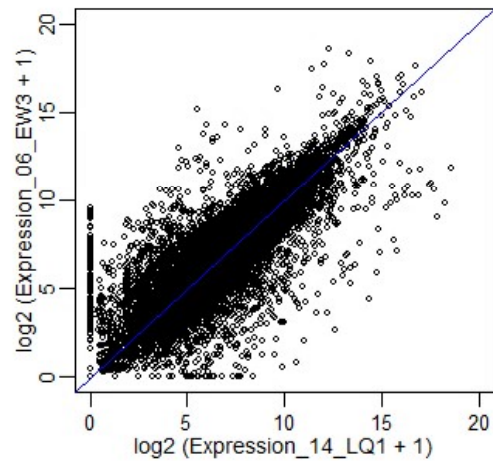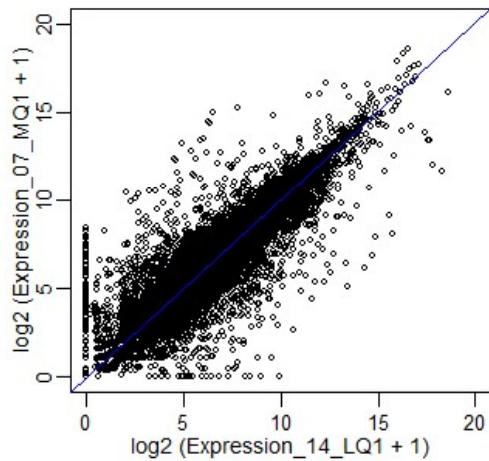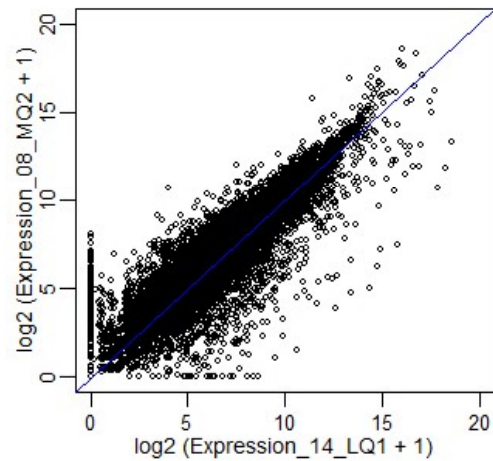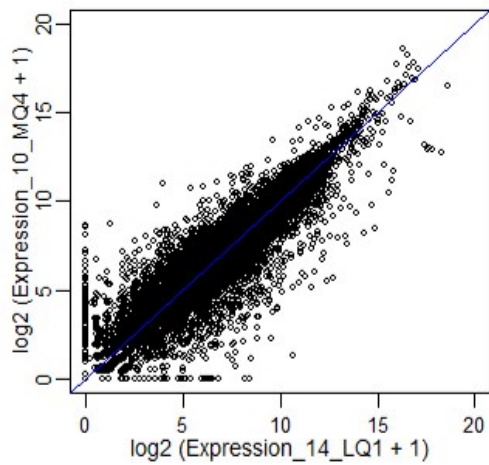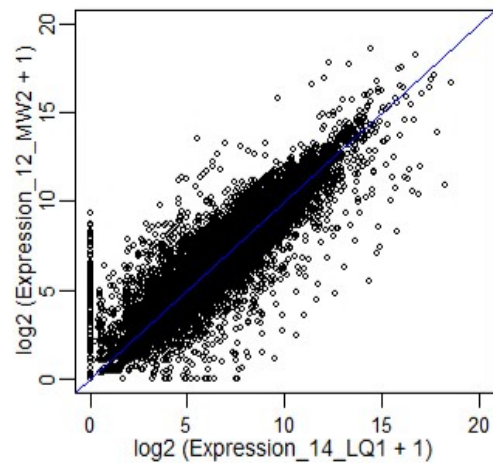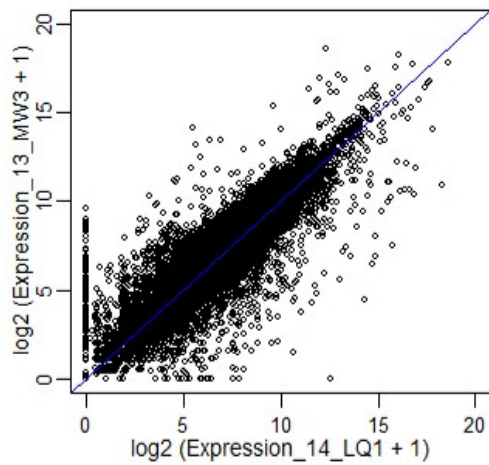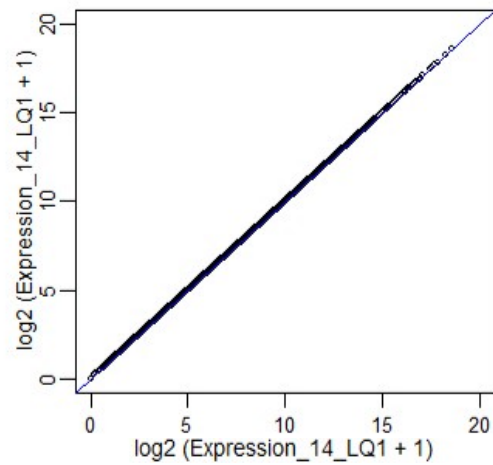

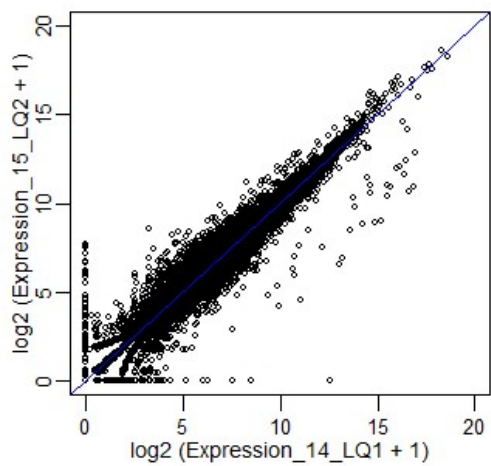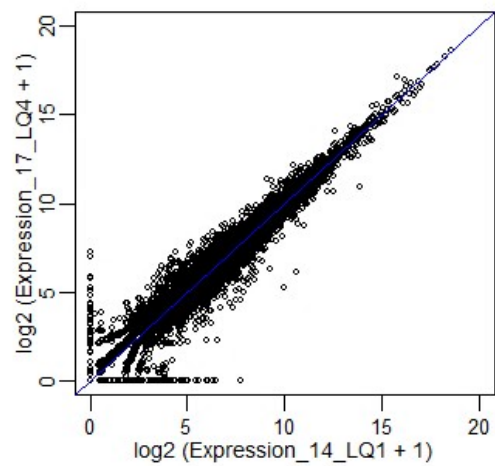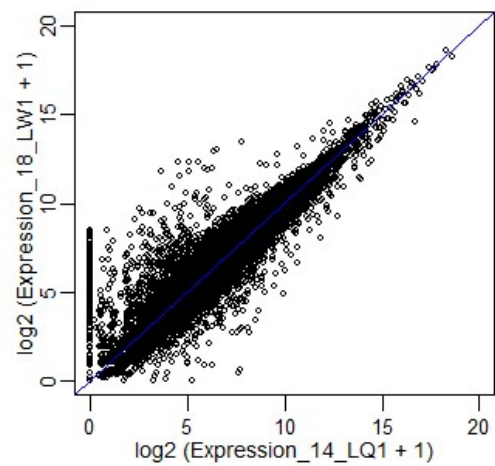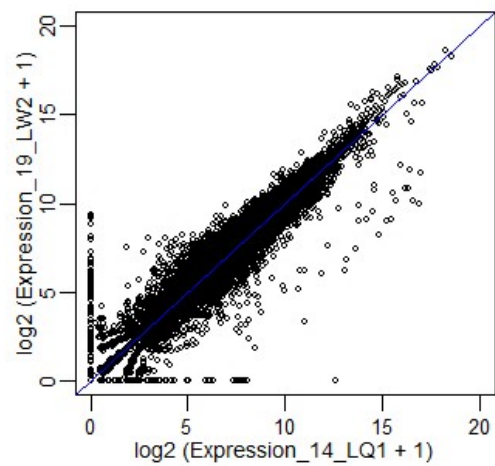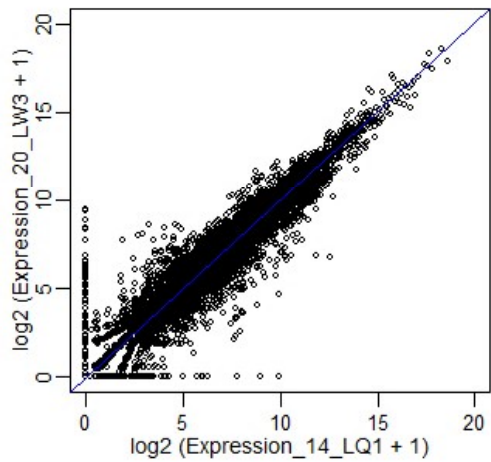



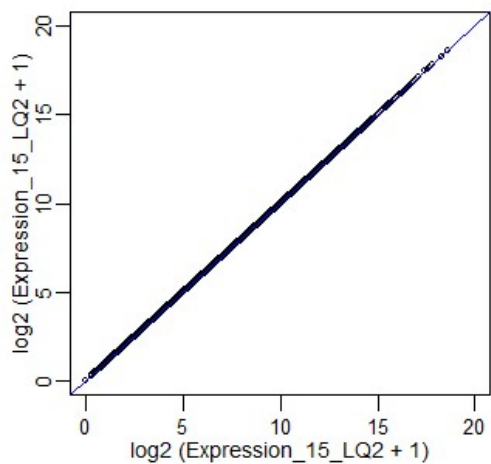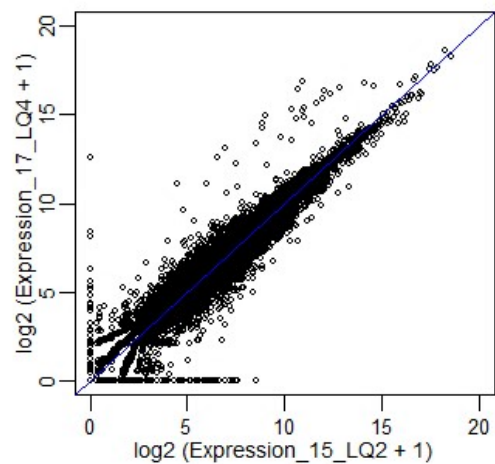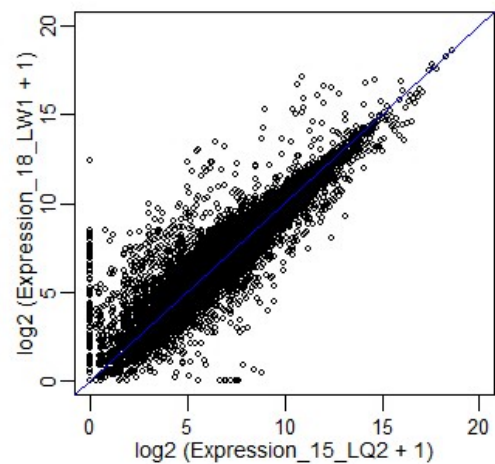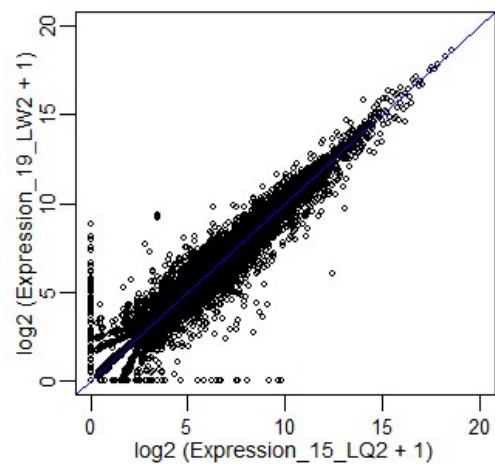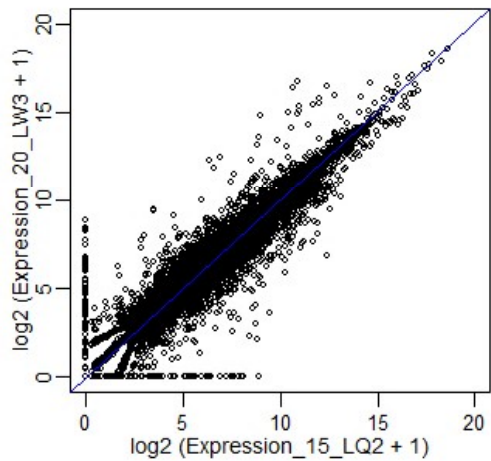



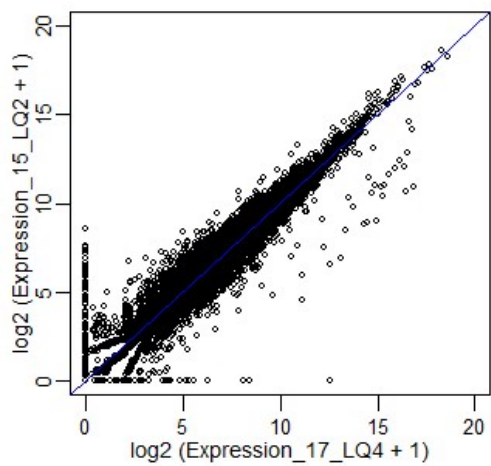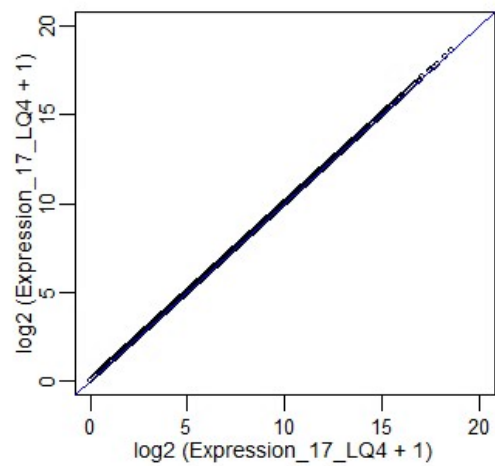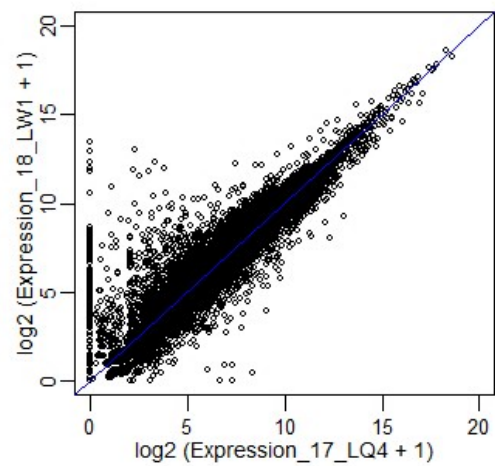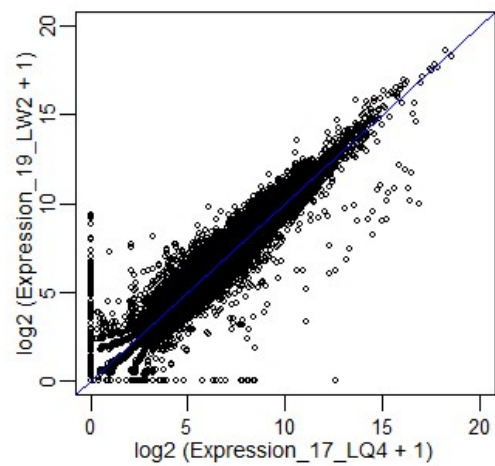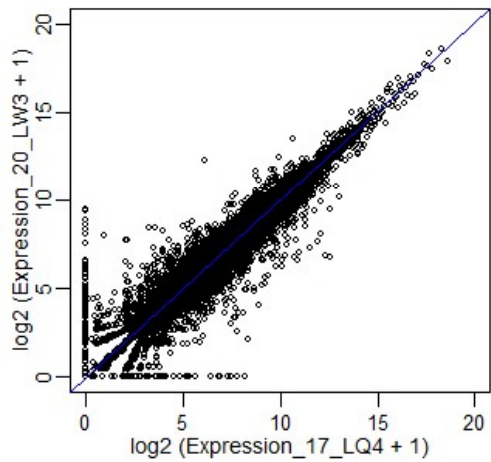



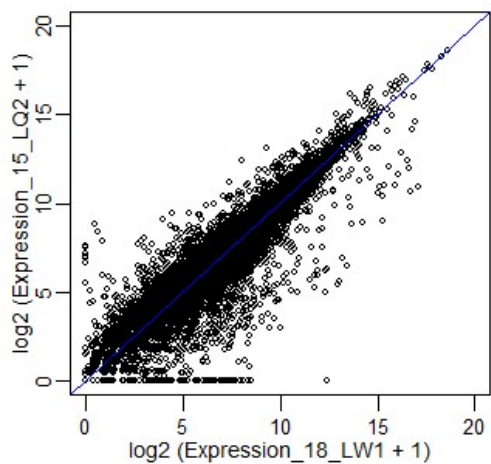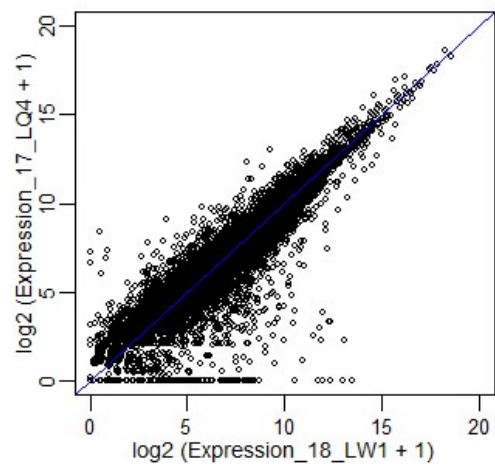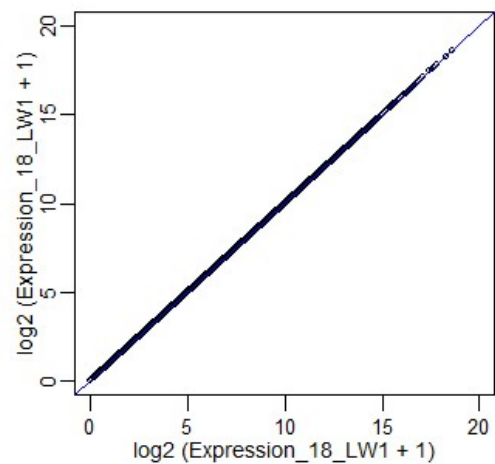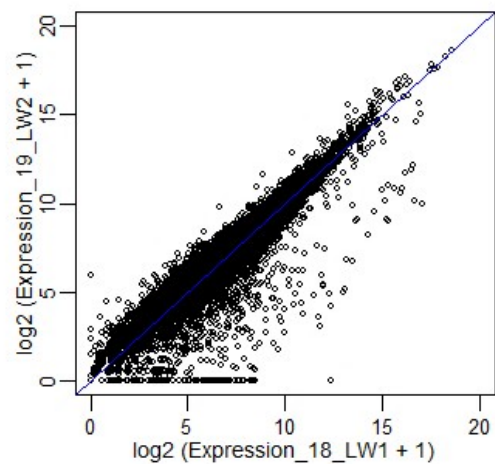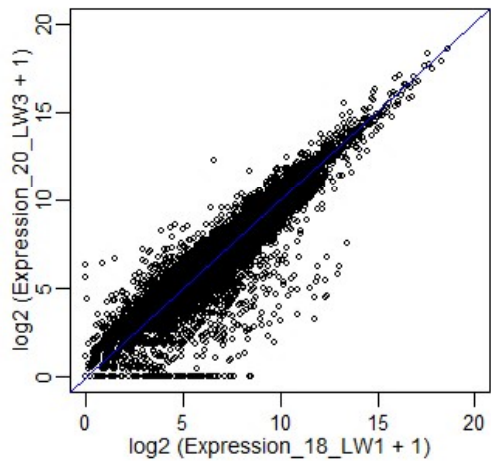



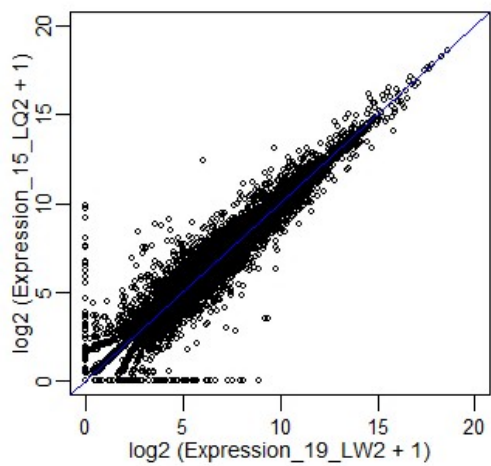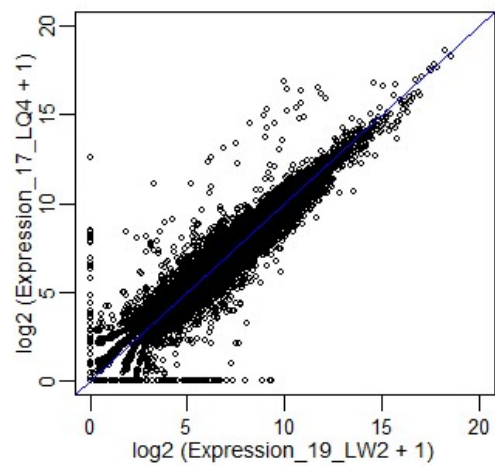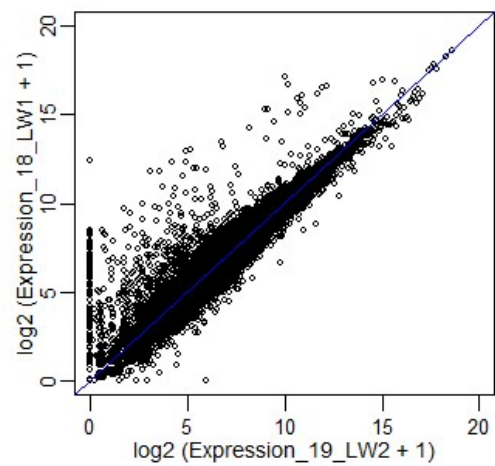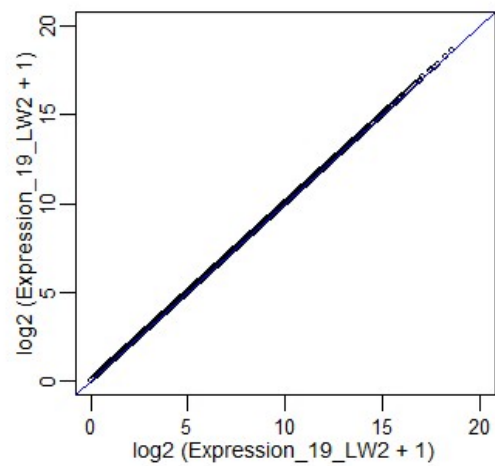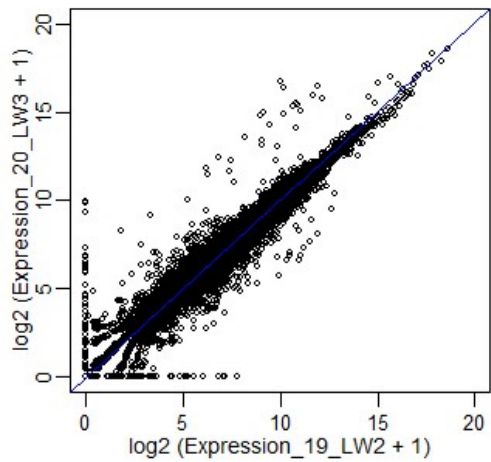



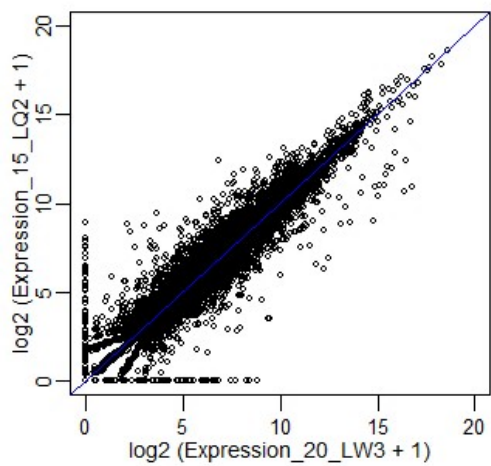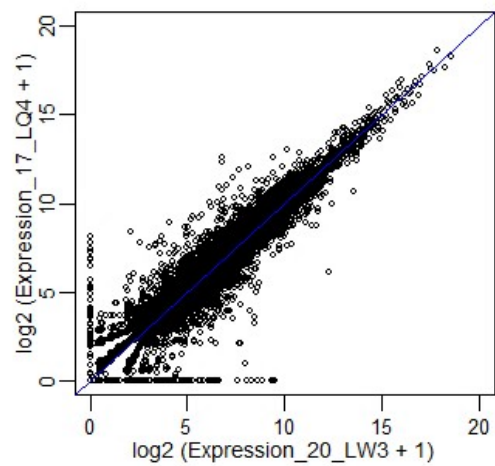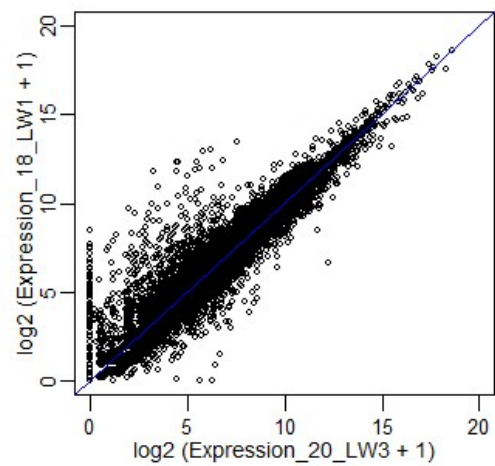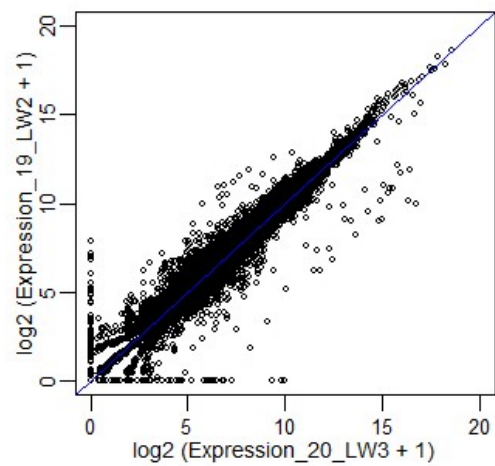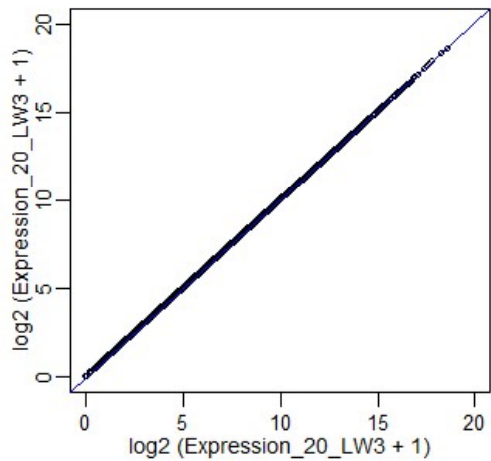

Supplement: Supplementary file 2 — Figure S7 [file MEC-30-718-s002.pdf]

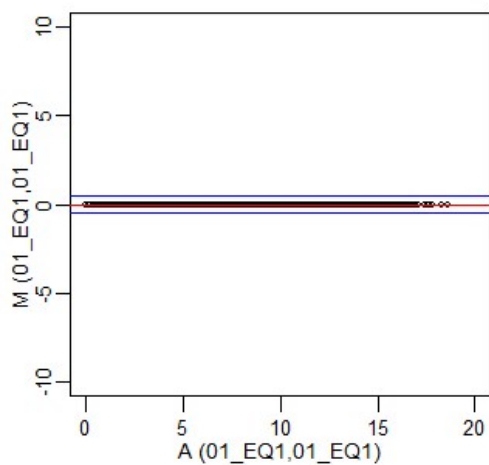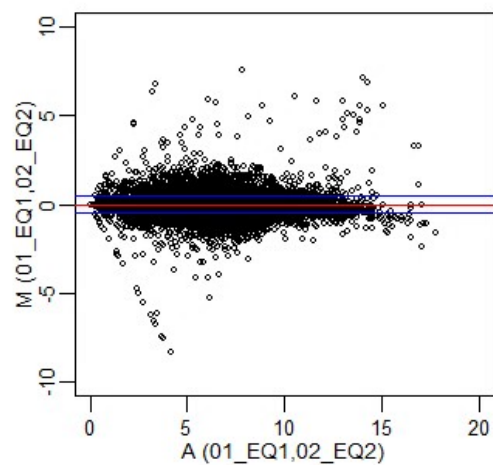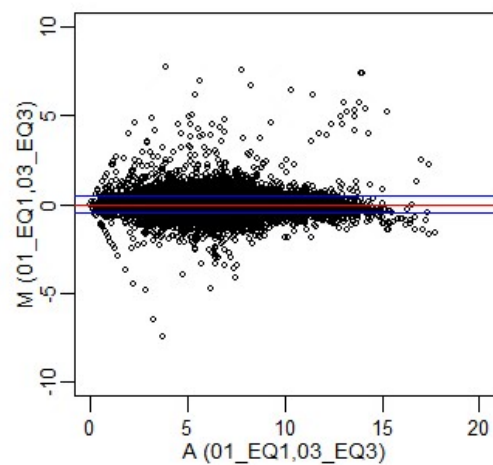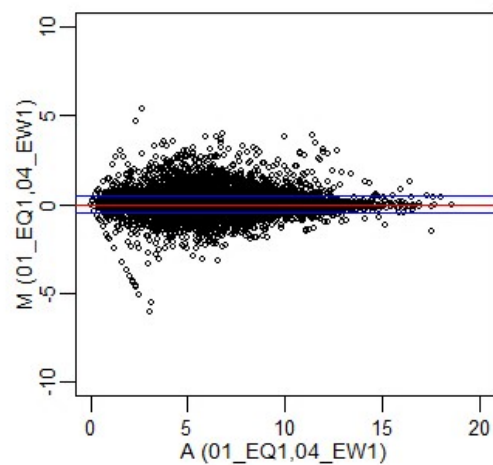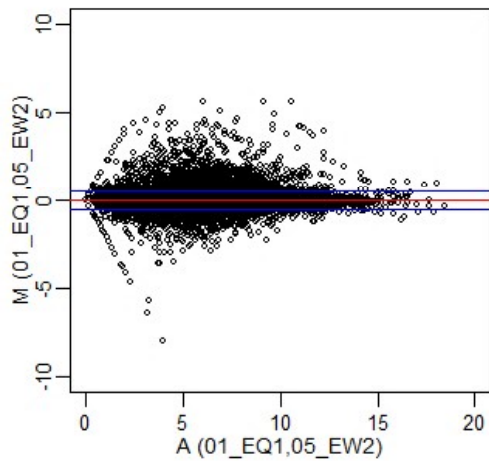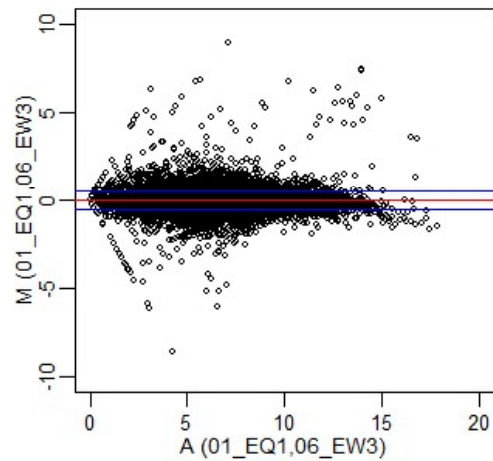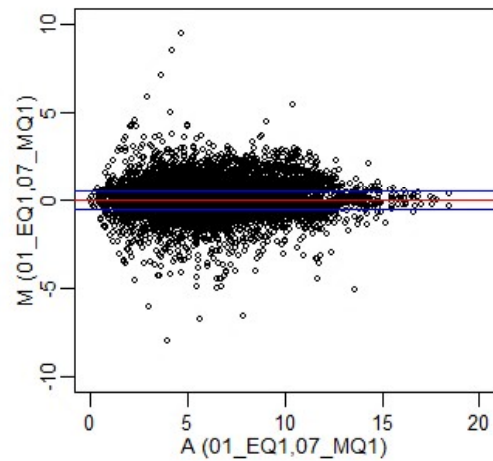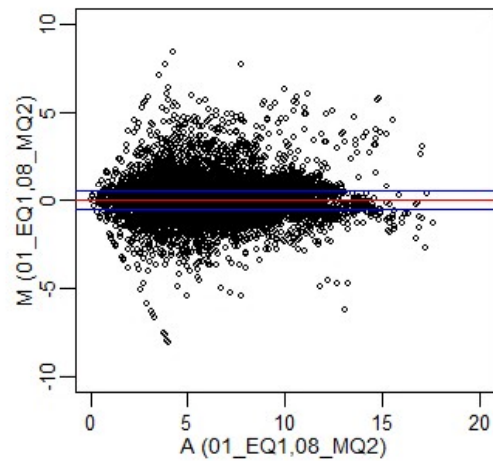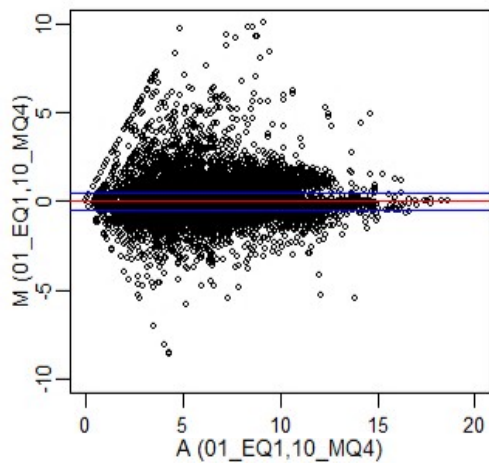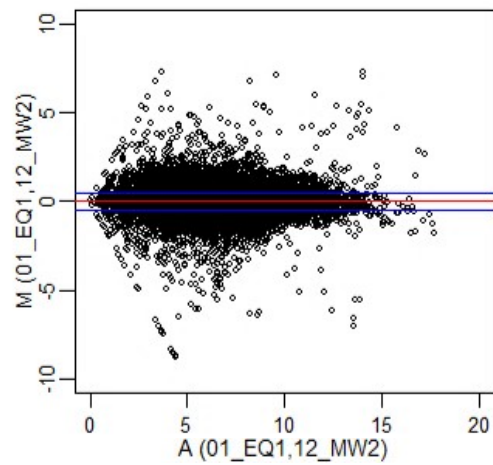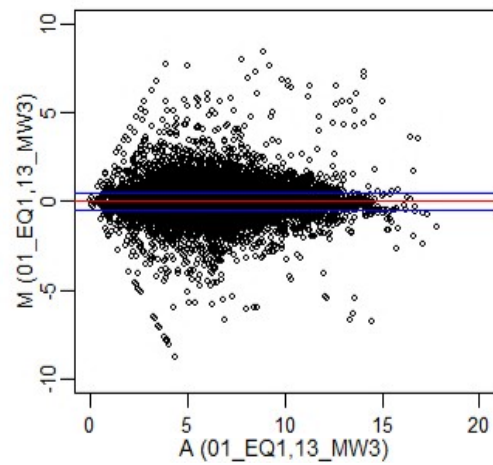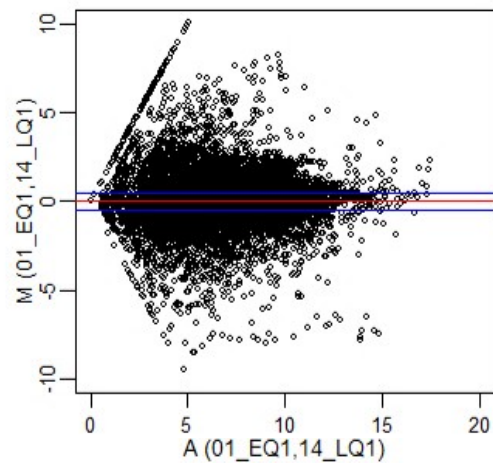

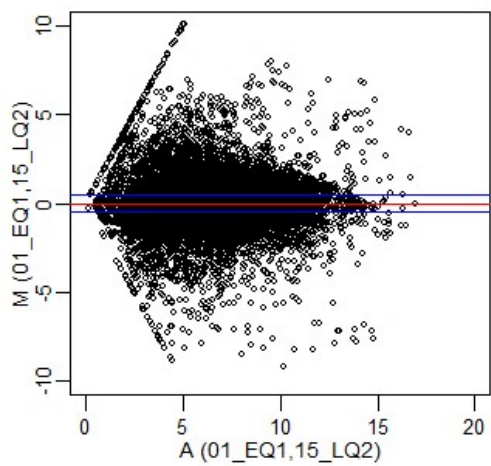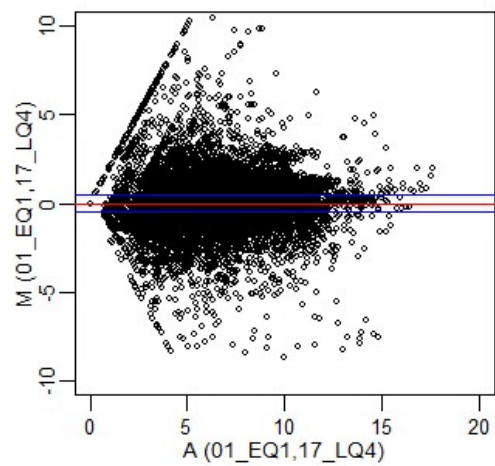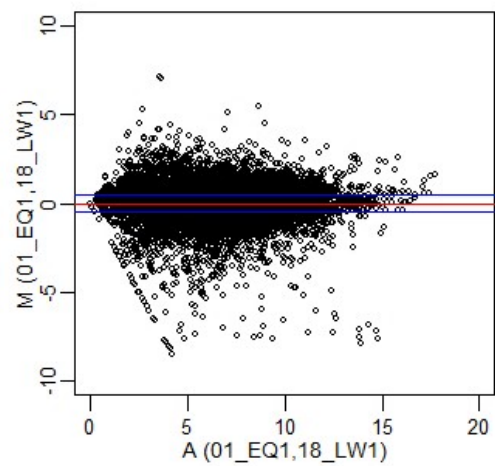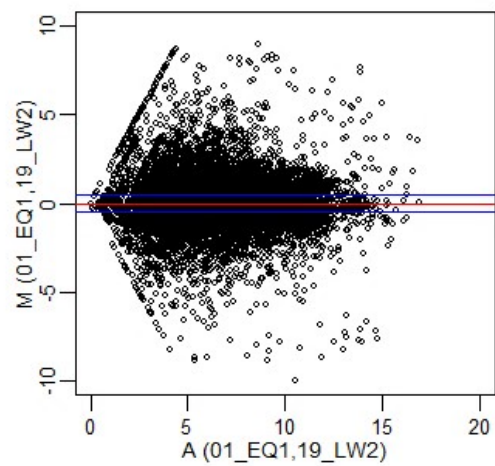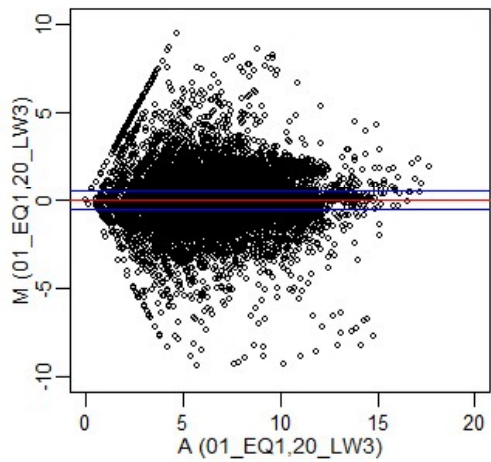

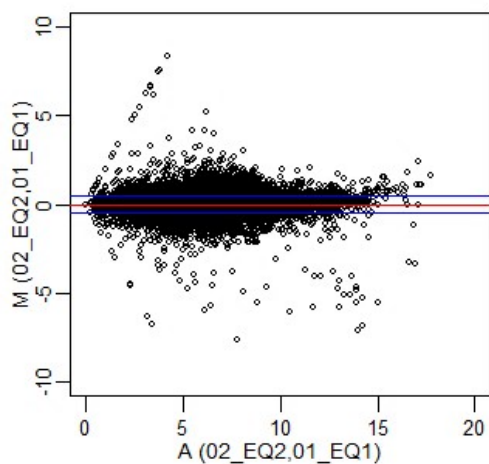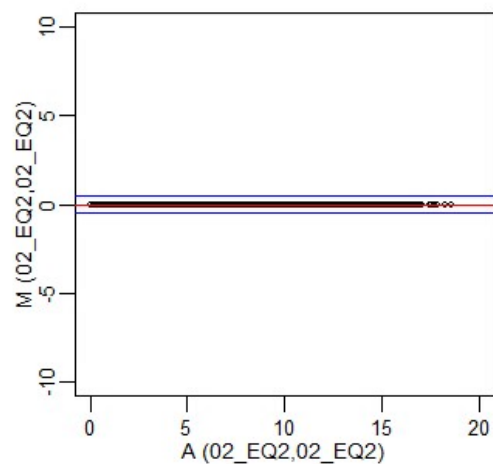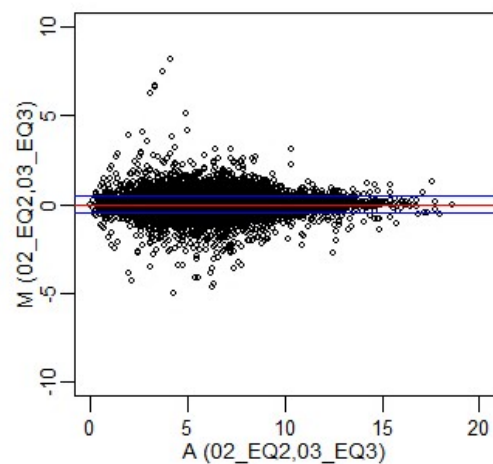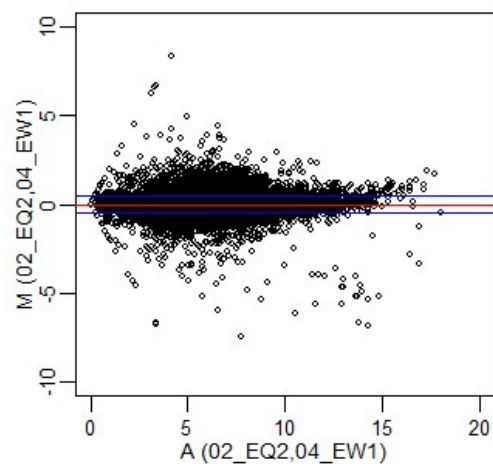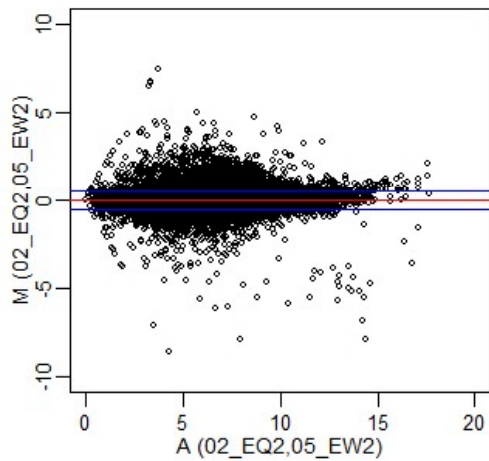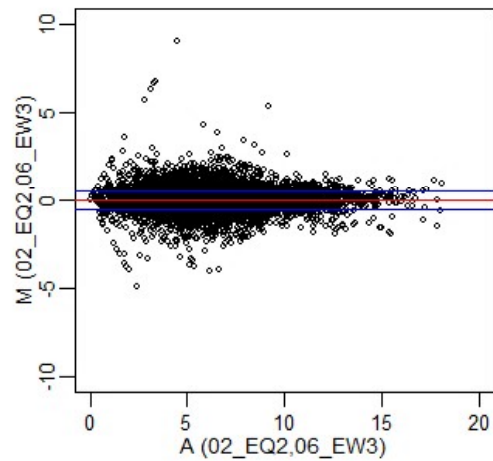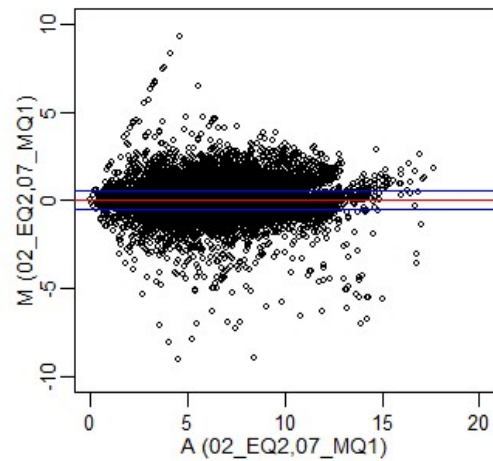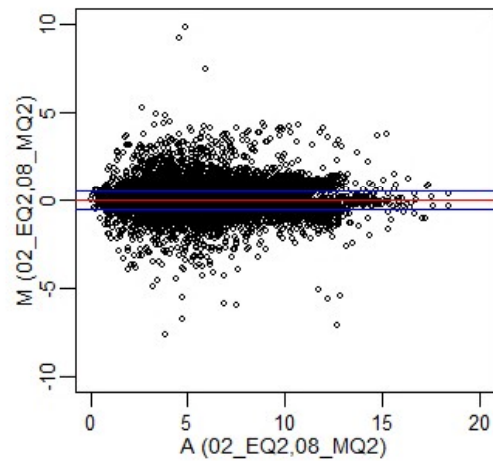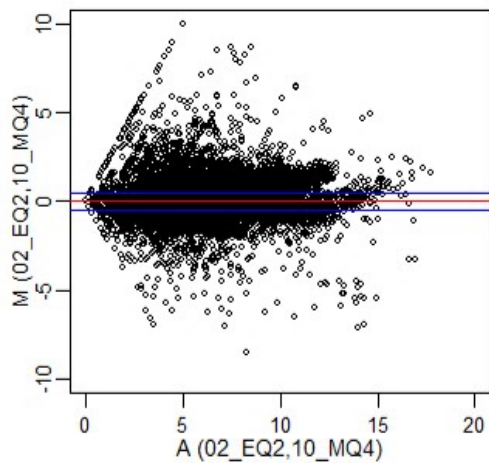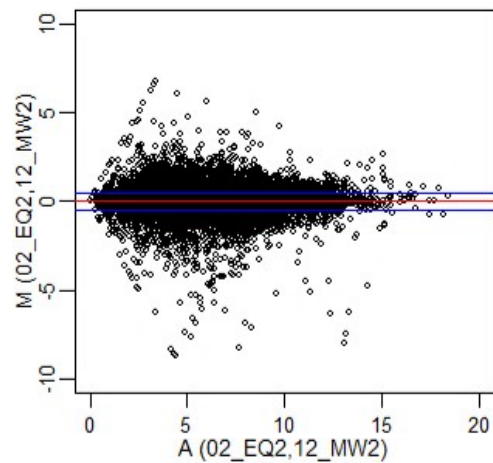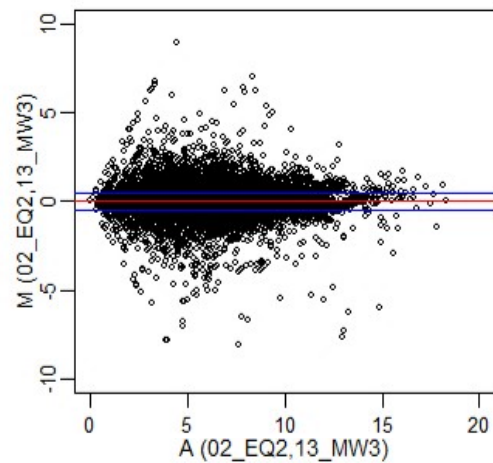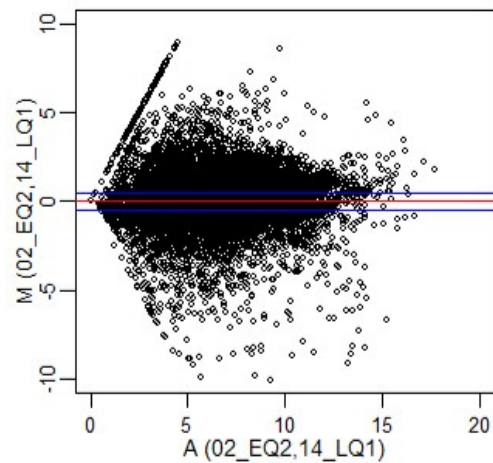

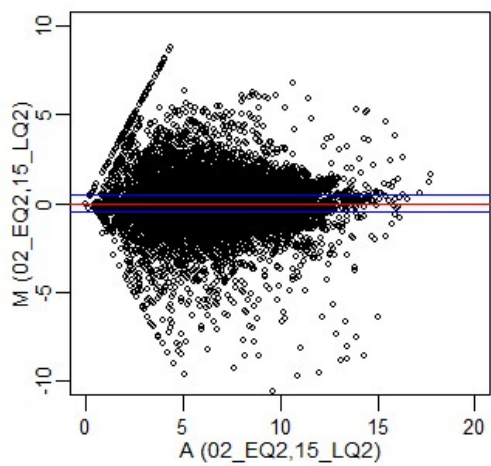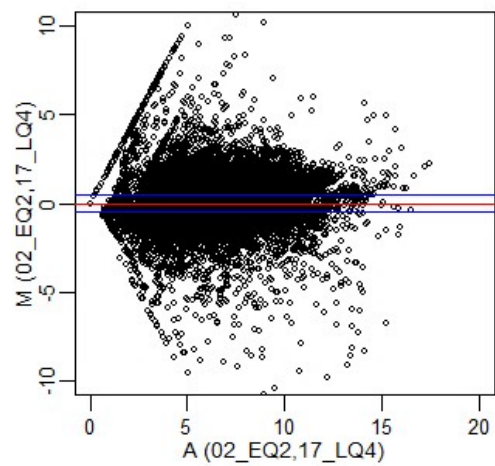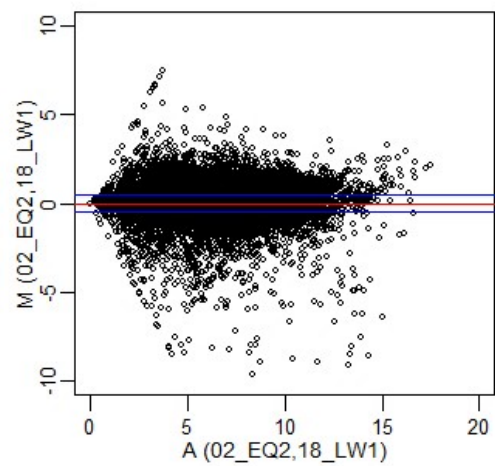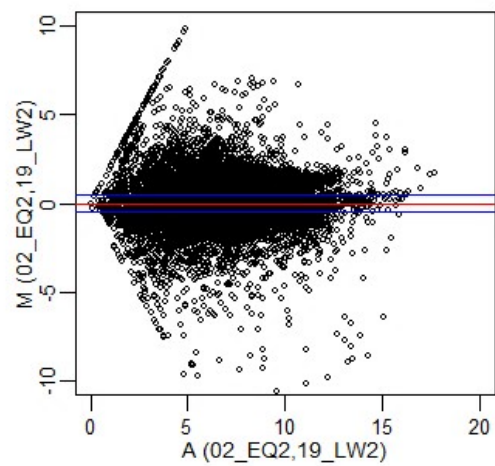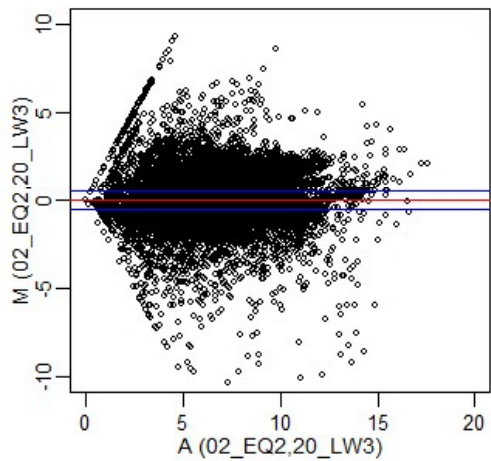



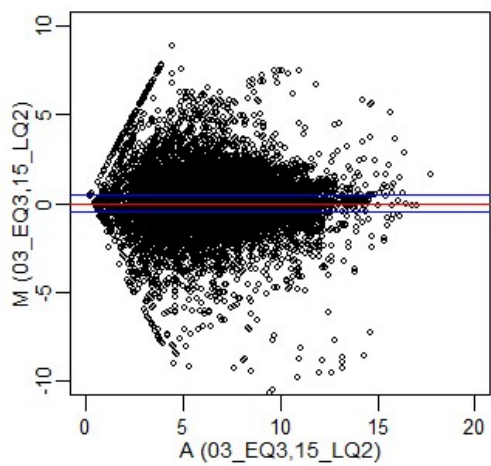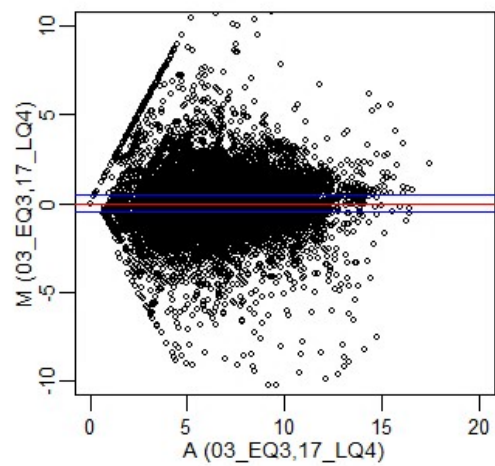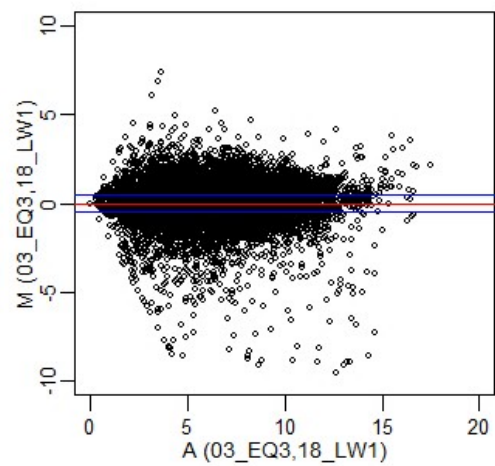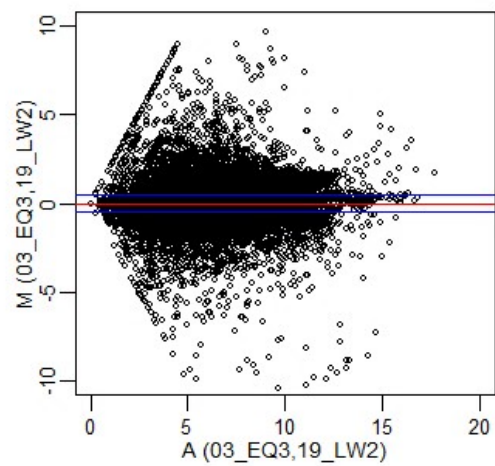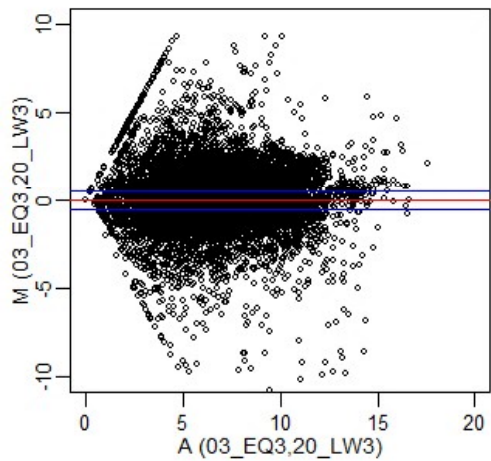



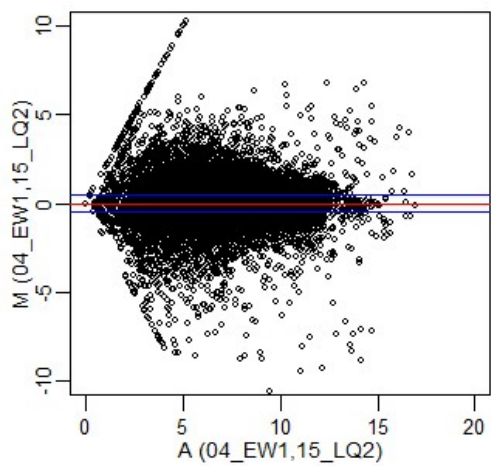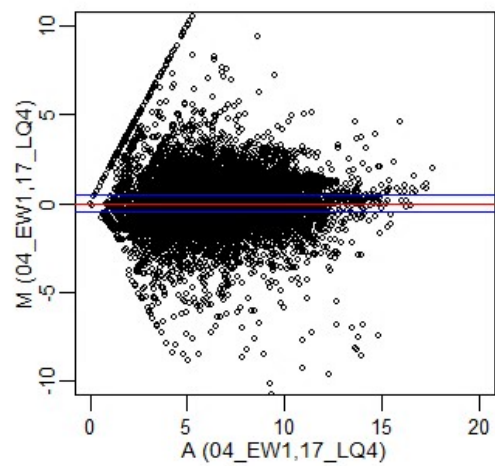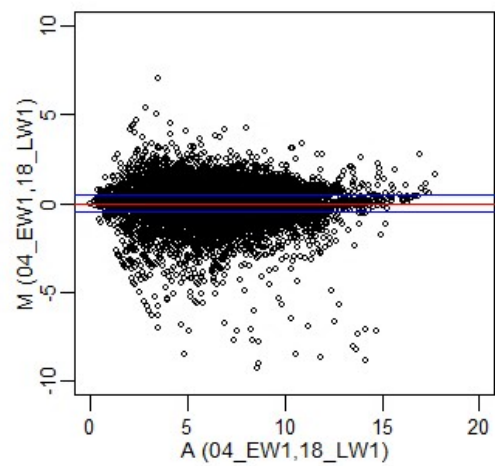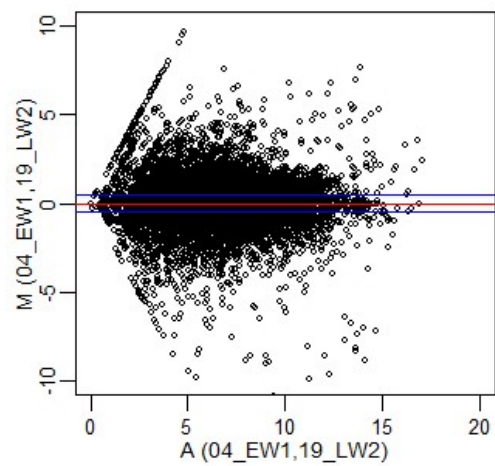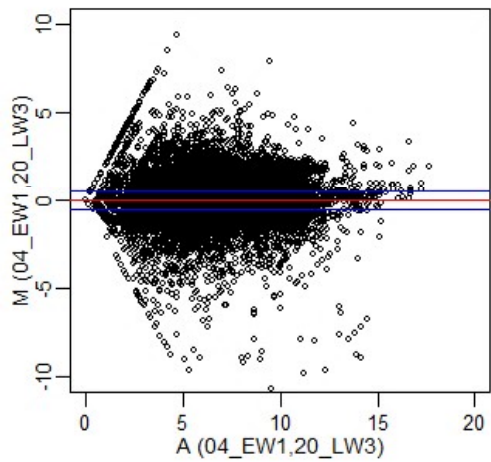



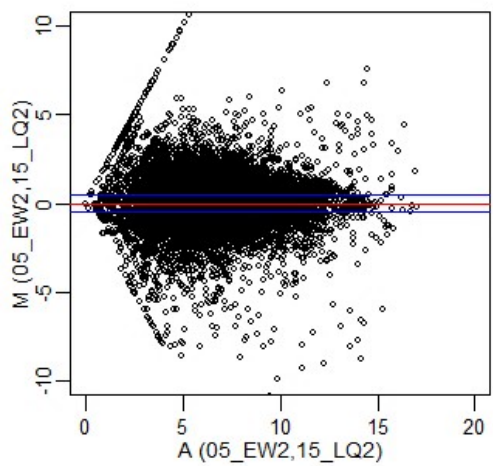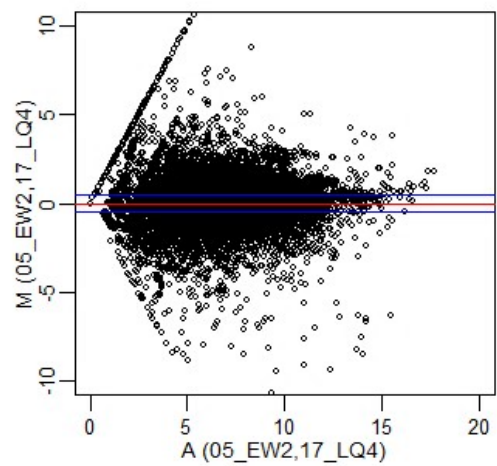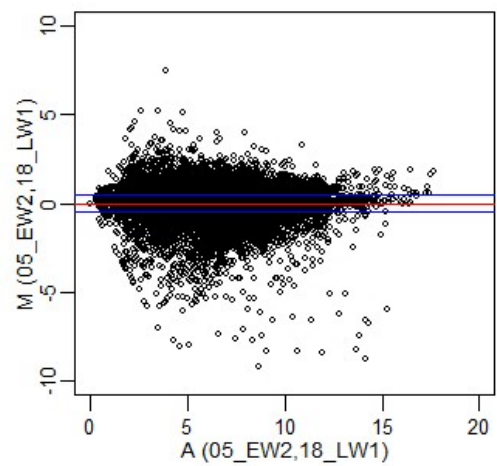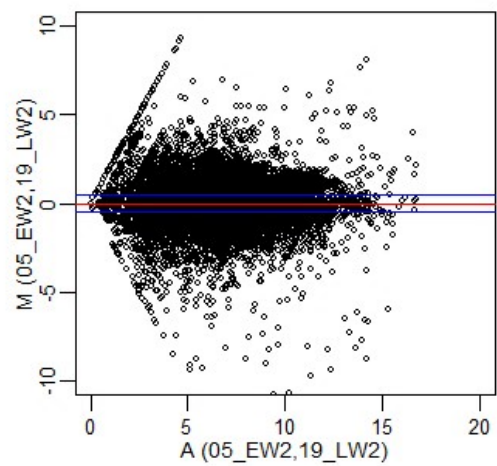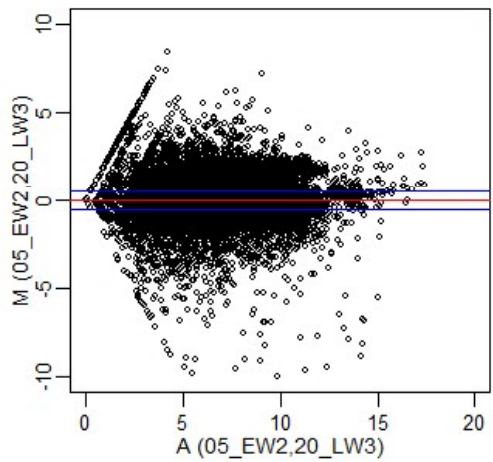



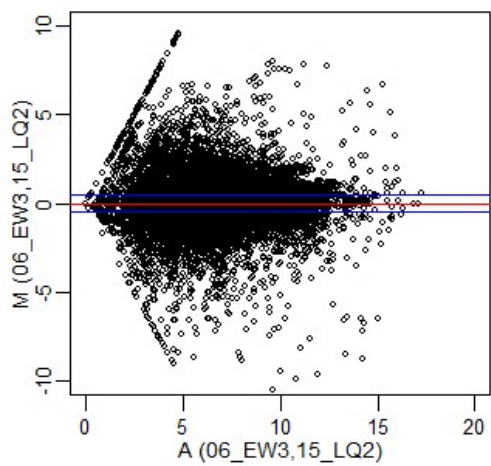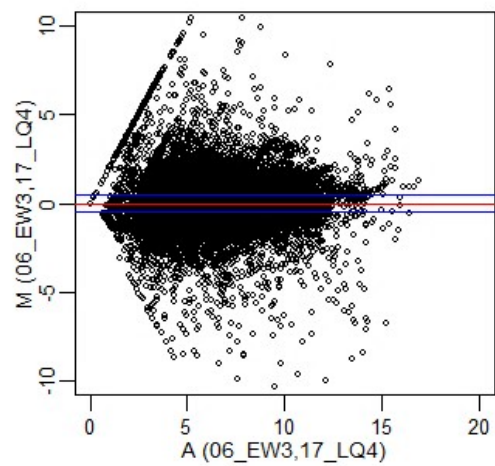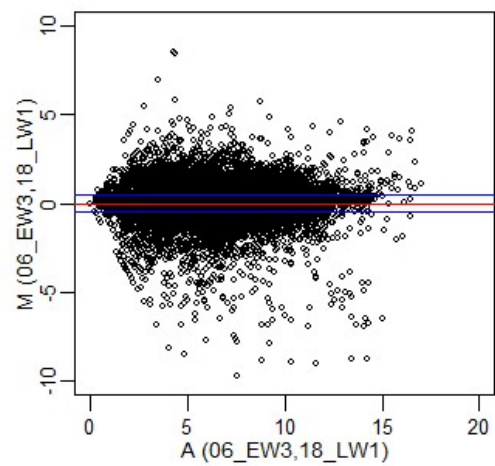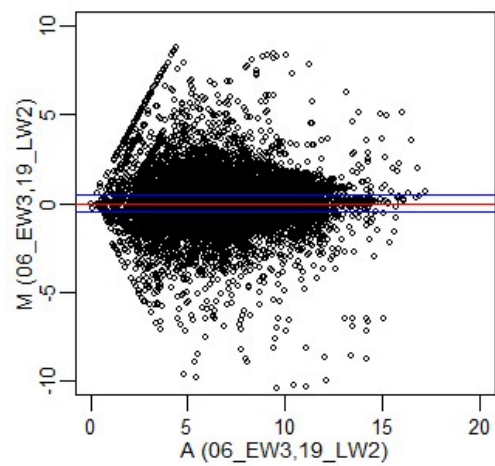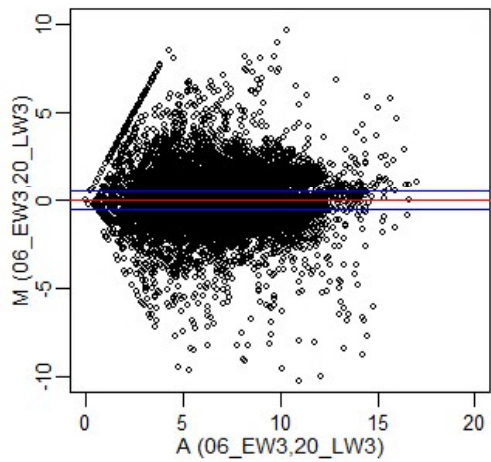



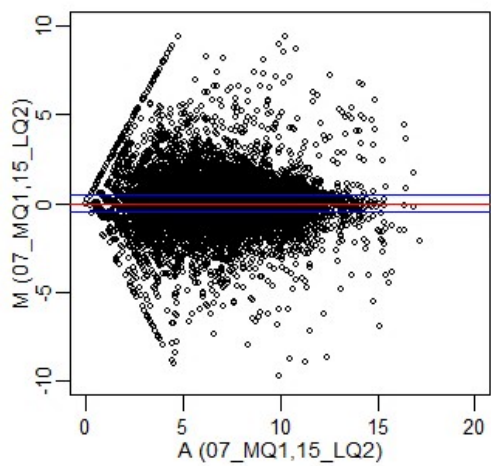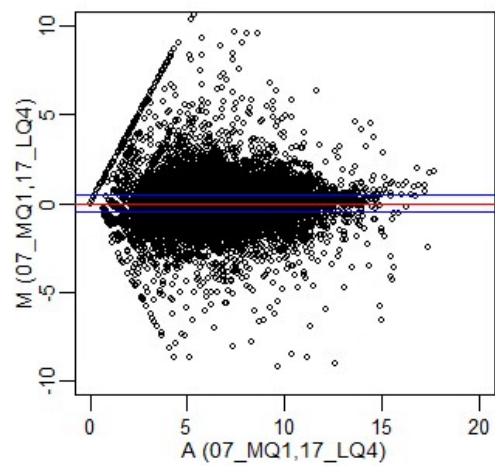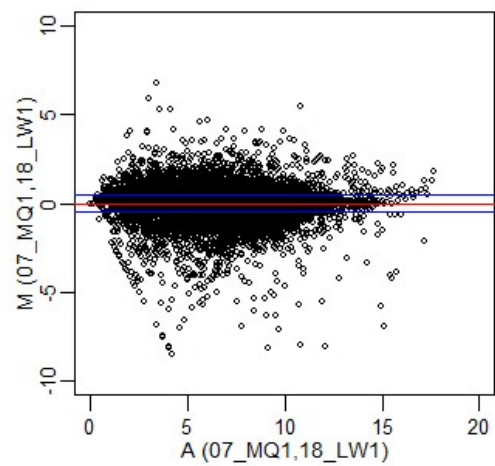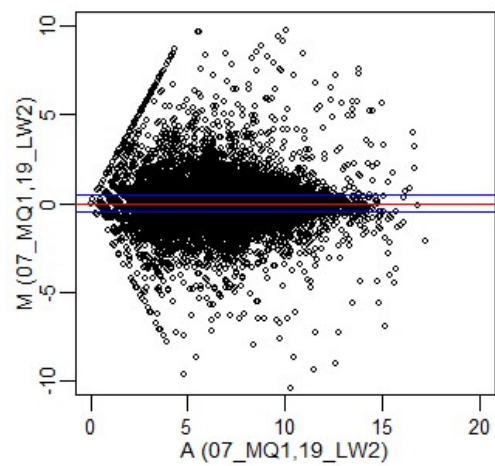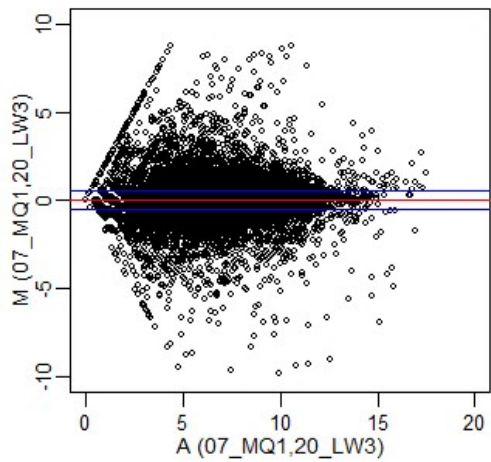

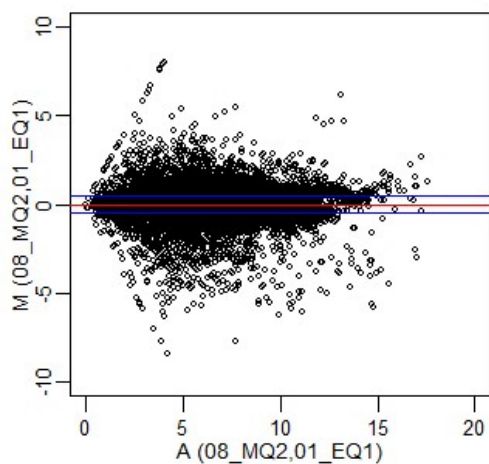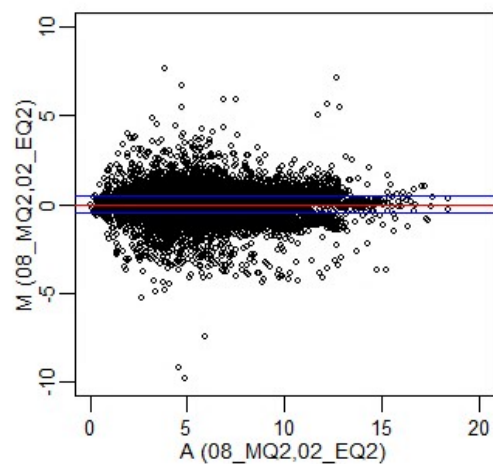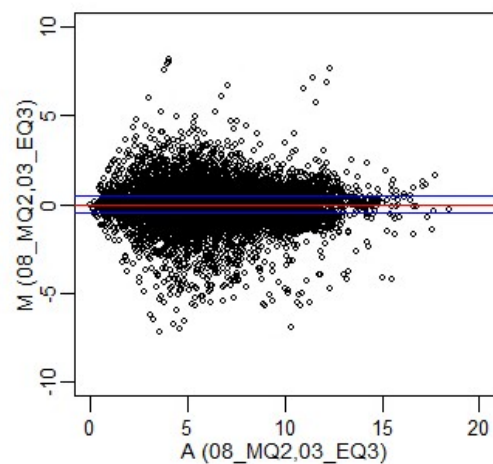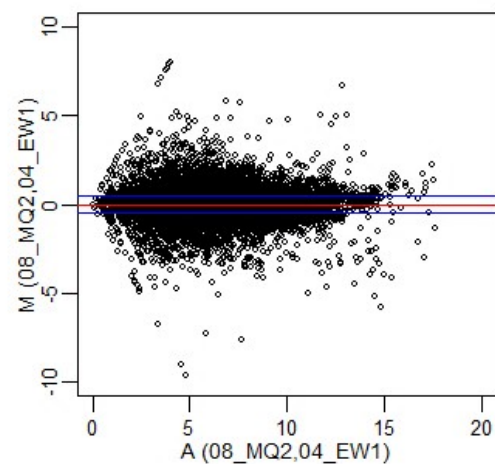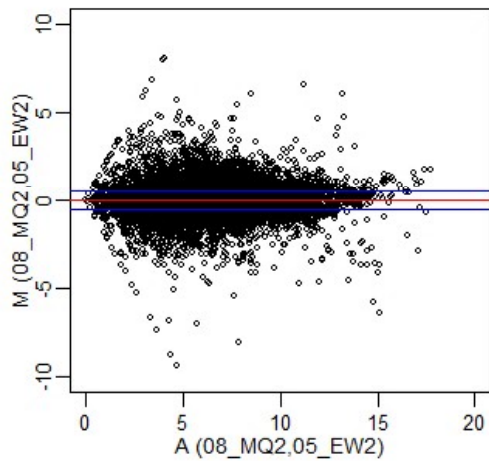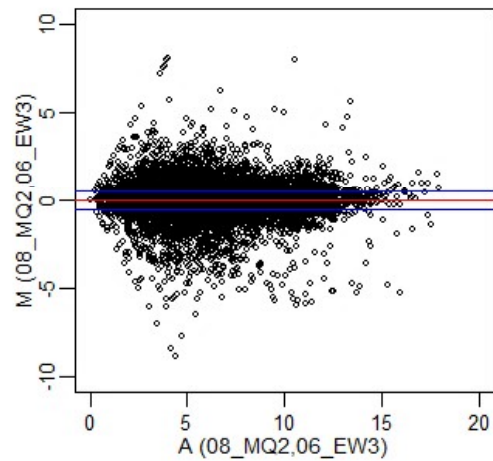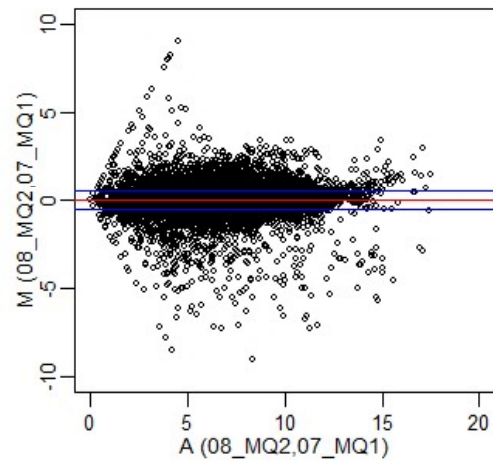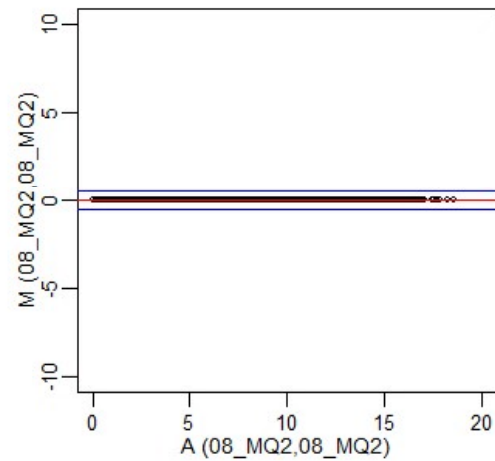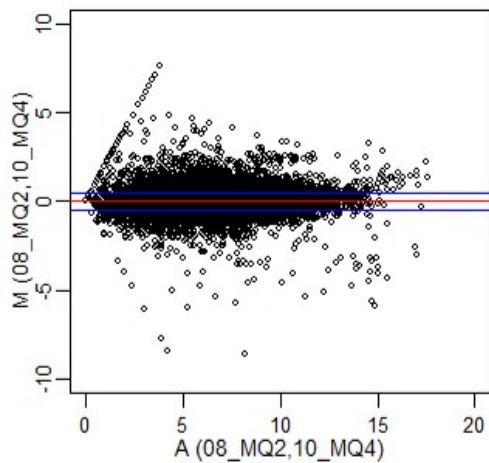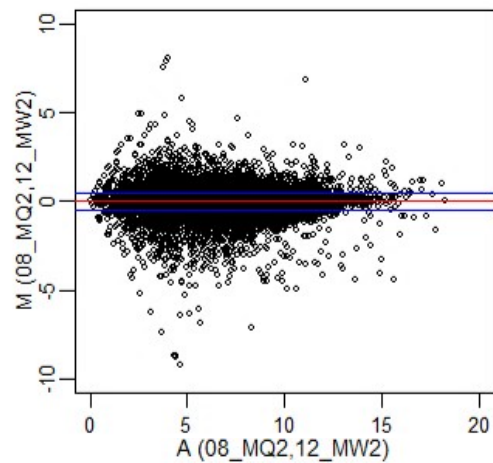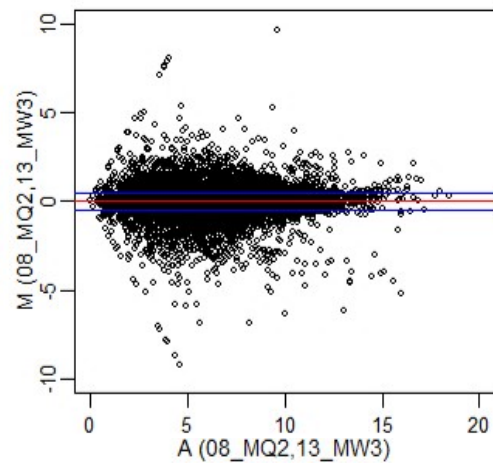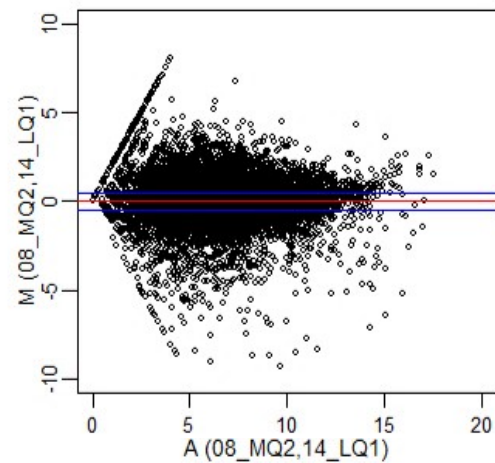

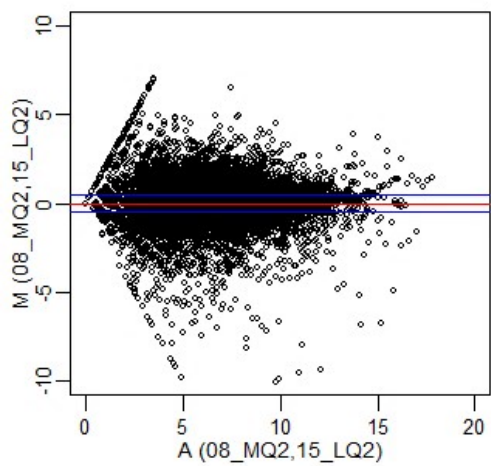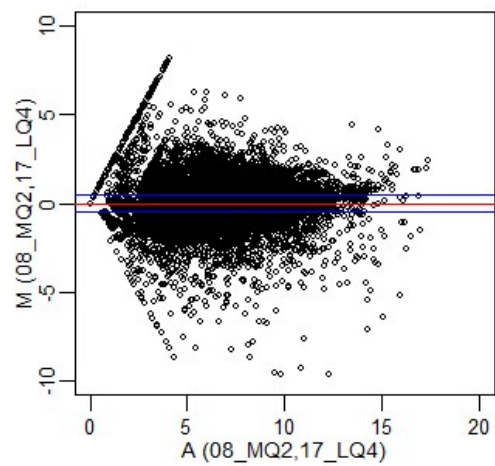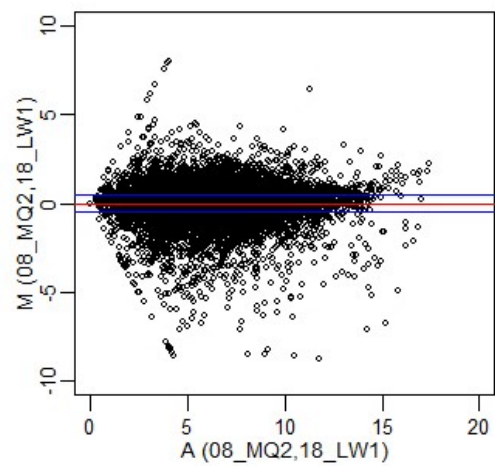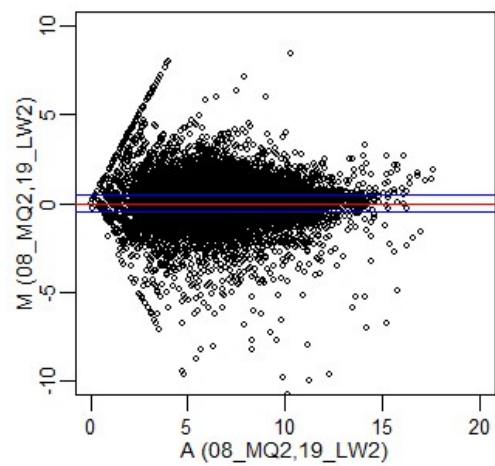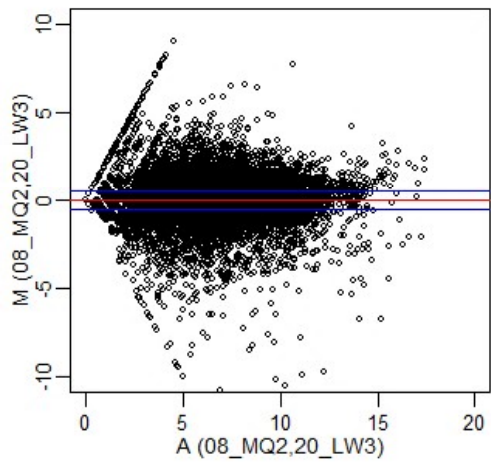



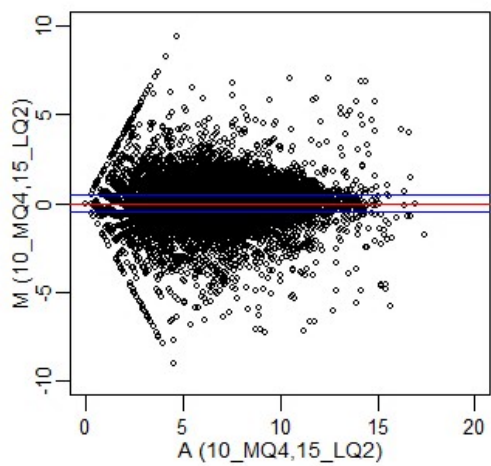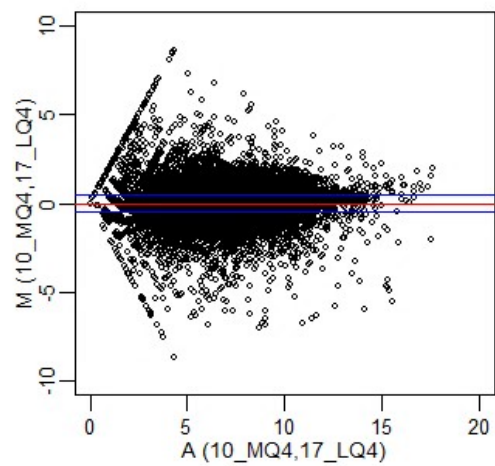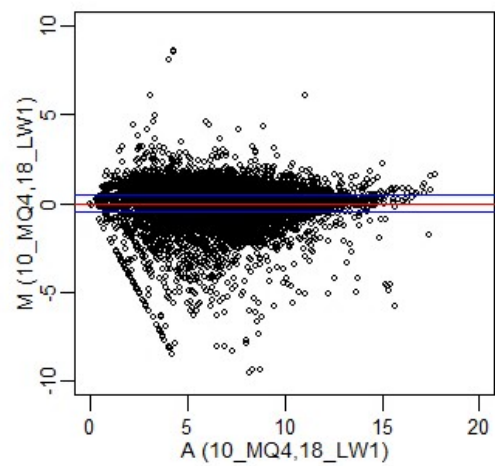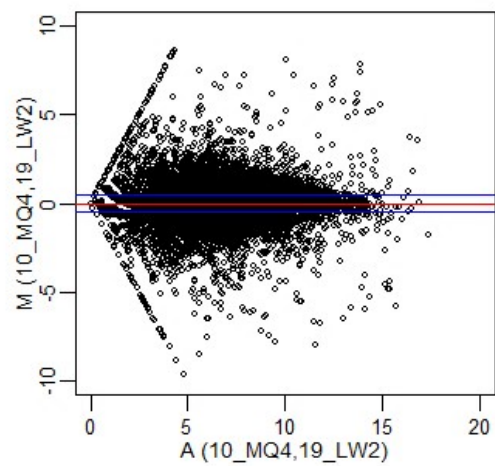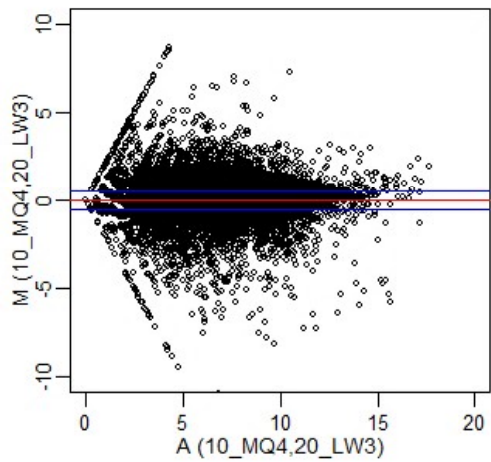

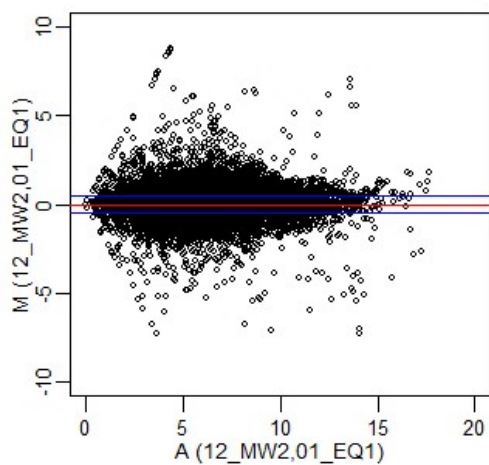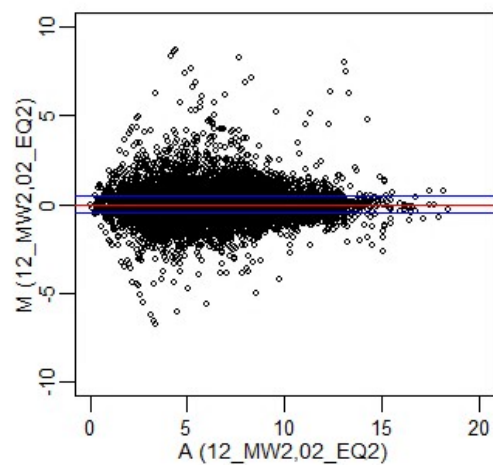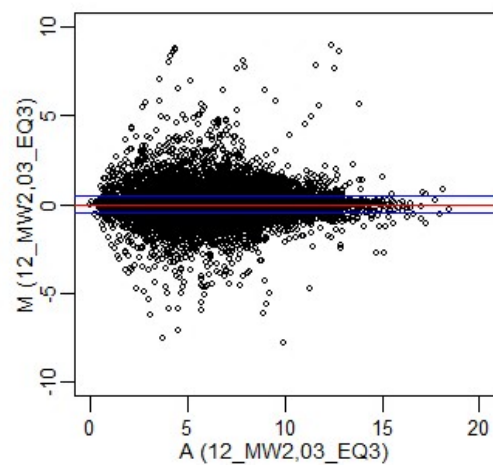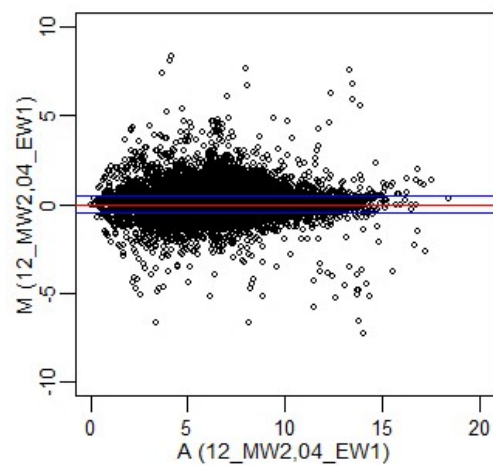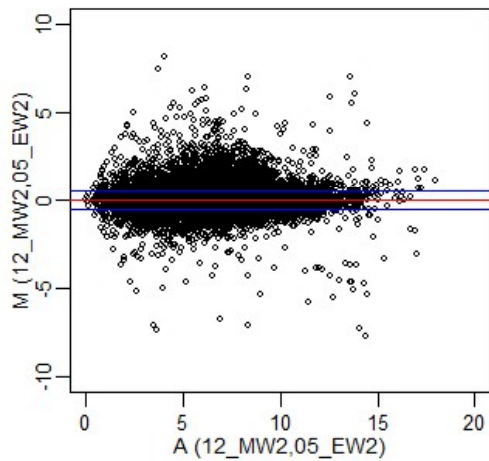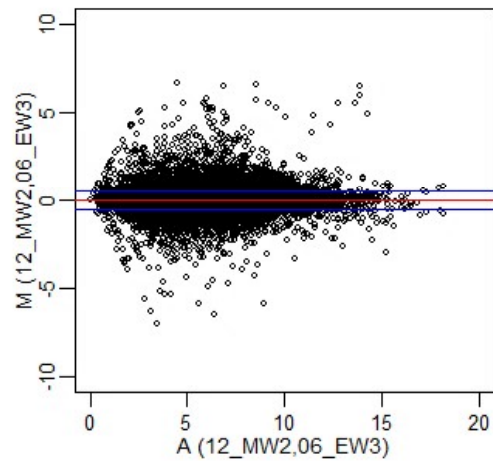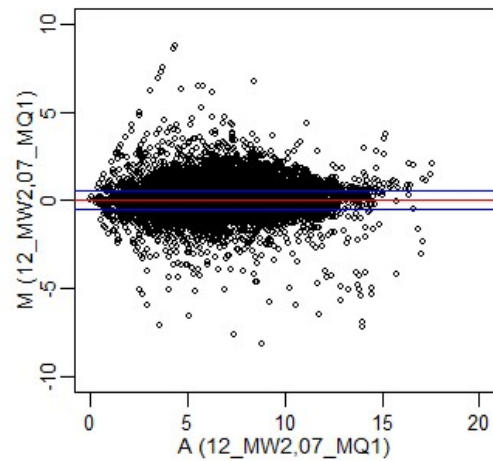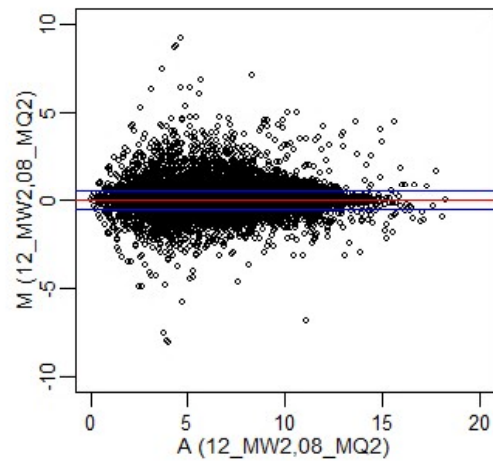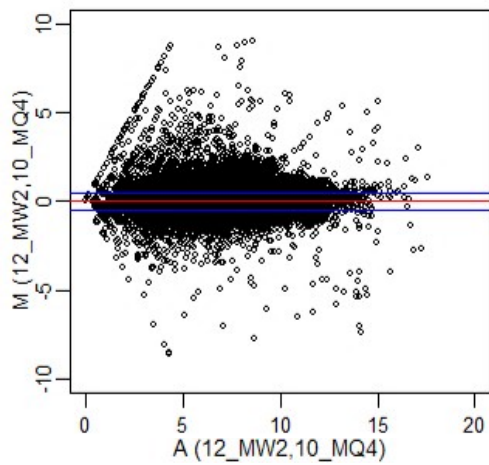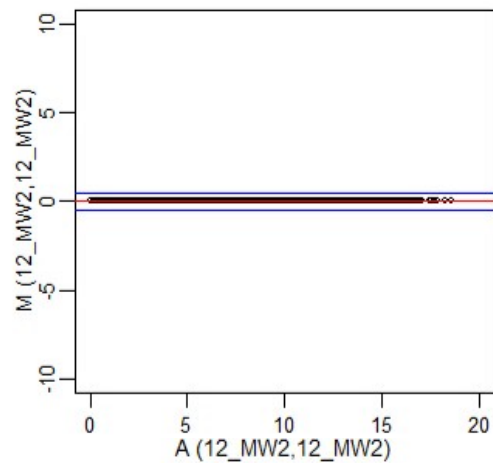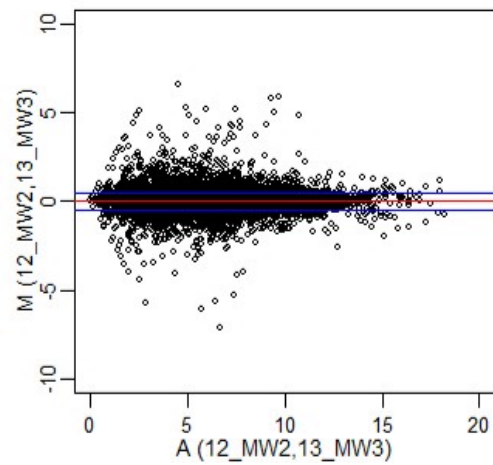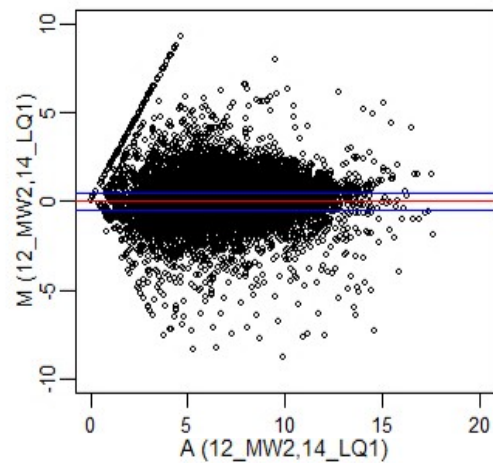

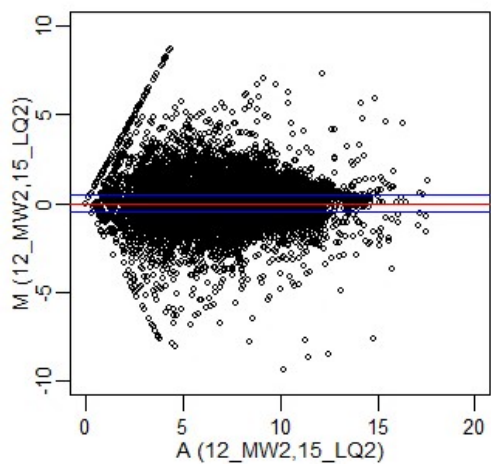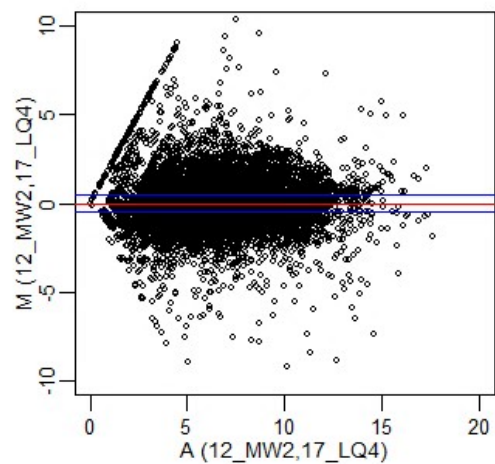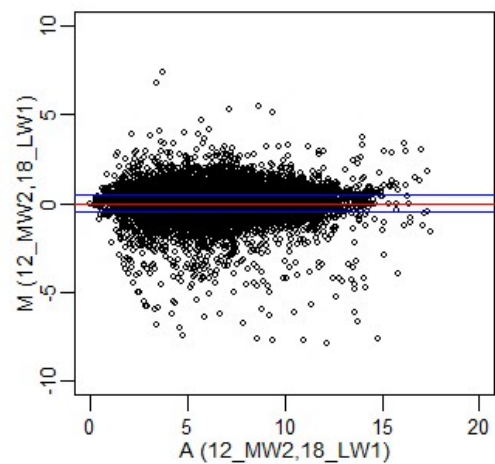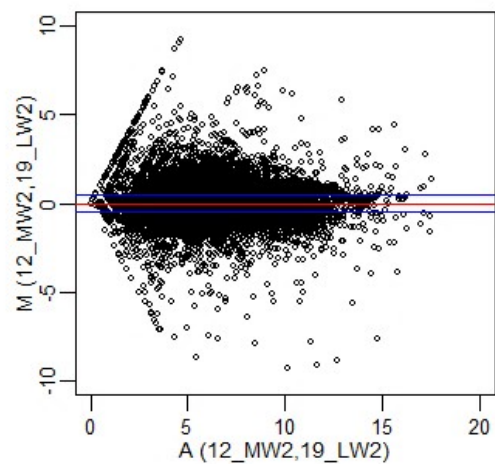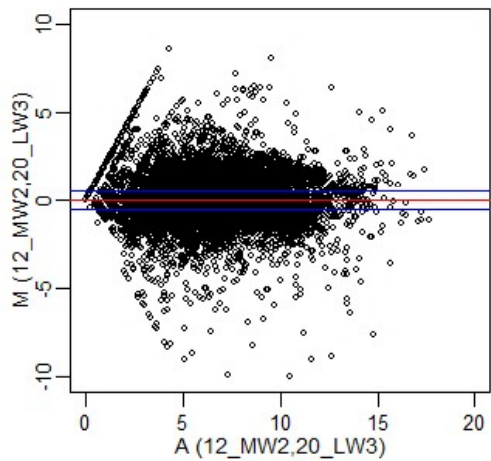

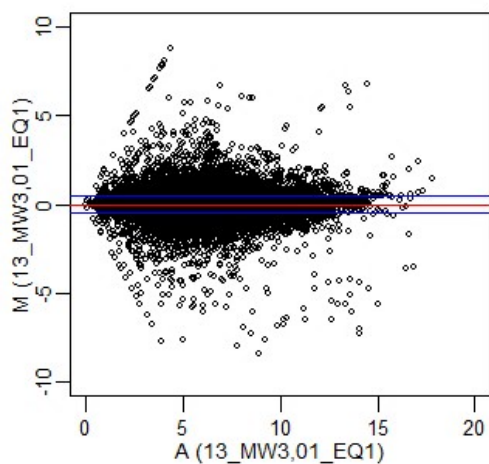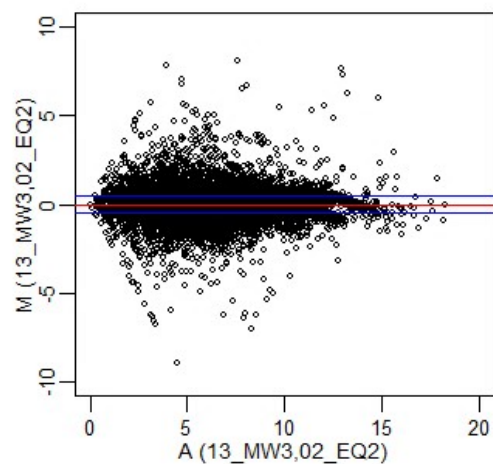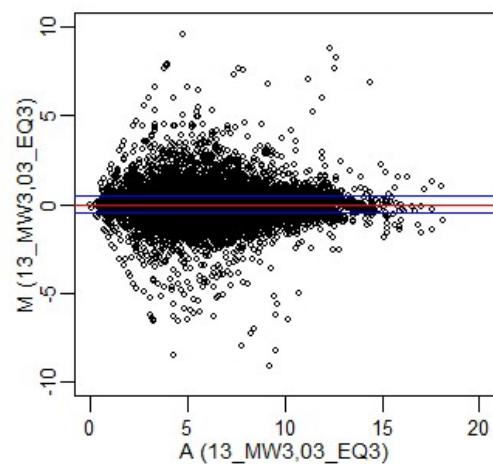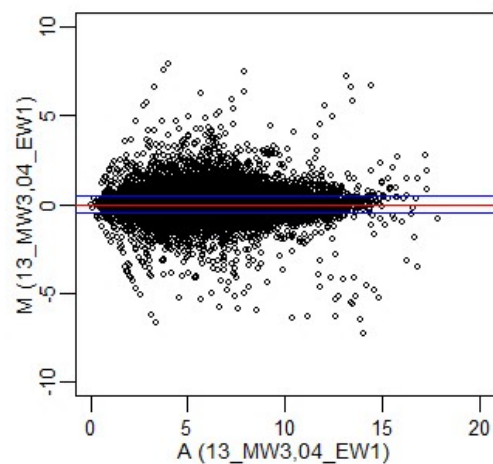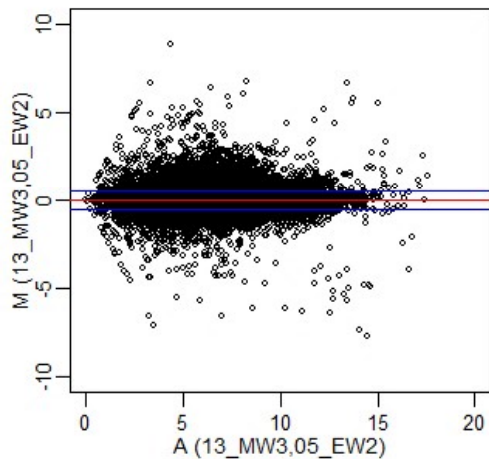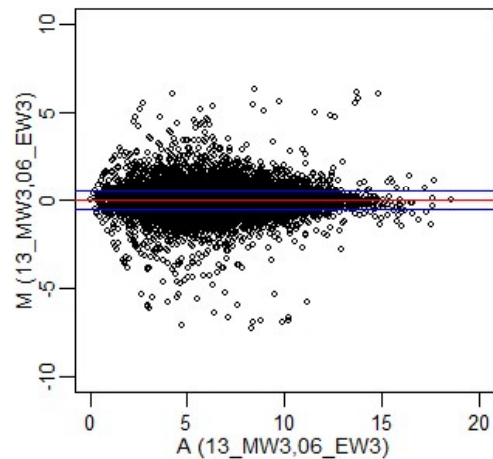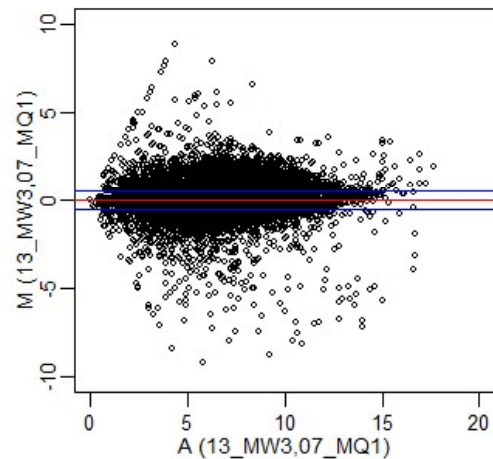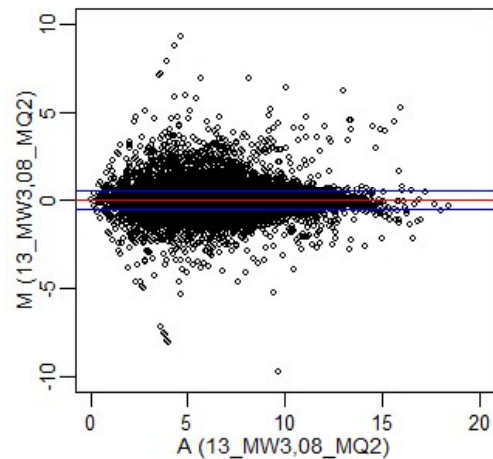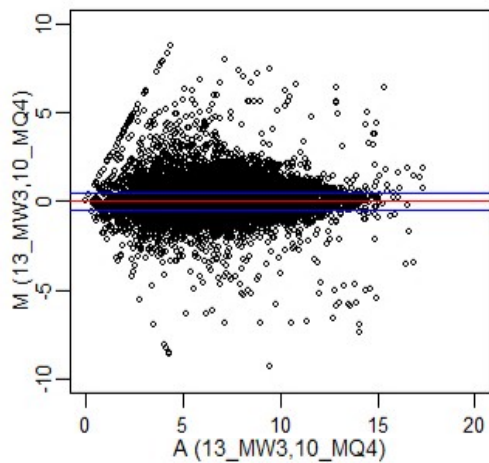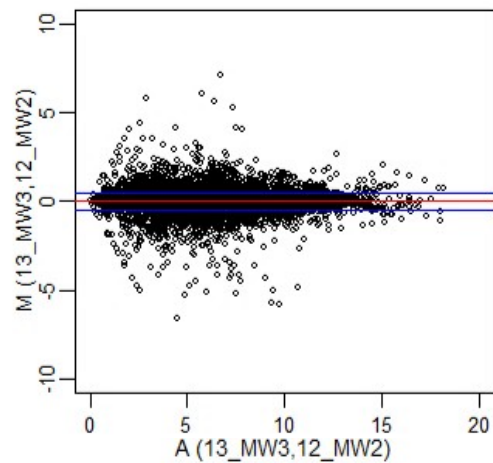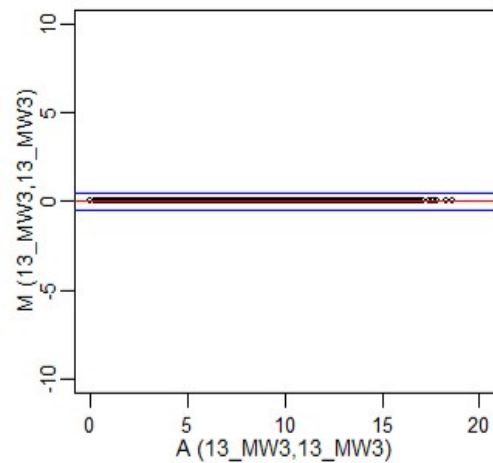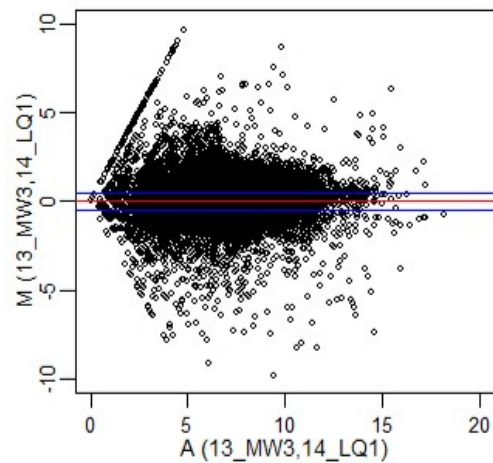

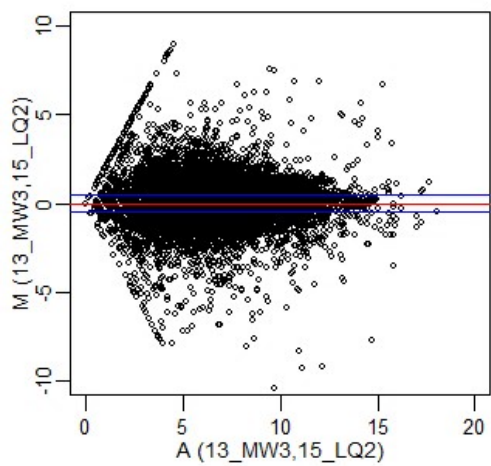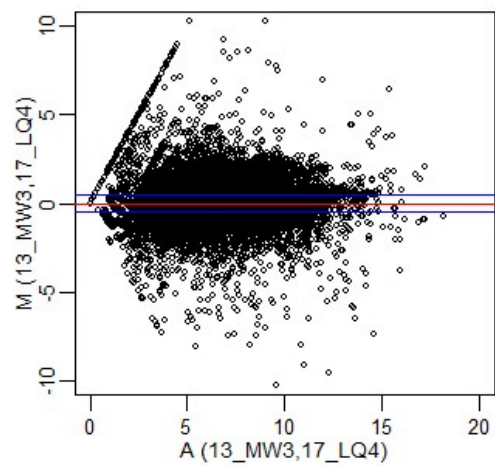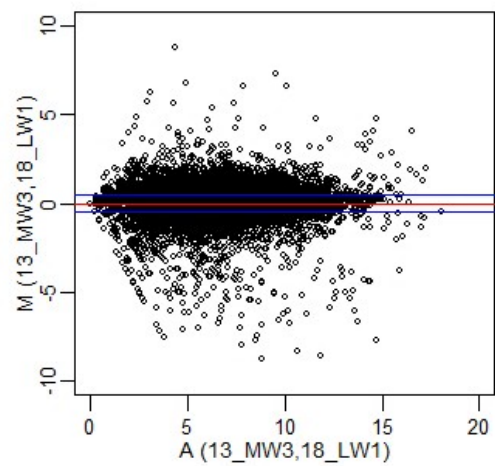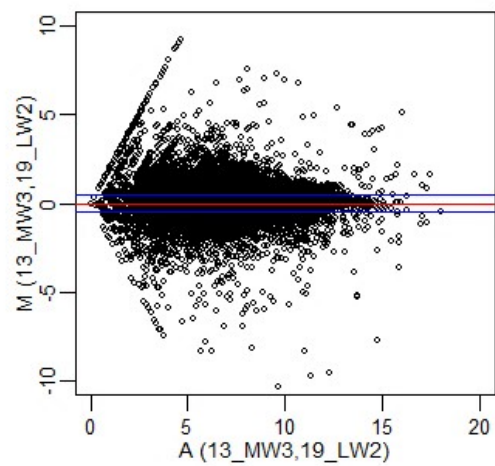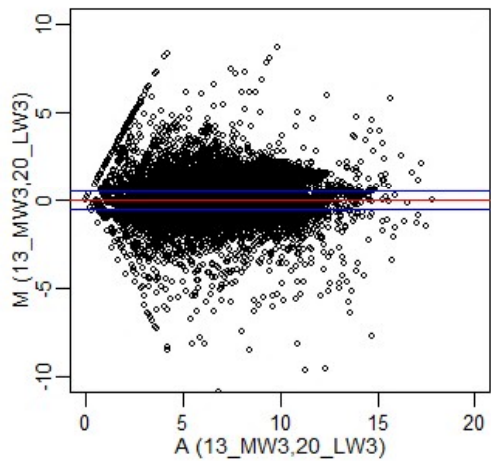



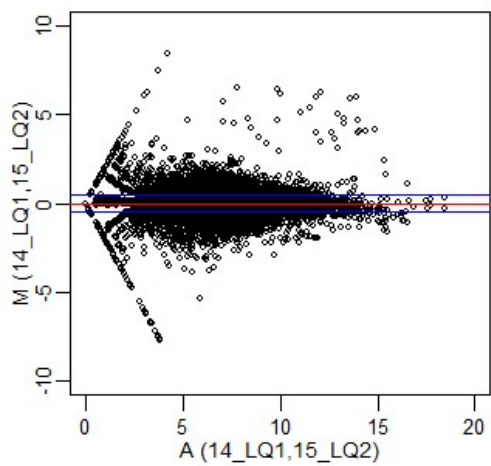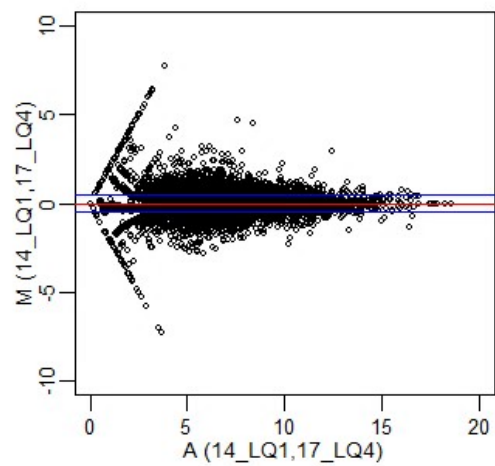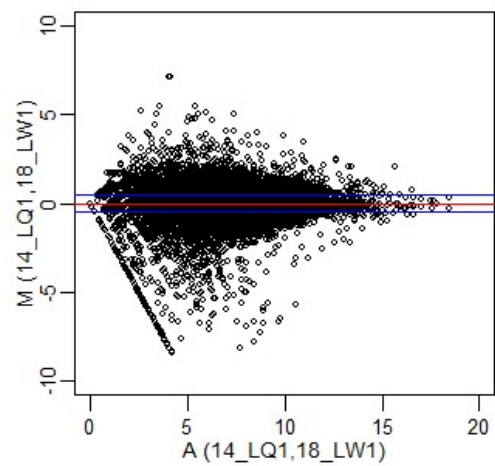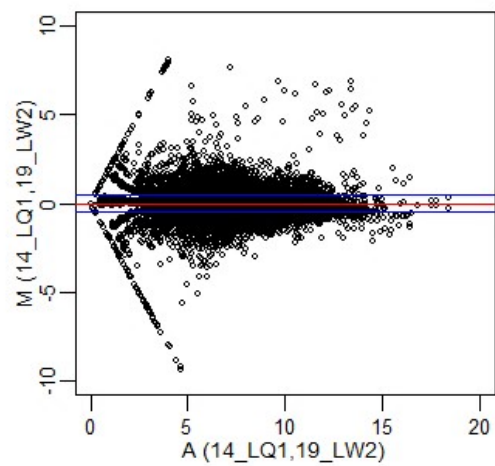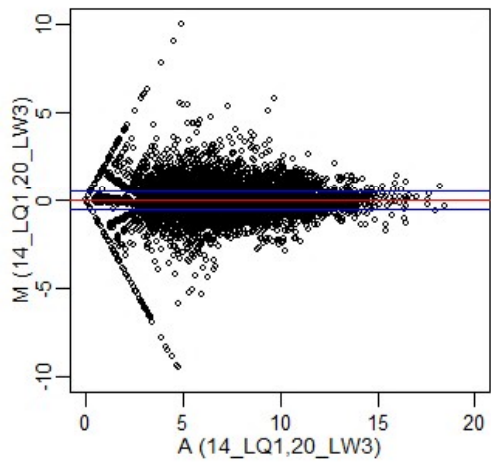



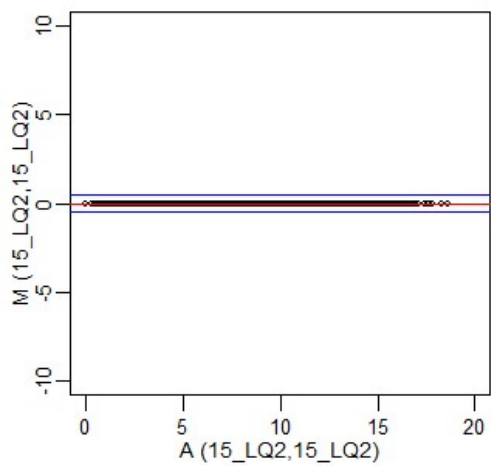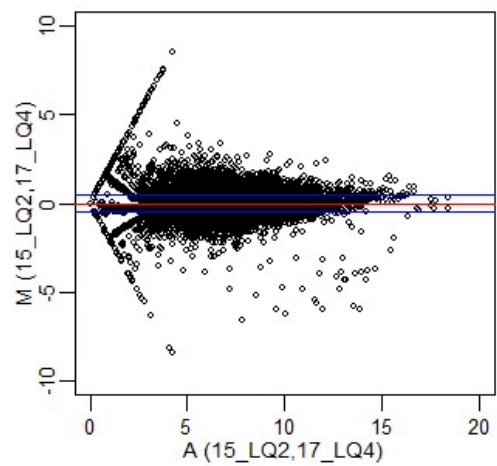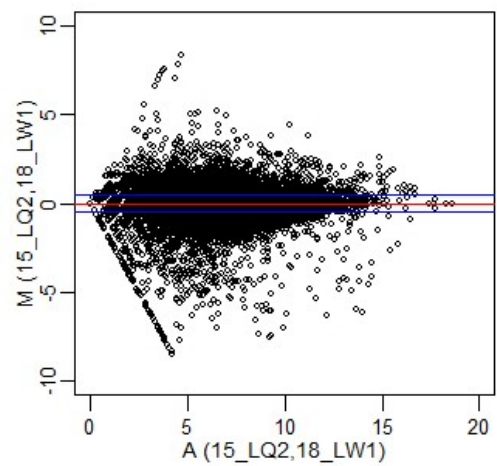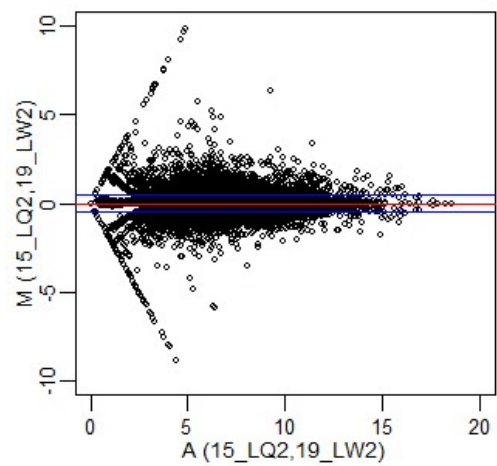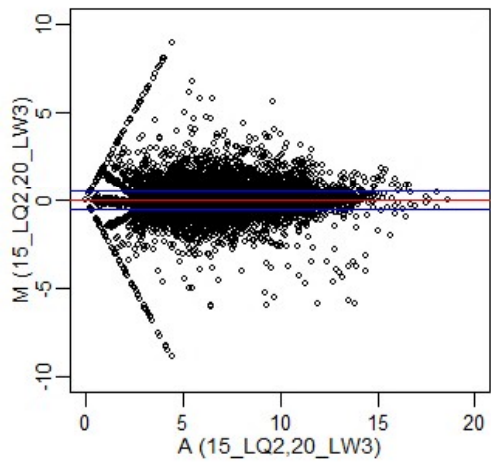



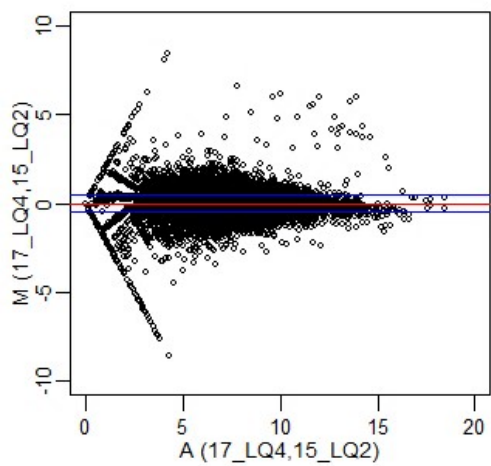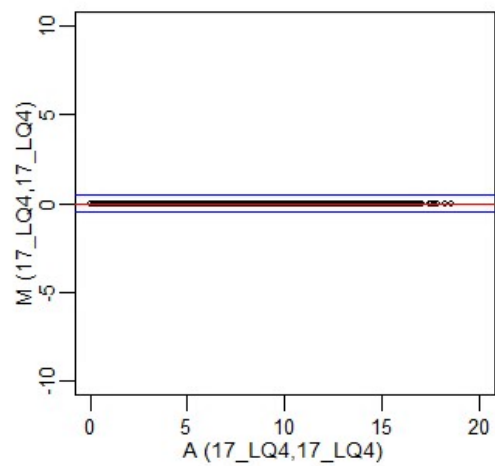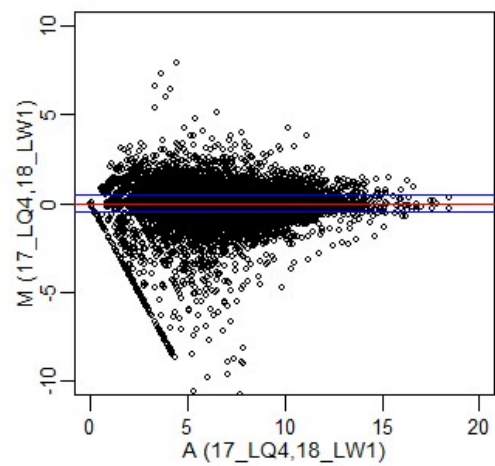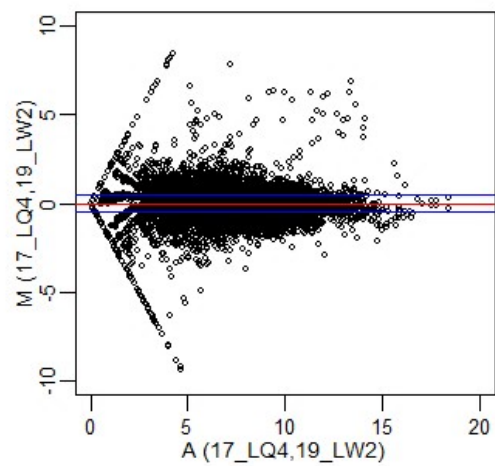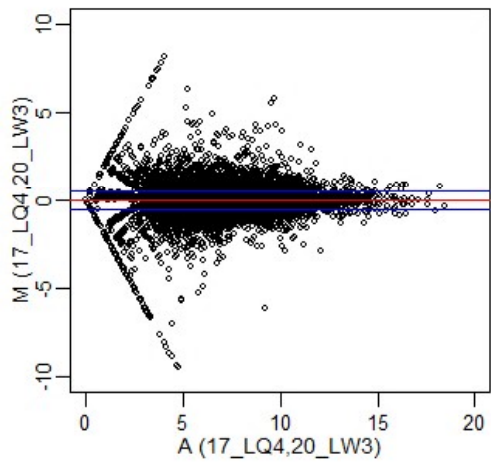

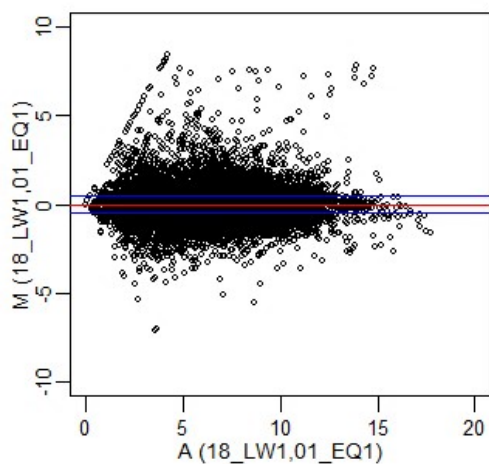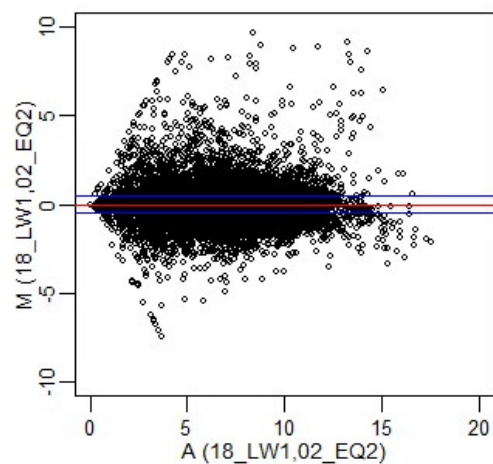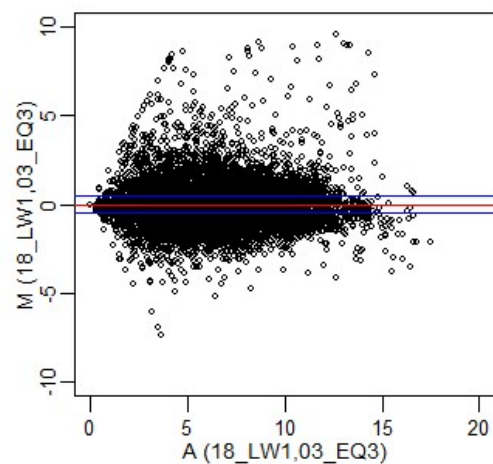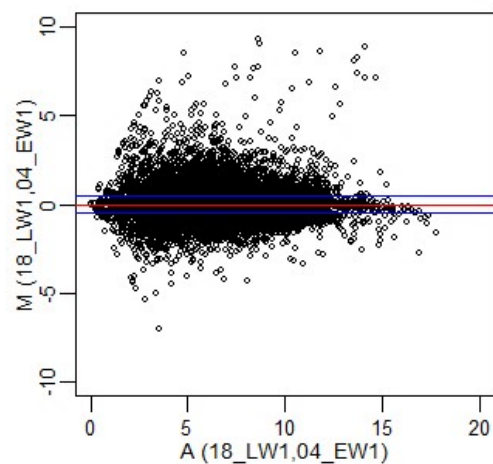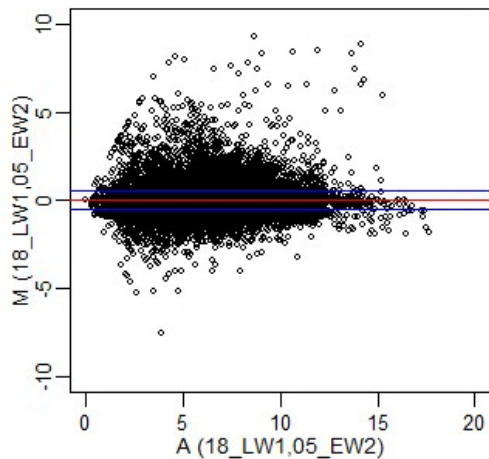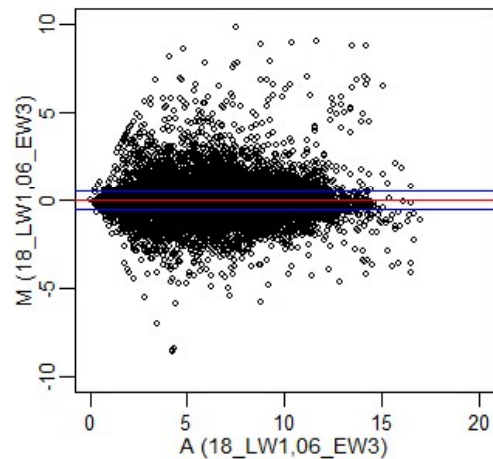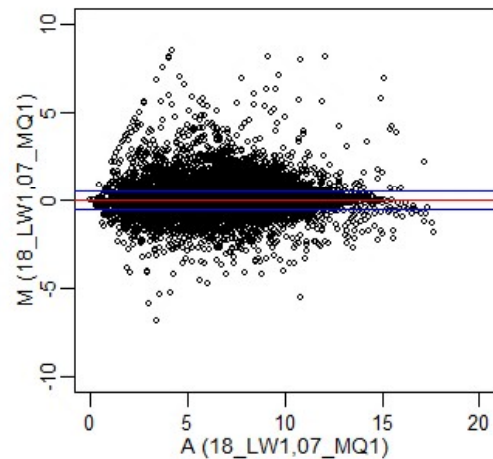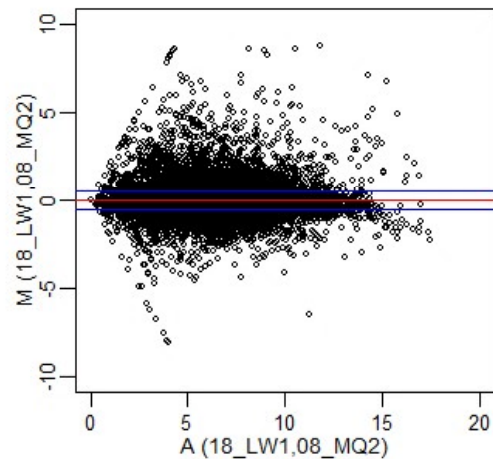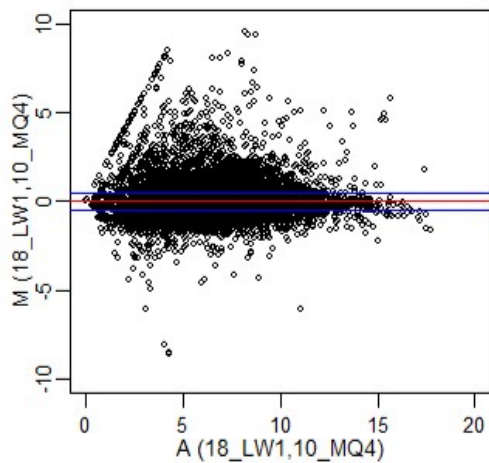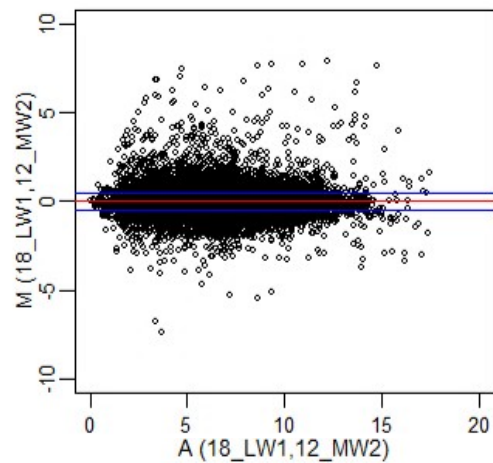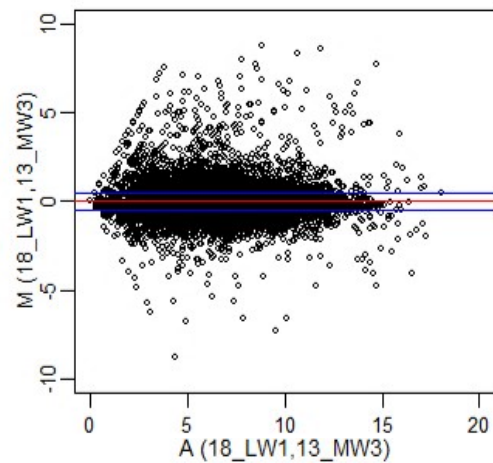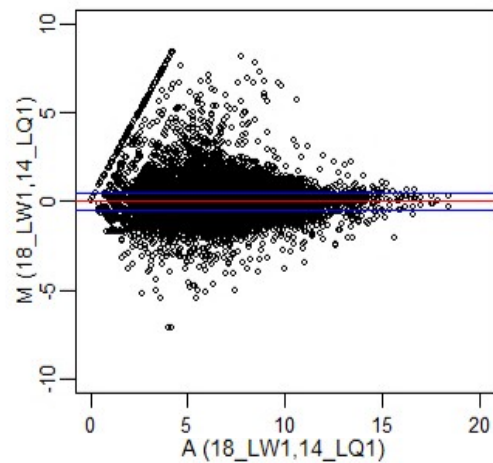

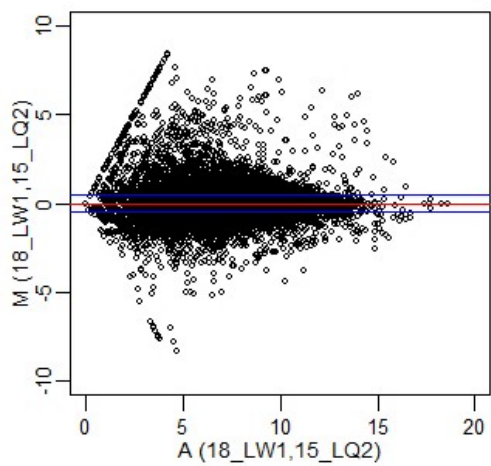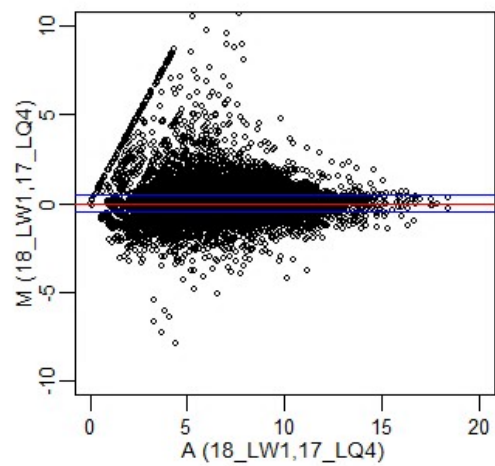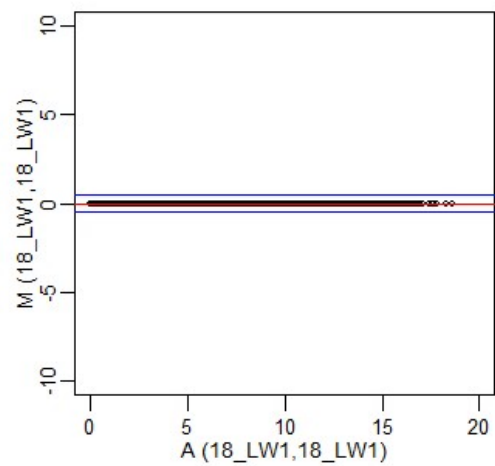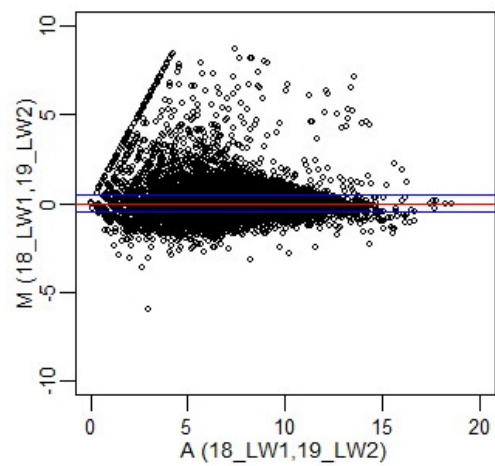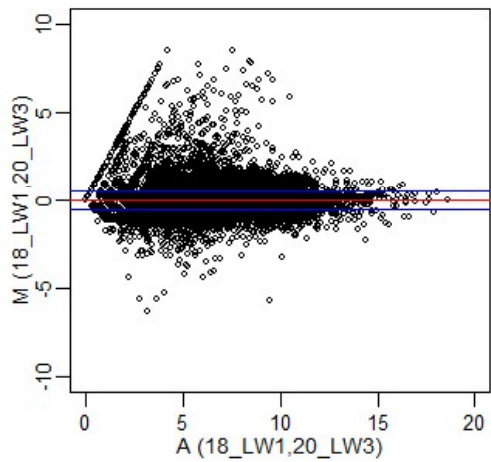



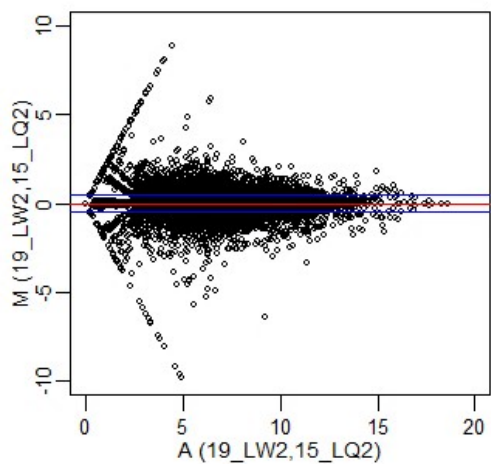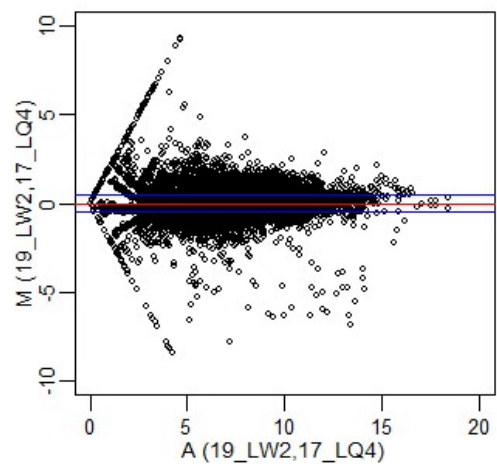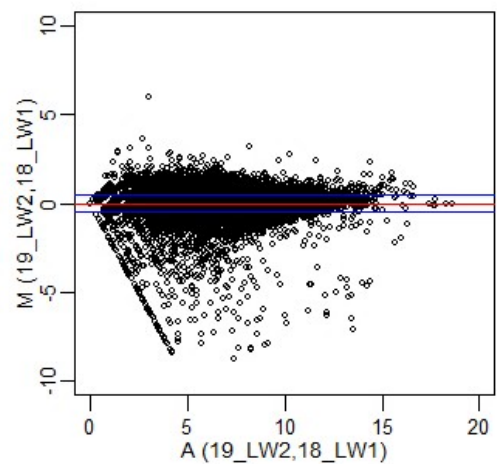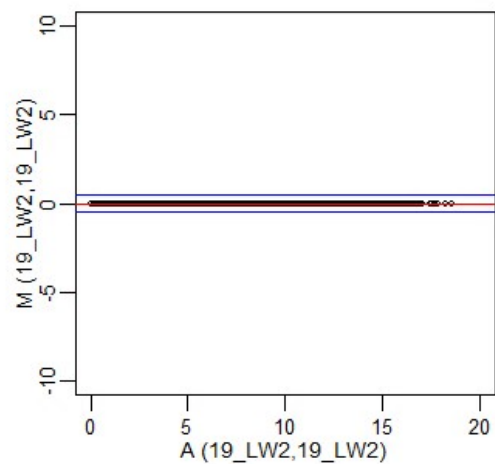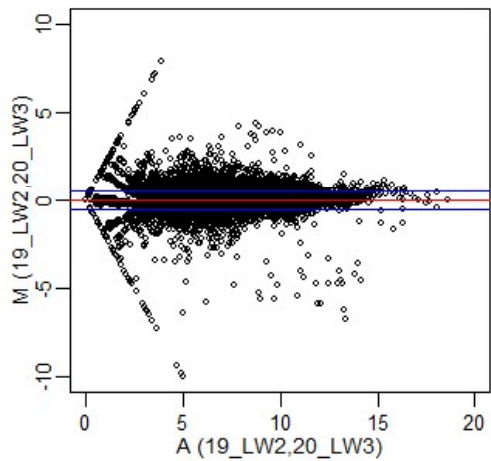



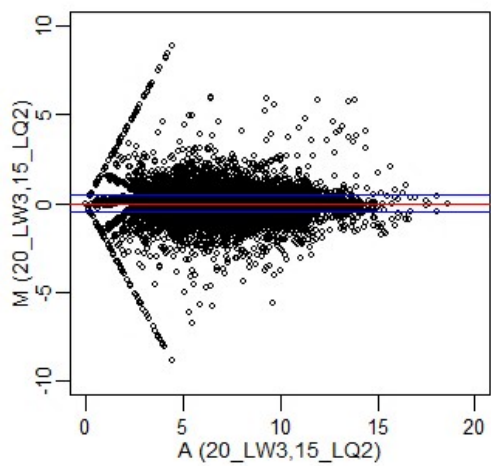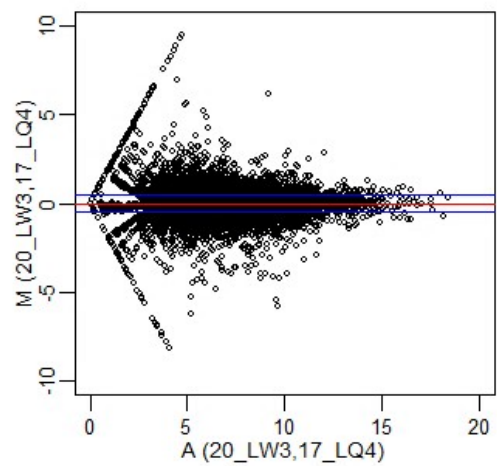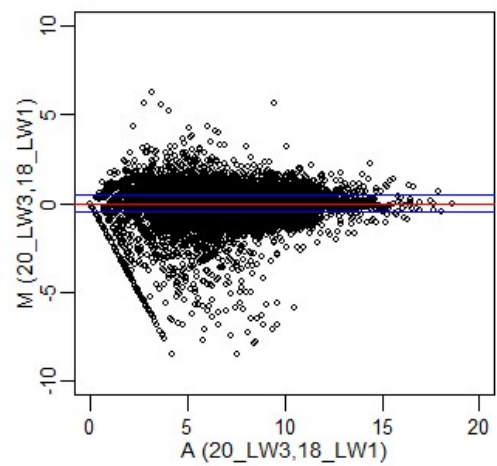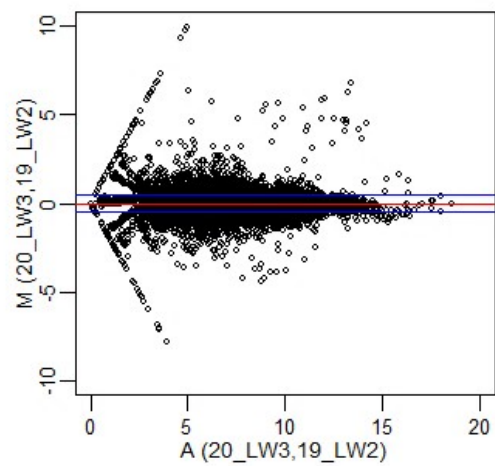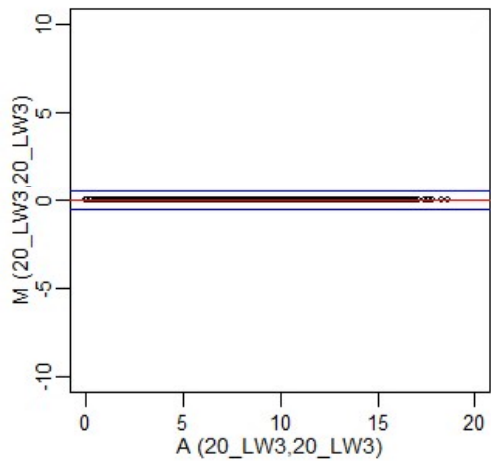

Supplement: Supplementary file 3 — Figure S8 [file MEC-30-718-s003.pdf]
